# Ansatz-Independent Variational Quantum Classifiers and the Price of Ansatz

**DOI:** 10.1038/s41598-022-20688-5

**Published:** 2022-11-14

**Authors:** Hideyuki Miyahara, Vwani Roychowdhury

**Affiliations:** Department of Electrical and Computer Engineering, Henry Samueli School of Engineering and Applied Science, University of California, Los Angeles, CA 90095 USA

**Keywords:** Engineering, Mathematics and computing, Physics

## Abstract

The paradigm of variational quantum classifiers (VQCs) encodes *classical information* as quantum states, followed by quantum processing and then measurements to generate classical predictions. VQCs are promising candidates for efficient utilizations of noisy intermediate scale quantum (NISQ) devices: classifiers involving *M*-dimensional datasets can be implemented with only $\lceil \log _2 M \rceil$ qubits by using an amplitude encoding. A general framework for designing and training VQCs, however, is lacking. An encouraging specific embodiment of VQCs, quantum circuit learning (QCL), utilizes an ansatz: a circuit with a predetermined circuit geometry and parametrized gates expressing a time-evolution unitary operator; training involves learning the gate parameters through a gradient-descent algorithm where the gradients themselves can be efficiently estimated by the quantum circuit. The representational power of QCL, however, depends strongly on the choice of the ansatz, as it limits the range of possible unitary operators that a VQC can search over. Equally importantly, the landscape of the optimization problem may have challenging properties such as barren plateaus and the associated gradient-descent algorithm may not find good local minima. Thus, it is critically important to estimate (i) the price of ansatz; that is, the gap between the performance of QCL and the performance of ansatz-independent VQCs, and (ii) the price of using quantum circuits as classical classifiers: that is, the performance gap between VQCs and equivalent classical classifiers. This paper develops a computational framework to address both these open problems. First, it shows that VQCs, including QCL, fit inside the well-known kernel method. Next it introduces a framework for efficiently designing ansatz-independent VQCs, which we call the unitary kernel method (UKM). The UKM framework enables one to estimate the first known computationally-determined bounds on both the price of ansatz and the price of any speedup advantages of VQCs: numerical results with datatsets of various dimensions, ranging from 4 to 256, show that the ansatz-induced gap can vary between 10 and 20$\%$, while the VQC-induced gap (between VQC and kernel method) can vary between 10 and 16$\%$. To further understand the role of ansatz in VQCs, we also propose a method of decomposing a given unitary operator into a quantum circuit, which we call the variational circuit realization (VCR): given any parameterized circuit block (as for example, used in QCL), it finds optimal parameters and the number of layers of the circuit block required to approximate any target unitary operator with a given precision.

## Introduction

Since the discovery of Shor’s algorithm^1^, much effort has been devoted to the development of quantum algorithms and quantum computers^2^. To exploit a near-term quantum device, several variational quantum algorithms (VQAs)^3^ have been proposed, including the quantum approximate optimization algorithm (QAOA)^4^ and the variational quantum eigensolver (VQE)^5^. Then, quantum circuit learning (QCL) was proposed in Refs.^6,7^ and is now considered to be a promising candidate to utilize near-term quantum devices for implementing efficient solutions of machine learning (ML) tasks. QCL itself, however, is a special case of a larger set of hybrid quantum-classical classifiers – a class that we refer to as variational quantum classifiers (VQCs) – since it assumes an ansatz, where the circuit geometry is fixed and only the gates are parameterized. Thus, several questions remain unanswered, including (i) whether one can get better performance than QCL by systematically designing an ansatz-independent VQC, (ii) given that a VQC and QCL perform end-to-end classical machine learning (ML) tasks, whether they are related to well-known classical ML algorithms that perform better. Furthermore, any ansatz-independent upper bound of the performance of QCL is of great interest since the performance of QCL itself heavily depends on both an ansatz and on an optimization method.

In this paper, we first discuss the correspondence between a VQC and the well-known kernel method^8,9^. Then we propose an ansatz-independent VQC, which we call the unitary kernel method (UKM). By using the UKM, we present ansatz-independent upper bounds on the performance of QCL for a wide range of classification tasks, i.e., the price paid by any chosen ansatz, as well as by the use of the gradient-descent algorithm for learning the parameters of the chosen ansatz. Next, we construct QCL-type circuits that could implement the unitary operator computed by the UKM. Since the UKM computes an ansatz-independent unitary evolution operator (hence, computable by a quantum circuit), it provides a tighter bound on QCL than obtained by the classical kernel method. It also provides an estimate of the gap between a VQC and a classical kernel-method classifier.

To effectively use the unitary operator obtained by the UKM, we propose a unitary decomposition method to create a circuit geometry, which we call the variational circuit realization (VCR). By combining the UKM and the VCR, we can efficiently construct a circuit geometry that works well for classification problems.

In the rest of the paper we also use the term *quantum advantage* to capture both, (i) any potential gain in performance over classical ML algorithms, and (ii) any potential gain in hardware efficiency. For example, in the case of amplitude encoding, the number of qubits used is logarithmic in the dimension of the data. Thus, even if a classical ML algorithm performs better, VQCs can still have an advantage: when quantum devices become cheap and well-developed, it could lead to practical methods for implementing high dimensional classification problems.

**Figure 1:**
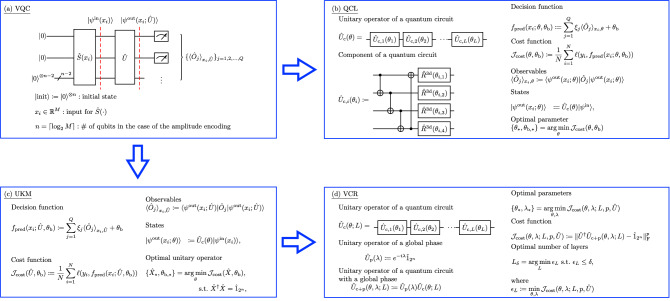
Schematic of the algorithms discussed in this paper: (**a**) A general form of a hybrid quantum-classical classifier, which we refer to as a VQC, (**b**) QCL, (**c**) UKM, and (**d**) VCR. (**a**) In the architecture of a VQC, the initial state is $| \mathrm {init} \rangle :=| 0 \rangle ^{\otimes n}$. We first encode a given classical vector $x_i$: $| \psi ^\mathrm {in} (x_i) \rangle :=\hat{S} (x_i) | \mathrm {init} \rangle$. One can embed $x_i$ into a higher dimensional vector $\phi (x_i) \in {\mathbb {R}}^L$ with $L= {\mathcal {O}} (M^c)$ and then use the rest of the framework; the number of qubits *n* will still be ${\mathcal {O}} (\log M)$, thus retaining any potential quantum advantage. Second, we apply $\hat{U}$: $| \psi ^\mathrm {out} (x_i; \hat{U}) \rangle :=\hat{U} | \psi ^\mathrm {in} (x_i) \rangle$. Third, we perform measurements with respect to $\{ \hat{O}_j \}_{j=1, 2, \dots , Q}$. Finally, we make a prediction on the label of $x_i$ by using the outputs of the measurements. (**b**) In QCL, we assume a circuit geometry parameterized by $\theta$ for $\hat{U}$: $\hat{U}_\mathrm {c} (\theta )$. In most cases, a circuit used for QCL is composed of single- and two-qubit operators and has a layered structure. A typical example is shown. (**c**) In the UKM, we directly optimize $\hat{U}$. (**d**) In the VCR, we decompose a unitary operator into a quantum circuit by assuming a layered structure for a quantum circuit. For a circuit realization, a simpler circuit is preferable; so, we explicitly denote the number of layers *L*.

Figure 1 presents a schematic of a general VQC, introduces and compares QCL and the UKM, and explains the VCR.

## Variational quantum classifier

We first introduce an analytical formalism for a VQC. Suppose that we are given an *n*-qubit system and a classical dataset ${\mathcal {D}} :=\{ x_i, y_i \}_{i=1}^N$, where $x_i \in {\mathbb {R}}^M$ is a feature vector and $y_i \in \{1, -1\}$ is the corresponding label for $i = 1, 2, \dots , N$. In this paper, we consider amplitude encoding^7^; thus we fix $n = \lceil \log _2 M \rceil$. One can embed $x_i$ into a higher dimensional vector $\phi (x_i) \in {\mathbb {R}}^L$ with $L= {\mathcal {O}} (M^c)$ and then use the rest of the framework; the number of qubits *n* will still be ${\mathcal {O}} (\log M)$, thus retaining any potential quantum advantage. Here, ${\mathcal {O}} (\cdot )$ is the big-O notation and *c* is a certain constant. In this paper, however, we stick to amplitude encoding. Let us consider making a prediction on $y_i$ by the following function:

1 $$\begin{aligned} f_\mathrm {pred} (x_i; \hat{U}, \theta _\mathrm {b})&:=\sum _{j=1}^Q \xi _j \langle \hat{O}_j \rangle _{x_i, \hat{U}} + \theta _\mathrm {b}, \end{aligned}$$

where

2 $$\begin{aligned} \left\langle \hat{O}_j \right\rangle _{x_i, \hat{U}} :=\left\langle \psi ^\mathrm {out} \left( x_i; \hat{U}\right) \left| \hat{O}_j \right| \psi ^\mathrm {out} \left( x_i; \hat{U}\right) \right\rangle , \end{aligned}$$

$| \psi ^\mathrm {out} (x_i; \hat{U}) \rangle :=\hat{U} | \psi ^\mathrm {in} (x_i) \rangle$, and *Q* is the number of observables. In the case of $M = 2^n$ [For the details of amplitude encoding, see also Sect. S–S-V of the supplemental material (SM) and Refs.^7,10^. Amplitude encoding in the case of $M \ne 2^n$ is described in Sect. V of the SM.],

3 $$\begin{aligned} | \psi ^\mathrm {in} (x_i) \rangle :=\frac{1}{\sqrt{\sum _{j=1}^M | x_{i, j} |^2}} \sum _{j=1}^M x_{i, j} | j \rangle , \end{aligned}$$

where *n* is the number of qubits. Here, $x_{i, j}$ is the *j*-th element of $x_i$. We denote, by $\hat{S} (x_i)$, the unitary operator that maps $| \mathrm {init} \rangle :=| 0 \rangle ^{\otimes n}$ into $|\psi ^\mathrm {in} (x_i) \rangle$ in Eq. (3): $| \psi ^\mathrm {in} (x_i) \rangle = \hat{S} (x_i) | \mathrm {init} \rangle$. While $\{ \xi _j \}_{j=1}^Q$ can also be learned and optimized, the convention in Refs.^6,7^ is to treat them as fixed parameters and $\theta _\mathrm {b}$ is a bias term to be estimated.

In a VQC, we estimate $\hat{U}$ and $\theta _\mathrm {b}$ in Eq. (1) imposing the unitarity constraint on $\hat{U}$ as follows:

4 $$\begin{aligned} \{ \hat{U}_*, \theta _{\mathrm {b}, *} \}&= {{\,\mathrm{arg\,min}\,}}_{\hat{U}, \theta _\mathrm {b}} {\mathcal {J}}_\mathrm {cost} (\hat{U}, \theta _\mathrm {b}), \nonumber \\&\quad \mathrm {subject \ to} \ \hat{U}^\dagger \hat{U} = \hat{1}_{2^n}, \end{aligned}$$

where

5 $$\begin{aligned} {\mathcal {J}}_\mathrm {cost} (\hat{U}, \theta _\mathrm {b})&:=\frac{1}{N} \sum _{i=1}^N \ell (y_i, f_\mathrm {pred} (x_i; \hat{U}, \theta _\mathrm {b})). \end{aligned}$$

here $\ell (\cdot , \cdot )$ is a loss function, such as the mean-squared error function or the hinge function^8,9^, and $\hat{1}_n$ is the *n*-dimensional identity operator. As explained later, we consider a parameterized unitary operator and optimize it in QCL and we directly optimize the unitary operator in the UKM.

## Correspondence between a VQC and the kernel method

In the conventional kernel method^8,9,11^, a function $\phi (\cdot ): {\mathbb {R}}^P \rightarrow {\mathbb {R}}^G$ is used to map any input data point $z_i \in {\mathbb {R}}^P$ to $\phi (z_i) \in {\mathbb {R}}^G$, and then a linear function is used to make a prediction on $y_i$ by

6 $$\begin{aligned} f_\mathrm {pred} (z_i; v)&:=\sum _{k=1}^G v_k \phi _k (z_i), \end{aligned}$$

where $\phi _k (z_i)$ is the *k*-th element of $\phi (z_i)$, and $v :=[v_1, v_2, \dots , v_G]^\intercal$ is a real vector. For example, in a commonly used degree-2 polynomial kernel function, the products of all the pairs of the coordinates of $z_i$ are used to generate a higher dimensional embedding, along with a constant term. That is, $G = P^2+1$, $\phi _{k + P (l-1)} (z_i) = z_{i, k} \cdot z_{i, l}$, for $k, l = 1, 2, \dots , P$, and finally $\phi _{(P^2+1)} = 1$. With this choice of a kernel function, Eq. (6) can be written as

7 $$\begin{aligned} f_\mathrm {pred} (z_i; v)&:=\sum _{k=1}^{P^2+1} v_k \phi _k (z_i) \end{aligned}$$

8 $$\begin{aligned}&:=\sum _{k, l = 1}^{P} ( z_{i, k} v_{k+P(l-1)} z_{i, l} ) + v_{(P^2+1)}. \end{aligned}$$

Once an embedding has been defined, we minimize the following function to determine *v*:

9 $$\begin{aligned} {\mathcal {J}}_\mathrm {cost} (v)&:=\frac{1}{N} \sum _{i=1}^N \ell (y_i,f_\mathrm {pred} (z_i; v)). \end{aligned}$$

We show next how the VQC problem in Eq. (4) can be mapped to the above kernel form, i.e. any solution obtained by a VQC is a constrained solution of a corresponding kernel based classifier. Thus, the performance of a suitably defined kernel method – without any constraints on $\{ v_k \}_k$ – will always provide an upper bound on the performance of a VQC, including classifiers based on QCL. In the case of VQCs, we have $P=2^n$. Introducing $\psi _l^\mathrm {in} (x_i) :=\langle l | \psi ^\mathrm {in} (x_i) \rangle$, $O_{j, (k, l)} :=\langle k | \hat{O}_j | l \rangle$, and $u_{k, l} :=\langle k | \hat{U} | l \rangle$ for $k, l = 1, 2, \dots , 2^n$, $\langle \hat{O}_j \rangle _{x_i, \hat{U}}$, introduced in Eq. (2), can be rewritten as

10 $$\begin{aligned} \langle \hat{O}_j \rangle _{x_i, \hat{U}} = \sum _{k, l = 1}^{2^n} \psi _k^\mathrm {in} (x_i) w_{j, (k, l)} \psi _l^\mathrm {in} (x_i), \end{aligned}$$

where, for $$k, l = 1, 2, \dots , 2^n$$,

11 $$\begin{aligned} w_{j, (k, l)}&:=\sum _{k', l' = 1}^{2^n} u_{k, k'}^* O_{j, (k', l')} u_{l', l}, \end{aligned}$$

$u_k :=[u_{1, k}, u_{2, k}, \dots , u_{2^n, k}]^\mathrm {H}$ for $k = 1, 2, \dots , 2^n$ ($(\cdot )^\mathrm {H}$ is the Hermitian conjugate), and $u_k^\mathrm {H} u_l = \delta _{k, l}$.

By using Eqs. (10) and (11), the VQC prediction function in Eq. (1) can be written as

12 $$\begin{aligned} f_\mathrm {pred} (x_i; \hat{U}, \theta _\mathrm {b})&:=\sum _{k, l = 1}^{2^n} \psi _k^\mathrm {in} (x_i) \bigg ( \sum _{j=1}^Q \xi _j w_{j, (k, l)} \bigg ) \psi _l^\mathrm {in} (x_i) + \theta _\mathrm {b}. \end{aligned}$$

Now, if we compare the VQC prediction function in (12), to the kernel method prediction function in (8), we get a direct correspondence, where a VQC is reduced to a constrained version of the kernel method, and thus, the kernel method provides an upper bound on the performance of VQCs. Formally, the following choice of $\phi _m (\cdot )$ and $v_m$ in (6) is required [The kernel method is discussed in Sect. S-VI of the SM and the relationship between a VQC and the kernel method is discussed in Sect. S-VII of the SM in detail.]: for $i = 1, 2, \dots , 2^n$, $z_i = \psi ^\mathrm {in} (x_i)$, for $k, l = 1, 2, \dots , 2^n$,

13 $$\begin{aligned} \phi _{k + (l-1) 2^n} (x_i)&= \psi _k^\mathrm {in} (x_i) \psi _l^\mathrm {in} (x_i), \end{aligned}$$

14 $$\begin{aligned} v_{k + (l-1) 2^n}&= \sum _{j=1}^Q w_{j, (k, l)}, \end{aligned}$$

and

15 $$\begin{aligned} \phi _{2^{2n}+1}&= 1, \end{aligned}$$

16 $$\begin{aligned} v_{2^{2n}+1}&= \theta _\mathrm {b}. \end{aligned}$$

Furthermore, we have $P=2^n$ and $G=2^{2n}+1$.

Another advantage of showing this relationship is that it helps us benchmark how well a VQC optimization method performs: since the kernel method is an upper bound, if the VQC attains performs very close to that of the kernel method, then it would show that it is performing at its highest capacity.

## Quantum circuit learning

Here, we review QCL proposed in Refs.^6,7^ from the viewpoint of a VQC. In QCL, we assume a parameterized unitary operator $\hat{U}_\mathrm {c} (\theta )$ [Both $\hat{U}_\mathrm {c} (\theta )$ and $\hat{U}_\mathrm {c} (\theta ; L)$ are used to denote a unitary operator realized by a quantum circuit; but we use $\hat{U}_\mathrm {c} (\theta ; L)$ when we want to explicitly emphasize the number of layers *L*.] as $\hat{U}$ and optimize $\theta$ [Refer to Sect. S-V B of the SM for the details of quantum circuits.]. We then compute $| \psi ^\mathrm {out} (x_i; \theta ) \rangle :=\hat{U}_\mathrm {c} (\theta ) | \psi ^\mathrm {in} (x_i) \rangle$. Then, we make a prediction on $x_i$ by

17 $$\begin{aligned} f_\mathrm {pred} (x_i; \theta , \theta _\mathrm {b})&:=\sum _{j=1}^Q \xi _j \langle \hat{O}_j \rangle _{x_i, \theta } + \theta _\mathrm {b}, \end{aligned}$$

where $\langle \hat{O}_j \rangle _{x_i, \theta } :=\langle \psi ^\mathrm {out} (x_i; \theta ) | \hat{O}_j | \psi ^\mathrm {out} (x_i; \theta ) \rangle$. Similarly to Eq. (1), $\{ \xi _j \}_{j=1}^Q$ are fixed parameters and $\theta _\mathrm {b}$ is a bias term to be estimated. The second step of QCL is to update $\theta$ and $\theta _\mathrm {b}$ by

18 $$\begin{aligned} \{ \theta _*, \theta _{\mathrm {b}, *} \}&= {{\,\mathrm{arg\,min}\,}}_{\theta , \theta _\mathrm {b}} {\mathcal {J}}_\mathrm {cost} (\theta , \theta _\mathrm {b}), \end{aligned}$$

where

19 $$\begin{aligned} {\mathcal {J}}_\mathrm {cost} (\theta , \theta _\mathrm {b})&:=\frac{1}{N} \sum _{i=1}^N \ell (y_i, f_\mathrm {pred} (x_i; \theta , \theta _\mathrm {b})), \end{aligned}$$

and $\ell (\cdot , \cdot )$ is a loss function [For details, refer to Sec. S-V of the SM.]. For this purpose, we often use the Nelder-Mead method^12^ and other sophisticated numerical methods^13,14^.

As mentioned above, QCL assumes a parameterized unitary operator $\hat{U}_\mathrm {c} (\theta )$; thus, its performance heavily depends on the circuit geometry of $\hat{U}_\mathrm {c} (\theta )$. An assumed circuit geometry is also called an ansatz; thus we can say that QCL is an ansatz-dependent VQC. This fact strongly motivates us to devise an ansatz-independent VQC, that is, the UKM. Furthermore, Ref.^15^ pointed out the difficulty of learning parameters of quantum circuits, which they call the barren plateau problem. Then, a VQC that is free of the barren plateau problem is of interest.

## Unitary kernel method

We here describe the UKM, which is one of the main algorithms in this paper. In the UKM, we directly minimize Eq. (5). To this end, we employ the unitary version of the method of splitting orthogonal constraints (SOC)^16^. Hereafter, we denote, by $\hat{X}$, an operator obtained via the method of SOC. We introduce $\hat{P}$ and $\hat{D}$ and iterate update equations for $\hat{X}$, $\hat{P}$, and $\hat{D}$ until convergence. Furthermore, we denote $\hat{X}$, $\hat{P}$, $\hat{D}$, and $\theta _\mathrm {b}$ at the *k*-th iteration by $\hat{X}_k$, $\hat{P}_k$, $\hat{D}_k$, and $\theta _{\mathrm {b}, k}$, respectively. At the first step of the *k*-th iteration, we compute $\hat{X}_k$ and $\theta _{\mathrm {b}, k}$ by

20 $$\begin{aligned} \left\{ \hat{X}_k, \theta _{\mathrm {b}, k} \right\}&= {{\,\mathrm{arg\,min}\,}}_{\hat{X}, \theta _\mathrm {b}} {\mathcal {J}}_\mathrm {UKM} \left( \hat{X}, \theta _\mathrm {b}; \hat{P}_{k-1}, \hat{D}_{k-1}\right) , \end{aligned}$$

where

21 $$\begin{aligned} {\mathcal {J}}_\mathrm {UKM} \left( \hat{X}, \theta _\mathrm {b}; \hat{P}, \hat{D}\right)&:={\mathcal {J}}_\mathrm {cost} \left( \hat{X}, \theta _\mathrm {b}\right) + \frac{r}{2} \Vert \hat{X} - \hat{P} + \hat{D} \Vert _\mathrm {F}^2. \end{aligned}$$

To solve Eq. (20), we optimize the real and complex parts of $\hat{X}_k$ independently [See Sect. S-IX B of the SM for details.]. Next, we compute $\hat{P}_k$ by

22 $$\begin{aligned} \hat{P}_k&= \hat{K}_{1, k} \hat{K}_{2, k}^\dagger , \end{aligned}$$

where $\hat{K}_{1, k}$ and $\hat{K}_{2, k}^\dagger$ are unitary operators that satisfy $\hat{K}_{1, k} \hat{\Sigma }_k \hat{K}_{2, k}^\dagger = \hat{X}_k + \hat{D}_{k-1}$ and $\hat{\Sigma }_k$ is a diagonal operator. At the end of the *k*-th iteration, we compute

23 $$\begin{aligned} \hat{D}_k&= \hat{D}_{k-1} + \hat{X}_k - \hat{P}_k. \end{aligned}$$

We repeat the above equations, Eqs. (20), (22), and (23), until convergence. We call this method the UKM. In Algorithm 1, the UKM is summarized [For the details of the UKM, refer to Sect. S-IX A of the SM.].

It is clear from the formulation of the method of SOC that $\hat{X}$ does not strictly satisfy the unitarity constraint; instead, $\hat{P}$ and OU of $\hat{X}$ does. Thus, using the optimal value of $\hat{X}$ obtained from the UKM leads to a classical classifier (it cannot be implemented using a quantum circuit), and will in general have higher performance than the unitary operators given by $\hat{P}$ and OU of $\hat{X}$ that approximate $\hat{X}$. Thus, we compute the success rates for the training and test datasets by using all the versions: $\hat{X}$, $\hat{P}$, and OU of $\hat{X}$ [OU is explained in Sect. S-IX A of the SM.], of which only $\hat{P}$ and OU of $\hat{X}$ correspond to VQCs.

## Variational circuit realization

There are some studies on decomposing unitary operators into quantum circuits^17–19^, including Knill’s decomposition and the quantum Shannon decomposition (QSD). In these methods, however, the number of the CNOT gates scales quadratically in *M*.

Here we propose an alternate method: the assumed circuit is comprised of *L* layers of a parameterized sub-circuit with parameterized gates and a fixed circuit geometry; similar to the ansatz used in QCL. We then solve for the minimum number of layers *L*, such that the optimized circuit approximates the given unitary operator with a specified precision of $\delta$. We refer to this circuit methodology as the VCR. The schematic of the VCR is demonstrated in Fig. 1d. Let $\hat{U}$ and $\hat{U}_\mathrm {c} (\theta ; L)$ be a target unitary operator and a unitary operator realized by a quantum circuit that is parametrized by $\theta$ and has *L* layers, respectively. Typically, the target unitary operator is obtained by the UKM discussed above. Furthermore, we define the global phase unitary operator $\hat{\Phi }_{2^n} (\lambda ) :=e^{- i \lambda } \hat{1}_{2^n}$. When $\hat{U}$ and $\hat{U}_\mathrm {c+p} (\theta , \lambda ; L) :=\hat{\Phi }_{2^n} (\lambda ) \hat{U}_\mathrm {c} (\theta ; L)$ are identical, we have

24 $$\begin{aligned} \hat{U}^\dagger \hat{U}_\mathrm {c+p} (\theta , \lambda ; L)&= \hat{1}_{2^n}. \end{aligned}$$

Then, we can estimate $\theta$, for any $p > 0$, by

25 $$\begin{aligned} \{ \theta _*, \lambda _* \}&= {{\,\mathrm{arg\,min}\,}}_{\theta , \lambda } {\mathcal {J}}_\mathrm {cost} \left( \theta , \lambda ; L, p, \hat{U}\right) , \end{aligned}$$

where

26 $$\begin{aligned} {\mathcal {J}}_\mathrm {cost} \left( \theta , \lambda ; L, p, \hat{U}\right)&:=\Vert \hat{U}^\dagger \hat{U}_\mathrm {c+p} \left( \theta , \lambda ; L\right) - \hat{1}_{2^n} \Vert _\mathrm {F}^p. \end{aligned}$$

In a circuit realization, the complexity of a circuit is of great interest. In this paper, we assume a layered structure for a quantum circuit. Thus, given an error threshold $\delta$, it is convenient to define $L_\delta$:

27 $$\begin{aligned} L_\delta&:={{\,\mathrm{arg\,min}\,}}_L \epsilon _L, \nonumber \\&\quad \mathrm {subject \ to} \ \epsilon _L \le \delta , \end{aligned}$$

where

28 $$\begin{aligned} \epsilon _L&:=\min _{\theta , \lambda } {\mathcal {J}}_\mathrm {cost} (\theta , \lambda ; L, p, \hat{U}). \end{aligned}$$

## Numerical simulation

We first show the numerical results of QCL and the UKM for the cancer dataset (0 or 1) [The iris dataset in the UCI repository^20^ has two labels: (0) ‘B’ and (1) ‘M.’ In the cancer dataset (0 or 1), we consider the classification problem between the 0 label and the 1 label. Furthermore, we relabel 0 with $-1$ to adjust labels with the eigenvalues of $\hat{\sigma }_z$. For the numerical results for other datasets, refer to Sect. S-IX B of the SM.] in the UCI repository^20^. The results for multiple datasets with different dimensions, *M*, are presented in Table 1.

**Table 1:** Results of 5-fold CV with 5 different random seeds of the UKM ($\hat{X}$, $\hat{P}$, and OU of $\hat{X}$), QCL, and the kernel method for all the datasets. The numbers of data points *N* and dimensions *M* of the datasets are shown. The number of qubits *n* required for amplitude encoding is also shown. Note that $n = \lceil \log _2 M \rceil$.

				Variational quantum classifiers–			Classical classifiers	
Dataset	*N*	*M*	*n*	UKM ($\hat{P}$)	UKM (OU of $\hat{X}$)	QCL	UKM ($\hat{X}$)	Kernel method
Iris (0 or 1)	100	4	2	1.0000/**1.0000**	1.0000/**1.0000**	1.0000/**1.0000**	1.0000/1.0000	1.0000/1.0000
Iris (0 or non-0)	150	4	2	1.0000/0.9987	1.0000/**1.0000**	1.0000/**1.0000**	1.0000/1.0000	1.0000/1.0000
Iris (1 or non-1)	150	4	2	0.7880/0.7789	0.7953/**0.7994**	0.6801/0.5872	0.9781/0.9618	0.9751/0.9666
Cancer (0 or 1)	569	30	5	0.9194/**0.9131**	0.9184/0.9115	0.8797/0.8768	0.9218/0.9160	0.9618/0.9568
Sonar (0 or 1)	208	60	6	0.9159/**0.7985**	0.9175/0.7909	0.7455/0.6924	0.8903/0.7774	1.0000/0.8198
Wine (0 or non-0)	178	14	4	0.9200/**0.9185**	0.9212/0.9171	0.9155/0.9126	0.9364/0.9313	0.9987/0.9955
Semeion (0 or 1)	323	256	8	1.0000/0.9943	1.0000/**0.9945**	0.9210/0.9099	1.0000/0.9957	1.0000/1.0000
Semeion (0 or non-0)	1593	256	8	0.9988/0.9949	0.9990/**0.9953**	0.8989/0.8982	0.9969/0.9925	1.0000/0.9955
MNIST256 (0 or 1)	569	256	8	0.9991/**0.9969**	1.0000/0.9951	0.9511/0.9459	0.9985/0.9966	1.0000/1.0000
MNIST256 (0 or non-0)	2766	256	8	0.9922/0.9871	0.9927/**0.9889**	0.9053/0.9050	0.9894/0.9859	0.9992/0.9953

The performance cells are of the format “training performance/test performance.” We choose the model that shows the best test performance for each algorithm. For the UKM, we consider the complex and real cases with and without the bias term. We set $r = 0.010$. For QCL, we consider the CNOT-based, CRot-based, 1d-Heisenberg, and FC-Heisenberg circuits with and without the bias term for the iris, cancer, sonar, and wine datasets, and the CNOT-based and CRot-based circuits with and without the bias term for the semeion and MNIST256 datasets. We set the number of layers *L* to 5. For $\phi (\cdot )$ in the kernel method, we consider linear and quadratic functions with and without the bias term for $\lambda = 10^{-2}, 10^{-1}, 1$. The values of the best VQC for each dataset are printed in bold.

Before getting into the numerical results, we state the numerical setup [For the details of numerical settings, refer to Sect. S-IX A of the SM.]. For the UKM, we put $r = 0.010$ and set $K = 30$ in Algo. 1. Furthermore, we use the conjugate gradient (CG) method to find the solution of Eq. (20) and run the CG iteration 10 times [Refer to Sect. S-IX B of the SM for the details of the CG method and Sect. S-IX C of the SM for the details of the UKM with the CG method.]. The UKM can be programmed to yield both real and complex unitary matrices and hence, we consider the performance for both cases separately; see the appendix and the SM. For QCL, we consider four types of quantum circuits: the CNOT-based circuit, the CRot-based circuit, the 1-dimensional (1d) Heisenberg circuit, and the fully-connected (FC) Heisenberg circuit [The definitions of the CNOT-based circuit, the CRot-based circuit, the 1d Heisenberg circuit, and the FC Heisenberg circuit are given in Sect. S-V B of the SM.], and run iterations 300 times. To accelerate QCL, we utilize the stochastic gradient descent method^9^. In both cases, we use the squared error function $\ell _\mathrm {SE} (a, b) :=\frac{1}{2} | a - b |^2$ for $\ell (\cdot , \cdot )$ in Eqs. (5) and (19), and set $Q = 1$ and $\xi _1 = 1$ in Eqs. (1) and (17). Furthermore, we consider two cases with the bias term and without the bias term in Eqs. (1) and (17). Note that we use the **optimize** function provided in the SciPy package^21^ for the implementation of the UKM and the Pennylane package^22^ for QCL. Because of the nature of SOC, the performance of the solutions often oscillates; thus, we run the UKM for a certain number of iterations and choose the solution of the best performance. Then we summarize the results of 5-fold cross-validation (CV) with 5 different random seeds of QCL and the UKM in Tables 2 and 3, respectively. For each method, we select the best model for the training dataset over iterations to compute the performance.

**Table 2:** Results of 5-fold CV with 5 different random seeds for the cancer dataset (0 or 1).

Algo.	Condition	Training	Test
QCL	CNOT-based, w/o bias	0.8797	0.8768
QCL	CNOT-based, w/ bias	0.8597	0.8577
QCL	CRot-based, w/o bias	0.7866	0.7752
QCL	CRot-based, w/ bias	0.8085	0.8052
QCL	1d Heisenberg, w/o bias	0.6568	0.6512
QCL	1d Heisenberg, w/ bias	0.7515	0.7427
QCL	FC Heisenberg, w/o bias	0.7435	0.7444
QCL	FC Heisenberg, w/ bias	0.7744	0.7789

The number of layers *L* is 5 and the number of iterations is 300. We consider four types of circuits with and without the bias term: the CNOT-based circuit, the CRot-based circuit, 1d Heisenberg circuit, and the FC Heisenberg circuit. As shown in Fig. 3, increasing the number of layers *L* does not lead to better performance, and can in fact decrease performance of QCL.

**Table 3:** Results of 5-fold cross-validation (CV) with 5 different random seeds for the cancer dataset (0 or 1).

Algo.	Condition	Training	Test
UKM	$\hat{X}$, complex, w/o bias	0.9219	0.9143
UKM	$\hat{P}$, complex, w/o bias	0.9204	0.9093
UKM	OU of $\hat{X}$, complex, w/o bias	0.9184	0.9115
UKM	$\hat{X}$, complex, w/ bias	0.9207	0.9143
UKM	$\hat{P}$, complex, w/ bias	0.8870	0.8753
UKM	OU of $\hat{X}$, complex, w/ bias	0.8912	0.8805
UKM	$\hat{X}$, real, w/o bias	0.9213	0.9107
UKM	$\hat{P}$, real, w/o bias	0.9194	0.9131
UKM	OU of $\hat{X}$, real, w/o bias	0.9170	0.9112
UKM	$\hat{X}$, real, w/ bias	0.9218	0.9160
UKM	$\hat{P}$, real, w/ bias	0.7929	0.7879
UKM	OU of $\hat{X}$, real, w/ bias	0.8107	0.8014

We show the performance obtained by $\hat{X}$, $\hat{P}$, and OU of $\hat{X}$. We consider real and complex matrices for the initial input with and without the bias term. We set $r = 0.010$ and $K = 30$. We repeat the CG iteration for Eq. (20) 10 times in each step of the method of SOC. Due to the inherent formulation of the method of SOC, $\hat{X}$ does not strictly satisfy the unitarity condition; $\hat{P}$ and OU of $\hat{X}$ strictly satisfy the unitarity condition, yielding VQCs. The overall higher performance of $\hat{X}$ can be attributed to it being a classical classifier; a special case of the kernel method. Note, however, that the classifier created by the UKM without bias yield better performance than the best classifiers created by QCL, as shown in Table 2.

**Figure 2:**
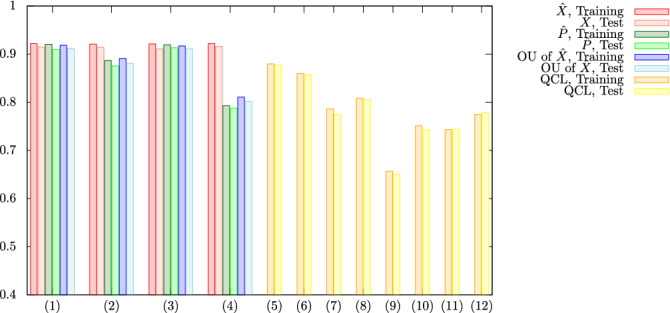
Results of 5-fold CV with 5 different random seeds for the cancer dataset (0 or 1). For the UKM, we put $r = 0.010$ and $K = 30$; see the appendix for the definitions of *r* and *K*. We repeat the CG iteration for Eq. (20) 10 times in each step of the method of SOC. For QCL, the number of layers *L* is 5 and the number of iterations is 300. The numerical settings are as follows: (1) UKM: complex matrix without bias, (2) UKM: complex matrix with bias, (3) UKM: real matrices without the bias term, (4) UKM: real matrices with the bias term, (5) QCL: CNOT-based circuit without the bias term, (6) QCL: CNOT-based circuit with the bias term, (7) QCL: CRot-based circuit without the bias term, (8) QCL: CRot-based circuit with the bias term, (9) QCL: 1d Heisenberg circuit without the bias term, (10) QCL: 1d Heisenberg circuit with the bias term, (11) QCL: FC Heisenberg circuit without the bias term, and (12) QCL: FC Heisenberg circuit with the bias term.

In Fig. 2, we plot the data shown in Tables 2 and 3.

As shown in Fig. 2, the performance of the UKM is better that of QCL in several numerical setups.

Given our analytical results showing that the kernel method is a superset of VQCs, we next present the performance of the kernel method [Particularly, we use Ridge classification as the kernel method. In Ridge classification, we use the squared error function $\ell _\mathrm {SE} (\cdot , \cdot )$ for $\ell (\cdot , \cdot )$ in ${\mathcal {J}}_\mathrm {cost} (v) :=\frac{1}{N} \sum _{i=1}^N \ell (y_i, f_\mathrm {pred} (x_i; v)) + \frac{\lambda }{2} \Vert v \Vert _\mathrm {F}^2$. For the details of the kernel method, see Sect. S-VI of the SM. More specifically, Ridge classification is described in Sect. S-VI B of the SM. Refs.^8,9^ are also helpful.]. We set $\lambda = 10^{-1}$, which is the coefficient of the regularization term, and consider linear and quadratic functions for $\phi (\cdot )$ with and without normalization. The norm of the vector of each data point is not unity. Normalization means that we normalize the vector of each data point before performing classification. The purpose is to see the effect of normalization incorporated into amplitude encoding though the original setup of classification does not have the process. Note that we use the scikit-learn package^23^ for the kernel method. Then we summarize the results of 5-fold CV with 5 different random seeds of the kernel method in Table 4.

**Table 4:** Results of 5-fold CV with 5 different random seeds of the kernel method for the cancer dataset (0 or 1).

Algo.	Condition	Training	Test
Kernel	Linear, w/o normalization	0.9623	0.9549
Kernel	Linear, w/ normalization	0.9205	0.9176
Kernel	Quadratic, w/o normalization	0.9936	0.9361
Kernel	Quadratic, w/ normalization	0.9210	0.9195

We set $\lambda = 10^{-1}$. For $\phi (\cdot )$, we use linear and quadratic functions with and without normalization.

For some $\lambda$, the performance of the kernel method is better than QCL and the UKM, as expected.

**Figure 3:**
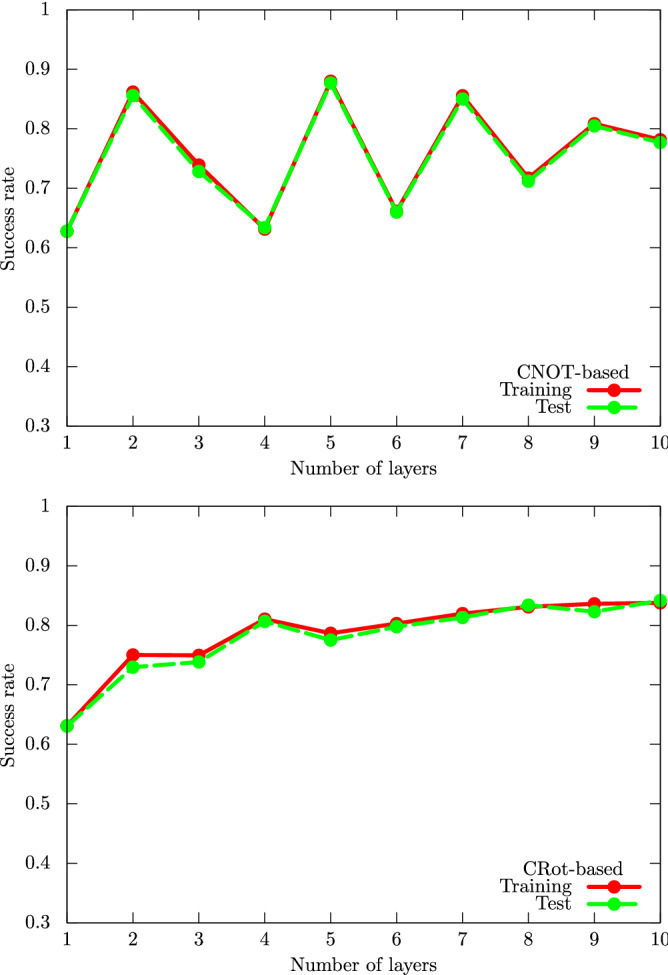
Performance dependence of QCL on the number of layers *L* for the cancer dataset (0 or 1). We use the CNOT-based (upper panel) and CRot-based (lower panel) circuit geometries and set $\theta _\mathrm {bias} = 0$. We iterate the computation 300 times. Note that, for any *L*, the CRot-based circuit has inherently more expressive power than the CNOT-based circuit: just fix the controlled versions of the 3-dimensional rotation gate to the CNOT gates. The fact that performance in the lower panel is worse than that in the upper panel indicates the optimization problems faced in QCL.

Next, we explore the performance dependence of QCL on the number of layers *L*. The result is shown in Fig. 3.

One would naturally expect that increasing the number of layers *L* leads to better performance. In general, a circuit with $L+1$ layers can clearly do at least as well as the circuit with *L* layers: pick the same parameters for the first *L* layers, and choose parameters to create an identity operator with the last layer. But Fig. 3 shows it is not the case. Rather, the test performance gets worse when we increase the number of layers *L*. This variability is potentially related to the structure of the cost function landscape: as the number of parameters is increased by adding an extra layer, there are potentially more local minima or the landscape develops what has been referred to as a “barren plateau” in Ref.^15^.

**Figure 4:**
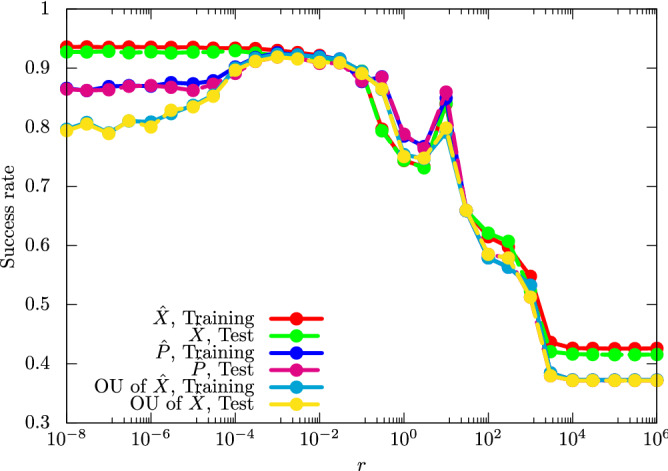
Performance dependence of the UKM on *r*, which is the coefficient of the second term in the right-hand side of Eq. (20) for the cancer dataset (0 or 1). We use complex matrices for the initial input and set $\theta _\mathrm {bias} = 0$. We put $r = 0.010$ and $K = 30$. We repeat the CG iteration for Eq. (20) 10 times in each step of the method of SOC.

We also see the performance dependence of the UKM on *r*, which is the coefficient of the second term in the right-hand side of Eq. (20). The result is shown in Fig. 4.

For small *r*, $\hat{X}$ in the UKM deviates from unitary matrices and the performance gets better. On the other hand, for large *r*, $\hat{X}$ in the UKM becomes closer to unitary matrices but the performance gets worse. Thus, we should choose an appropriate value of *r*.

**Figure 5:**
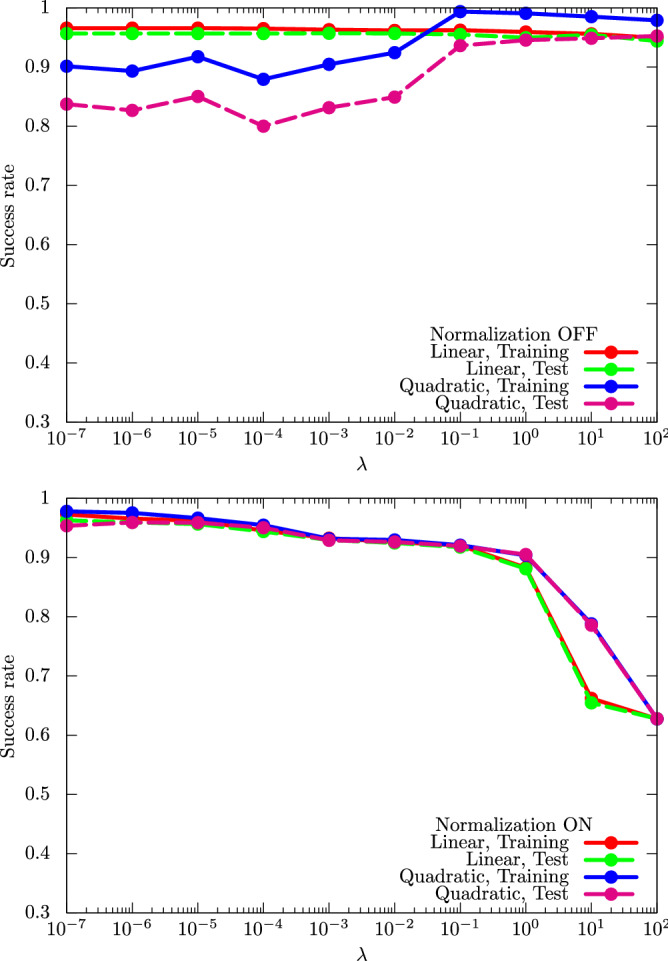
Performance dependence of the kernel method on $\lambda$, which is the coefficient of the regularization term for the cancer dataset (0 or 1). For $\phi (\cdot )$, we use linear and quadratic functions (upper panel) with and (lower panel) without normalization.

In Fig. 5, we show the performance dependence of the kernel method on $\lambda$, which is the coefficient of the regularization term.

Like *r* in the UKM, we also need to choose an appropriate $\lambda$ to realize good performance.

In Table 1, we summarize the performance of QCL, the UKM, and the kernel method for all the datasets investigated in this study. We choose the model that shows the best test performance for each algorithm. For the UKM, we consider the complex and real cases with and without the bias term. We set $r = 0.010$. For QCL, we consider the CNOT-based, CRot-based, 1d-Heisenberg, and FC-Heisenberg circuits with and without the bias term for the iris, cancer, sonar, and wine datasets, and the CNOT-based and CRot-based circuits with and without bias term for the semeion and MNIST256 datasets. We set the number of layers *L* to 5. For $\phi (\cdot )$ in the kernel method, we consider linear and quadratic functions with and without the bias term for $\lambda = 10^{-2}, 10^{-1}, 1$. The numerical results support the claim that the UKM lies between the kernel method and QCL. We also show the detailed numerical results for all the datasets in the SM [In Sect. S-XI of the SM, the numerical results for other datasets are shown.]. The results shown in the SM are consistent with this paper. Finally, we note the difference between the squared error and hinge functions. In this paper, we have used the squared error function; we show the results of the hinge function in the SM. The results are qualitatively same as obtained with the squared error function, and the statements about the relative performances of QCL and the UKM do not change.

**Figure 6:**
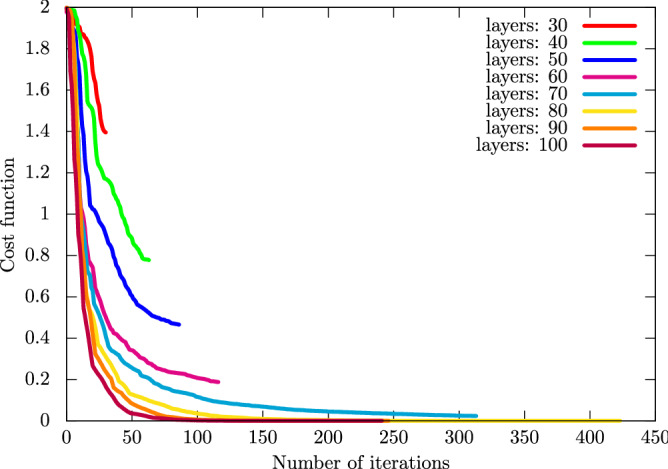
Values of the cost function $J_\mathrm {cost} (\theta , \lambda ; L, 2, \hat{U})$, Eq. (26) with $p = 2$, for the cancer dataset (0 or 1). We set $L = 30, 40, 50, 60, 70, 80, 90, 100$.

We then show numerical simulations on the VCR. Let $\hat{U}_\mathrm {c} (\theta ; L)$ be the unitary operator realized by a quantum circuit that is parametrized by $\theta$ and has *L* layers. For $\hat{U}_\mathrm {c} (\theta ; L)$, we use the CNOT-based circuit. Furthermore, we use the BFGS method^14^ to solve Eq. (25). Note that we use the **optimize** function provided in the SciPy package^21^ for the implementation of the VCR. Here, let us consider the cancer dataset (0 or 1) and minimize Eq. (26) with $p = 2$. As a target unitary operator, we use the unitary operator that gives the success rate for the training dataset 0.9194 and that for the test dataset 0.9131. In Fig. 6, we show the values of the cost function in the right-hand side of Eq. (26) with different numbers of layers *L*. In Table 5, we summarize the performance of the input unitary operator, QCL, and the circuit geometries computed by the VCR.

**Table 5:** Performance of the VCR for the cancer dataset (0 or 1).

Algo.	Condition	Cost	Training	Test
Input	UKM, $\hat{P}$, real, w/o bias	–	0.9139	0.9483
VCR	# of layers: 10	1.9694	0.3929	0.2931
VCR	# of layers: 20	1.9734	0.6071	0.7069
VCR	# of layers: 30	1.3950	0.6071	0.7069
VCR	# of layers: 40	0.7777	0.6909	0.7586
VCR	# of layers: 50	0.4657	0.8499	0.9224
VCR	# of layers: 60	0.1877	0.9073	0.9483
VCR	# of layers: 70	0.0236	0.9073	0.9483
VCR	# of layers: 80	0.0000	0.9139	0.9483
VCR	# of layers: 90	0.0000	0.9139	0.9483
VCR	# of layers: 100	0.0000	0.9139	0.9483
UKM	$\hat{P}$, real, w/o bias	–	0.9194	0.9131
QCL	# of layers: 5	–	0.8798	0.8768
QCL	# of layers: 10	–	0.7814	0.7767

We show the success rates for the training and test datasets and the value of the cost function for the VCR. The input for the VCR is $\hat{P}$ created by the UKM under the condition of real matrices without the bias term with $r = 0.010$. For reference, we add the last three rows that show the results of 5-fold CV. The table shows that around 50 layers, by combining the UKM with the VCR one can get a better performance than that of QCL.

Figure 6 and Table 5 show that $\hat{U}_\mathrm {c} (\theta ; L)$ gives fairly high performance. Furthermore, we have $L_{0.001} = 80$ where the definition of $L_\delta$ is given in Eq. (27). This implies that 80 layers are sufficient to approximate the given unitary operator in the case of the CNOT-based circuit.

Note that the optimization problems arising in VQC involve well-known cost functions used in machine learning, with the added constraint of unitarity. Thus, in general, the UKM optimization problem is nonconvex and there is no rigorous proof that the UKM will achieve the best possible solution; the same is the case with the ansatz-dependent QCL. We can, however, make the following observations: (i) Clearly, the optimal performance of the UKM is an upper bound for the optimal performance of QCL; by focusing on unitary operations, the UKM searches over all possible ansätze; (ii) Given any QCL solution, the UKM can almost always show better performance (in the worst case, the same performance), by initializing it with the QCL solution; (iii) Even with random initializations, as shown numerically in the paper, the expected performance of the UKM can be better than the expected performance of QCL. Thus, the UKM can be framework enables one to estimate the first known computationally-determined bounds on the price of ansatz.

We also emphasize that the initialization of the UKM is done randomly, but the numerical simulations show that the UKM works stably.

## Discussions

As shown in this paper, the performance of QCL is bounded from above by the UKM, which in turn has its performance bounded above by kernel method based classical classifiers. One of the primary contributing factors is the difference in the degrees of freedom in QCL and the UKM. In the UKM, we have ${\mathcal {O}} (M^2)$ parameters to estimate; on the other hand, the number of parameters in QCL is ${\mathcal {O}} (L \ln M)$. This difference implies that a circuit ansatz introduces a strong bias in QCL, and may restrict the performance of QCL considerably. Thus, by designing the UKM, we can explore the ultimate power of QCL and at least, for the case of a small number of qubits *n*, the numerical results in this paper show that the ultimate power of QCL is limited (see Table 1); the performance of the UKM could be up to 10-20% higher than that of the QCL. As noted earlier, we can also explore the potential limitations of QCL from the viewpoint of optimization. Figure 3 implies the difficulty of optimizing parameters in QCL. The success rates in Fig. 3 should be more smooth and monotonically increasing: clearly, a circuit with *L* layers should perform better than a circuit with $L-1$ layers, but it seems the QCL can easily get stuck in local minima. This phenomenon may come from the barren plateau problem^15^. On the other hand, the performance of the UKM is very high and close to that of the kernel method in Fig. 4; thus, we can say that the UKM does not suffer from a similar optimization problem. This also implies that finding a proper ansatz such that the QCL paradigm attains the same performance as the UKM is a computationally challenging problem. Even if an ansatz has the representation capability to yield optimal results, the QCL optimization algorithm might not find the optimal gate parameters.

Then, we turn our attention to discussing the numerical results of the VCR. Recall that *M* and *L* are the dimension of the data points and the number of layers in an ansatz adopted in QCL, respectively. Note also that we use amplitude encoding in this paper. Then circuits in QCL have $\lceil \ln M \rceil$ qubits and have ${\mathcal {O}} (L \ln M)$ gates. The number of parameters to estimate is of the same order since we use the three-dimensional rotation gate as a parametrized gate. The UKM also has the same number of qubits $\lceil \ln M \rceil$; so it retains the qubit efficiency, but it optimizes over ${\mathcal {O}} (M^2)$ parameters. Moreover, circuits obtained by the combination of the UKM and the VCR are still of complexity ${\mathcal {O}} (L \ln M)$, except that now *L* is not a constant, as in QCL. For the datasets used in this paper, the VCR yields much more compact circuits than traditional methods for obtaining circuits for unitary operators, such as the QSD, where the number of gates will be ${\mathcal {O}} (M^2)$. Thus, the VCR yields better performance than the traditional methods.

Also, we show using the VCR that we can realize the unitary operator obtained by the UKM using the same ansatz used in QCL. Furthermore, the combination of the UKM and the VCR leads to better performance and a circuit with fewer gates or layers than QCL in some cases; see also the section on the numerical simulation of the VCR in the SM [See Sect. S-XII of the SM. We show the numerical results of the VCR on two additional datasets, and their results are consistent.]. In other cases, we have bigger circuits (i.e., *L* is larger) but with better performance. If a dataset has very high dimensions, i.e. *M* is very large, the computational time and circuit size might be very large, ${\mathcal {O}} (M^2)$. But we still have the $\lceil \ln M \rceil$ advantage in the number of qubits *n*. However, QCL also has two major potential problems, when *M* is very large. First, the dataset size has to be very large due to the curse of dimensionality, as *M* increases. So the training time and convergence complexity will be a problem no matter what the parameter size is. Second, there is no guarantee that a kernel function with ${\mathcal {O}} (L \ln M)$ parameters will do well, especially for small *L*. The performance for small *L* and large *M* could be poor. There is no theoretical proof that, for large *M*, QCL will do well with small *L*. We both use the same number of qubits $\lceil \ln M \rceil$; so in terms of intermediate-scale quantum computers, we both have the same advantage. And the computation of the VCR is ${\mathcal {O}} (M^2)$; so it is doable for any reasonable dimensions *M*. *In particular, we believe the UKM can be used to derive VQC implementations on NISQ devices comprising up to 20 qubits, (i.e.* $M=10^6$ *or million dimensional data sets) using enough classical computing resources.* Thus, in addition to the application of UKM in deriving bounds and understanding the role of ansatz in quantum algorithms, it can even complement QCL in the short term and design optimal VQCs for NISQ devices.

In this paper, we focused on amplitude encoding. Recently, the relationship between QCL and the kernel method was discussed from the viewpoint of encoding in Ref.^11^. More specifically, the basis encoding, the angle encoding, coherent state encoding, and other encodings were investigated besides amplitude encoding. We also would like to note that amplitude encoding provides a logarithmic compression: the number of qubits needed is logarithm in the dimension of the data. Most other encoding schemes require qubits proportional to the dimension (1/2 for example). Given that VQCs are a sub-class of classical kernel methods – that is, there is no performance gain over classical ML algorithms – the only potential quantum advantage lies in the logarithmic compression in qubits. Hence, from a practical perspective amplitude encoding is the most interesting case to study. However, from a research perspective, it will be interesting to investigate the performance of VQCs for such encodings via the UKM and to compare the relative performances of QCL and the UKM for such encodings as well.

We next mention some recent related literature related QML. In Ref.^24,25^, VQAs that changes the form of a circuit adaptively are studied. Such an incremental search over the ansatz space might lead to better performance than vanilla VQAs. However, their performance should be limited compared to the UKM because the UKM directly solves an optimization problem with a weaker constraint: by optimizing over unitary operations, the UKM efficiently searches over all ansatz. Thus, we think that the UKM provides one with better bounds though they are not compared in this study. In Ref.^26^, the authors insist that classical machine learning (ML) with data rivals quantum ML. This point is very important because it implies the difficulty of showing a quantum advantage of quantum ML. In our manuscript, we demonstrated that the expressive power of QCL is lower than the vanilla kernel method even if we get rid of the assumption of a specific form of a quantum circuit, i.e., VQCs performance is bounded above by a kernel method. We believe that both, our manuscript and Ref.^26^, use different approaches to reach the same conclusion that QCL may not not be as promising as researchers had originally expected it to be.

Finally, we mention the possible applicability of the UKM to other problems. In the QAOA and the VQE, optimization problems are dealt with and similarly to QCL some kinds of underlying circuit geometries are assumed. By using the UKM, it is expected that we can clarify the power of the QAOA and the VQE in an ansatz-independent manner. Furthermore, VQAs for a number of problems have been proposed: the general stochastic simulation of mixed states^27^, time evolution simulation with a non-Hermitian Hamiltonian, linear algebra problem, and open quantum system dynamics^28^, stochastic differential equations^29^, quantum fisher information^30^, the simulation of nonequilibrium steady states^31^, and molecular simulation^24^. We believe that the UKM is also applicable for this class of problems and may clarify the hidden power of VQAs.

## Concluding remarks

In this paper, we have first discussed the mathematical relationship between VQCs, which are a superset of QCL, and the kernel method. This relationship implies that VQCs including QCL is a subset of the classical kernel method and cannot outperform the kernel method.

Then we have proposed the UKM for classification problems. Mathematically the UKM lies between the kernel method and QCL, and thus it is expected to provide us an upper bound on the performance of QCL. By extensive numerical simulations, we have shown that the UKM is better than QCL, as expected. We also have proposed the VCR to find a circuit geometry that realizes a given unitary operator. By combining the UKM and the VCR, we have shown that we can find a circuit geometry that shows high performance in classification.

In future work, we plan to explore the performance of VQCs for other methods of encoding the related classical data. For example, one straightforward extension would be to embed the feature vector $x_i \in {\mathbb {R}}^M$ into a higher dimensional vector $\phi (x_i) \in {\mathbb {R}}^L$ with $L= {\mathcal {O}} (M^c)$ and then use the rest of the framework; the number of qubits *n* will still be ${\mathcal {O}} (\log M)$, thus retaining any potential quantum advantage. Such extensions can increase the power of both VQCs and QCL.

## Supplementary Information

Supplementary Information.

## Data Availability

The datasets used and/or analyzed during the current study available from the corresponding author on reasonable request.
