# Supplementary material for: Ansatz-Independent Variational Quantum Classifiers and the Price of Ansatz
Source: Sci Rep. 2022 Nov 14;12:19520. doi: 10.1038/s41598-022-20688-5 (PMC9663545; doi:10.1038/s41598-022-20688-5)
Supplement: Supplementary file 1 — Supplementary Information. [file 41598_2022_20688_MOESM1_ESM.pdf]

Supplemental Material
# – Ansatz-Independent Variational Quantum Classifiers and the Price of Ansatz –

Hideyuki Miyahara and Vwani Roychowdhury
Department of Electrical and Computer Engineering,
Henry Samueli School of Engineering and Applied Science,
University of California, Los Angeles, California 90095
(Dated: September 23, 2022)

This is the supplemental material for the paper entitled "Ansatz-Independent Variational Quantum Classifier."

CONTENTS

SI. Introduction 2

SII. Notation 2
 A. Product operators 2
 B. Matrix norms 2

SIII. Matrix derivative 2
 A. Matrix derivative 3
  1. Real case 3
  2. Complex case 3
 B. Derivative of the expectation value with respect to a unitary operator 4

SIV. Review of optimization methods 5
 A. Basics of the gradient method 5
  1. Iteration method based on Linear search 5
  2. Linear search 6
 B. Nonlinear CG method 7
  1. Linear CG method 7
  2. Nonlinear CG method 7
 C. BFGS method 8
  1. Algorithmic details of the quasi-Newton method 8
  2. Condition for B_k 9
  3. BFGS formula 9

SV. Quantum circuit learning 10
 A. Encoding 10
 B. Quantum circuits 10
 C. Measurement and prediction 13
 D. Cost function and loss functions 13
 E. Algorithmic procedure of QCL 13

SVI. Kernel method 14
 A. Algorithmic details of the kernel method 14
 B. Ridge classification 15
 C. Optimal solution in the case of Ridge classification 15

SVII. Correspondence between QCL and the kernel method 17
 A. Rewriting QCL 17
 B. Quadratic form of the kernel method 17
 C. Relationship between QCL and the kernel method 18

SVIII. Splitting method for orthogonality constrained problems 18
 A. Bregman iterative regularization 18
 B. Correspondence between Bregman iterative regularization and the augmented Lagrange method 20
 C. Penalty method and augmented Lagrangian method for optimization problems with orthogonality constraints 21
 D. Algorithmic details of the method of SOC 21

SIX. Unitary kernel method 23
 A. Algorithmic details of the UKM 24
 B. Derivatives of the cost function 25
 C. UKM with the nonlinear CG method 27

SX. Variational circuit realization 27
 A. Algorithmic details of the VCR 28
 B. Derivatives of the cost functions 28

SXI. Numerical simulation of QCL, the UKM, and the kernel method 29

A. Numerical setting 29
B. Datasets 29
C. Summary 30
D. Iris dataset (0 or 1) 31
E. Iris dataset (0 or non-0) 36
F. Iris dataset (1 or non-1) 41
G. Cancer dataset (0 or 1) 46
H. Sonar dataset (0 or 1) 51
I. Wine dataset (0 or non-0) 56
J. Semeion dataset (0 or 1) 61
K. Semeion dataset (0 or non-0) 66
L. MNIST256 dataset (0 or 1) 71
M. MNIST256 dataset (0 or non-0) 76
N. Discussions 81

S-XII. Numerical simulation of the VCR 81
A. Numerical setting 81
B. Datasets 81
C. Iris dataset (1 or non-1) 81
D. Cancer dataset (0 or 1) 81
E. Wine dataset (0 or non-0) 82
F. Discussions 83

S-XIII. Conclusion 83

References 83

S-I. INTRODUCTION

This is the supplemental material (SM) for the paper entitled "Ansatz-Independent Variational Quantum Classifier."

The SM has two major purposes. The first one is to provide the detailed descriptions of the unitary kernel method (UKM). The second one is to show additional numerical results of the UKM to support the statements in the main text. Furthermore, we explain some useful formulas and related algorithms.

S-II. NOTATION

We introduce some symbols: product operators and matrix norms.

A. Product operators

We define

$$\prod^{\uparrow}_{j=k,k+1,\ldots,k+l} \hat{A}_j \coloneqq \hat{A}_k \hat{A}_{k+1} \ldots \hat{A}_{k+l}, \tag{S-II.1.1}$$

$$\prod^{\downarrow}_{j=k,k+1,\ldots,k+l} \hat{A}_j \coloneqq \hat{A}_{k+l} \hat{A}_{k+l-1} \ldots \hat{A}_k. \tag{S-II.1.2}$$

When Eq. (S-II.1.1) and Eq. (S-II.1.2) are identical, we denote them by $\prod_{j=k}^{k+l} \hat{A}_j$.

B. Matrix norms

Let A be a $M \times N$ matrix. We define $a_{i,j} \coloneqq [A]_{i,j}$ for $i = 1, 2, \ldots, M$ and $j = 1, 2, \ldots, N$. For $p > 0$, we then define the matrix norm by

$$\|A\|_p \coloneqq \left(\sum_i \sum_j |a_{i,j}|^p \right)^{\frac{1}{p}}. \tag{S-II.2.1}$$

From Eq. (S-II.2.1), we have

$$\begin{aligned} \|A\|_{\mathrm{F}} &\coloneqq \|A\|_2 && \text{(S-II.2.2)} \\ &= (\mathrm{Tr}[A^{\mathrm{H}} A])^{\frac{1}{2}} && \text{(S-II.2.3)} \\ &= \left(\sum_{i=1}^{M} \sum_{j=1}^{N} |a_{i,j}|^2 \right)^{\frac{1}{2}} && \text{(S-II.2.4)} \\ &= \left(\sum_{i=1}^{\min(M,N)} \sigma_i^2 \right)^{\frac{1}{2}}, && \text{(S-II.2.5)} \end{aligned}$$

where $(\cdot)^{\mathrm{H}}$ denotes the Hermitian conjugate of a matrix.

S-III. MATRIX DERIVATIVE

In the UKM, we optimize the cost function of a unitary matrix. Then, we provide the formula of matrix derivatives.

A. Matrix derivative

We review the derivative of the trace with respect to a matrix. For details, refer to Refs. [1, 2].

1. Real case

Let A and X be square matrices and define $a_{i,j}$ and $x_{i,j}$ by

$$\begin{aligned} a_{i,j} &\coloneqq [A]_{i,j}, && \text{(S-III.1.1)} \\ x_{i,j} &\coloneqq [X]_{i,j}, && \text{(S-III.1.2)} \end{aligned}$$

where $[\cdot]_{i,j}$ is the element in the i-th row and the j-th column of a matrix. Then, we have

$$\begin{aligned} \frac{d}{dx_{i,j}} \mathrm{Tr}[A X^{\top}] &= a_{i,j}, && \text{(S-III.1.3)} \\ \frac{d}{dx_{i,j}} \mathrm{Tr}[A X] &= -a_{j,i}. && \text{(S-III.1.4)} \end{aligned}$$

The matrix representations of Eqs. (S-III.1.3) and (S-III.1.4) are, respectively,

$$\begin{aligned} \frac{d}{dX} \mathrm{Tr}[A X^{\top}] &= A, && \text{(S-III.1.5)} \\ \frac{d}{dX} \mathrm{Tr}[A X] &= A^{\top}. && \text{(S-III.1.6)} \end{aligned}$$

By using Eq. (S-III.1.5) and (S-III.1.6), we have

$$\frac{d}{dX} \mathrm{Tr}[A X B X^{\top} C] = A^{\top} C^{\top} X B^{\top} + C A X B. \tag{S-III.1.7}$$

2. Complex case

So far, we have considered the matrix derivatives of the trace in the case of real matrices. Here, we present the matrix derivatives in the case of complex matrices.

Let $(\cdot)^*$, $(\cdot)^\top$, and $(\cdot)^{\mathrm{H}}$ denote the complex conjugate, the transpose, and the Hermitian conjugate of a matrix. Then we have

$$A^{\mathrm{H}} = (A^*)^\top \tag{S-III.1.8}$$
$$= (A^\top)^*. \tag{S-III.1.9}$$

For a complex matrix X, we denote, by $\mathfrak{R}[X]$ and $\mathfrak{I}[X]$, the real and complex parts of X:

$$\mathfrak{R}[X] \coloneqq \frac{X + X^*}{2}, \tag{S-III.1.10}$$
$$\mathfrak{I}[X] \coloneqq \frac{X - X^*}{2i}. \tag{S-III.1.11}$$

Furthermore, we define

$$x_{i,j} \coloneqq [X]_{i,j}, \tag{S-III.1.12}$$

and

$$x^{\mathfrak{R}}_{i,j} \coloneqq \mathfrak{R}[x_{i,j}], \tag{S-III.1.13}$$
$$x^{\mathfrak{I}}_{i,j} \coloneqq \mathfrak{I}[x_{i,j}]. \tag{S-III.1.14}$$

From Eq. (S-III.1.10), we have

$$\mathrm{Tr}\big[AX^{\mathrm{H}}\big] = \mathrm{Tr}\big[A\mathfrak{R}\big[X^{\mathrm{H}}\big]\big] + i\mathrm{Tr}\big[A\mathfrak{I}\big[X^{\mathrm{H}}\big]\big] \tag{S-III.1.15}$$
$$= \mathrm{Tr}\big[A\mathfrak{R}\big[X^\top\big]\big] - i\mathrm{Tr}\big[A\mathfrak{I}\big[X^\top\big]\big]. \tag{S-III.1.16}$$

Then, the complex versions of Eqs. (S-III.1.3) and (S-III.1.4) are, respectively,

$$\frac{d}{dx^{\mathfrak{R}}_{i,j}}\mathrm{Tr}[AX^{\mathrm{H}}] = a_{i,j}, \tag{S-III.1.17}$$
$$i\frac{d}{dx^{\mathfrak{I}}_{i,j}}\mathrm{Tr}[AX^{\mathrm{H}}] = a_{i,j}, \tag{S-III.1.18}$$

and

$$\frac{d}{dx^{\mathfrak{R}}_{i,j}}\mathrm{Tr}[AX] = a_{j,i}, \tag{S-III.1.19}$$
$$i\frac{d}{dx^{\mathfrak{I}}_{i,j}}\mathrm{Tr}[AX] = a_{j,i}. \tag{S-III.1.20}$$

Furthermore, from Eqs. (S-III.1.17) and (S-III.1.18), the complex versions of Eqs. (S-III.1.5) and (S-III.1.6) are, respectively,

$$\frac{d}{d\mathfrak{R}[X]}\mathrm{Tr}[AX^{\mathrm{H}}] = A, \tag{S-III.1.21}$$
$$i\frac{d}{d\mathfrak{I}[X]}\mathrm{Tr}[AX^{\mathrm{H}}] = A, \tag{S-III.1.22}$$

and

$$\frac{d}{d\mathfrak{R}[X]}\mathrm{Tr}[AX] = A^\top, \tag{S-III.1.23}$$
$$i\frac{d}{d\mathfrak{I}[X]}\mathrm{Tr}[AX] = -A^\top. \tag{S-III.1.24}$$

B. Derivative of the expectation value with respect to a unitary operator

Let $(\cdot)^\dagger$ denote the Hermitian conjugate of an operator. For an operator $\hat{A}$, we have

$$\hat{A}^\dagger = (\hat{A}^*)^\top \tag{S-III.2.1}$$
$$= (\hat{A}^\top)^*. \tag{S-III.2.2}$$

As defined in Eqs. (S-III.1.10) and (S-III.1.11), we denote, by $\mathfrak{R}[\hat{U}]$ and $\mathfrak{I}[\hat{U}]$, the real and imaginary parts of $\hat{U}$:

$$\mathfrak{R}[\hat{U}] \coloneqq \frac{\hat{U} + \hat{U}^*}{2}, \tag{S-III.2.3}$$
$$\mathfrak{I}[\hat{U}] \coloneqq \frac{\hat{U} - \hat{U}^*}{2i}. \tag{S-III.2.4}$$

The expectation value of $\hat{O}$ with respect to the state that evolves from $\hat{\rho}$ by $\hat{U}$ is given by $\mathrm{Tr}[\hat{U}^\dagger \hat{O} \hat{U} \hat{\rho}]$. In the UKM, it is the central problem to find $\hat{U}$ that minimizes a given cost function. Then, it is helpful if we have the derivative of $\mathrm{Tr}[\hat{U}^\dagger \hat{O} \hat{U} \hat{\rho}]$ with respect to $\hat{U}$. By using Eqs. (S-III.1.21) and (S-III.1.22), we have

$$\frac{d}{d\mathfrak{R}[\hat{U}]}\mathrm{Tr}[\hat{U}^\dagger \hat{O} \hat{U} \hat{\rho}] = \frac{d}{d\mathfrak{R}[\hat{U}]}\mathrm{Tr}[\hat{U}^\dagger \hat{O} \hat{V} \hat{\rho}]|_{\hat{V}=\hat{U}} + \frac{d}{d\mathfrak{R}[\hat{U}]}\mathrm{Tr}[\hat{V}^\dagger \hat{O} \hat{U} \hat{\rho}]|_{\hat{V}^\dagger=\hat{U}^\dagger} \tag{S-III.2.5}$$
$$= \hat{O}\hat{U}\hat{\rho} + \hat{O}^\top \hat{U}^* \hat{\rho}^\top. \tag{S-III.2.6}$$

Here, we have used

$$\frac{d}{d\mathfrak{R}[\hat{U}]}\mathrm{Tr}[\hat{U}^\dagger \hat{O} \hat{V} \hat{\rho}]|_{\hat{V}=\hat{U}} = \frac{d}{d\mathfrak{R}[\hat{U}]}\mathrm{Tr}[\hat{O} \hat{V} \hat{\rho} \hat{U}^\dagger]|_{\hat{V}=\hat{U}} \tag{S-III.2.7}$$
$$= \hat{O}\hat{U}\hat{\rho}, \tag{S-III.2.8}$$
$$\frac{d}{d\mathfrak{R}[\hat{U}]}\mathrm{Tr}[\hat{V}^\dagger \hat{O} \hat{U} \hat{\rho}]|_{\hat{V}^\dagger=\hat{U}^\dagger} = \frac{d}{d\mathfrak{R}[\hat{U}]}\mathrm{Tr}[\hat{\rho} \hat{V}^\dagger \hat{O} \hat{U}]|_{\hat{V}^\dagger=\hat{U}^\dagger} \tag{S-III.2.9}$$
$$= (\hat{\rho}\hat{U}^\dagger \hat{O})^\top \tag{S-III.2.10}$$
$$= \hat{O}^\top \hat{U}^* \hat{\rho}^\top. \tag{S-III.2.11}$$

Similarly, we also have

$$\frac{d}{d\mathfrak{I}[\hat{U}]}\mathrm{Tr}[\hat{U}^\dagger \hat{O} \hat{U} \hat{\rho}] = \frac{d}{d\mathfrak{I}[\hat{U}]}\mathrm{Tr}[\hat{U}^\dagger \hat{O} \hat{V} \hat{\rho}]|_{\hat{V}=\hat{U}} + \frac{d}{d\mathfrak{I}[\hat{U}]}\mathrm{Tr}[\hat{V}^\dagger \hat{O} \hat{U} \hat{\rho}]|_{\hat{V}^\dagger=\hat{U}^\dagger} \tag{S-III.2.12}$$
$$= -i\hat{O}\hat{U}\hat{\rho} + i\hat{O}^\top \hat{U}^* \hat{\rho}^\top. \tag{S-III.2.13}$$

Here, we have used

$$i\frac{d}{d\mathfrak{I}[\hat{U}]}\mathrm{Tr}[\hat{U}^\dagger \hat{O} \hat{V} \hat{\rho}]|_{\hat{V}=\hat{U}} = i\frac{d}{d\mathfrak{I}[\hat{U}]}\mathrm{Tr}[\hat{O} \hat{V} \hat{\rho} \hat{U}^\dagger]|_{\hat{V}=\hat{U}} \tag{S-III.2.14}$$
$$= \hat{O}\hat{U}\hat{\rho}, \tag{S-III.2.15}$$
$$i\frac{d}{d\mathfrak{I}[\hat{U}]}[\hat{V}^\dagger \hat{O} \hat{U} \hat{\rho}]|_{\hat{V}^\dagger=\hat{U}^\dagger} = i\frac{d}{d\mathfrak{I}[\hat{U}]}[\hat{\rho} \hat{V}^\dagger \hat{O} \hat{U}]|_{\hat{V}^\dagger=\hat{U}^\dagger} \tag{S-III.2.16}$$
$$= -(\hat{\rho}\hat{U}^\dagger \hat{O})^\top \tag{S-III.2.17}$$
$$= -\hat{O}^\top \hat{U}^* \hat{\rho}^\top. \tag{S-III.2.18}$$

We utilize Eqs. (S-III.2.6) and (S-III.2.13) in the UKM.

S-IV. REVIEW OF OPTIMIZATION METHODS

In the UKM, optimization algorithms play an important role. There are many approaches to solve minimization problems, such as the gradient method, the Newton method, the quasi-Newton method [3, 4]. Among them, the

ALGORITHM S-1: Iterative method based on linear search

1: initialize x_0
2: **for** $k = 1, 2, \dots, K$ **do**
3: compute d_k that satisfies Eq. (S-IV.1.3)
4: compute α_k
5: compute x_k by Eq. (S-IV.1.2)
6: **end for**

nonlinear conjugate gradient (CG) method [5, 6] is very simple and work well for optimization problems associated with a high dimensional vector [5, 6] and the Broyden-Fletcher-Goldfarb-Shanno (BFGS) method [5, 7–10] is one of the most sophisticated methods. In this SM, the CG method and the UKM and the BFGS method are used for the UKM, respectively.

In this section, we review the CG method and the BFGS method as optimization methods. For details, refer to Refs. [3, 4].

A. Basics of the gradient method

Before getting into the CG method the BFGS method, we review the basics of the gradient method.

1. Iteration method based on Linear search

Let us consider the following optimization problem:

$$\min_x \mathcal{J}_{\mathrm{cost}}(x). \tag{S-IV.1.1}$$

Let d_k and α_k be the gradient descent direction and the step size at the k-th iteration, respectively. Then we update x_k by

$$x_k = x_{k-1} + \alpha_k d_k. \tag{S-IV.1.2}$$

To decrease $\mathcal{J}_{\mathrm{cost}}(x)$ in Eq. (S-IV.1.1), the gradient direction d_k must satisfy the following condition:

$$\nabla \mathcal{J}_{\mathrm{cost}}(x_k)^\top d_k < 0. \tag{S-IV.1.3}$$

By using the Taylor expansion, we have

$$\mathcal{J}_{\mathrm{cost}}(x_k) - \mathcal{J}_{\mathrm{cost}}(x_{k-1}) = \alpha_k \left(\nabla \mathcal{J}_{\mathrm{cost}}(x_{k-1})^\top d_k + \frac{o(\alpha_k)}{\alpha_k} \right). \tag{S-IV.1.4}$$

Then, α_k is sufficiently small, Eq. (S-IV.1.2) is expected to work well. We summarize the iterative method based on linear search in Algo. S-1.

2. Linear search

It is quite important to compute appropriate α_k, and there exist well-known conditions for good α_k. Here we review the conditions for α_k.

To derive the conditions for α_k, let us consider the minimization problem of the following function:

$$\phi(\alpha) \coloneqq \mathcal{J}_{\mathrm{cost}}(x + \alpha d). \tag{S-IV.1.5}$$

The simplest condition for α_k is the Armijo condition [11] given by

$$\phi(\alpha) \le \phi(0) + c_1 \alpha \phi'(0), \tag{S-IV.1.6}$$

for $0 < c_1 < 1$. When we use Eq. (S-IV.1.6), α_k is likely to be very small. Another well-used condition for α_k is the Wolfe condition [12, 13] given by

$$\phi(\alpha) \leq \phi(0) + c_1 \alpha \phi'(0), \tag{S-IV.1.7a}$$
$$\phi'(\alpha) \geq c_2 \phi'(0), \tag{S-IV.1.7b}$$

for $0 < c_1 < c_2 < 1$. In this case, $\phi'(\alpha)$ may take a large positive number. To avoid this problem, the following condition for α, which is called the strong Wolfe condition, is used:

$$\phi(\alpha) \leq \phi(0) + c_1 \alpha \phi'(0), \tag{S-IV.1.8a}$$
$$|\phi'(\alpha)| \geq c_2 |\phi'(0)|, \tag{S-IV.1.8b}$$

for $0 < c_1 < c_2 < 1$. Finally, we also mention the Goldstein condition for α given by

$$\phi(0) + (1 - c)\alpha\phi'(0) \leq \phi(\alpha) \tag{S-IV.1.9}$$
$$\leq \phi(0) + c\alpha\phi'(0), \tag{S-IV.1.10}$$

for $0 < c < 1/2$.

To find α_k that satisfies one of the above conditions, the backtracking line search is often used. In the backtracking line search, we set $\alpha_0 > 0$ and iterate the following equation until Eq. (S-IV.1.6) is satisfied:

$$\alpha_k = \rho \alpha_{k-1}, \tag{S-IV.1.11}$$

where $\rho \in (0, 1)$.

B. Nonlinear CG method

In the UKM, the gradient method is utilized to $\hat{U}_k$ in Eq. (S-IX.1.8). The BFGS method is known as a sophisticated method but requires relatively large memory space. Then, we use the CG method in the UKM. Here, we explain the CG method.

1. *Linear CG method*

We begin with the CG method. Let us consider minimizing

$$\mathcal{J}_{\text{cost}}(x) \coloneqq \frac{1}{2}(x - x_*)^\top A (x - x_*), \tag{S-IV.2.1}$$

where $A \succ O$.

In the CG method, we set x_0 and $d_0 = -\nabla \mathcal{J}_{\text{cost}}(x_0)$, and then iterate, until convergence,

$$x_k = x_{k-1} + \alpha_k d_{k-1}, \tag{S-IV.2.2a}$$
$$d_k = -\nabla \mathcal{J}_{\text{cost}}(x_k) + \beta_k d_{k-1}, \tag{S-IV.2.2b}$$

where

$$\alpha_k = -\frac{\nabla \mathcal{J}_{\text{cost}}(x_{k-1})^\top d_{k-1}}{d_{k-1}^\top A d_{k-1}}, \tag{S-IV.2.3a}$$
$$\beta_k = \frac{\nabla \mathcal{J}_{\text{cost}}(x_k)^\top A d_{k-1}}{d_{k-1}^\top A d_{k-1}}. \tag{S-IV.2.3b}$$

The CG method is summarized in Algo. S-2.

ALGORITHM S-2: Conjugate gradient (CG) method

```
initialize x_0
set d_0 = −∇J_cost(x_0)
for k = 1, 2, ..., K do
    compute x_k by Eq. (S-IV.2.2a) with Eq. (S-IV.2.3a)
    compute d_k by Eq. (S-IV.2.2b) with Eq. (S-IV.2.3b)
end for
```

ALGORITHM S-3: Nonlinear conjugate gradient (CG) method

```
initialize x_0
set d_0 = −∇J_cost(x_0)
for k = 1, 2, ..., K do
    compute α_k by Eq. (S-IV.2.4)
    compute x_k by Eq. (S-IV.2.2a)
    compute β_k by Eq. (S-IV.2.5)
    compute d_k by Eq. (S-IV.2.2b)
end for
```

2. Nonlinear CG method

Eq. (S-IV.2.3) requires the Hessian matrix A. When we do not know the Hessian, we need to replace Eq. (S-IV.2.3) with different approaches. These approaches are called the nonlinear CG method [5, 6, 14–16].

First, let us focus on how to compute α_k because Eq. (S-IV.2.3a) is not available when we do not know the Hessian. It is very simple because we can use the line search method:

$$\alpha_k = \arg\min_{\alpha} \mathcal{J}_{\text{cost}}(x_{k-1} + \alpha d_{k-1}). \tag{S-IV.2.4}$$

Here, Eqs. (S-IV.1.6) and (S-IV.1.7) are often used.

Next, let us turn our attention to β_k. While we do not elaborate on them, there are several methods to replace β_k in Eq. (S-IV.2.3b):

$$\beta_k = \frac{\|\nabla \mathcal{J}_{\text{cost}}(x_k)\|_{\text{F}}^2}{\|\nabla \mathcal{J}_{\text{cost}}(x_{k-1})\|_{\text{F}}^2}, \tag{S-IV.2.5}$$

$$\beta_k = \frac{\nabla \mathcal{J}_{\text{cost}}(x_k)^{\top} y_k}{\|\nabla \mathcal{J}_{\text{cost}}(x_{k-1})\|_{\text{F}}^2}, \tag{S-IV.2.6}$$

$$\beta_k = \frac{\nabla \mathcal{J}_{\text{cost}}(x_k)^{\top} y_k}{d_k^{\top} y_k}, \tag{S-IV.2.7}$$

$$\beta_k = \frac{\|\nabla \mathcal{J}_{\text{cost}}(x_k)\|_{\text{F}}^2}{d_k^{\top} y_k}, \tag{S-IV.2.8}$$

where $y_k \coloneqq \nabla \mathcal{J}_{\text{cost}}(x_k) - \nabla \mathcal{J}_{\text{cost}}(x_{k-1})$. In this SM, we utilize Eq. (S-IV.2.5) called the Fletcher-Reeves method [5]. Eq. (S-IV.2.6), Eq. (S-IV.2.7), and Eq. (S-IV.2.8) are called the Polak-Ribière method [14], the Hestenes-Stiefel method [15], and the Dai-Yuan method [16], respectively.

The nonlinear CG method is summarized in Algo. S-3.

C. BFGS method

The aim of this subsection is to describe the BFGS method [5, 7–10].

ALGORITHM S-4: Newton's method

1: initialize x_0
2: **for** $k = 1, 2, \ldots, K$ **do**
3: compute x_k by Eq. (S-IV.3.1)
4: **end for**

1. *Algorithmic details of the quasi-Newton method*

Let us consider the optimization problem, Eq. (S-IV.1.1). We begin with Newton's method. In Newton's method, we set x_0 and iterate the following equation until convergence:

$$x_k = x_{k-1} - (\nabla^2 \mathcal{J}_{\text{cost}}(x_{k-1}))^{-1} \nabla \mathcal{J}_{\text{cost}}(x_{k-1}). \tag{S-IV.3.1}$$

Newton's method is summarized in Algo. S-4 The biggest problem of Newton's method is the difficulty of computing $\nabla^2 \mathcal{J}_{\text{cost}}(x_{k-1}))^{-1}$ in Eq. (S-IV.3.1). Then some alternative methods, called the quasi-Newton method, were proposed [4].

Let us turn our attention to the quasi-Newton method. In the quasi-Newton method, the gradient descent direction is computed as

$$d_k = -B_k^{-1} \nabla \mathcal{J}_{\text{cost}}(x_k), \tag{S-IV.3.2}$$

where B_k is an approximation of $\nabla^2 \mathcal{J}_{\text{cost}}(x)$. Similarly, Eq. (S-IV.3.2) is equivalent to

$$d_k = -H_k \nabla \mathcal{J}_{\text{cost}}(x_k), \tag{S-IV.3.3}$$

where H_k is an approximation of $[\nabla^2 \mathcal{J}_{\text{cost}}(x)]^{-1}$. Next, by using Eqs. (S-IV.1.6) and (S-IV.1.7), we compute α_k by

$$\alpha_k = \arg\min_{\alpha} \mathcal{J}_{\text{cost}}(x_{k-1} + \alpha d_k). \tag{S-IV.3.4}$$

Then, we update x_k, in the quasi-Newton method, by

$$x_k = x_{k-1} + \alpha_k d_k. \tag{S-IV.3.5}$$

2. *Condition for B_k*

Next, we consider the condition for B_k. By the Taylor expansion, we have

$$\nabla^2 \mathcal{J}_{\text{cost}}(x_k) \times (x_k - x_{k-1}) = \nabla \mathcal{J}_{\text{cost}}(x_k) - \nabla \mathcal{J}_{\text{cost}}(x_{k-1}) + \mathcal{O}(\|x_k - x_{k-1}\|^2). \tag{S-IV.3.6}$$

By introducing

$$y_k \coloneqq \nabla \mathcal{J}_{\text{cost}}(x_k) - \nabla \mathcal{J}_{\text{cost}}(x_{k-1}), \tag{S-IV.3.7}$$

$$s_k \coloneqq x_k - x_{k-1}, \tag{S-IV.3.8}$$

Eq. (S-IV.3.6) is also expressed as

$$\nabla^2 \mathcal{J}_{\text{cost}}(x_k) s_k = y_k + \mathcal{O}(\|s_k\|^2). \tag{S-IV.3.9}$$

Thus, in the quasi-Newton method, the following secant condition is imposed:

$$B_k s_k = y_k. \tag{S-IV.3.10}$$

Eq. (S-IV.3.10) is equivalent to

$$H_k y_k = s_k. \tag{S-IV.3.11}$$

The necessary condition for B_k that satisfies the secant condition, Eq. (S-IV.3.10), to exist, is

$$s_k^\top y_k > 0, \tag{S-IV.3.12}$$

The proof of Eq. (S-IV.3.12) is as follows.

ALGORITHM S-5: BFGS method

```
initialize x_0
set B_0 = I
for k = 1, 2, ..., K do
    compute d_k by Eq. (S-IV.3.2)
    compute α_k by Eq. (S-IV.3.4)
    compute x_k by Eq. (S-IV.3.5)
    compute B_k by Eq. (S-IV.3.16)
end for
```

Proof. From Eq. (S-IV.1.7b) and $\alpha_k > 0$, we have

$$\nabla \mathcal{J}_{\mathrm{cost}}(x_k)^\top s_k \geq c_2 \nabla \mathcal{J}_{\mathrm{cost}}(x_{k-1})^\top s_k, \tag{S-IV.3.13}$$

where c_2 is a constant that appears in the Wolfe condition, Eq. (S-IV.1.7). Then, we have

$$\begin{aligned} y_k^\top s_k &= \nabla \mathcal{J}_{\mathrm{cost}}(x_k)^\top s_k - \nabla \mathcal{J}_{\mathrm{cost}}(x_{k-1})^\top s_k && \text{(S-IV.3.14)} \\ &\geq -(1 - c_2) \nabla \mathcal{J}_{\mathrm{cost}}(x_{k-1})^\top s_k. && \text{(S-IV.3.15)} \end{aligned}$$

Since $c_2 < 1$ and s_k is a gradient descent direction, we obtain Eq. (S-IV.3.12) as a necessary condition for the secant condition, Eq. (S-IV.3.10).

□

3. BFGS formula

We do not elaborate on the BFGS method, we here give the BFGS formula. The BFGS formula is given by

$$B_k = B_{k-1} - \frac{B_{k-1} s_k s_k^\top B_{k-1}}{s_k^\top B_{k-1} s_k} + \frac{y_k y_k^\top}{s_k^\top y_k}. \tag{S-IV.3.16}$$

In the case of BFGS formula, we have

$$H_k = \left(I - \frac{s_k y_k^\top}{s_k^\top y_k} \right) H_{k-1} \left(I - \frac{y_k s_k^\top}{s_k^\top y_k} \right) + \frac{s_k s_k^\top}{s_k^\top y_k}. \tag{S-IV.3.17}$$

The quasi-Newton method is summarized in Algo. S-5

So far, we explained Eq. (S-IV.3.16), but another formula for B_k is also known. The Davidon-Fletcher-Powell (DFP) formula is given in Ref. [4]

$$B_k = \left(I - \frac{y_k s_k^\top}{s_k^\top y_k} \right) B_{k-1} \left(I - \frac{s_k y_k^\top}{s_k^\top y_k} \right) + \frac{y_k y_k^\top}{s_k^\top y_k}. \tag{S-IV.3.18}$$

S-V. QUANTUM CIRCUIT LEARNING

QCL was proposed in Refs. [17, 18] as an application of the quantum approximate optimization algorithm (QAOA) [19] and the variational quantum eigensolver (VQE) [20] for machine learning. In this section, we review Refs. [17, 18]. We first explain the ingredients of QCL and then describe the whole procedure of QCL.

A. Encoding

Suppose that we have $\mathcal{D} \coloneqq \{x_i, y_i\}_{i=1}^N$ and a n-qubit quantum system, and let y_i be the label of x_i. It is an important thing to encode $x_i \in \mathbb{R}^M$ on qubits efficiently. Let the initial state be $|\mathrm{init}\rangle$. A typical example of $|\mathrm{init}\rangle$ is given by

$$|\mathrm{init}\rangle \coloneqq |\underbrace{00\ldots0}_{n}\rangle. \tag{S-V.1.1}$$

We then encode x_i by applying $\hat{S}(x_i)$ on $|\text{init}\rangle$:

$$|\psi^{\text{in}}(x_i)\rangle \coloneqq \hat{S}(x_i)|\text{init}\rangle. \tag{S-V.1.2}$$

There exist some novel quantum encoding methods; in Refs. [17, 21], amplitude encoding is used. In amplitude encoding, we construct the quantum state for x_i:

$$|\psi^{\text{in}}(x_i)\rangle = \sum_{j=1}^{2^n} \tilde{x}_{i,j}|j\rangle, \tag{S-V.1.3}$$

where

$$\tilde{x}_i \coloneqq \frac{1}{\chi_i}[x_{i,1}, x_{i,2}, \ldots, x_{i,M}, c_{i,1}, c_{i,2}, \ldots, c_{i,M'}], \tag{S-V.1.4a}$$

$$\chi_i \coloneqq \sqrt{\sum_{j=1}^{M}|x_{i,j}|^2 + \sum_{j=1}^{M'}|c_{i,j}|^2}. \tag{S-V.1.4b}$$

Here, $\tilde{x}_{i,j}$ is the j-th element of $\tilde{x}_i$ and $\{c_{i,j}\}_{j=1}^{M'}$ are certain constant values for padding and $M + M' = 2^n$.

In Ref. [22], the relationship between QCL and the kernel method is discussed from the viewpoint of encoding. More specifically, the basis encoding, the angle encoding, coherent state encoding, and other encodings are discussed in addition to amplitude encoding; these encodings may be efficient for some purposes.

B. Quantum circuits

A near-term quantum device is believed to realize a quantum circuit [23]. Suppose that we have a n-qubit system and a quantum circuit that is parameterized by θ. By applying $\hat{U}_{\text{c}}(\theta)$ realized by the quantum circuit, $|\psi^{\text{in}}(x_i)\rangle$ evolves into

$$|\psi^{\text{out}}(x_i;\theta)\rangle \coloneqq \hat{U}_{\text{c}}(\theta)|\psi^{\text{in}}(x_i)\rangle, \tag{S-V.2.1}$$

where

$$\hat{U}_{\text{c}}(\theta) \coloneqq \prod_{i=1,2,\ldots,L}^{\downarrow} \hat{U}_{\text{c},i}(\theta_i), \tag{S-V.2.2}$$

$\theta \coloneqq \{\theta_i\}_{i=1}^{L}$, and the definition of $\prod^{\downarrow}$ is given by Eq. (S-II.1.2).

The functional form of $\hat{U}_{\text{c},i}(\theta_i)$ in Eq. (S-V.2.2), depends on quantum circuits. In Refs. [17, 18, 24], the following form is often assumed for $\hat{U}_{\text{c},i}(\theta_i)$ in Eq. (S-V.2.2):

$$\hat{U}_{\text{c},i}(\theta_i) \coloneqq \left[\prod_{j=1}^{n} \hat{R}_j^{\text{3d}}(\theta_{i,j})\right]\hat{U}_{\text{ent}}. \tag{S-V.2.3}$$

Graphically, $\hat{U}_{\text{c},i}(\theta_i)$, Eq. (S-V.2.3), for $n = 4$ is expressed as

. (S-V.2.7)

$$\hat{U}_{\text{c},i}(\theta_i) = [rowsep = 0.2cm, columnsep = 0.2cm] \qquad [wires = 4]\hat{U}_{\text{ent}}\hat{R}^{\text{3d}}$$

.

Here, $\theta_i \coloneqq \{\theta_{i,j}\}_{j=1}^{n}$, $\theta_{i,j} \coloneqq \{\theta_{i,j,k}\}_{k=1}^{3}$ and $\hat{R}_j^{\text{3d}}(\theta_{i,j}) \coloneqq \hat{R}_j^{z}(\theta_{i,j,3})\hat{R}_j^{y}(\theta_{i,j,2})\hat{R}_j^{z}(\theta_{i,j,1})$ is the 3-dimensional rotation gate on the j-th qubit [25, 26]. Note that the 3-dimensional rotation gate can be, in general, decomposed into the product of the z-rotation gate and the y-rotation gate as shown in Refs. [25, 26].

Two-qubit gates are known to be universal [27]. In Refs. [17, 24], the CNOT gates and the controlled-rotation gates are used for $\hat{U}_{\rm ent}$ in Eq. (S-V.2.3). For example, we can use the following for $\hat{U}_{\rm ent}$ in Eq. (S-V.2.3):

$$\hat{U}_{\rm ent} = \prod_{j=1,2,\ldots,n}^{\downarrow} {\rm Ct}_j[\hat{X}_{j+1}], \tag{S-V.2.8}$$

where $\hat{X}_{n+1} \coloneqq \hat{X}_1$. Graphically $\hat{U}_{{\rm c},i}(\theta_i)$, Eq. (S-V.2.8), for $n = 4$ is expressed as

. (S-V.2.12)

$\hat{U}_{\rm ent} = [rowsep = 0.2cm, columnsep = 0.2cm]$ 1

.

Then, $\hat{U}_{{\rm c},i}(\theta_i)$ in Eq. (S-V.2.3) with Eq. (S-V.2.8) for $n = 4$ graphically becomes

. (S-V.2.16)

$\hat{U}_{{\rm c},i}(\theta_i) = [rowsep = 0.2cm, columnsep = 0.2cm]$ 1

.

We call this circuit geometry the CNOT-based circuit.

If only the CNOT gates are used to construct $\hat{U}_{\rm ent}$ like the above example, $\hat{U}_{\rm ent}$ is fixed and free from parameters. Instead of Eq. (S-V.2.3), we can also use

$$\hat{U}_{{\rm c},i}(\theta_i) \coloneqq \left[\prod_{j=1}^{n} \hat{R}_j^{\rm 3d}(\theta_{i,j}^1)\right] \hat{U}_{\rm ent}(\theta_{i,j}^2), \tag{S-V.2.17}$$

where

$$\hat{U}_{\rm ent}(\theta_i^2) \coloneqq \prod_{j=1}^{n}{}^{\downarrow} {\rm Ct}_j[\hat{R}_{j+1}^{\rm 3d}(\theta_{i,j}^2)], \tag{S-V.2.18}$$

and $\hat{R}_{n+1}^{\rm 3d}(\theta_{i,n+1}^2) \coloneqq \hat{R}_1^{\rm 3d}(\theta_{i,1}^2)$. In the case of Eq. (S-V.2.17), the definition of θ_i is repaced by $\theta_i \coloneqq \{\theta_i^1, \theta_i^2\}$, and we set $\theta_i^1 \coloneqq \{\theta_{i,j}^1\}_{j=1}^n$, $\theta_i^2 \coloneqq \{\theta_{i,j}^2\}_{j=1}^n$, $\theta_{i,j}^1 \coloneqq \{\theta_{i,j,k}^1\}_{k=1}^3$, and $\theta_{i,j}^2 \coloneqq \{\theta_{i,j,k}^2\}_{k=1}^3$. Graphically, $\hat{U}_{{\rm c},i}(\theta_i)$ in Eq. (S-V.2.17) for $n = 4$ becomes

. (S-V.2.22)

$\hat{U}_{{\rm c},i}(\theta_i) = [rowsep = 0.2cm, columnsep = 0.2cm]$ 1 $\hat{R}^{\rm 3d}$

.

We call this circuit geometry the CRot-based circuit.

In Ref. [18], the following operator is used for $\hat{U}_{\rm ent}$ in Eq. (S-V.2.3):

$$\hat{U}_{\rm ent} = e^{-i\Delta t \hat{H}_{\rm H}}, \tag{S-V.2.23}$$

where $\hat{H}_{\rm H}$ is the Heisenberg model given by

$$\hat{H}_{\rm H} \coloneqq \sum_{i,j} J_{i,j} \hat{\sigma}_i \cdot \hat{\sigma}_j + \sum_i \sum_{w=x,y,z} h_i^w \hat{\sigma}_i^w, \tag{S-V.2.24}$$

$$\hat{\sigma}_i \coloneqq [\hat{\sigma}_i^x, \hat{\sigma}_i^y, \hat{\sigma}_i^z]^\top. \tag{S-V.2.25}$$

More generally, $\hat{H}_{\mathrm{H}}$ can involve n-body terms with $n \geq 3$. Graphically, $\hat{U}_{\mathrm{c},i}(\theta_i)$, Eq. (S-V.2.3), with Eq. (S-V.2.23) for $n = 4$ is expressed as

. (S-V.2.29)

$\hat{U}_{\mathrm{c},i}(\theta_i) = [rowsep = 0.2cm, columnsep = 0.2cm]$ $[wires = 4]e^{-i\Delta t\hat{H}_{\mathrm{H}}}\hat{R}^{3\mathrm{d}}$

.

We call this circuit geometry the Heisenberg circuit. More specifically, we call it the 1-dimensional (1d) Heisenberg circuit, when the coefficients in Eq. S-V.2.24 are set as

$$J_{i,j} = \delta_{j,i+1}, \tag{S-V.2.30}$$
$$h_i^w = 0, \tag{S-V.2.31}$$

for $i = 1, 2, \ldots, n$ and $w = x, y, z$. And we call it the fully-connected (FC) Heisenberg circuit, when the coefficients in Eq. S-V.2.24 are set as

$$J_{i,j} = \frac{1}{N}, \tag{S-V.2.32}$$
$$h_i^w = 0, \tag{S-V.2.33}$$

for $i = 1, 2, \ldots, n$ and $w = x, y, z$.

In numerical simulation, we compare the CNOT-based circuit, the CRot-based circuit, the 1d Heisenberg circuit, and the FC Heisenberg circuit. See also Sec. S-XI A.

C. Measurement and prediction

Next, we turn our attention to measurements on $|\psi^{\mathrm{out}}(x_i;\theta)\rangle$ and prediction based on measurements. We obtain the measurement of $\hat{O}_j$ with respect to $|\psi^{\mathrm{out}}(x_i;\theta)\rangle$:

$$\langle \hat{O}_j \rangle_{x_i,\theta} \coloneqq \langle \psi^{\mathrm{out}}(x_i;\theta)|\hat{O}_j|\psi^{\mathrm{out}}(x_i;\theta)\rangle. \tag{S-V.3.1}$$

In QCL, the label of x_i is predicted by

$$f_{\mathrm{pred}}(x_i;\theta,\theta_{\mathrm{b}}) \coloneqq \sum_{j=1}^{Q} \xi_j \langle \hat{O}_j \rangle_{x_i,\theta} + \theta_{\mathrm{b}}, \tag{S-V.3.2}$$

where $\xi \coloneqq [\xi_1, \xi_2, \ldots, \xi_Q]$ are fixed parameters. Here θ_{b} is a bias term to be estimated, but we may set $\theta_{\mathrm{b}} = 0$ for simplicity.

So far, we have focused on quantum circuit leaning [17, 18], in which θ is optimized. We here mention the difference between QCL and related algorithms. In the cases of the QAOA and the VQE, $\{\xi_j\}_{j=1}^{Q}$ are fixed as in case case of QCL; buts $\{\xi_j\}_{j=1}^{Q}$ in the QAOA and the VQE encode the problem of interest, though $\{\xi_j\}_{j=1}^{Q}$ in QCL are just fixed parameters.

For the almost same purpose with QCL, quantum reservoir computing (QRC) was proposed in Refs. [28, 29]. In QRC, θ is fixed and ξ is optimized. Then, in the above problem setting, $\{\xi_j\}_{j=1}^{Q}$ are optimized.

D. Cost function and loss functions

To find a good θ, we formulate the optimization problem with respect to θ. In QCL, we consider the optimization problem of minimizing the following cost function:

$$\mathcal{J}_{\mathrm{cost}}(\theta,\theta_{\mathrm{b}}) \coloneqq \frac{1}{N}\sum_{i=1}^{N} \ell(y_i, f_{\mathrm{pred}}(x_i;\theta,\theta_{\mathrm{b}})), \tag{S-V.4.1}$$

ALGORITHM S-6: Quantum circuit learning (QCL)

while termination condition is not satisfied **do**
  compute $|\psi^{\text{in}}(x_i)\rangle$ in Eq. (S-V.1.2) for $i = 1, 2, \ldots, N$
  compute $|\psi^{\text{out}}(x_i; \theta)\rangle$ in Eq. (S-V.2.1) for $i = 1, 2, \ldots, N$
  compute $\langle \hat{O}_j \rangle_{x_i, \theta}$ in Eq. (S-V.3.1) for $j = 1, 2, \ldots, Q$ and $i = 1, 2, \ldots, N$
  compute $f_{\text{pred}}(x_i; \theta, \theta_{\text{b}}) = \sum_j \xi_j \langle \hat{O}_j \rangle_{x_i, \theta} + \theta_{\text{b}}$ in Eq. (S-V.3.2) for $i = 1, 2, \ldots, N$
  update θ and θ_{b} by minimizing $\mathcal{J}_{\text{cost}}(\theta, \theta_{\text{b}})$ in Eq. (S-V.4.1)
end while

ALGORITHM S-7: Quantum circuit learning (QCL) with stochastic gradient descent (SGD)

set $N' < N$
while termination condition is not satisfied **do**
  sample i_l from $[1, 2, \ldots, N]$ for $l = 1, 2, \ldots, N'$
  compute $|\psi^{\text{in}}(x_{i_l})\rangle$ in Eq. (S-V.1.2) for $l = 1, 2, \ldots, N'$
  compute $|\psi^{\text{out}}(x_{i_l}; \theta)\rangle$ in Eq. (S-V.2.1) for $l = 1, 2, \ldots, N'$
  compute $\langle \hat{O}_j \rangle_{x_{i_l}, \theta}$ in Eq. (S-V.3.1) for $j = 1, 2, \ldots, Q$ and $l = 1, 2, \ldots, N'$
  compute $f_{\text{pred}}(x_{i_l}; \theta, \theta_{\text{b}}) = \sum_j \xi_j \langle \hat{O}_j \rangle_{x_{i_l}, \theta} + \theta_{\text{b}}$ in Eq. (S-V.3.2) for $l = 1, 2, \ldots, N'$
  update θ and θ_{b} by minimizing $\sum_l \ell(y_{i_l}, f_{\text{pred}}(x_{i_l}; \theta, \theta_{\text{b}}))$
end while

where $\ell(\cdot,\cdot)$ is a loss function [30, 31]. For example, $\ell(\cdot,\cdot)$ takes the form

$$\ell_{\text{SE}}(y, \tilde{y}) \coloneqq \frac{1}{2}|y - \tilde{y}|^2, \tag{S-V.4.2}$$

which is called the squared error function. Letting y and $\tilde{y}$ be a label that takes ± 1 and a prediction on y, respectively, another example is

$$\ell_{\text{hinge}}(y, \tilde{y}) \coloneqq \max(0, 1 - y\tilde{y}), \tag{S-V.4.3}$$

which is called the hinge function. Letting y and $\tilde{y}$ be a label that takes 0 or 1 and a prediction on y, respectively, we can use

$$\ell_{\text{XE}}(y, \tilde{y}) \coloneqq -y \ln \tilde{y} - (1 - y) \ln(1 - \tilde{y}), \tag{S-V.4.4}$$

which is called the cross-entropy loss function. In Eq. (S-V.4.4), y and $\tilde{y}$ are not symmetric though, in Eqs. (S-V.4.4) and (S-V.4.3), y and $\tilde{y}$ are symmetric. Note that Eqs. (S-V.4.2), (S-V.4.3), and (S-V.4.4) are not specific to QCL but also used for many machine learning algorithms [30, 31] and the UKM, which is one of the main algorithms of this SM. In Ref. [17], a different cost function is introduced. However, we do not use it in this SM.

E. Algorithmic procedure of QCL

So far, we have explained the ingredients of QCL. Here we explain the whole procedure of QCL. In QCL, we estimate θ in Eq. (S-V.2.2) by iterating the following two steps. The first step is compute $\langle \hat{O}_j \rangle_{x_i, \theta}$ in Eq. (S-V.3.1). The first step is done in a quantum device. The second step is update θ and θ_{b} by minimizing $\mathcal{J}_{\text{cost}}(\theta, \theta_{\text{b}})$ in Eq. (S-V.4.1). To update θ, the derivative of $\mathcal{J}_{\text{cost}}(\theta, \theta_{\text{b}})$ is required. The second step is expected done in a classical device. We repeat these steps until convergence.

Finally, we summarize QCL in Algo. S-6. In practice, stochastic gradient descent (SGD) is also used for QCL. Then, the procedure of QCL with SGD is summarized in Algo. S-7.

S-VI. KERNEL METHOD

Machine learning provides many kinds of tool kits to analyze a wide range of datasets [30, 31]. Among them, the kernel method is one of the most important approaches because it is very powerful and mathematically simple compared with a mixture model, neural networks, etc [30, 31]. This section is devoted to the kernel method [30, 31].

A. Algorithmic details of the kernel method

Let us assume that we have

$$\mathcal{D} := [(x_1, y_1), (x_2, y_2), \dots, (x_N, y_N)], \tag{S-VI.1.1}$$

where x_i is a data point and y_i is the label of x_i for $i = 1, 2, \dots, N$. We also assume that each data point x_i is a M-dimensional vector:

$$x_i := [x_i^1, x_i^2, \dots, x_i^M]^\top, \tag{S-VI.1.2}$$

where $(\cdot)^\top$ represents the transpose.

We first define the feature map $\phi(\cdot)$ by

$$\phi(x_i) := [\phi_1(x_i), \phi_2(x_i), \dots, \phi_G(x_i)]^\top. \tag{S-VI.1.3}$$

Then, in the kernel method, by using the feature map (S-VI.1.3), the prediction on x_i is done by

$$f_{\mathrm{pred}}(x_i; v) = \sum_{j=1}^{G} v_j \phi_j(x_i), \tag{S-VI.1.4}$$

where $v := [v_1, v_2, \dots, v_G]^\top$ is the parameter to be estimated. The performance of $f_{\mathrm{pred}}(\cdot; v)$ heavily depends on v; then it is quite important to estimate v.

To find a good v, the following optimization problem is often considered:

$$\min_v \mathcal{J}_{\mathrm{cost}}(v), \tag{S-VI.1.5}$$

where

$$\mathcal{J}_{\mathrm{cost}}(v) := \frac{1}{N} \sum_{i=1}^{N} \ell(y_i, f_{\mathrm{pred}}(x_i; v)) + \frac{\lambda}{2} \|v\|_{\mathrm{F}}^2, \tag{S-VI.1.6}$$

and $\|\cdot\|_{\mathrm{F}}$ is the Frobenius norm. For example, the squared error function, Eq. (S-V.4.2), the hinge function, Eq. (S-V.4.3), and the cross-entropy loss function, Eq. (S-V.4.4), are often used for $\ell(\cdot, \cdot)$ in Eq. (S-VI.1.6). Then, the optimal v is given by

$$v_* = \arg\min_v \mathcal{J}_{\mathrm{cost}}(v). \tag{S-VI.1.7}$$

B. Ridge classification

As Eq. (S-VI.1.4), we consider the following function to predict the label of x_i:

$$f_{\mathrm{pred}}(x_i; v) = \sum_{j=1}^{G} v_j \phi_j(x_i). \tag{S-VI.2.1}$$

Furthermore, let us consider the squared error function, Eq. (S-V.4.2) for Eq. (S-VI.1.6): In this case, Eq. (S-VI.1.6) becomes

$$\mathcal{J}_{\mathrm{cost}}(v) = \frac{1}{N} \sum_{i=1}^{N} \ell_{\mathrm{SE}}(y_i, f_{\mathrm{pred}}(x_i; v)) + \frac{\lambda}{2} \|v\|_{\mathrm{F}}^2 \tag{S-VI.2.2}$$

$$= \frac{1}{N} \sum_{i=1}^{N} \ell_{\mathrm{SE}}\left(y_i, \sum_{j=1}^{G} v_j \phi_j(x_i) \right) + \frac{\lambda}{2} \sum_{j=1}^{G} |v_j|^2 \tag{S-VI.2.3}$$

$$= \frac{1}{2N} \sum_{i=1}^{N} \left[y_i - \sum_{j=1}^{G} v_j \phi_j(x_i) \right]^2 + \frac{\lambda}{2} \sum_{j=1}^{G} |v_j|^2. \tag{S-VI.2.4}$$

The classification method based on Eq. (S-VI.1.7) with Eq. (S-VI.2.4) is called Ridge classification.

C. Optimal solution in the case of Ridge classification

Here, we consider the optimal solution of Eq. (S-VI.1.7) in the case of Eq. (S-VI.2.4). By solving $\frac{d}{dv}\mathcal{J}_{\rm cost}(v) = 0$, we have

$$v = \frac{1}{\lambda N}\sum_{i=1}^{N}\left[y_i - \sum_{j=1}^{G} v_j\phi_j(x_i)\right]\phi(x_i). \tag{S-VI.3.1}$$

Defining, for $i = 1, 2, \ldots, N$,

$$a_i \coloneqq \frac{1}{\lambda N}\left[y_i - \sum_{j=1}^{G} v_j\phi_j(x_i)\right], \tag{S-VI.3.2}$$

Eq. (S-VI.3.1) is rewritten as

$$v = \sum_{i=1}^{N} a_i\phi(x_i). \tag{S-VI.3.3}$$

Introducing

$$a \coloneqq [a_1, a_2, \ldots, a_N]^\top, \tag{S-VI.3.4}$$

Eq. (S-VI.2.4) is transformed into

$$\begin{aligned}
\mathcal{J}_{\rm cost}(v) &= \frac{1}{2N}\sum_{i=1}^{N}\left[y_i - \sum_{l=1}^{G}\left(\sum_{j=1}^{N} a_j\phi_l(x_j)\right)\phi_l(x_i)\right]^2 + \frac{\lambda}{2}\sum_{l=1}^{G}\left[\sum_{i=1}^{N} a_i\phi_l(x_i)\right]^2 && \text{(S-VI.3.5)}\\
&= \frac{1}{2N}\sum_{i=1}^{N}\left[y_i - \sum_{j=1}^{N} a_j k(x_j, x_i)\right]^2 + \frac{\lambda}{2}\sum_{i,j=1}^{N} a_i k(x_i, x_j) a_j && \text{(S-VI.3.6)}\\
&= \frac{1}{2N}[y - Ka]^\top[y - Ka] + \frac{\lambda}{2}a^\top Ka. && \text{(S-VI.3.7)}
\end{aligned}$$

Here we have used

$$\begin{aligned}
k(x_i, x_j) &\coloneqq \phi^\top(x_i)\phi(x_j), && \text{(S-VI.3.8)}\\
y &\coloneqq [y_1, y_2, \ldots, y_N]^\top, && \text{(S-VI.3.9)}\\
K &\coloneqq \Phi\Phi^\top, && \text{(S-VI.3.10)}\\
\Phi &\coloneqq [\phi(x_1), \phi(x_2), \ldots, \phi(x_N)]^\top. && \text{(S-VI.3.11)}
\end{aligned}$$

Each element of the matrix K is also expressed as

$$[K]_{i,j} = k(x_i, x_j), \tag{S-VI.3.12}$$

where $[\cdot]_{i,j}$ is the element in the i-th row and j-th column. Thus, Eq. (S-VI.1.5) is equivalent to

$$\min_a \mathcal{J}_{\rm cost}(a), \tag{S-VI.3.13}$$

where, as shown in Eq. (S-VI.3.7),

$$\mathcal{J}_{\rm cost}(a) \coloneqq \frac{1}{2N}[y - Ka]^\top[y - Ka] + \frac{\lambda}{2}a^\top Ka. \tag{S-VI.3.14}$$

As the solution of Eq. (S-VI.3.13), we have

$$a_\star = \frac{1}{N}(K + \lambda N I_N)^{-1}y. \tag{S-VI.3.15}$$

where that I_N is the $N \times N$ identity matrix. The proof of Eq. (S-VI.3.15) is as follows.

Proof. From Eq. (S-VI.3.14), the derivative of $\mathcal{J}_{\text{cost}}(a)$ with respect to a is given by

$$\frac{d}{da}\mathcal{J}_{\text{cost}}(a) = -\frac{1}{N}K[y - Ka] + \lambda K a \tag{S-VI.3.16}$$

$$= -\frac{1}{N}K[y - (K + \lambda N I_N)a]. \tag{S-VI.3.17}$$

By setting the derivative of $\mathcal{J}_{\text{cost}}(a)$, Eq. (S-VI.3.17), equal to zero and solving it, we have

$$\frac{d}{da}\mathcal{J}_{\text{cost}}(a_*) = 0 \Leftrightarrow -\frac{1}{N}K[y - (K + \lambda N I_N)a_*] = 0 \tag{S-VI.3.18}$$

$$\Leftrightarrow (K + \lambda N I_N)a_* = y \tag{S-VI.3.19}$$

$$\Leftrightarrow a_* = (K + \lambda N I_N)^{-1} y. \tag{S-VI.3.20}$$

Therefore, we have obtained Eq. (S-VI.3.15).

□

From Eq. (S-VI.3.4) and (S-VI.3.11), we have

$$v = a^\top \Phi. \tag{S-VI.3.21}$$

Then, the optimal $f_{\text{pred}}(x; v_*)$ is written as

$$f_{\text{pred}}(x; v_*) = v_*^\top \phi(x) \tag{S-VI.3.22}$$

$$= a_*^\top \Phi \phi(x) \tag{S-VI.3.23}$$

$$= y^\top (K + \lambda N I_N)^{-1} k(x), \tag{S-VI.3.24}$$

where

$$k(x) \coloneqq \Phi \phi(x) \tag{S-VI.3.25}$$

$$= [k(x_1, x), k(x_2, x), \ldots, k(x_N, x)]^\top. \tag{S-VI.3.26}$$

S-VII. CORRESPONDENCE BETWEEN QCL AND THE KERNEL METHOD

In this section, we explain the correspondence between QCL and the kernel method.

A. Rewriting QCL

In Sec. S-V, we considered $\hat{U}(\theta)$, which is a parametrized unitary operator. To discuss the relationship between QCL and the kernel method, we here use $\hat{U}$ instead of $\hat{U}(\theta)$; then, we rewrite Eqs. (S-V.3.1) and (S-V.3.2), respectively, by

$$\langle \hat{O}_j \rangle_{x_i, \hat{U}} \coloneqq \langle \psi^{\text{out}}(x_i; \hat{U}) | \hat{O}_j | \psi^{\text{out}}(x_i; \hat{U}) \rangle, \tag{S-VII.1.1}$$

and

$$f_{\text{pred}}(x_i; \hat{U}, \theta_{\text{b}}) \coloneqq \sum_{j=1}^{Q} \xi_j \langle \hat{O}_j \rangle_{x_i, \hat{U}} + \theta_{\text{b}}. \tag{S-VII.1.2}$$

Then the estimation problem of $\hat{U}$ and θ_{b} in Eq. (S-IX.1.2) is written as

$$\{\hat{U}_*, \theta_{\text{b},*}\} = \operatorname*{arg\,min}_{\hat{U}, \theta_{\text{b}}} \mathcal{J}_{\text{cost}}(\hat{U}, \theta_{\text{b}}),$$
$$\text{subject to } \hat{U}^\dagger \hat{U} = \hat{1}_{2^n}, \tag{S-VII.1.3}$$

where

$$\mathcal{J}_{\text{cost}}(\hat{U}, \theta_{\text{b}}) \coloneqq \frac{1}{N} \sum_{i=1}^{N} \ell(y_i, f_{\text{pred}}(x_i; \hat{U}, \theta_{\text{b}})), \tag{S-VII.1.4}$$

$\ell(\cdot, \cdot)$ is a loss function, such as the mean-squared error function or the hinge function [30, 31], and $\hat{1}_n$ is the n-dimensional identity operator. In Sec. S-IX, the detail explanation is given.

B. Quadratic form of the kernel method

The conventional kernel method [22, 30, 31] uses a function $\phi(\cdot) : \mathbb{R}^P \to \mathbb{R}^G$ to map any input data point $z_i \in \mathbb{R}^P$ to $\phi(z_i) \in \mathbb{R}^G$, and make a prediction on y_i by

$$f_{\mathrm{pred}}(z_i; v) \coloneqq \sum_{k=1}^{G} v_k \phi_k(z_i), \tag{S-VII.2.1}$$

where $\phi_k(z_i)$ is the k-th element of $\phi(z_i)$, and $v \coloneqq [v_1, v_2, \dots, v_G]^\top$ is a real vector to be optimized. For example, we use the products of all the pairs of the coordinates of z_i to generate a higher dimensional embedding, along with a constant term in a commonly used degree-2 polynomial kernel function. We thus have $G = P^2 + 1$, $\phi_{k+P(l-1)}(z_i) = z_{i,k} \cdot z_{i,l}$, for $k, l = 1, 2, \dots, P$, and $\phi_{(P^2+1)} = 1$. With this choice of a kernel function, Eq. (S-VII.2.1) is expressed as

$$f_{\mathrm{pred}}(z_i; v) \coloneqq \sum_{k=1}^{P^2+1} v_k \phi_k(z_i) \tag{S-VII.2.2}$$

$$\coloneqq \sum_{k,l=1}^{P} (z_{i,k} v_{k+P(l-1)} z_{i,l}) + v_{(P^2+1)}. \tag{S-VII.2.3}$$

Once an embedding has been defined, we minimize the following function to determine v:

$$\mathcal{J}_{\mathrm{cost}}(v) \coloneqq \frac{1}{N} \sum_{i=1}^{N} \ell(y_i, f_{\mathrm{pred}}(z_i; v)). \tag{S-VII.2.4}$$

C. Relationship between QCL and the kernel method

We explain how the optimization problem in Eq. (S-VII.1.3) is written in the form of the kernel method. In the case of VQCs, P defined in Sec. S-VII B becomes 2^n and introducing $\psi_l^{\mathrm{in}}(x_i) \coloneqq \langle l | \psi^{\mathrm{in}}(x_i) \rangle$, $O_{j,(k,l)} \coloneqq \langle k | \hat{O}_j | l \rangle$, and $u_{k,l} \coloneqq \langle k | \hat{U} | l \rangle$ for $k, l = 1, 2, \dots, 2^n$, $\langle \hat{O}_j \rangle_{x_i, \hat{U}}$, which is defined in Eq. (S-VII.1.1), is rewritten as

$$\langle \hat{O}_j \rangle_{x_i, \hat{U}} = \sum_{k,l=1}^{2^n} \psi_k^{\mathrm{in}}(x_i) w_{j,(k,l)} \psi_l^{\mathrm{in}}(x_i), \tag{S-VII.3.1}$$

where, for $k, l = 1, 2, \dots, 2^n$,

$$w_{j,(k,l)} \coloneqq \sum_{k',l'=1}^{2^n} u_{k,k'}^{*} O_{j,(k',l')} u_{l',l}, \tag{S-VII.3.2}$$

$u_k \coloneqq [u_{1,k}, u_{2,k}, \dots, u_{2^n,k}]^{\mathrm{H}}$ for $k = 1, 2, \dots, 2^n$ ($(\cdot)^{\mathrm{H}}$ is the Hermitian conjugate), and $u_k^{\mathrm{H}} u_l = \delta_{k,l}$.

By using Eqs. (S-VII.3.1) and (S-VII.3.2), the prediction function of VQCs in Eq. (S-IX.1.2) is expressed as

$$f_{\mathrm{pred}}(x_i; \hat{U}, \theta_{\mathrm{b}}) \coloneqq \sum_{k,l=1}^{2^n} \psi_k^{\mathrm{in}}(x_i) \left(\sum_{j=1}^{Q} \xi_j w_{j,(k,l)} \right) \psi_l^{\mathrm{in}}(x_i) + \theta_{\mathrm{b}}. \tag{S-VII.3.3}$$

Now, if we compare the VQC prediction function in (S-VII.3.3), to the kernel method prediction function in (S-VII.2.3), we obtain a direct correspondence, where a VQC is reduced to a constrained version of the kernel method, and hence, the kernel method provides an upper bound on the performance of a VQC. Formally, the following choice of $\phi_m(\cdot)$ and v_m in (S-VII.2.1) is required: for $i = 1, 2, \dots, n$, $z_i = \psi^{\mathrm{in}}(x_i)$, for $k, l = 1, 2, \dots, 2^n$,

$$\phi_{k+(l-1)2^n}(x_i) = \psi_k^{\mathrm{in}}(x_i) \psi_l^{\mathrm{in}}(x_i), \tag{S-VII.3.4}$$

$$v_{k+(l-1)2^n} = \sum_{j=1}^{Q} w_{j,(k,l)}, \tag{S-VII.3.5}$$

and

$$\phi_{2^{2n}+1} = 1, \tag{S-VII.3.6}$$

$$v_{2^{2n}+1} = \theta_{\mathrm{b}}. \tag{S-VII.3.7}$$

Note that we also have $P = 2^n$, $G = 2^{2n} + 1$.

S-VIII. SPLITTING METHOD FOR ORTHOGONALITY CONSTRAINED PROBLEMS

The main purpose of this section is to review the method of splitting orthogonality constraints (SOC) proposed in Ref. [32]. The method of SOC is based on Bregman iterative regularization proposed in Refs. [33, 34]. We begin with Bregman iterative regularization and discuss its relation with the augmented Lagrange method. Then, we review the method of SOC.

A. Bregman iterative regularization

The method of SOC proposed in Ref. [32] is based on Bregman iterative regularization proposed in Refs. [33, 34]. We then explain Bregman iterative regularization. Let us consider the following optimization problem:

$$\min_x \mathcal{J}_{\mathrm{cost}}(x), \tag{S-VIII.1.1a}$$

$$\text{subject to } Ax = g, \tag{S-VIII.1.1b}$$

where A is a matrix.

Bregman iterative regularization solves Eq. (S-VIII.1.1) by the following equations:

$$x_{k+1} = \arg\min_x \mathcal{J}_{\mathrm{BIR}}(x; x_k, p_k), \tag{S-VIII.1.2a}$$

$$p_{k+1} = p_k - rA^\top (Ax_{k+1} - g), \tag{S-VIII.1.2b}$$

where

$$\mathcal{J}_{\mathrm{BIR}}(x; y, p) \coloneqq \mathcal{B}^p_{\mathcal{J}_{\mathrm{cost}}(\cdot)}(x, y) + \frac{r}{2}\|Ax - g\|_{\mathrm{F}}^2, \tag{S-VIII.1.3}$$

$$\mathcal{B}^p_{\mathcal{J}_{\mathrm{cost}}(\cdot)}(x, y) \coloneqq \mathcal{J}_{\mathrm{cost}}(x) - \mathcal{J}_{\mathrm{cost}}(y) - \langle p, x - y\rangle. \tag{S-VIII.1.4}$$

Here, r is a positive constant. The performance of Bregman iterative regularization depends on r; thus we need to find an appropriate value of r.

For a better understanding, we elaborate on Eq. (S-VIII.1.2b). We first define

$$\partial \mathcal{J}_{\mathrm{cost}}(y) \coloneqq \left\{ c \middle| \forall x \in \Omega, \mathcal{J}_{\mathrm{cost}}(x) - \mathcal{J}_{\mathrm{cost}}(y) \geq c(x - y) \right\}. \tag{S-VIII.1.5}$$

where Ω is the convex domain of $\mathcal{J}_{\mathrm{cost}}(\cdot)$ [35]. Note that the conventional Bregman divergence is [36]

$$\mathcal{B}_{\mathcal{J}_{\mathrm{cost}}(\cdot)}(x, y) \coloneqq \mathcal{J}_{\mathrm{cost}}(x) - \mathcal{J}_{\mathrm{cost}}(y) - \langle \nabla \mathcal{J}_{\mathrm{cost}}(y), x - y\rangle; \tag{S-VIII.1.6}$$

then Eq. (S-VIII.1.4) can be regarded as the subderivative extension of Eq. (S-VIII.1.6). The derivative of $\mathcal{J}_{\mathrm{BIR}}(x; y, p)$ takes the form

$$\frac{d}{dx}\mathcal{J}_{\mathrm{BIR}}(x; y, p) = \frac{d}{dx}\mathcal{J}_{\mathrm{cost}}(x) - p + rA^\top (Ax - g). \tag{S-VIII.1.7}$$

Then the following relation holds:

$$\frac{d}{dx}\mathcal{J}_{\mathrm{BIR}}(x; y, p) = 0 \Leftrightarrow \frac{d}{dx}\mathcal{J}_{\mathrm{cost}}(x) = p + rA^\top (Ax - g). \tag{S-VIII.1.8}$$

Thus, for $x \in \Omega$, we have

$$p_* \in \partial J_{\mathrm{cost}}(x), \tag{S-VIII.1.9}$$

where

$$p_* \coloneqq p + rA^\top (Ax - g). \tag{S-VIII.1.10}$$

Eq. (S-VIII.1.10) is used for Eq. (S-VIII.1.2b).

For practical calculations, it is convenient to rewrite Eq. (S-VIII.1.2). It can be also expressed as [32]

$$x_{k+1} = \arg\min_x \left[\mathcal{J}_{\text{cost}}(x) + \frac{r}{2}\|Ax - g + b_k\|_{\text{F}}^2 \right], \tag{S-VIII.1.11a}$$

$$b_{k+1} = b_k + Ax_{k+1} - g. \tag{S-VIII.1.11b}$$

By setting $g_k \coloneqq g - b_k$, Eq. (S-VIII.1.11) is also written as [34]

$$x_{k+1} = \arg\min_x \left[\mathcal{J}_{\text{cost}}(x) + \frac{r}{2}\|Ax - g_k\|_{\text{F}}^2 \right], \tag{S-VIII.1.12a}$$

$$g_{k+1} = g_k - (Ax_{k+1} - g). \tag{S-VIII.1.12b}$$

The equivalence between Eqs. (S-VIII.1.2) with $r = 1$ and (S-VIII.1.12) with $r = 1$ is shown as follows.

Proof. For clarity, when we consider the algorithm described by (S-VIII.1.12), we use $\tilde{x}_k$ instead of x_k.

We first assume, for a certain k, $p_k = A^\top(g_k - A\tilde{x}_k)$. From Eq. (S-VIII.1.4), we have

$$\mathcal{B}^{p_k}_{\mathcal{J}_{\text{cost}}(\cdot)}(x, x_k) + \frac{1}{2}\|Ax - g\|_{\text{F}}^2 = \mathcal{J}_{\text{cost}}(x) - \mathcal{J}_{\text{cost}}(x_k) - \langle p_k, x - x_k \rangle + \frac{1}{2}\|Ax - g\|_{\text{F}}^2 \tag{S-VIII.1.13}$$

$$= \mathcal{J}_{\text{cost}}(x) - \langle (g_k - A\tilde{x}_k), Ax \rangle + \frac{1}{2}\|Ax - g\|_{\text{F}}^2 + C_1 \tag{S-VIII.1.14}$$

$$= \mathcal{J}_{\text{cost}}(x) + \frac{1}{2}\|Ax - g - (g_k - A\tilde{x}_k)\|_{\text{F}}^2 + C_2 \tag{S-VIII.1.15}$$

$$= \mathcal{J}_{\text{cost}}(x) + \frac{1}{2}\|Ax - g_{k+1}\|_{\text{F}}^2 + C_2, \tag{S-VIII.1.16}$$

where

$$C_1 \coloneqq -\mathcal{J}_{\text{cost}}(x_k) - \langle p_k, -x_k \rangle, \tag{S-VIII.1.17}$$

$$C_2 \coloneqq C_1 - \frac{1}{2}\|g_k - A\tilde{x}_k\|_{\text{F}}^2. \tag{S-VIII.1.18}$$

Thus, we have shown that Eqs. (S-VIII.1.2) and (S-VIII.1.12) are identical. For details, refer to Ref. [37].

□

B. Correspondence between Bregman iterative regularization and the augmented Lagrange method

It seems meaningful to clarify the relationship between Bregman iterative regularization and a well-known algorithm. Here we explain the penalty method and the augmented Lagrangian method [3, 4]. Then we see the correspondence between Bregman iterative regularization and the augmented Lagrange method. To this end, let us consider the following optimization problem:

$$\min_x \ \mathcal{J}_{\text{cost}}(x), \tag{S-VIII.2.1a}$$

$$\text{subject to } c_i(x) = 0 \ (i = 1, 2, \ldots, m). \tag{S-VIII.2.1b}$$

To find the solution of Eq. (S-VIII.2.1), the penalty method solves

$$\lim_{\lambda \to \infty} \min_x \mathcal{J}_{\text{pm}}(x; \lambda), \tag{S-VIII.2.2}$$

where

$$\mathcal{J}_{\text{pm}}(x; \lambda) \coloneqq \mathcal{J}_{\text{cost}}(x) + \frac{\lambda}{2}\sum_i \|c_i(x)\|_{\text{F}}^2. \tag{S-VIII.2.3}$$

Introducing $\lambda \coloneqq [\lambda_1, \lambda_2, \ldots, \lambda_m]^\top$ and $c(x) \coloneqq [c_1(x), c_2(x), \ldots, c_m(x)]^\top$, the augmented Lagrange method for Eq. (S-VIII.2.1) solves, for $k = 1, 2, \ldots,$

$$\lim_{k \to \infty} \min_x \mathcal{J}_{\text{alm}}(x; \lambda_k), \tag{S-VIII.2.4}$$

where

$$\mathcal{J}_{\mathrm{alm}}(x;\lambda_k) = \mathcal{J}_{\mathrm{cost}}(x) + \lambda_k^\top c(x) + \frac{r}{2}\|c(x)\|_{\mathrm{F}}^2, \tag{S-VIII.2.5}$$

$$\lambda_{k+1} = \lambda_k + rc(x_{k+1}), \tag{S-VIII.2.6}$$

and $r > 0$ is a parameter.

We note that Eq. (S-VIII.1.3) with $r = 1$ and Eq. (S-VIII.2.5) with $r = 1$ are equivalent when $c(x)$ is linear. The proof is shown below.

Proof. From the assumption, we can set

$$c(x) = Ax - g. \tag{S-VIII.2.7}$$

For a certain k, we impose the following relation:

$$p_k = -A^\top \lambda_k. \tag{S-VIII.2.8}$$

Then we have

$$\begin{aligned}
\mathcal{J}_{\mathrm{alm}}(x;\lambda_k) &= \mathcal{J}_{\mathrm{cost}}(x) + \langle \lambda_k, (Ax-g)\rangle + \frac{1}{2}\|Ax-g\|_{\mathrm{F}}^2 && \text{(S-VIII.2.9)}\\
&= \mathcal{J}_{\mathrm{cost}}(x) + \langle \lambda_k, Ax\rangle + \frac{1}{2}\|Ax-g\|_{\mathrm{F}}^2 + C_1 && \text{(S-VIII.2.10)}\\
&= \mathcal{J}_{\mathrm{cost}}(x) - \langle p_k, x\rangle + \frac{1}{2}\|Ax-g\|_{\mathrm{F}}^2 + C_1 && \text{(S-VIII.2.11)}\\
&= \mathcal{J}_{\mathrm{cost}}(x) - \mathcal{J}_{\mathrm{cost}}(x_k) - \langle p_k, (x-x_k)\rangle + \frac{1}{2}\|Ax-g\|_{\mathrm{F}}^2 + \mathcal{J}_{\mathrm{cost}}(x_k) - \langle p_k, x_k\rangle + C_1 && \text{(S-VIII.2.12)}\\
&= \mathcal{B}^{p_k}_{\mathcal{J}_{\mathrm{cost}}(\cdot)}(x, x_k) + \frac{1}{2}\|Ax-g\|_{\mathrm{F}}^2 + C_2. && \text{(S-VIII.2.13)}
\end{aligned}$$

where C_1 and C_2 are constant terms with respect to x given by

$$C_1 \coloneqq -\langle \lambda_k, g\rangle, \tag{S-VIII.2.14}$$

$$C_2 \coloneqq C_1 + \mathcal{J}_{\mathrm{cost}}(x_k) - \langle p_k, x_k\rangle. \tag{S-VIII.2.15}$$

Thus we have shown the equivalence between Eq. (S-VIII.1.3) with $r = 1$ and Eq. (S-VIII.2.5) with $r = 1$.

□

Furthermore, Eq. (S-VIII.2.6) leads to Eq. (S-VIII.1.2b). Therefore, we have confirmed the equivalence between Bregman iterative regularization and the augmented Lagrange method.

C. Penalty method and augmented Lagrangian method for optimization problems with orthogonality constraints

Before getting into the method of SOC, we explain the penalty method and the augmented Lagrangian method for optimization problems with orthogonality constraints [3, 4]. Then let us consider the following optimization problem:

$$\min_X \mathcal{J}_{\mathrm{cost}}(X), \tag{S-VIII.3.1a}$$

$$\text{subject to } X^\top AX = I, \tag{S-VIII.3.1b}$$

where $A \succ O$. Here, I and O are the identity matrix and the zero matrix, respectively.

The penalty method for Eq. (S-VIII.3.1) can be formulated as follows:

$$\lim_{\lambda\to\infty} \min_X \mathcal{J}_{\mathrm{pm}}(X;\lambda), \tag{S-VIII.3.2}$$

where

$$\mathcal{J}_{\mathrm{pm}}(X;\lambda) \coloneqq \mathcal{J}_{\mathrm{cost}}(X) + \frac{\lambda}{2}\|X^\top AX - I\|_{\mathrm{F}}^2. \tag{S-VIII.3.3}$$

The augmented Lagrange method for Eq. (S-VIII.3.1) solves, for $k = 1, 2, \ldots,$

$$\lim_{k\to\infty} \min_X \mathcal{J}_{\mathrm{alm}}(X; \Lambda_k), \tag{S-VIII.3.4}$$

where

$$\mathcal{J}_{\mathrm{alm}}(X; \Lambda_k) \coloneqq \mathcal{J}_{\mathrm{cost}}(X) + \mathrm{Tr}[(\Lambda_k)^\top (X^\top A X - I)] + \frac{r}{2}\|X^\top A X - I\|_{\mathrm{F}}^2, \tag{S-VIII.3.5}$$

$$\Lambda_{k+1} = \Lambda_k + r(X^\top A X - I), \tag{S-VIII.3.6}$$

and $r > 0$ is a parameter.

The main problem with the above approaches is that they are very slow. Then, we review the method of SOC proposed in Ref. [32] in the next subsection.

D. Algorithmic details of the method of SOC

Let us consider the optimization problem given in Eq. (S-VIII.3.1). By splitting the constraints, we first rewrite Eq. (S-VIII.3.1) as

$$\min_{X,P} \mathcal{J}_{\mathrm{cost}}(X), \tag{S-VIII.4.1a}$$

$$\text{subject to } LX = P, \tag{S-VIII.4.1b}$$

$$P^\top P = I, \tag{S-VIII.4.1c}$$

where L is a matrix that satisfies

$$A = L^\top L. \tag{S-VIII.4.2}$$

Next we apply Bregman iterative regularization described in Sec. S-VIII A to Eq. (S-VIII.4.1); then, we solve it by iterating the following equations:

$$\{X_k, P_k\} = \arg\min_{X,P} \mathcal{J}_{\mathrm{SOC}}(X; P, D_{k-1}),$$

$$\text{subject to } P^\top P = I, \tag{S-VIII.4.3a}$$

$$D_k = D_{k-1} + LX_k - P_k, \tag{S-VIII.4.3b}$$

where

$$\mathcal{J}_{\mathrm{SOC}}(X; P, D) \coloneqq \mathcal{J}_{\mathrm{cost}}(X) + \frac{r}{2}\|LX - P + D\|_{\mathrm{F}}^2. \tag{S-VIII.4.4}$$

Here, r is a positive constant. The performance of the method of SOC depends on r; thus, we need to find a good value of r. Note that Eq. (S-VIII.4.3a) comes from Eq. (S-VIII.1.11a).

By dividing Eq. (S-VIII.4.3a) into two equations, we rewrite Eq. (S-VIII.4.3) as follows:

$$X_k = \arg\min_X \mathcal{J}_{\mathrm{SOC}}(X; P_{k-1}, D_{k-1}), \tag{S-VIII.4.5a}$$

$$P_k = \arg\min_P \frac{r}{2}\|P - (LX_k + D_{k-1})\|_{\mathrm{F}}^2,$$

$$\text{subject to } P^\top P = I, \tag{S-VIII.4.5b}$$

$$D_k = D_{k-1} + LX_k - P_k. \tag{S-VIII.4.5c}$$

Note that Eq. (S-VIII.4.5b) comes form the fact that $\mathcal{J}_{\mathrm{cost}}(X)$ does not depend on P:

$$\arg\min_P \frac{r}{2}\|P - (LX_k + D_{k-1})\|_{\mathrm{F}}^2 = \arg\min_P \mathcal{J}_{\mathrm{SOC}}(X_k; P, D_{k-1}). \tag{S-VIII.4.6}$$

Furthermore, the closed form solution of Eq. (S-VIII.4.5b) is already known as shown in Refs. [32, 38, 39]. We first compute

$$K_{1,k}\Sigma_k K_{2,k}^\top = LX_k + D_{k-1}, \tag{S-VIII.4.7}$$

ALGORITHM S-8: Method of splitting orthogonality constraints (SOC)

```
set P_0 and D_0
for k = 1, 2, ..., K do
    compute X_k by Eq. (S-VIII.4.5a)
    compute P_k by Eq. (S-VIII.4.8)
    compute D_k by Eq. (S-VIII.4.5c)
end for
```

where $K_{1,k}$ and $K_{2,k}^{\top}$ are orthogonal matrices and Σ_k is a $p \times q$ matrix, the elements of which in the i-th row and the j-th column for $i \neq j$ are zero, and then we can compute P_k in Eq. (S-VIII.4.5b) in a closed form by

$$P_k = K_{1,k} I_{p,q} K_{2,k}^{\top}. \tag{S-VIII.4.8}$$

We call Eq. (S-VIII.4.5) the method of SOC and it is summarized in Algo. S-8.

To explain Eq. (S-VIII.4.8), we then state the related theorem by following Ref. [32].

Theorem S-1. *Suppose that we have a $p \times q$ matrix Y and let us consider the following problem:*

$$\begin{aligned} P_Y = \arg\min_{P} \frac{1}{2} \|P - Y\|, \\ \text{subject to } P^{\top} P = I. \end{aligned} \tag{S-VIII.4.9}$$

Then, the solution of Eq. (S-VIII.4.9) *is given by*

$$P_Y = K_1 I_{p,q} K_2^{\top}. \tag{S-VIII.4.10}$$

where K_1 and $K_2^{\top}$ satisfy $K_1 \Sigma K_2^{\top} = Y$ and Σ is a diagonal matrix whose diagonal elements are the singular values of Y.

By setting $Y = LX_k + D_{k-1}$ in Eq. (S-VIII.4.9), Eq. (S-VIII.4.10) leads to Eq. (S-VIII.4.8). The proof of Thm. S-1 is given as follows.

Proof. Let us consider the singular value decomposition of Y:

$$Y = K_1 \Sigma K_2^{\top}, \tag{S-VIII.4.11}$$

where K_1 and $K_2^{\top}$ are $p \times p$ and $q \times q$ orthogonal matrices, respectively, and Σ is a $p \times q$ diagonal matrix in the sense that $\{[\Sigma]_{i,i}\}_i$ are the singular values of Y and $[\Sigma]_{i,j} = 0$ for $i \neq j$.

By using K_1 and $K_2^{\top}$ in Eq. (S-VIII.4.11), we define

$$\tilde{P} \coloneqq K_1^{\top} P K_2. \tag{S-VIII.4.12}$$

Then, we have

$$\begin{aligned} \|P - Y\|_{\mathrm{F}}^2 &= \|K_1 (K_1^{\top} P K_2 - \Sigma) K_2^{\top}\|_{\mathrm{F}}^2 && \text{(S-VIII.4.13)} \\ &= \|K_1 (\tilde{P} - \Sigma) K_2^{\top}\|_{\mathrm{F}}^2 && \text{(S-VIII.4.14)} \\ &= \|\tilde{P} - \Sigma\|_{\mathrm{F}}^2. && \text{(S-VIII.4.15)} \end{aligned}$$

Furthermore, from Eq. (S-VIII.4.12), we also have

$$P^{\top} P = I \Leftrightarrow \tilde{P}^{\top} \tilde{P} = I. \tag{S-VIII.4.16}$$

Then, Eq. (S-VIII.4.9) is almost equivalent to

$$\begin{aligned} P_{\Sigma} = \arg\min_{\tilde{P}} \frac{1}{2} \|\tilde{P} - \Sigma\|_{\mathrm{F}}^2, \\ \text{subject to } \tilde{P}^{\top} \tilde{P} = I. \end{aligned} \tag{S-VIII.4.17}$$

It is almost trivial that the solution of Eq. (S-VIII.4.17) takes the following form:

$$P_\Sigma = I_{p,q}, \tag{S-VIII.4.18}$$

where

$$[I_{p,q}]_{k,l} = \begin{cases} 1 & (k = l) \\ 0 & (k \neq l) \end{cases}. \tag{S-VIII.4.19}$$

Here $[\cdot]_{k,l}$ is the element in the k-th row and l-th column. Thus, the closed form solution of Eq. (S-VIII.4.9) is given by

$$P_Y = K_1 P_\Sigma K_2^\top \tag{S-VIII.4.20}$$

$$= K_1 I_{p,q} K_2^\top. \tag{S-VIII.4.21}$$

Thus we have obtained Eq. (S-VIII.4.10).

□

The UKM described in Sec. S-IX is based on the method of SOC. More precisely, the UKM is based on the complex version of the method of SOC.

S-IX. UNITARY KERNEL METHOD

This section describes the UKM, which is one of the main algorithms in this SM. We first provide an overview of the UKM and then discuss how to implement it by using the CG method.

A. Algorithmic details of the UKM

We explain the UKM in this section. We first describe the optimization problem and then state how to solve it.

In the UKM, we make a prediction on y_i by $f_{\mathrm{pred}}(x_i; \hat{U}, \theta_{\mathrm{b}})$. The problem of the UKM is to estimate $\hat{U}$. To do so, similarly to Eq. (S-V.3.2), we consider the following cost function:

$$\mathcal{J}_{\mathrm{cost}}(\hat{U}, \theta_{\mathrm{b}}) \coloneqq \frac{1}{N} \sum_{i=1}^{N} \ell(y_i, f_{\mathrm{pred}}(x_i; \hat{U}, \theta_{\mathrm{b}})), \tag{S-IX.1.1}$$

where

$$f_{\mathrm{pred}}(x_i; \hat{U}, \theta_{\mathrm{b}}) \coloneqq \sum_{j=1}^{Q} \xi_j \langle \hat{O}_j \rangle_{x_i, \hat{U}} + \theta_{\mathrm{b}}, \tag{S-IX.1.2}$$

$$\langle \hat{O}_j \rangle_{x_i, \hat{U}} \coloneqq \langle \psi^{\mathrm{out}}(x_i; \hat{U}) | \hat{O}_j | \psi^{\mathrm{out}}(x_i; \hat{U}) \rangle, \tag{S-IX.1.3}$$

$$|\psi^{\mathrm{out}}(x_i; \hat{U})\rangle \coloneqq \hat{U} |\psi^{\mathrm{in}}(x_i)\rangle, \tag{S-IX.1.4}$$

$$|\psi^{\mathrm{in}}(x_i)\rangle \coloneqq \hat{S}(x_i) |\mathrm{init}\rangle. \tag{S-IX.1.5}$$

Here, $|\mathrm{init}\rangle$ is a initial quantum state, $\{\xi_j\}_{j=1}^{Q}$ are fixed parameters, $\{\hat{O}_j\}_{j=1}^{Q}$ are the set of measurements, $\hat{S}(x_i)$ is the operator to encode x_i on qubits, and $\ell(\cdot,\cdot)$ is a loss function. For example, amplitude encoding is used for $\hat{S}(x_i)$; see Sec. S-V A and Refs. [17, 40]. In addition, the squared error function, Eq. (S-V.4.2), the hinge function, Eq. (S-V.4.3), and the cross-entropy loss function, Eq. (S-V.4.4), are often used for $\ell(\cdot,\cdot)$ [30, 31]. Note that the dimension of $\hat{U}$ can take any positive integer, but to consider the correspondence with quantum computing, we consider a n-qubit system and then the dimension of $\hat{U}$ is 2^n. Here, θ_{b} is a bias term to be estimated, but we may set $\theta_{\mathrm{b}} = 0$ for simplicity. Due to the nature of quantum mechanics, $\hat{U}$ in Eq. (S-IX.1.1) is a unitary operator and, to find a unitary operator, the method of SOC discussed in Sec. S-VIII D is applicable. Similarly to Eq. (S-VIII.3.1), we estimate $\hat{U}$ and θ_{b} by solving the following optimization problem:

$$\min_{\hat{U}, \theta_{\mathrm{b}}} \mathcal{J}_{\mathrm{cost}}(\hat{U}, \theta_{\mathrm{b}}), \tag{S-IX.1.6a}$$

$$\text{subject to } \hat{U}^\dagger \hat{U} = \hat{1}_{2^n}. \tag{S-IX.1.6b}$$

To solve Eq. (S-IX.1.6), we use the method of SOC, Eq. (S-VIII.4.1). Hereafter we denote, by $\hat{X}$, an operator obtained by the method of SOC since it does not strictly satisfy the unitarity condition. Then, we rewrite Eq. (S-IX.1.6) as

$$\min_{\hat{X},\theta_\text{b}} \mathcal{J}_\text{cost}(\hat{X},\theta_\text{b}), \tag{S-IX.1.7a}$$

$$\text{subject to } \hat{X} = \hat{P}, \tag{S-IX.1.7b}$$

$$\hat{P}^\dagger \hat{P} = \hat{1}_{2^n}. \tag{S-IX.1.7c}$$

Note that L in Eq. (S-VIII.4.1) is replaced by the identity operator and the UKM deals with unitary operators though the method of SOC considers the real matrices. Then, we try to explicitly write the update equations for Eq. (S-IX.1.7) by following the method of SOC. From now on, we denote $\hat{X}$, $\hat{P}$, $\hat{D}$, and θ_b at the k-th iteration by $\hat{X}_k$, $\hat{P}_k$, $\hat{D}_k$, and $\theta_{\text{b},k}$, respectively. Like Eqs. (S-VIII.4.5a) and (S-VIII.4.5b), by introducing $\hat{D}$, we split Eq. (S-IX.1.7) into

$$\{\hat{X}_k, \theta_{\text{b},k}\} = \arg\min_{\hat{X},\theta_\text{b}} \mathcal{J}_\text{UKM}(\hat{X},\theta_\text{b};\hat{P}_{k-1},\hat{D}_{k-1}), \tag{S-IX.1.8}$$

and

$$\hat{P}_k = \arg\min_{\hat{P}} \frac{r}{2}\|\hat{P} - (\hat{X}_k + \hat{D}_{k-1})\|_\text{F}^2,$$
$$\text{subject to } \hat{P}^\dagger \hat{P} = \hat{1}_{2^n}, \tag{S-IX.1.9}$$

where

$$\mathcal{J}_\text{UKM}(\hat{X},\theta_\text{b};\hat{P},\hat{D}) \coloneqq \mathcal{J}_\text{cost}(\hat{X},\theta_\text{b}) + \frac{r}{2}\|\hat{X} - \hat{P} + \hat{D}\|_\text{F}^2. \tag{S-IX.1.10}$$

Then, we turn our attention to how to solve Eq. (S-IX.1.9). In Thm. S-1, we limit ourselves to real matrices. Note that, while Thm. S-1 in the previous section and Ref. [32] focus on an orthogonal case, Refs. [38, 39] discuss a unitary case. In the case of operators, Eq. (S-VIII.4.9) is transformed into

$$\hat{P}_* = \arg\min_{\hat{P}} \frac{1}{2}\|\hat{P} - \hat{Y}\|,$$
$$\text{subject to } \hat{P}^\dagger \hat{P} = \hat{1}. \tag{S-IX.1.11}$$

Then, the solution of Eq. (S-IX.1.11) becomes

$$\hat{P}_* = \hat{K}_1 \hat{K}_2^\dagger, \tag{S-IX.1.12}$$

where $\hat{K}_1$ and $\hat{K}_2$ are unitary matrices that satisfy

$$\hat{Y} = \hat{K}_1 \hat{\Sigma} \hat{K}_2^\dagger, \tag{S-IX.1.13}$$

and $\hat{\Sigma}$ is a diagonal operator in the sense that $\{\langle i|\hat{\Sigma}|i\rangle\}_i$ are the singular values of $\hat{Y}$ and $\langle i|\hat{\Sigma}|j\rangle = 0$ for $i \neq j$. In this SM, we call Eq. (S-IX.1.12) with Eq. (S-IX.1.13) operator unitarization (OU) and in the numerical sections, Sec. S-XI, we use OU to obtain matrices that exactly satisfy the unitarity constraint. Thus, to find the solution of Eq. (S-IX.1.9), we first apply the SVD to $\hat{X}_k + \hat{D}_{k-1}$ and compute $\hat{K}_{1,k}$, $\hat{\Sigma}_k$, and $\hat{K}_{2,k}^\dagger$ such that

$$\hat{K}_{1,k}\hat{\Sigma}_k \hat{K}_{2,k}^\dagger = \hat{X}_k + \hat{D}_{k-1}, \tag{S-IX.1.14}$$

where $\hat{K}_{1,k}$ and $\hat{K}_{2,k}^\dagger$ are unitary operators and $\hat{\Sigma}_k$ is a diagonal operator whose diagonal elements are the singular values of $\hat{X}_k + \hat{D}_{k-1}$. We then compute

$$\hat{P}_k = \hat{K}_{1,k}\hat{K}_{2,k}^\dagger. \tag{S-IX.1.15}$$

ALGORITHM S-9: Unitary kernel method (UKM)

```
set P̂_0 and D̂_0
for k = 1, 2, ..., K do
    compute X̂_k and θ_{b,k} by Eq. (S-IX.1.8)
    compute P̂_k by Eq. (S-IX.1.15)
    compute D̂_k by Eq. (S-IX.1.16)
end for
```

Note that the dimensions of $\hat{K}_{1,k}$ and $\hat{K}_{2,k}^{\dagger}$ are identical and thus nothing need to be inserted between $\hat{K}_{1,k}$ and $\hat{K}_{2,k}^{\dagger}$ though $I_{p,q}$ in Eq. (S-VIII.4.19) is inserted between K_1 and $K_2^{\top}$ in Eq. (S-VIII.4.10).

At the end of the k-th iteration, like Eq. (S-VIII.4.5c), we update $\hat{D}_k$ by

$$\hat{D}_k = \hat{D}_{k-1} + \hat{X}_k - \hat{P}_k. \tag{S-IX.1.16}$$

In the UKM, we iterate Eq. (S-IX.1.8), Eq. (S-IX.1.15), and Eq. (S-IX.1.16) until convergence. The UKM is summarized in Algo. S-9 In the next subsection, we elaborate on how to solve Eq. (S-IX.1.8).

B. Derivatives of the cost function

Let us consider the derivatives of Eq. (S-IX.1.1) and (S-IX.1.10). For simplicity, we set $Q = 1$ and $\xi_1 = 1$ in Eq. (S-IX.1.2); we denote the measurement by $\hat{O}$. Furthermore, we define $\hat{\rho}_i := |\psi^{\mathrm{in}}(x_i)\rangle\langle\psi^{\mathrm{in}}(x_i)|$.

First, we consider the derivatives of Eq. (S-IX.1.1). By using Eq. (S-III.2.6), we have

$$\frac{d}{d\Re[\hat{X}]}\mathcal{J}_{\mathrm{cost}}(\hat{X},\theta_{\mathrm{b}}) = \frac{1}{N}\sum_{i=1}^{N}\left[\frac{d}{dz}\ell(y_i,z)\bigg|_{z=f_{\mathrm{pred}}(x_i;\hat{X},\theta_{\mathrm{b}})}\right]\frac{d}{d\Re[\hat{X}]}f_{\mathrm{pred}}(x_i;\hat{X},\theta_{\mathrm{b}}) \tag{S-IX.2.1}$$

$$= \frac{1}{N}\sum_{i=1}^{N}\left[\frac{d}{dz}\ell(y_i,z)\bigg|_{z=f_{\mathrm{pred}}(x_i;\hat{X},\theta_{\mathrm{b}})}\right](\hat{O}\hat{X}\hat{\rho}_i + \hat{O}^{\top}\hat{X}^{*}\hat{\rho}_i^{\top}). \tag{S-IX.2.2}$$

Similarly, by using Eq. (S-III.2.13), we get

$$\frac{d}{d\Im[\hat{X}]}\mathcal{J}_{\mathrm{cost}}(\hat{X},\theta_{\mathrm{b}}) = \frac{1}{N}\sum_{i=1}^{N}\left[\frac{d}{dz}\ell(y_i,z)\bigg|_{z=f_{\mathrm{pred}}(x_i;\hat{X},\theta_{\mathrm{b}})}\right]\frac{d}{d\Im[\hat{X}]}f_{\mathrm{pred}}(x_i;\hat{X},\theta_{\mathrm{b}}) \tag{S-IX.2.3}$$

$$= \frac{1}{N}\sum_{i=1}^{N}\left[\frac{d}{dz}\ell(y_i,z)\bigg|_{z=f_{\mathrm{pred}}(x_i;\hat{X},\theta_{\mathrm{b}})}\right](-i\hat{O}\hat{X}\hat{\rho}_i + i\hat{O}^{\top}\hat{X}^{*}\hat{\rho}_i^{\top}). \tag{S-IX.2.4}$$

Finally, we have

$$\frac{d}{d\theta_{\mathrm{b}}}\mathcal{J}_{\mathrm{cost}}(\hat{X},\theta_{\mathrm{b}}) = \frac{1}{N}\sum_{i=1}^{N}\left[\frac{d}{dz}\ell(y_i,z)\bigg|_{z=f_{\mathrm{pred}}(x_i;\hat{X},\theta_{\mathrm{b}})}\right]\frac{d}{d\theta_{\mathrm{b}}}f_{\mathrm{pred}}(x_i;\hat{X},\theta_{\mathrm{b}}) \tag{S-IX.2.5}$$

$$= \frac{1}{N}\sum_{i=1}^{N}\left[\frac{d}{dz}\ell(y_i,z)\bigg|_{z=f_{\mathrm{pred}}(x_i;\hat{X},\theta_{\mathrm{b}})}\right]. \tag{S-IX.2.6}$$

Thus, we have obtained the derivatives of Eq. (S-IX.1.1).

We also give the derivatives of the square of the Frobenius norm since they appear in Eq. (S-IX.1.8). By using Eqs. (S-III.1.21) and (S-III.1.23), we have

$$\frac{d}{d\Re[\hat{X}]}\|\hat{X}-\hat{Y}\|_{\mathrm{F}}^2 = \frac{d}{d\Re[\hat{X}]}\mathrm{Tr}[(\hat{X}-\hat{Y})^{\dagger}(\hat{X}-\hat{Y})] \tag{S-IX.2.7}$$

$$= (\hat{X}-\hat{Y}) + (\hat{X}-\hat{Y})^{*} \tag{S-IX.2.8}$$

$$= 2\Re[\hat{X}-\hat{Y}]. \tag{S-IX.2.9}$$

Similarly, by using Eqs. (S-III.1.22) and (S-III.1.24), we have

$$\frac{d}{d\Im[\hat{X}]}\|\hat{X}-\hat{Y}\|_{\mathrm{F}}^2 = \frac{d}{d\Im[\hat{X}]}\mathrm{Tr}[(\hat{X}-\hat{Y})^\dagger(\hat{X}-\hat{Y})] \tag{S-IX.2.10}$$

$$= -i(\hat{X}-\hat{Y}) + i(\hat{X}-\hat{Y})^* \tag{S-IX.2.11}$$

$$= 2\Im[\hat{X}-\hat{Y}]. \tag{S-IX.2.12}$$

Finally, we consider the derivatives of Eq. (S-IX.1.10). Eqs. (S-IX.2.2) and (S-IX.2.9) lead to

$$\frac{d}{d\Re[\hat{X}]}\mathcal{J}_{\mathrm{UKM}}(\hat{X},\theta_{\mathrm{b}};\hat{P},\hat{D}) = \frac{1}{N}\left[\frac{d}{dz}\ell(y_i,z)\bigg|_{z=f_{\mathrm{pred}}(x_i;\hat{X},\theta_{\mathrm{b}})}\right](\hat{O}\hat{X}\hat{\rho}_i + \hat{O}^\top\hat{X}^*\hat{\rho}_i^\top) + r\Re[\hat{X}-\hat{P}+\hat{D}], \tag{S-IX.2.13}$$

and Eqs. (S-IX.2.4) and (S-IX.2.12) also lead to

$$\frac{d}{d\Im[\hat{X}]}\mathcal{J}_{\mathrm{UKM}}(\hat{X},\theta_{\mathrm{b}};\hat{P},\hat{D}) = \frac{1}{N}\sum_{i=1}^{N}\left[\frac{d}{dz}\ell(y_i,z)\bigg|_{z=f_{\mathrm{pred}}(x_i;\hat{X},\theta_{\mathrm{b}})}\right](-i\hat{O}\hat{X}\hat{\rho}_i + i\hat{O}^\top\hat{X}^*\hat{\rho}_i^\top) + r\Im[\hat{X}-\hat{P}+\hat{D}]. \tag{S-IX.2.14}$$

The second term of Eq. (S-IX.1.10) does not depend on θ_{b}. Thus, similarly to Eq. (S-IX.2.6), we have

$$\frac{d}{d\theta_{\mathrm{b}}}\mathcal{J}_{\mathrm{UKM}}(\hat{X},\theta_{\mathrm{b}};\hat{P},\hat{D}) = \frac{1}{N}\sum_{i=1}^{N}\left[\frac{d}{dz}\ell(y_i,z)\bigg|_{z=f_{\mathrm{pred}}(x_i;\hat{X},\theta_{\mathrm{b}})}\right]. \tag{S-IX.2.15}$$

Thus, we have obtained the derivatives of Eq. (S-IX.1.10).

Without specifying $\ell(\cdot,\cdot)$ in Eq. (S-IX.1.1), we cannot go further. Then let us consider the squared error function, Eq. (S-V.4.2), for $\ell(\cdot,\cdot)$ in Eq. (S-IX.1.1); the derivative of the loss function takes the form

$$\frac{d}{dz}\ell_{\mathrm{SE}}(y_i,z)\bigg|_{z=f_{\mathrm{pred}}(x_i;\hat{X},\theta_{\mathrm{b}})} = -[y_i - f_{\mathrm{pred}}(x_i;\hat{X},\theta_{\mathrm{b}})]. \tag{S-IX.2.16}$$

Substituting Eq. (S-IX.2.16) into Eqs. (S-IX.2.13) and (S-IX.2.14), we obtain the derivatives of Eq. (S-IX.1.10) in the case of the squared error function, Eq. (S-V.4.2).

We employ the **optimize** function in the SciPy package [41] to solve Eq. (S-IX.1.8). The SciPy package [41] provides the **optimize** function, in which the CG method, the BFGS method, and other optimization methods are implemented. Eqs. (S-IX.2.13) and (S-IX.2.14) help it run faster.

C. UKM with the nonlinear CG method

We have explained the UKM in the previous subsection. Here we state how to solve Eq. (S-IX.1.8).

We first define the $2N^2+1$-dimensional real vector $\tilde{x}$ whose element is given by

$$[\tilde{x}]_{2(N(i-1)+(j-1))+1} := \Re[\langle i|\hat{X}|j\rangle], \tag{S-IX.3.1a}$$

$$[\tilde{x}]_{2(N(i-1)+(j-1))+2} := \Im[\langle i|\hat{X}|j\rangle], \tag{S-IX.3.1b}$$

$$[\tilde{x}]_{2N^2+1} := \theta_{\mathrm{b}}, \tag{S-IX.3.1c}$$

for $i,j=1,2,\ldots,N$, where N is the dimension of $\hat{X}$. From Eq. (S-IX.1.1) and Eq. (S-IX.1.8), we then define

$$\mathcal{J}_{\mathrm{CG}}(\tilde{x}) := \mathcal{J}_{\mathrm{cost}}(\hat{X},\theta_{\mathrm{b}}) + \frac{r}{2}\|\hat{X}-\hat{P}_{k-1}+\hat{D}_{k-1}\|_{\mathrm{F}}^2. \tag{S-IX.3.2}$$

Then, similarly to Eq. (S-IV.2.2), we solve Eq. (S-IX.1.8) by

$$\tilde{x}_k = \tilde{x}_{k-1} + \alpha_k d_{k-1}, \tag{S-IX.3.3a}$$

$$d_k = -\nabla\mathcal{J}_{\mathrm{CG}}(\tilde{x}_k) + \beta_k d_{k-1}. \tag{S-IX.3.3b}$$

Here we use the CG method: Eq. (S-IV.2.4) for α_k and Eq. (S-IV.2.5) for β_k, since the BFGS method requires a large memory space then the CG method and for the case of a large number of qubits, the memory space problem becomes severe. Note that Eqs. (S-III.2.6) and (S-III.2.13) are available to compute $\nabla\mathcal{J}_{\mathrm{cost}}(\hat{X},\theta_{\mathrm{b}})$ and $\nabla\mathcal{J}_{\mathrm{CG}}(\tilde{x})$. Then, we compute $\hat{X}_k$ from $\tilde{x}$ by using by Eq. (S-IX.3.1). Furthermore, we repeat the SOC update K times and the CG update K' times.

We summarize the UKM with the CG method in Algo. S-10. In this SM, we perform the UKM with the CG method, Algo. S-10, and see its performance in Sec. S-XI.

ALGORITHM S-10: Unitary kernel method (UKM) with the conjugate gradient (CG) method

```
set P̂_0 and D̂_0
for k = 1, 2, ..., K do
    initialize x̃_0 by Eq. (S-IX.3.1)
    set d_0 = −∇J_CG(x̃_0)
    for k' = 1, 2, ..., K' do
        compute x̃_{k'} by Eq. (S-IX.3.3a)
        compute d_{k'} by Eq. (S-IX.3.3b)
    end for
    compute X̂_k and θ_{b,k} from x̃_{K'} by Eq. (S-IX.3.1)
    compute P̂_k by Eq. (S-IX.1.9)
    compute D̂_k by Eq. (S-IX.1.15)
end for
```

S-X. VARIATIONAL CIRCUIT REALIZATION

In the literature, some novel unitary decomposition methods were proposed: the quantum Shannon decomposition (QSD) [42], the column-by-column decomposition [2], Knill's decomposition [26, 43], column-by-column decomposition [44], etc. Despite their usefulness, they require an exponentially large number of the CNOT gates when the number of qubits n increases.

In this section, we propose a variational method to obtain a quantum circuit that realizes a given unitary operator, which we call the VCR.

A. Algorithmic details of the VCR

We first assume that a target unitary and a quantum circuit that has the set of parameters. Then, let $\hat{U}$ and $\hat{U}_{\mathrm{c}}(\theta; L)$ be the target unitary operator and the unitary operator composed of gates that are parametrized by $\theta \coloneqq [\theta_1, \theta_2, \dots]^{\top}$, respectively. So far, we have denoted, by $\hat{U}_{\mathrm{c}}(\theta)$, the unitary realized by a quantum circuit, but we denote it by $\hat{U}_{\mathrm{c}}(\theta; L)$ to emphasize the number of layers L in this section.

In the VCR, we construct a cost function and estimate θ by minimizing the cost function. For example, we can estimate θ by

$$\{\theta_*, \lambda_*\} = \arg\min_{\theta,\lambda} \tilde{\mathcal{J}}_{\mathrm{cost}}(\theta, \lambda; L, p, \hat{U}), \tag{S-X.1.1}$$

where, for arbitrary $p > 0$,

$$\tilde{\mathcal{J}}_{\mathrm{cost}}(\theta, \lambda; L, p, \hat{U}) \coloneqq \|\hat{U} - \hat{U}_{\mathrm{c+p}}(\theta, \lambda; L)\|_{\mathrm{F}}^{p}. \tag{S-X.1.2}$$

The global phase does not matter physically, but it matters for approximating a unitary operator; then we have used, in Eq. (S-X.1.2), the unitary operator $\hat{U}_{\mathrm{c+p}}(\theta, \lambda; L)$ defined by

$$\hat{U}_{\mathrm{c+p}}(\theta, \lambda; L) \coloneqq \hat{\Phi}_{2^n}(\lambda)\hat{U}_{\mathrm{c}}(\theta; L), \tag{S-X.1.3}$$

where $\hat{\Phi}_{2^n}(\lambda) \coloneqq e^{-i\lambda}\hat{1}_{2^n}$.

We give another example of a cost function. When $\hat{U}$ and $\hat{U}_{\mathrm{c+p}}(\theta, \lambda; L)$ are identical, we have

$$\hat{U}^{\dagger}\hat{U}_{\mathrm{c+p}}(\theta, \lambda; L) = \hat{1}_{2^n}. \tag{S-X.1.4}$$

Then, we can estimate θ by

$$\{\theta_*, \lambda_*\} = \arg\min_{\theta,\lambda} \mathcal{J}_{\mathrm{cost}}(\theta, \lambda; L, p, \hat{U}), \tag{S-X.1.5}$$

where, for any $p > 0$,

$$\mathcal{J}_{\mathrm{cost}}(\theta, \lambda; L, p, \hat{U}) \coloneqq \|\hat{U}^{\dagger}\hat{U}_{\mathrm{c+p}}(\theta, \lambda; L) - \hat{1}_{2^n}\|_{\mathrm{F}}^{p}. \tag{S-X.1.6}$$

In a circuit realization, the complexity of a circuit is of great interest. In this paper, we assume a layered structure for a quantum circuit. Thus, given an error threshold δ, it is convenient to define L_δ:

$$L_\delta = \arg\min_{L} \epsilon_L, \quad \text{subject to } \epsilon_L \le \delta, \tag{S-X.1.7}$$

where

$$\epsilon_L := \min_{\theta,\lambda} \mathcal{J}_{\rm cost}(\theta,\lambda;L,p,\hat{U}). \tag{S-X.1.8}$$

The advantage of the VCR is that the number of the CNOT gates is expected to be small though the number of the CNOT gates exponentially grows in the QSD. In Sec. S-XII, we perform the VCR and see its performance.

B. Derivatives of the cost functions

To employ the **optimize** function in the SciPy package [41], the derivative of a cost function is required for fast computing. We explain the derivatives of Eqs. (S-X.1.2) and (S-X.1.6).

From Eq. (S-II.2.3), we can rewrite the Frobenius norm by using the trace operator; then Eqs. (S-X.1.2) and (S-X.1.2) for $p=2$ become, respectively,

$$\tilde{\mathcal{J}}_{\rm cost}(\theta,\lambda;L,2,\hat{U}) = {\rm Tr}\Big[\Big(\hat{U}-\hat{U}_{\rm c+p}(\theta,\lambda;L)\Big)^\dagger\Big(\hat{U}-\hat{U}_{\rm c+p}(\theta,\lambda;L)\Big)\Big], \tag{S-X.2.1}$$

$$\mathcal{J}_{\rm cost}(\theta,\lambda;L,2,\hat{U}) = {\rm Tr}\Big[\Big(\hat{U}^\dagger\hat{U}_{\rm c+p}(\theta,\lambda;L)-\hat{1}_{2^n}\Big)^\dagger\Big(\hat{U}^\dagger\hat{U}_{\rm c+p}(\theta,\lambda;L)-\hat{1}_{2^n}\Big)\Big]. \tag{S-X.2.2}$$

Due to the linearity of the trace operation, the following relation holds:

$$\frac{d}{d\theta_i}{\rm Tr}[\hat{A}(\theta)] = {\rm Tr}\Big[\frac{d}{d\theta_i}\hat{A}(\theta)\Big]. \tag{S-X.2.3}$$

Then we have

$$\frac{d}{d\theta_i}\tilde{\mathcal{J}}_{\rm cost}(\theta,\lambda;L,2,\hat{U}) = -{\rm Tr}\Big[\frac{d}{d\theta_i}\hat{U}^\dagger_{\rm c+p}(\theta,\lambda;L)\Big(\hat{U}-\hat{U}_{\rm c+p}(\theta,\lambda;L)\Big)\Big] - {\rm Tr}\Big[\Big(\hat{U}-\hat{U}_{\rm c+p}(\theta,\lambda;L)\Big)^\dagger\frac{d}{d\theta_i}\hat{U}_{\rm c+p}(\theta,\lambda;L)\Big], \tag{S-X.2.4}$$

and

$$\begin{aligned}&\frac{d}{d\theta_i}\mathcal{J}_{\rm cost}(\theta,\lambda;L,2,\hat{U})\\ &= {\rm Tr}\Big[\Big(\hat{U}^\dagger\frac{d}{d\theta_i}\hat{U}_{\rm c+p}(\theta,\lambda;L)\Big)^\dagger\Big(\hat{U}^\dagger\hat{U}_{\rm c+p}(\theta,\lambda;L)-\hat{1}_{2^n}\Big)\Big] + {\rm Tr}\Big[\Big(\hat{U}^\dagger\hat{U}_{\rm c+p}(\theta,\lambda;L)-\hat{1}_{2^n}\Big)^\dagger\Big(\hat{U}^\dagger\frac{d}{d\theta_i}\hat{U}_{\rm c+p}(\theta,\lambda;L)\Big)\Big].\end{aligned} \tag{S-X.2.5}$$

Then we compute $\frac{d}{d\theta_i}\hat{U}_{\rm c+p}(\theta,\lambda;L)$ in Eqs. (S-X.2.4) and (S-X.2.5).

S-XI. NUMERICAL SIMULATION OF QCL, THE UKM, AND THE KERNEL METHOD

In this section, we show numerical simulations of QCL, the UKM, and the kernel method for several datasets. We first describe the numerical setting and the properties of datasets. Then, we show the results.

A. Numerical setting

To evaluate the performance of QCL, the UKM, and the kernel method, we use 5-fold cross-validation (CV) with 5 different random initial conditions. For each method, we select the best model for the training dataset over iterations to compute the performance.

For the UKM, we show the performance of $\hat{X}$, $\hat{P}$, and OU of $\hat{X}$. Refer to Sec. S-VIII D for the details of OU. Furthermore, we consider real and complex matrices as initial inputs.

For QCL we show the performance of the CNOT-based circuit, the CRot-based circuit, the 1d Heisenberg circuit, and the FC Heisenberg circuit. Refer to Sec. S-V B for details. In the case of the Heisenberg circuits, we set $\Delta t = 0.1$ in Eq. (S-V.2.23). For QCL, we utilize the stochastic gradient descent method [31].

To create $|\psi^{\mathrm{in}}(x_i)\rangle$ in Eq. (S-V.1.2) and (S-IX.1.5), we need to fix $\hat{S}(x_i)$. For $\hat{S}(x_i)$ for $i = 1, 2, \ldots, N$, we use amplitude encoding with $c_i^k = 0$ in Eq. (S-V.1.4) for all i and k, described in Sec. S-V A. For $\ell(\cdot,\cdot)$ in Eqs. (S-V.4.1) and (S-IX.1.1), we utilize the squared error function, Eq. (S-V.4.2). Furthermore, we set $Q = 1$ and $\xi_1 = 1$ in Eqs. (S-V.3.2) and (S-IX.1.2), and consider two cases on θ_{b} in Eqs. (S-V.3.2) and (S-IX.1.2): the cases of fixing $\theta_{\mathrm{b}} = 0$ and estimating θ_{b}.

We use the Pennylane package [45] for QCL and the scikit-learn packaged [46] for the kernel method.

B. Datasets

In this section, the following six datasets are used to compare the performance of QCL, the UKM, and the kernel method: the iris dataset, the cancer dataset, the wine dataset, the sonar dataset, the semeion dataset in the UCI repository [47], and the MNIST dataset [48].

The original MNIST dataset has 28×28 dimensions. We create the MNIST256 dataset by reducing its dimensions to 16×16 using coarse-graining.

We consider the binary classification problem. The cancer dataset has two labels, (0) "B" and (1) "M", and the wine dataset has two labels, (0) "R" and (1) "M".

On the other hand, other datasets have more labels. The iris dataset has three labels: (0) Iris-setosa, (1) Iris-versicolor, and (2) Iris-virginica. When we deal with the classification problem between (0) Iris-setosa and (1) Iris-versicolor, we write the dataset by Iris (0 or 1). When we deal with the classification problem between (0) Iris-setosa and the others, we write it by Iris (0 or non-0). Similarly, when we deal with the classification problem between (1) Iris-setosa and the others, we write the dataset by Iris (1 or non-1).

The wine dataset has three labels: (0) class 1, (1) class 2, and (3) class 3. We consider the classification problem between (0) class 1 and others, we write it as the wine dataset (0 or non-0).

The semeion dataset and the MNIST256 dataset has ten labels from 0 to 9. We write it as (0 or 1) when we consider the classification problem between 0 and 1, and we write it as (0 or non-0) when we consider the classification problem between 0 and the others.

At the end of this subsection, the numbers of datapoints N and dimensions M of the datasets are shown in Table S-1.

ID	N	M	n
Iris (0 or 1)	100	4	2
Iris (0 or non-0)	150	4	2
Iris (1 or non-1)	150	4	2
Cancer (0 or 1)	569	30	5
Sonar (0 or 1)	208	60	6
Wine (0 or non-0)	178	14	4
Semeion (0 or 1)	323	256	8
Semeion (0 or non-0)	1593	256	8
MNIST256 (0 or 1)	569	256	8
MNIST256 (0 or non-0)	2766	256	8

TABLE S-1: Numbers of datapoints N dimensions M of the datasets and the number of qubits n required for amplitude encoding. Note that $n = \lceil \log_2 M \rceil$.

C. Summary

The results for the datasets in Table S-1 are presented in Table S-2. Table S-2 shows that the results obtained by the UKM ($\hat{P}$ and OU of $\hat{X}$) are better than those obtained by QCL and that they are bound by the results obtained by the UKM ($\hat{X}$) and the kernel method. This is consistent with the analytical results discussed above. The detailed results are shown in the rest of this section.

Dataset	– Variational quantum classifiers – UKM ($\hat{P}$)	UKM (OU of $\hat{X}$)	QCL	– Classical classifiers – UKM ($\hat{X}$)	Kernel method
Iris (0 or 1)	1.0000/**1.0000**	1.0000/**1.0000**	1.0000/**1.0000**	1.0000/1.0000	1.0000/1.0000
Iris (0 or non-0)	1.0000/0.9987	1.0000/**1.0000**	1.0000/**1.0000**	1.0000/1.0000	1.0000/1.0000
Iris (1 or non-1)	0.7880/0.7789	0.7953/**0.7994**	0.6801/0.5872	0.9781/0.9618	0.9751/0.9666
Cancer (0 or 1)	0.9194/**0.9131**	0.9184/0.9115	0.8797/0.8768	0.9218/0.9160	0.9618/0.9568
Sonar (0 or 1)	0.9159/**0.7985**	0.9175/0.7909	0.7455/0.6924	0.8903/0.7774	1.0000/0.8198
Wine (0 or non-0)	0.9200/**0.9185**	0.9212/0.9171	0.9155/0.9126	0.9364/0.9313	0.9987/0.9955
Semeion (0 or 1)	1.0000/0.9943	1.0000/**0.9945**	0.9210/0.9099	1.0000/0.9957	1.0000/1.0000
Semeion (0 or non-0)	0.9988/0.9949	0.9990/**0.9953**	0.8989/0.8982	0.9969/0.9925	1.0000/0.9955
MNIST256 (0 or 1)	0.9991/**0.9969**	1.0000/0.9951	0.9511/0.9459	0.9985/0.9966	1.0000/1.0000
MNIST256 (0 or non-0)	0.9922/0.9871	0.9927/**0.9889**	0.9053/0.9050	0.9894/0.9859	0.9992/0.9953

TABLE S-2: Results of 5-fold CV with 5 different random seeds of the UKM ($\hat{X}$, $\hat{P}$, and OU of $\hat{X}$), QCL, and the kernel method for all the datasets. The performance cells are of the format "training performance/test performance." We choose the model that shows the best test performance for each algorithm. For the UKM, we consider the complex and real cases with and without the bias term. We set $r = 0.010$. For QCL, we consider the CNOT-based, CRot-based, 1d-Heisenberg, and FC-Heisenberg circuits with and without the bias term for the iris, cancer, sonar, and wine datasets, and the CNOT-based and CRot-based circuits with and without the bias term for the semeion and MNIST256 datasets. We set the number of layers L to 5. For $\phi(\cdot)$ in the kernel method, we consider linear and quadratic functions with and without the bias term for $\lambda = 10^{-2}, 10^{-1}, 1$. The values of the best VQC for each dataset are printed in bold.

D. Iris dataset (0 or 1)

We here show the numerical result for the iris dataset (0 or 1). For the UKM, we put $r = 0.010$ and set $K = 30$ and $K' = 10$ in Algo. S-10. For QCL, we run iterations 300 times. We use the squared error function $\ell_{\mathrm{SE}}(\cdot,\cdot)$, Eq. (S-V.4.2).

In Fig. S-1, we show the numerical results of QCL for the 5-fold datasets with 5 different random seeds. In Fig. S-2,

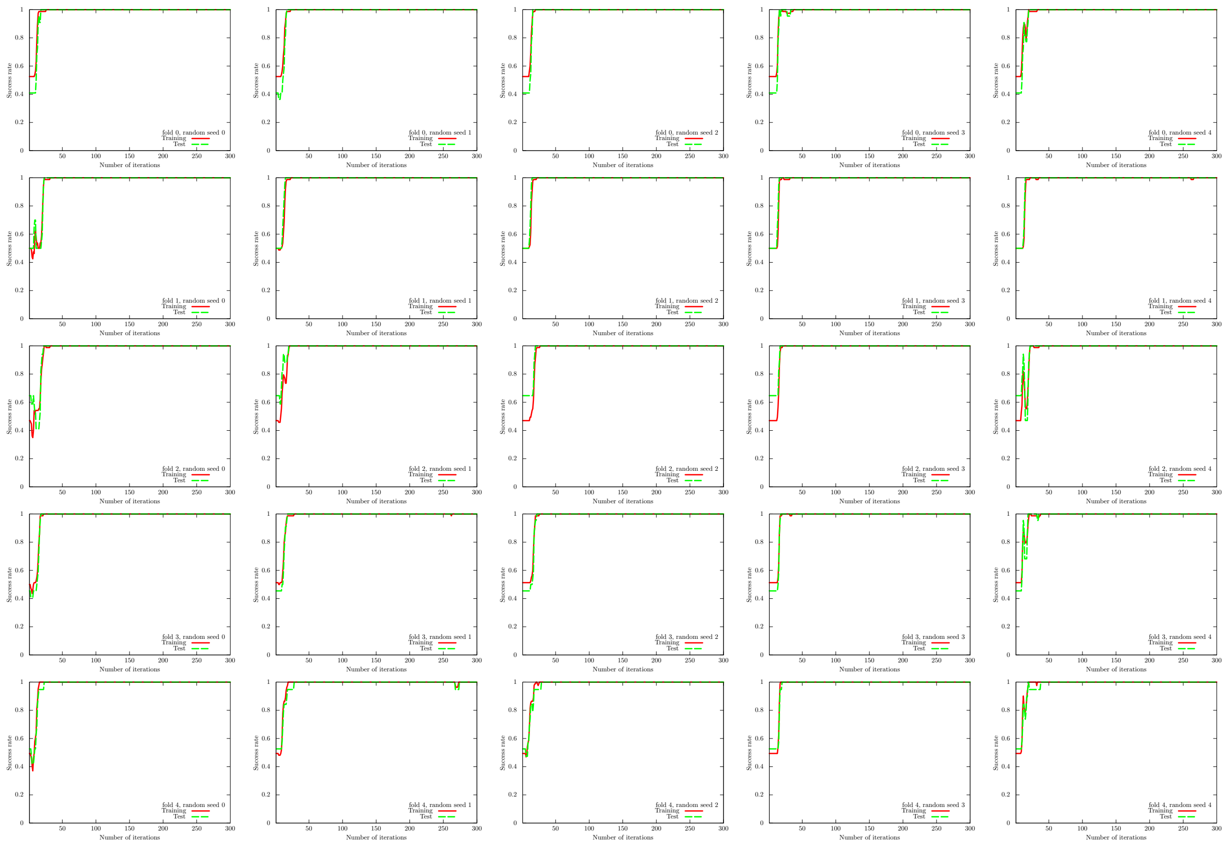

FIG. S-1: Results of QCL on the 5-fold datasets with 5 different random seeds for the iris dataset (0 or 1). We use the CNOT-based circuit and set $\theta_{\mathrm{bias}} = 0$. The number of layers L is set to 5.

we show the numerical results of $\hat{P}$ of the UKM for the 5-fold datasets with 5 different random seeds. In Fig. S-3, we also show the numerical results of OU of $\hat{X}$ of the UKM for the 5-fold datasets with 5 different random seeds.

We summarize the results of 5-fold CV with 5 different random seeds of QCL and the UKM in Tables S-3 and S-4, respectively. For QCL and the UKM, we select the best model for the training dataset over iterations to compute the performance. In Fig. S-4, we plot the data shown in Tables S-3 and S-4. We also summarize the results of 5-fold CV

Algo.	Condition	Training	Test
QCL	CNOT-based, w/o bias	1.0	1.0
QCL	CNOT-based, w/ bias	1.0	1.0
QCL	CRot-based, w/o bias	1.0	0.9953
QCL	CRot-based, w/ bias	1.0	0.9982
QCL	1d Heisenberg, w/o bias	1.0	0.9895
QCL	1d Heisenberg, w/ bias	1.0	0.9874
QCL	FC Heisenberg, w/o bias	1.0	0.9895
QCL	FC Heisenberg, w/ bias	1.0	0.9874

TABLE S-3: Results of 5-fold CV with 5 different random seeds of QCL for the iris dataset (0 or 1). We consider four types of circuits with and without the bias term: the CNOT-based circuit, the CRot-based circuit, 1d Heisenberg circuit, and the FC Heisenberg circuit. The number of layers L is set to 5 and the number of iterations is set to 300.

with 5 different random seeds of the kernel method in Table S-5. More specifically, we use Ridge classification, which

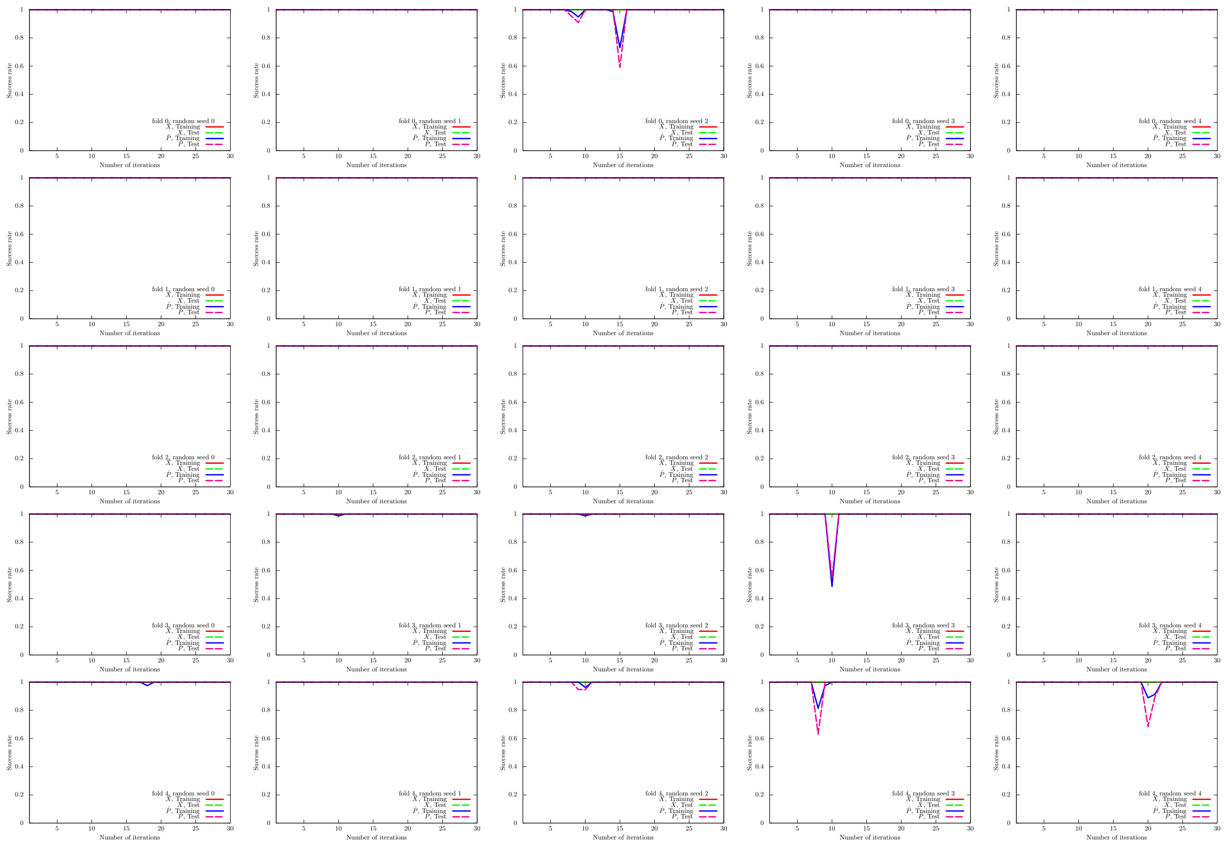

FIG. S-2: Results of the UKM ($\hat{X}$ and $\hat{P}$) on the 5-fold datasets with 5 different random seeds for the iris dataset (0 or 1). We use complex matrices for the initial input and set $\theta_{\text{bias}} = 0$. We set $r = 0.010$.

Algo.	Condition	Training	Test
UKM	$\hat{X}$, complex, w/o bias	1.0	1.0
UKM	$\hat{P}$, complex, w/o bias	1.0	1.0
UKM	OU of $\hat{X}$, complex, w/ bias	1.0	1.0
UKM	$\hat{X}$, complex, w/ bias	1.0	1.0
UKM	$\hat{P}$, complex, w/ bias	1.0	1.0
UKM	OU of $\hat{X}$, real, w/o bias	1.0	1.0
UKM	$\hat{X}$, real, w/o bias	1.0	1.0
UKM	$\hat{P}$, real, w/o bias	1.0	1.0
UKM	OU of $\hat{X}$, real w/o bias	1.0	1.0
UKM	$\hat{X}$, real, w/ bias	1.0	1.0
UKM	$\hat{P}$, real, w/ bias	1.0	0.9953
UKM	OU of $\hat{X}$, real, w/ bias	1.0	0.9953

TABLE S-4: Results of 5-fold CV with 5 different random seeds of the UKM for the iris dataset (0 or 1). We show the performance obtained by $\hat{X}$, $\hat{P}$, and OU of $\hat{X}$. Note that $\hat{P}$, and OU of $\hat{X}$ strictly satisfy the unitarity constraint while $\hat{X}$ does not. We consider real and complex matrices for the initial input with and without the bias term. We put $r = 0.010$ and set $K = 30$ and $K' = 10$.

is described in Sec. S-VI B. We consider linear and quadratic functions for $\phi(\cdot)$ in Eq. (S-VI.2.1) with and without normalization. We set $\lambda = 10^{-2}, 10^{-1}, 1$ where λ is the coefficient of the regularization term.

Next, we show the performance dependence of the three algorithms on their key parameters. We see the performance dependence of QCL on the number of layers L. The result is shown in Fig. S-5. We then see the performance dependence of the UKM on r, which is the coefficient of the second term in the right-hand side of Eq. (S-IX.1.8). The result is shown in Fig. S-6. In Fig. S-7, we show the performance dependence of the kernel method on λ, which is the

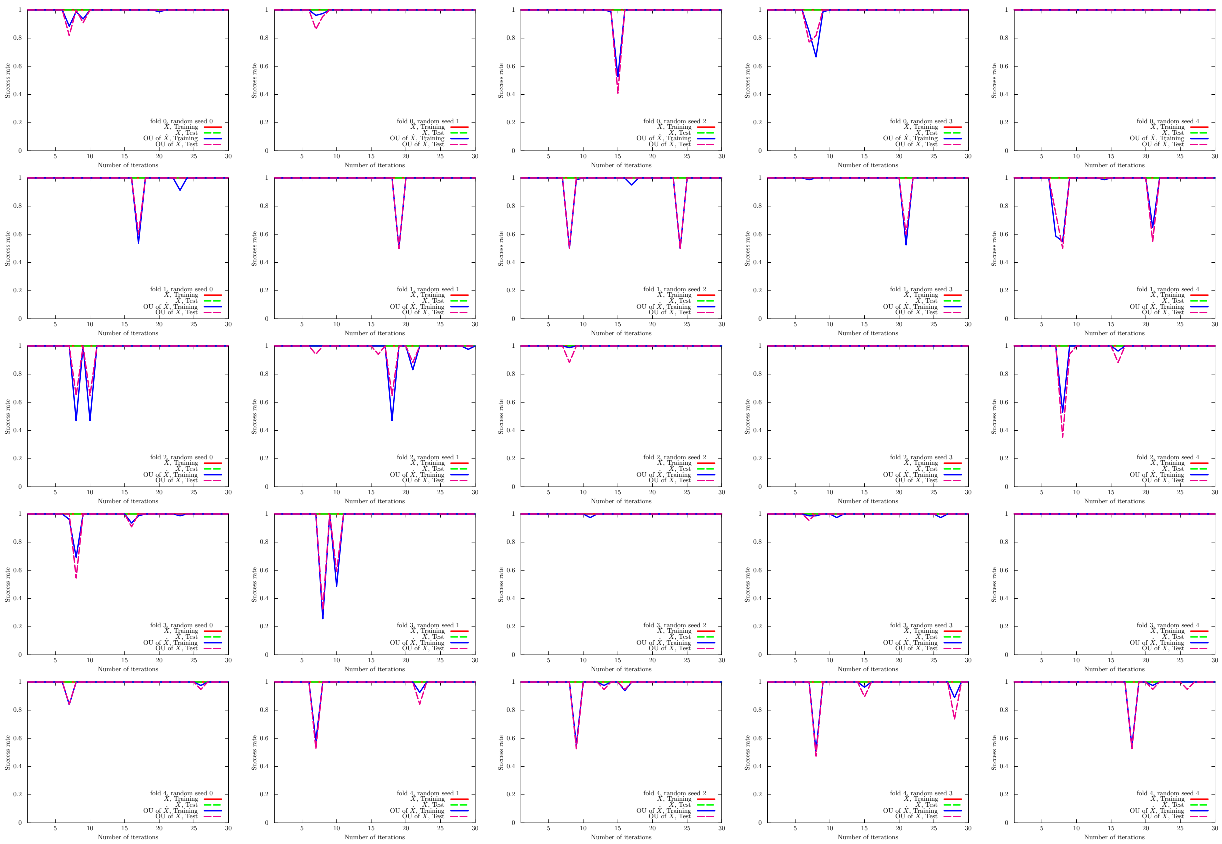

FIG. S-3: Results of the UKM ($\hat{X}$ and OU of $\hat{X}$) on the 5-fold datasets with 5 different random seeds for the iris dataset (0 or 1). We use complex matrices for the initial input and set $\theta_{\text{bias}} = 0$. We set $r = 0.010$.

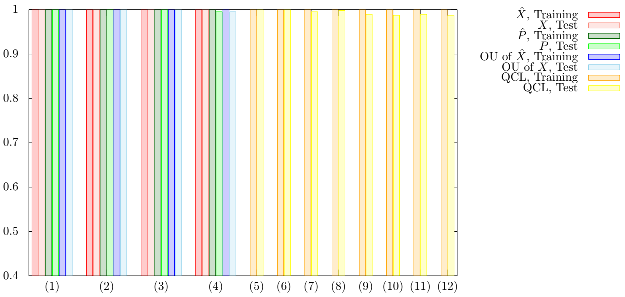

FIG. S-4: Results of 5-fold CV with 5 different random seeds for the iris dataset (0 or 1). For the UKM, we put $r = 0.010$ and set $K = 30$ and $K' = 10$. For QCL, the number of layers L is 5 and the number of iterations is 300. The numerical settings are as follows: (1) UKM: complex matrices without the bias term, (2) UKM: complex matrices with the bias term, (3) UKM: real matrices without the bias term, (4) UKM: real matrices with the bias term, (5) QCL: CNOT-based circuit without the bias term, (6) QCL: CNOT-based circuit with the bias term, (7) QCL: CRot-based circuit without the bias term, (8) QCL: CRot-based circuit with the bias term, (9) QCL: 1d Heisenberg circuit without the bias term, (10) QCL: 1d Heisenberg circuit with the bias term, (11) QCL: FC Heisenberg circuit without the bias term, and (12) QCL: FC Heisenberg circuit with the bias term.

coefficient of the second term in the right-hand side of Eq. (S-VI.2.4).

So far, we have used the squared error function $\ell_{\text{SE}}(\cdot,\cdot)$, Eq. (S-V.4.2). In Fig. S-8, we show the performance dependence of QCL on the number of layers L in the case of the hinge function $\ell_{\text{hinge}}(\cdot,\cdot)$, Eq. (S-V.4.3). In Fig. S-9, we show the performance dependence of the UKM on r, which is the coefficient of the second term in the right-hand side of Eq. (S-IX.1.8), in the case of the hinge function $\ell_{\text{hinge}}(\cdot,\cdot)$, Eq. (S-V.4.3).

Algo.	Condition	Training	Test
Kernel method	Linear, w/o normalization, $\lambda = 10^{-2}$	1.0000	1.0000
Kernel method	Linear, w/o normalization, $\lambda = 10^{-1}$	1.0000	1.0000
Kernel method	Linear, w/o normalization, $\lambda = 1$	1.0000	1.0000
Kernel method	Linear, w/ normalization, $\lambda = 10^{-2}$	1.0000	1.0000
Kernel method	Linear, w/ normalization, $\lambda = 10^{-1}$	1.0000	1.0000
Kernel method	Linear, w/ normalization, $\lambda = 1$	1.0000	1.0000
Kernel method	Quadratic, w/o normalization, $\lambda = 10^{-2}$	1.0000	1.0000
Kernel method	Quadratic, w/o normalization, $\lambda = 10^{-1}$	1.0000	1.0000
Kernel method	Quadratic, w/o normalization, $\lambda = 1$	1.0000	1.0000
Kernel method	Quadratic, w/ normalization, $\lambda = 10^{-2}$	1.0000	1.0000
Kernel method	Quadratic, w/ normalization, $\lambda = 10^{-1}$	1.0000	1.0000
Kernel method	Quadratic, w/ normalization, $\lambda = 1$	1.0000	1.0000

TABLE S-5: Results of 5-fold CV with 5 different random seeds of the kernel method for the iris dataset (0 or 1). We set $\lambda = 10^{-2}, 10^{-1}, 1$. For $\phi(\cdot)$, we use linear and quadratic functions with and without normalization. We use the squared error function.

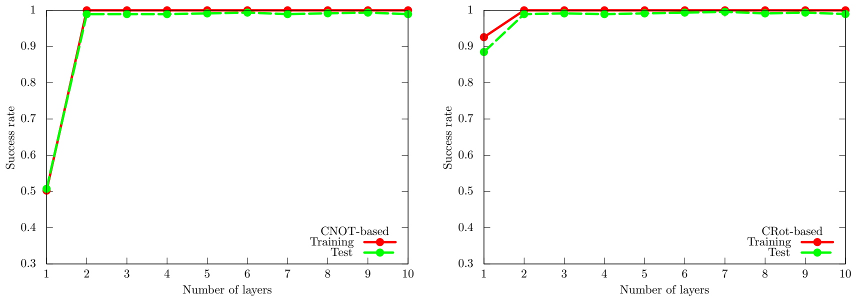

FIG. S-5: Performance dependence of QCL on the number of layers L for the iris dataset (0 or 1). We use the CNOT-based and CRot-based circuits. We set $\theta_{\text{bias}} = 0$. We iterate the computation 300 times.

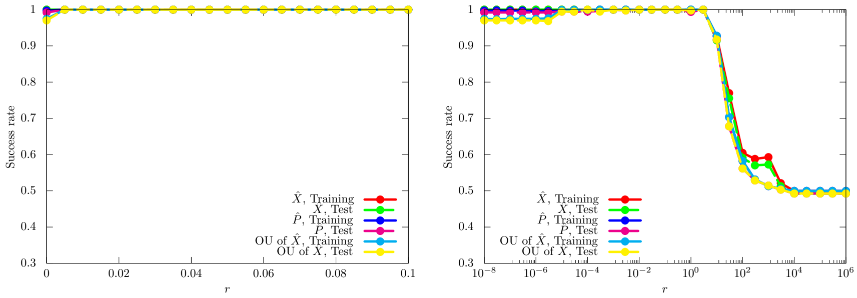

FIG. S-6: Performance dependence of the UKM on r, which is the coefficient of the second term in the right-hand side of Eq. (S-IX.1.8) for the iris dataset (0 or 1). We show the performance obtained by $\hat{X}$, $\hat{P}$, and OU of $\hat{X}$. Note that $\hat{P}$, and OU of $\hat{X}$ strictly satisfy the unitarity constraint while $\hat{X}$ does not. We use complex matrices for the initial input and set $\theta_{\text{bias}} = 0$. We set $K = 30$ and $K' = 10$.

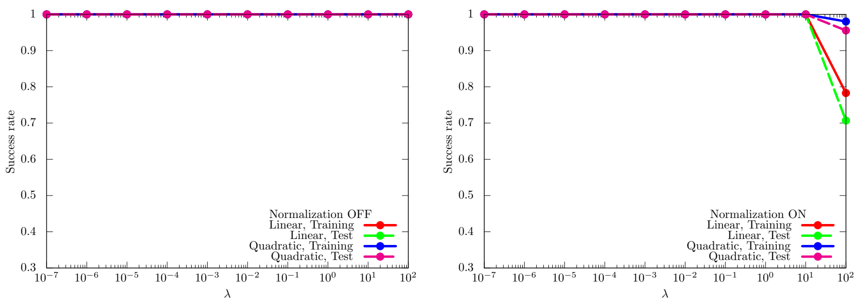

FIG. S-7: Performance dependence of the kernel method on λ, which is the coefficient of the second term in the right-hand side of Eq. (S-VI.2.4) for the iris dataset (0 or 1). For $\phi(\cdot)$ in Eq. (S-VI.2.1), we use linear and quadratic functions with and without normalization.

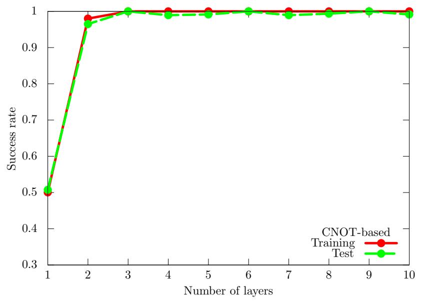

FIG. S-8: Performance dependence of QCL on the number of layers L for the iris dataset (0 or 1) in the case of the hinge function $\ell_{\text{hinge}}(\cdot,\cdot)$, Eq. (S-V.4.3). We use the CNOT-based circuit. We set $\theta_{\text{bias}} = 0$. We iterate the computation 300 times.

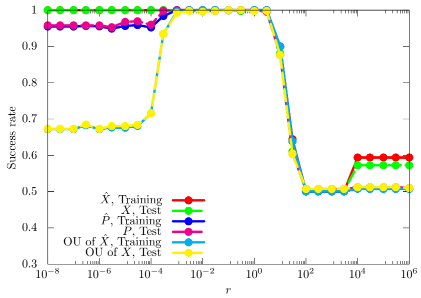

FIG. S-9: Performance dependence of the UKM on r, which is the coefficient of the second term in the right-hand side of Eq. (S-IX.1.8) for the iris dataset (0 or 1) in the case of the hinge function $\ell_{\text{hinge}}(\cdot,\cdot)$, Eq. (S-V.4.3). We show the performance obtained by $\hat{X}$, $\hat{P}$, and OU of $\hat{X}$. Note that $\hat{P}$, and OU of $\hat{X}$ strictly satisfy the unitarity constraint while $\hat{X}$ does not. We use complex matrices for the initial input and set $\theta_{\text{bias}} = 0$. We set $K = 30$ and $K' = 10$.

E. Iris dataset (0 or non-0)

We here show the numerical result for the iris dataset (0 or non-0). For the UKM, we put $r = 0.010$ and set $K = 30$ and $K' = 10$ in Algo. S-10. For QCL, we run iterations 300 times. We use the squared error function $\ell_{\mathrm{SE}}(\cdot,\cdot)$, Eq. (S-V.4.2).

In Fig. S-10, we show the numerical results of QCL for the 5-fold datasets with 5 different random seeds. In

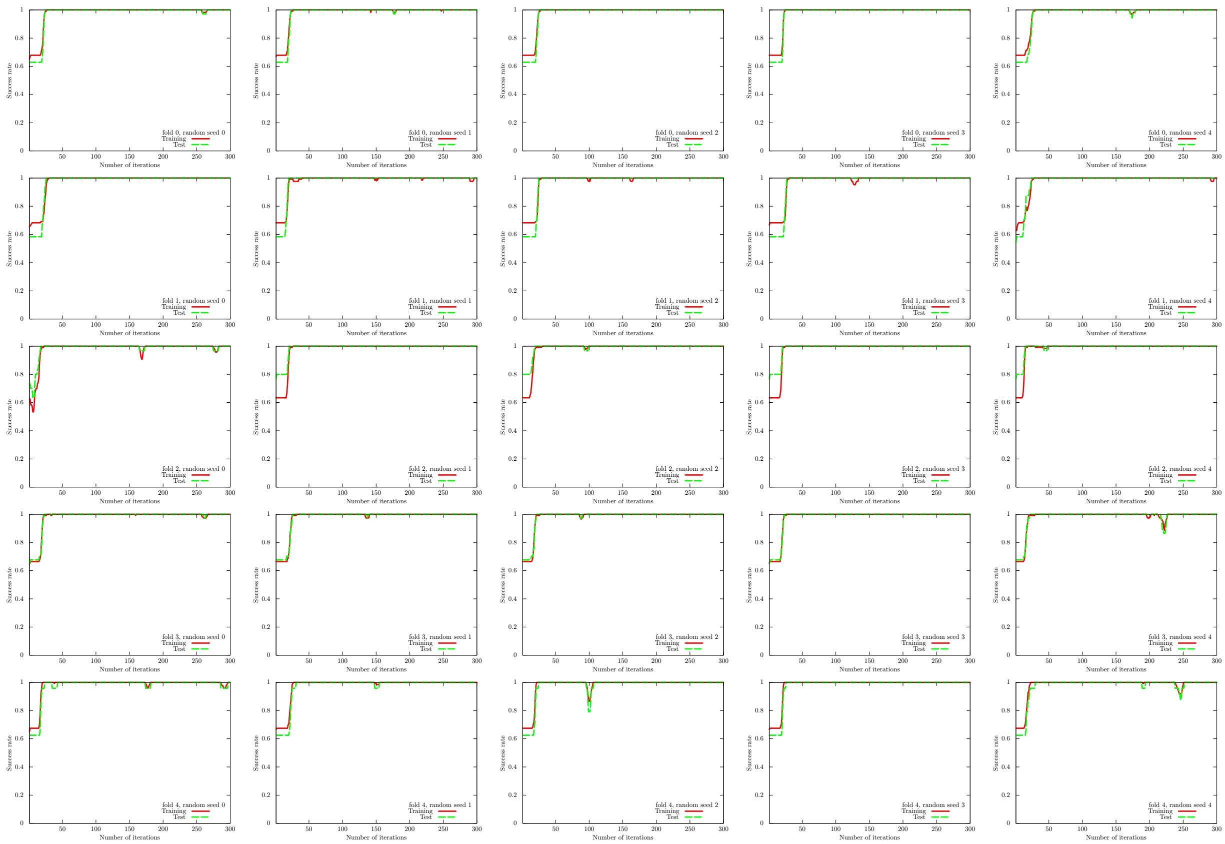

FIG. S-10: Results of QCL on the 5-fold datasets with 5 different random seeds for the iris dataset (0 or non-0). We use the CNOT-based circuit and set $\theta_{\mathrm{bias}} = 0$. The number of layers L is set to 5.

Fig. S-11, we show the numerical results of $\hat{P}$ of the UKM for the 5-fold datasets with 5 different random seeds. In Fig. S-12, we also show the numerical results of OU of $\hat{X}$ of the UKM for the 5-fold datasets with 5 different random seeds.

We summarize the results of 5-fold CV with 5 different random seeds of QCL and the UKM in Tables S-6 and S-7. For QCL and the UKM, we select the best model for the training dataset over iterations to compute the performance.

Algo.	Condition	Training	Test
QCL	CNOT-based, w/o bias	1.0	1.0
QCL	CNOT-based, w/ bias	1.0	1.0
QCL	CRot-based, w/o bias	1.0	1.0
QCL	CRot-based, w/ bias	1.0	1.0
QCL	1d Heisenberg, w/o bias	1.0	0.9957
QCL	1d Heisenberg, w/ bias	1.0	0.9920
QCL	FC Heisenberg, w/o bias	1.0	0.9957
QCL	FC Heisenberg, w/ bias	1.0	0.9920

TABLE S-6: Results of 5-fold CV with 5 different random seeds of QCL for the iris dataset (0 or non-0). We consider four types of circuits with and without the bias term: the CNOT-based circuit, the CRot-based circuit, 1d Heisenberg circuit, and the FC Heisenberg circuit. The number of layers L is set to 5 and the number of iterations is set to 300.

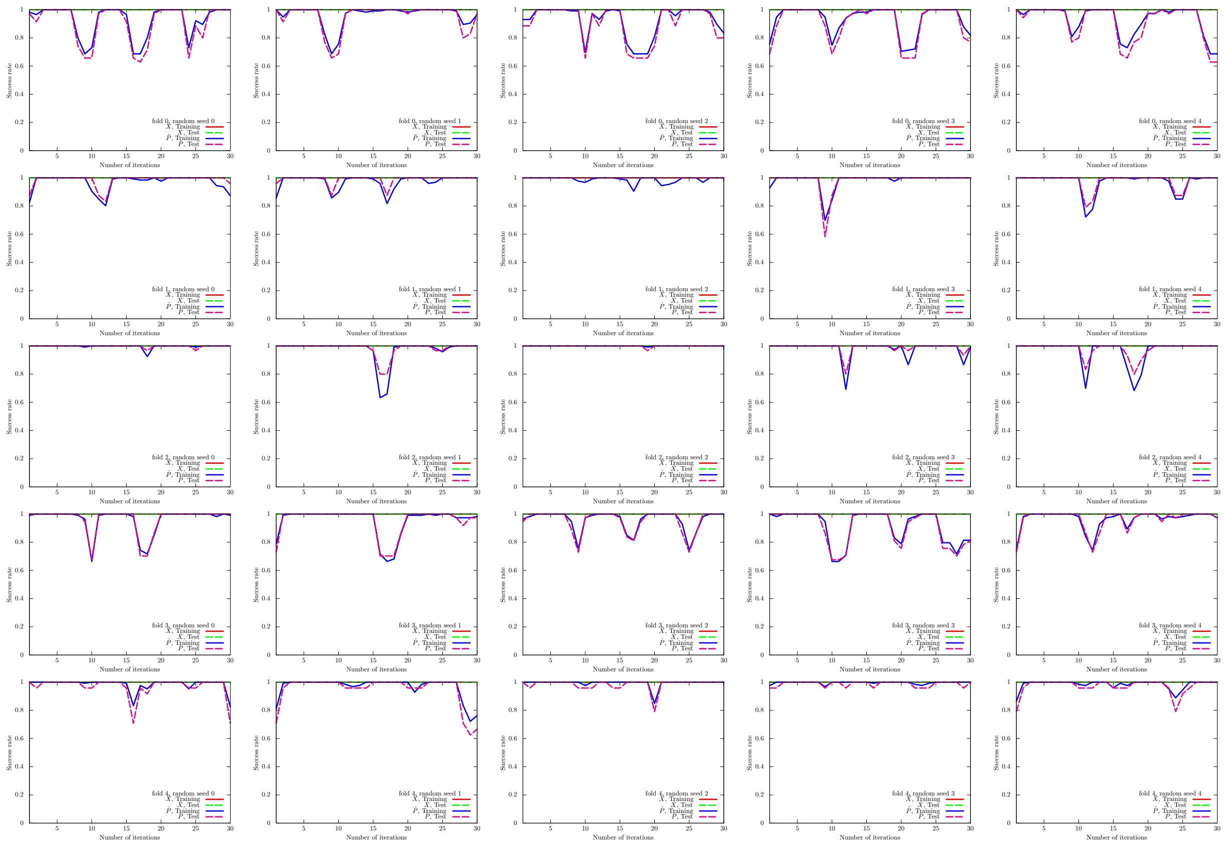

FIG. S-11: Results of the UKM ($\hat{X}$ and $\hat{P}$) on the 5-fold datasets with 5 different random seeds for the iris dataset (0 or non-0). We use complex matrices for the initial input and set $\theta_{\text{bias}} = 0$. We set $r = 0.010$.

Algo.	Condition	Training	Test
UKM	$\hat{X}$, complex, w/o bias	1.0	1.0
UKM	$\hat{P}$, complex, w/o bias	1.0	0.9917
UKM	OU of $\hat{X}$, complex, w/o bias	1.0	1.0
UKM	$\hat{X}$, complex, w/ bias	1.0	1.0
UKM	$\hat{P}$, complex, w/ bias	1.0	0.9987
UKM	OU of $\hat{X}$, complex, w/ bias	1.0	0.9987
UKM	$\hat{X}$, real, w/o bias	1.0	1.0
UKM	$\hat{P}$, real, w/o bias	1.0	0.9917
UKM	OU of $\hat{X}$, real, w/o bias	1.0	1.0
UKM	$\hat{X}$, real, w/ bias	1.0	1.0
UKM	$\hat{P}$, real, w/ bias	1.0	0.9970
UKM	OU of $\hat{X}$, real, w/ bias	1.0	0.9970

TABLE S-7: Results of 5-fold CV with 5 different random seeds of the UKM for the iris dataset (0 or non-0). We show the performance obtained by $\hat{X}$, $\hat{P}$, and OU of $\hat{X}$. Note that $\hat{P}$, and OU of $\hat{X}$ strictly satisfy the unitarity constraint while $\hat{X}$ does not. We consider real and complex matrices for the initial input with and without the bias term. We put $r = 0.010$ and set $K = 30$ and $K' = 10$.

In Fig. S-13, we plot the data shown in Tables S-6 and S-7. We also summarize the results of 5-fold CV with 5 different random seeds of the kernel method in Table S-8. More specifically, we use Ridge classification, which is described in Sec. S-VI B. We consider linear and quadratic functions for $\phi(\cdot)$ in Eq. (S-VI.2.1) with and without normalization. We set $\lambda = 10^{-2}, 10^{-1}, 1$ where λ is the coefficient of the regularization term.

Next, we show the performance dependence of the three algorithms on their key parameters. We see the performance dependence of QCL on the number of layers L. The result is shown in Fig. S-14. We then see the performance

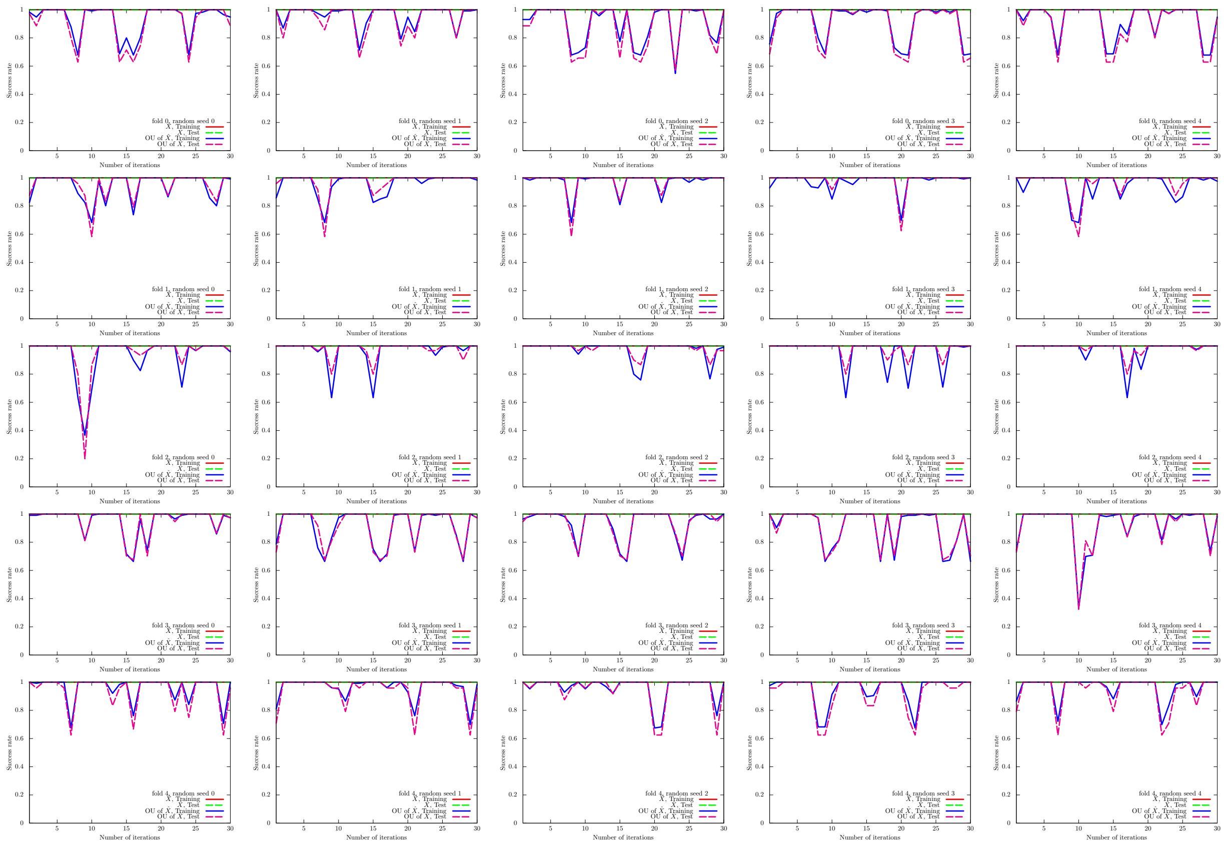

FIG. S-12: Results of the UKM ($\hat{X}$ and OU of $\hat{X}$) on the 5-fold datasets with 5 different random seeds for the iris dataset (0 or non-0). We use complex matrices for the initial input and set $\theta_{\text{bias}} = 0$. We set $r = 0.010$.

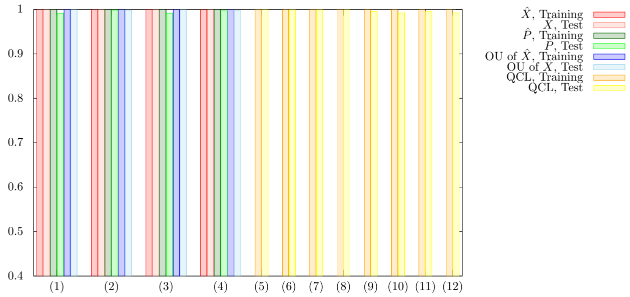

FIG. S-13: Results of 5-fold CV with 5 different random seeds for the iris dataset (0 or non-0). For the UKM, we put $r = 0.010$ and set $K = 30$ and $K' = 10$. For QCL, the number of layers L is 5 and the number of iterations is 300. The numerical settings are as follows: (1) UKM: complex matrices without the bias term, (2) UKM: complex matrices with the bias term, (3) UKM: real matrices without the bias term, (4) UKM: real matrices with the bias term, (5) QCL: CNOT-based circuit without the bias term, (6) QCL: CNOT-based circuit with the bias term, (7) QCL: CRot-based circuit without the bias term, (8) QCL: CRot-based circuit with the bias term, (9) QCL: 1d Heisenberg circuit without the bias term, (10) QCL: 1d Heisenberg circuit with the bias term, (11) QCL: FC Heisenberg circuit without the bias term, and (12) QCL: FC Heisenberg circuit with the bias term.

dependence of the UKM on r, which is the coefficient of the second term in the right-hand side of Eq. (S-IX.1.8). The result is shown in Fig. S-15. In Fig. S-16, we show the performance dependence of the kernel method on λ, which is the coefficient of the second term in the right-hand side of Eq. (S-VI.2.4).

So far, we have used the squared error function $\ell_{\text{SE}}(\cdot, \cdot)$, Eq. (S-V.4.2). In Fig. S-17, we show the performance dependence of QCL on the number of layers L in the case of the hinge function $\ell_{\text{hinge}}(\cdot, \cdot)$, Eq. (S-V.4.3). In Fig. S-18, we show the performance dependence of the UKM on r, which is the coefficient of the second term in the right-hand side of Eq. (S-IX.1.8), in the case of the hinge function $\ell_{\text{hinge}}(\cdot, \cdot)$, Eq. (S-V.4.3).

Algo.	Condition	Training	Test
Kernel method	Linear, w/o normalization, $\lambda = 10^{-2}$	1.0000	1.0000
Kernel method	Linear, w/o normalization, $\lambda = 10^{-1}$	1.0000	1.0000
Kernel method	Linear, w/o normalization, $\lambda = 1$	1.0000	1.0000
Kernel method	Linear, w/ normalization, $\lambda = 10^{-2}$	1.0000	1.0000
Kernel method	Linear, w/ normalization, $\lambda = 10^{-1}$	1.0000	1.0000
Kernel method	Linear, w/ normalization, $\lambda = 1$	1.0000	1.0000
Kernel method	Quadratic, w/o normalization, $\lambda = 10^{-2}$	1.0000	1.0000
Kernel method	Quadratic, w/o normalization, $\lambda = 10^{-1}$	1.0000	1.0000
Kernel method	Quadratic, w/o normalization, $\lambda = 1$	1.0000	1.0000
Kernel method	Quadratic, w/ normalization, $\lambda = 10^{-2}$	1.0000	1.0000
Kernel method	Quadratic, w/ normalization, $\lambda = 10^{-1}$	1.0000	1.0000
Kernel method	Quadratic, w/ normalization, $\lambda = 1$	1.0000	1.0000

TABLE S-8: Results of 5-fold CV with 5 different random seeds of the kernel method for the iris dataset (0 or non-0). We set $\lambda = 10^{-2}, 10^{-1}, 1$. For $\phi(\cdot)$, we use linear and quadratic functions with and without normalization. We use the squared error function.

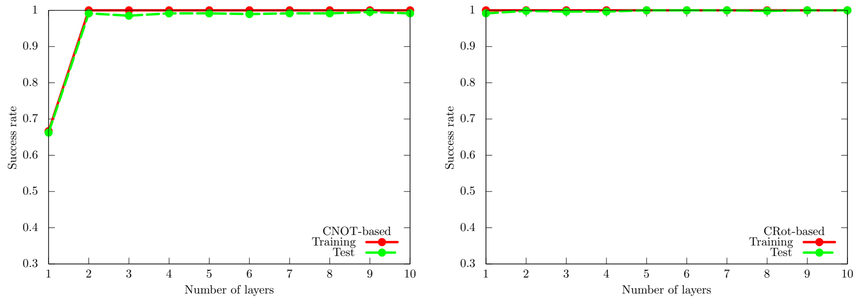

FIG. S-14: Performance dependence of QCL on the number of layers L for the iris dataset (0 or non-0). We use the CNOT-based and CRot-based circuits. We set $\theta_{\rm bias} = 0$. We iterate the computation 300 times.

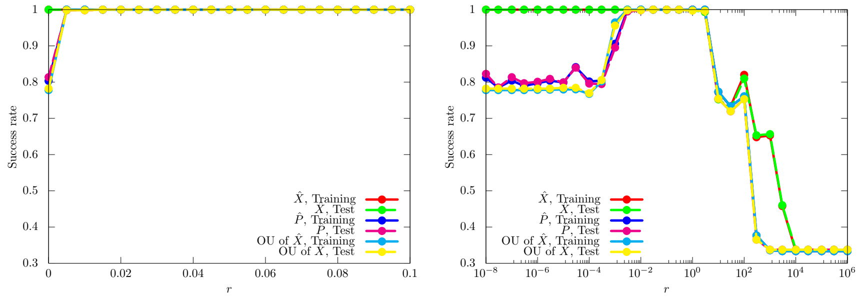

FIG. S-15: Performance dependence of the UKM on r, which is the coefficient of the second term in the right-hand side of Eq. (S-IX.1.8) for the iris dataset (0 or non-0). We show the performance obtained by $\hat{X}$, $\hat{P}$, and OU of $\hat{X}$. Note that $\hat{P}$, and OU of $\hat{X}$ strictly satisfy the unitarity constraint while $\hat{X}$ does not. We use complex matrices for the initial input and set $\theta_{\rm bias} = 0$. We set $K = 30$ and $K' = 10$.

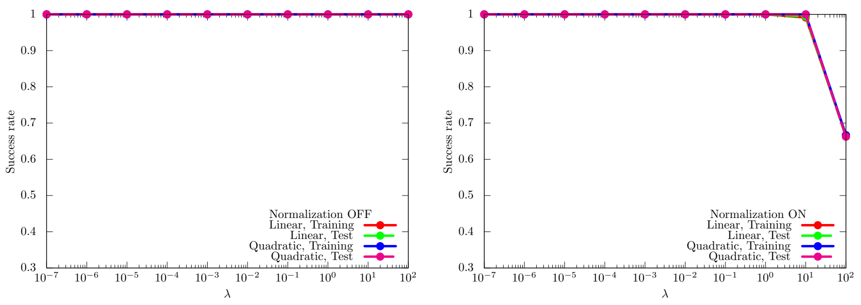

FIG. S-16: Performance dependence of the kernel method on λ, which is the coefficient of the second term in the right-hand side of Eq. (S-VI.2.4) for the iris dataset (0 or non-0). For $\phi(\cdot)$ in Eq. (S-VI.2.1), we use linear and quadratic functions with and without normalization.

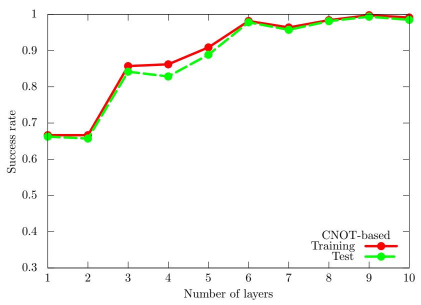

FIG. S-17: Performance dependence of QCL on the number of layers L for the iris dataset (0 or non-0) in the case of the hinge function $\ell_{\text{hinge}}(\cdot,\cdot)$, Eq. (S-V.4.3). We use the CNOT-based circuit. We set $\theta_{\text{bias}} = 0$. We iterate the computation 300 times.

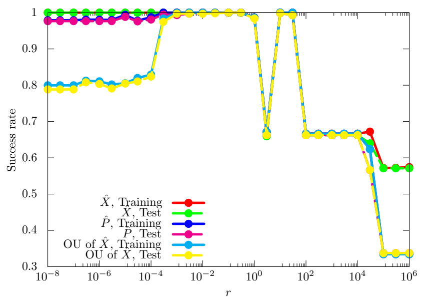

FIG. S-18: Performance dependence of the UKM on r, which is the coefficient of the second term in the right-hand side of Eq. (S-IX.1.8) for the iris dataset (0 or non-0) in the case of the hinge function $\ell_{\text{hinge}}(\cdot,\cdot)$, Eq. (S-V.4.3). We show the performance obtained by $\hat{X}$, $\hat{P}$, and OU of $\hat{X}$. Note that $\hat{P}$, and OU of $\hat{X}$ strictly satisfy the unitarity constraint while $\hat{X}$ does not. We use complex matrices for the initial input and set $\theta_{\text{bias}} = 0$. We set $K = 30$ and $K' = 10$.

F. Iris dataset (1 or non-1)

We here show the numerical result for the iris dataset (1 or non-1). For the UKM, we put $r = 0.010$ and set $K = 30$ and $K' = 10$ in Algo. S-10. For QCL, we run iterations 300 times. We use the squared error function $\ell_{\mathrm{SE}}(\cdot, \cdot)$, Eq. (S-V.4.2).

In Fig. S-19, we show the numerical results of QCL for the 5-fold datasets with 5 different random seeds. In Fig. S-20, we show the numerical results of $\hat{P}$ of the UKM for the 5-fold datasets with 5 different random seeds. In Fig. S-21, we also show the numerical results of OU of $\hat{X}$ of the UKM for the 5-fold datasets with 5 different random seeds.

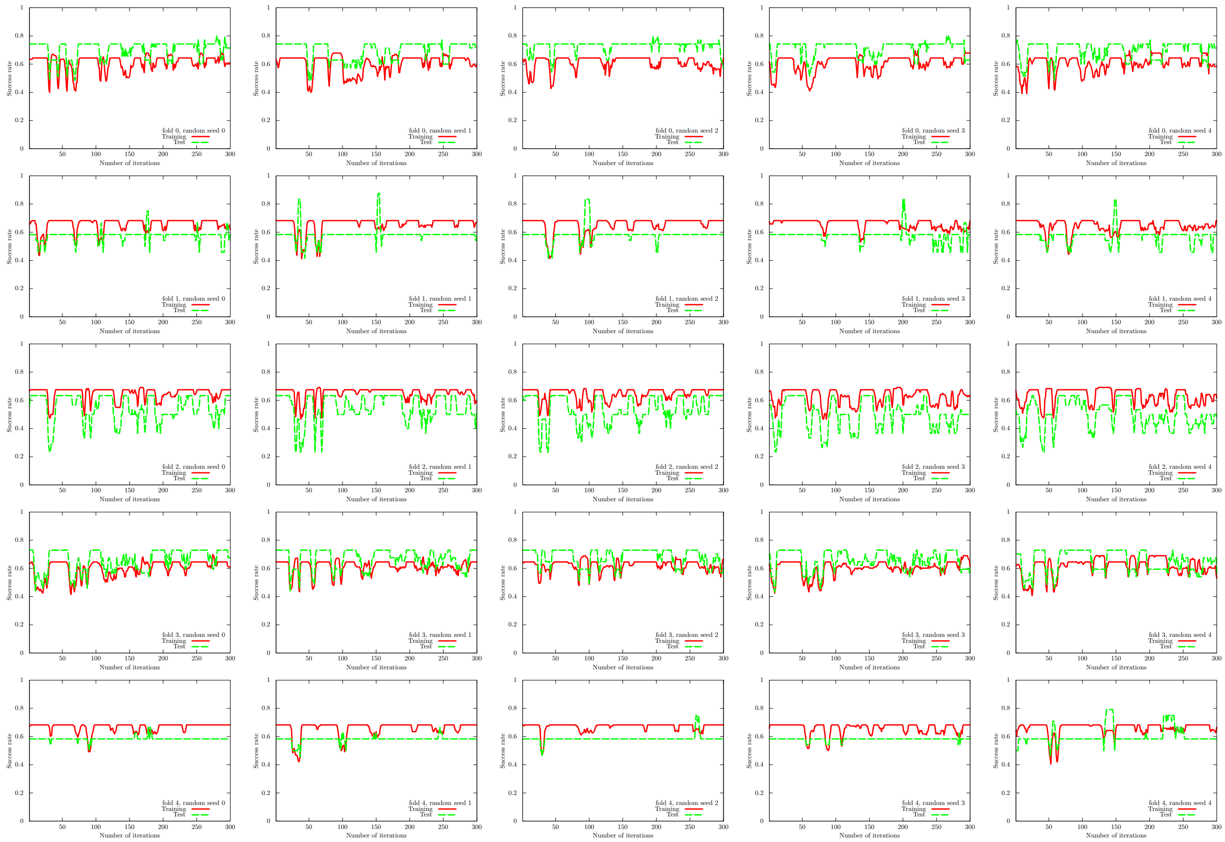

FIG. S-19: Results of QCL on the 5-fold datasets with 5 different random seeds for the iris dataset (1 or non-1). We use the CNOT-based circuit and set $\theta_{\mathrm{bias}} = 0$. The number of layers L is set to 5.

We summarize the results of 5-fold CV with 5 different random seeds of QCL and the UKM in Tables S-9 and S-10, respectively. For QCL and the UKM, we select the best model for the training dataset over iterations to compute the performance. In Fig. S-22, we plot the data shown in Tables S-9 and S-10. We also summarize the results of 5-fold

Algo.	Condition	Training	Test
QCL	CNOT-based, w/o bias	0.6851	0.5845
QCL	CNOT-based, w/ bias	0.6919	0.5854
QCL	CRot-based, w/o bias	0.6840	0.5793
QCL	CRot-based, w/ bias	0.6847	0.5793
QCL	1d Heisenberg, w/o bias	0.6760	0.6004
QCL	1d Heisenberg, w/ bias	0.6801	0.5872
QCL	FC Heisenberg, w/o bias	0.6760	0.6004
QCL	FC Heisenberg, w/ bias	0.6801	0.5872

TABLE S-9: Results of 5-fold CV with 5 different random seeds of QCL for the iris dataset (1 or non-1). We consider four types of circuits with and without the bias term: the CNOT-based circuit, the CRot-based circuit, 1d Heisenberg circuit, and the FC Heisenberg circuit. The number of layers L is set to 5 and the number of iterations is set to 300.

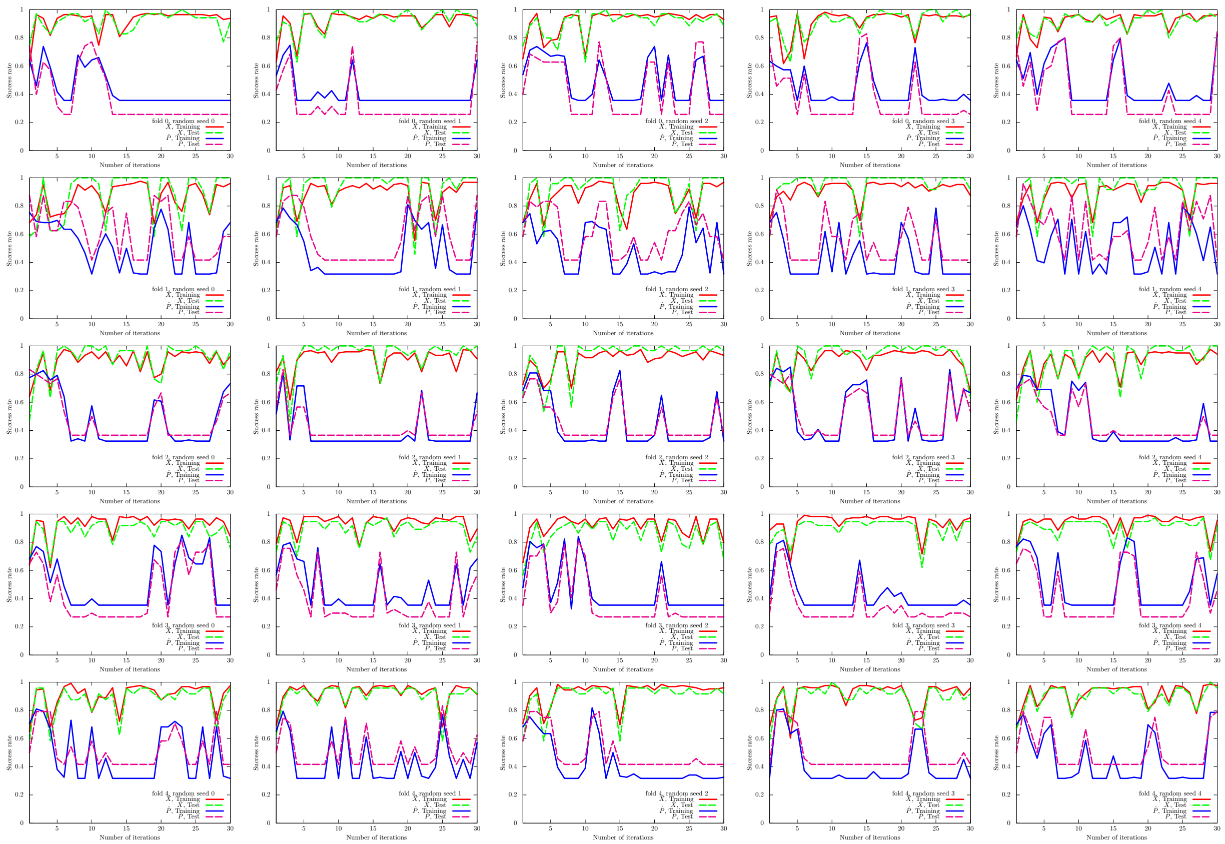

FIG. S-20: Results of the UKM ($\hat{X}$ and $\hat{P}$) on the 5-fold datasets with 5 different random seeds for the iris dataset (1 or non-1). We use complex matrices for the initial input and set $\theta_{\text{bias}} = 0$. We set $r = 0.010$.

Algo.	Condition	Training	Test
UKM	$\hat{X}$, complex, w/o bias	0.9781	0.9618
UKM	$\hat{P}$, complex, w/o bias	0.7873	0.7781
UKM	OU of $\hat{X}$, complex, w/o bias	0.7953	0.7994
UKM	$\hat{X}$, complex, w/ bias	0.9712	0.9581
UKM	$\hat{P}$, complex, w/ bias	0.6744	0.6564
UKM	OU of $\hat{X}$, complex, w/ bias	0.6734	0.6507
UKM	$\hat{X}$, real, w/o bias	0.9778	0.9717
UKM	$\hat{P}$, real, w/o bias	0.7880	0.7789
UKM	OU of $\hat{X}$, real, w/o bias	0.7869	0.7713
UKM	$\hat{X}$, real, w/ bias	0.9702	0.9569
UKM	$\hat{P}$, real, w/ bias	0.6746	0.6568
UKM	OU of $\hat{X}$, real, w/ bias	0.6794	0.6640

TABLE S-10: Results of 5-fold CV with 5 different random seeds of the UKM for the iris dataset (1 or non-1). We show the performance obtained by $\hat{X}$, $\hat{P}$, and OU of $\hat{X}$. Note that $\hat{P}$, and OU of $\hat{X}$ strictly satisfy the unitarity constraint while $\hat{X}$ does not. We consider real and complex matrices for the initial input with and without the bias term. We put $r = 0.010$ and set $K = 30$ and $K' = 10$.

CV with 5 different random seeds of the kernel method in Table S-11. More specifically, we use Ridge classification, which is described in Sec. S-VI B. We consider linear and quadratic functions for $\phi(\cdot)$ in Eq. (S-VI.2.1) with and without normalization. We set $\lambda = 10^{-2}, 10^{-1}, 1$ where λ is the coefficient of the regularization term.

Next, we show the performance dependence of the three algorithms on their key parameters. We see the performance dependence of QCL on the number of layers L. The result is shown in Fig. S-23. We then see the performance dependence of the UKM on r, which is the coefficient of the second term in the right-hand side of Eq. (S-IX.1.8). The

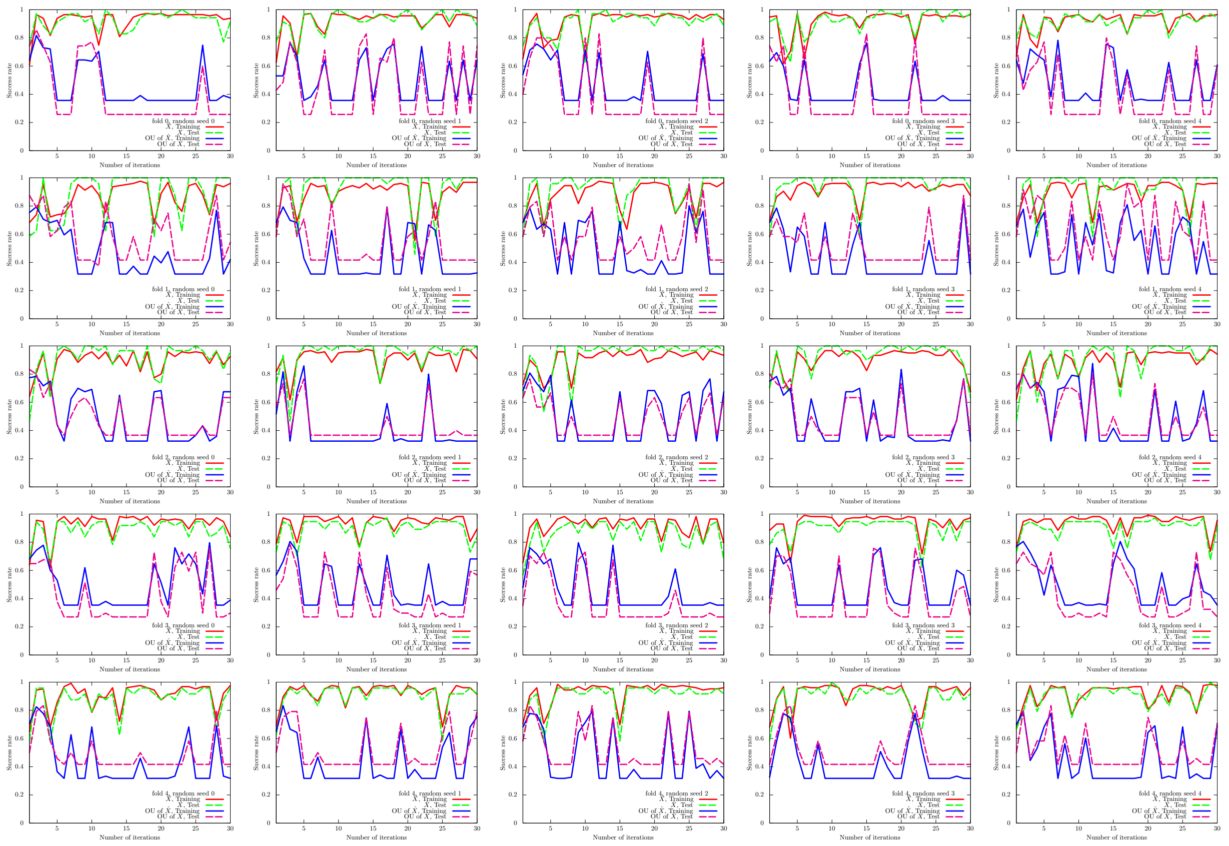

FIG. S-21: Results of the UKM ($\hat{X}$ and OU of $\hat{X}$) on the 5-fold datasets with 5 different random seeds for the iris dataset (1 or non-1). We use complex matrices for the initial input and set $\theta_{\text{bias}} = 0$. We set $r = 0.010$.

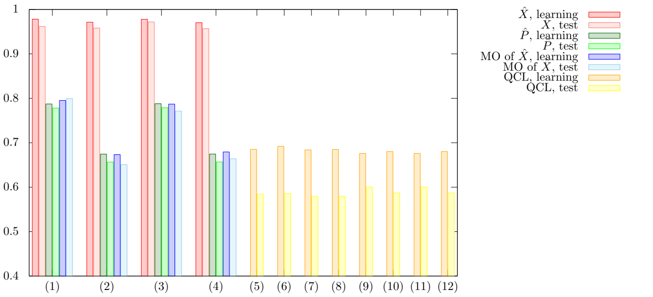

FIG. S-22: Results of 5-fold CV with 5 different random seeds for the iris dataset (1 or non-1). For the UKM, we put $r = 0.010$ and set $K = 30$ and $K' = 10$. For QCL, the number of layers L is 5 and the number of iterations is 300. The numerical settings are as follows: (1) UKM: complex matrices without the bias term, (2) UKM: complex matrices with the bias term, (3) UKM: real matrices without the bias term, (4) UKM: real matrices with the bias term, (5) QCL: CNOT-based circuit without the bias term, (6) QCL: CNOT-based circuit with the bias term, (7) QCL: CRot-based circuit without the bias term, (8) QCL: CRot-based circuit with the bias term, (9) QCL: 1d Heisenberg circuit without the bias term, (10) QCL: 1d Heisenberg circuit with the bias term, (11) QCL: FC Heisenberg circuit without the bias term, and (12) QCL: FC Heisenberg circuit with the bias term.

result is shown in Fig. S-24. In Fig. S-25, we show the performance dependence of the kernel method on λ, which is the coefficient of the second term in the right-hand side of Eq. (S-VI.2.4).

So far, we have used the squared error function $\ell_{\text{SE}}(\cdot,\cdot)$, Eq. (S-V.4.2). In Fig. S-26, we show the performance dependence of QCL on the number of layers L in the case of the hinge function $\ell_{\text{hinge}}(\cdot,\cdot)$, Eq. (S-V.4.3). In Fig. S-27, we show the performance dependence of the UKM on r, which is the coefficient of the second term in the right-hand side of Eq. (S-IX.1.8), in the case of the hinge function $\ell_{\text{hinge}}(\cdot,\cdot)$, Eq. (S-V.4.3).

Algo.	Condition	Training	Test
Kernel method	Linear, w/o normalization, $\lambda = 10^{-2}$	0.7463	0.7378
Kernel method	Linear, w/o normalization, $\lambda = 10^{-1}$	0.7445	0.7324
Kernel method	Linear, w/o normalization, $\lambda = 1$	0.7426	0.7240
Kernel method	Linear, w/ normalization, $\lambda = 10^{-2}$	0.9401	0.9561
Kernel method	Linear, w/ normalization, $\lambda = 10^{-1}$	0.7697	0.7276
Kernel method	Linear, w/ normalization, $\lambda = 1$	0.6382	0.6113
Kernel method	Quadratic, w/o normalization, $\lambda = 10^{-2}$	0.9751	0.9666
Kernel method	Quadratic, w/o normalization, $\lambda = 10^{-1}$	0.9733	0.9558
Kernel method	Quadratic, w/o normalization, $\lambda = 1$	0.9684	0.9558
Kernel method	Quadratic, w/ normalization, $\lambda = 10^{-2}$	0.9601	0.9601
Kernel method	Quadratic, w/ normalization, $\lambda = 10^{-1}$	0.9253	0.9312
Kernel method	Quadratic, w/ normalization, $\lambda = 1$	0.6778	0.6475

TABLE S-11: Results of 5-fold CV with 5 different random seeds of the kernel method for the iris dataset (1 or non-1). We set $\lambda = 10^{-2}, 10^{-1}, 1$. For $\phi(\cdot)$, we use linear and quadratic functions with and without normalization. We use the squared error function.

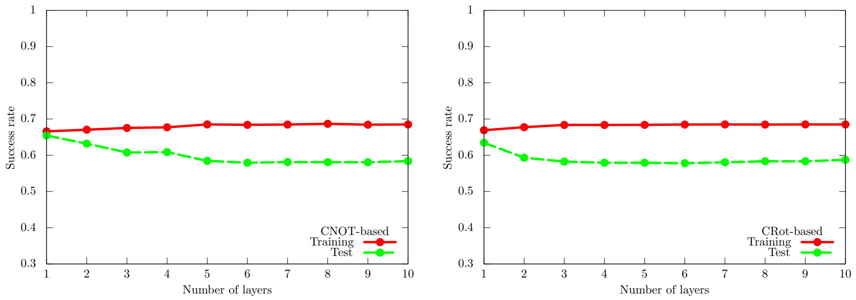

FIG. S-23: Performance dependence of QCL on the number of layers L for the iris dataset (1 or non-1). We use the CNOT-based and CRot-based circuits. We set $\theta_{\text{bias}} = 0$. We iterate the computation 300 times.

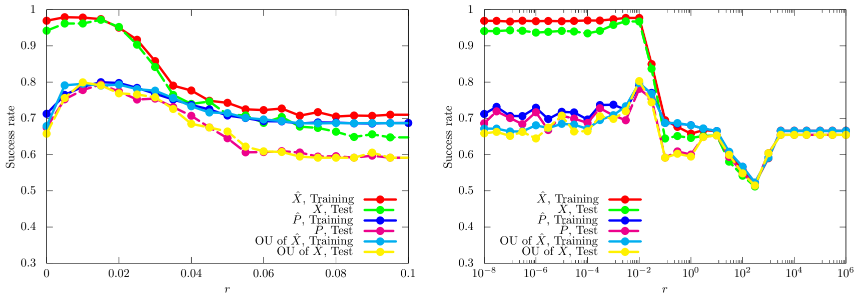

FIG. S-24: Performance dependence of the UKM on r, which is the coefficient of the second term in the right-hand side of Eq. (S-IX.1.8) for the iris dataset (1 or non-1). We show the performance obtained by $\hat{X}$, $\hat{P}$, and OU of $\hat{X}$. Note that $\hat{P}$, and OU of $\hat{X}$ strictly satisfy the unitarity constraint while $\hat{X}$ does not. We use complex matrices for the initial input and set $\theta_{\text{bias}} = 0$. We set $K = 30$ and $K' = 10$.

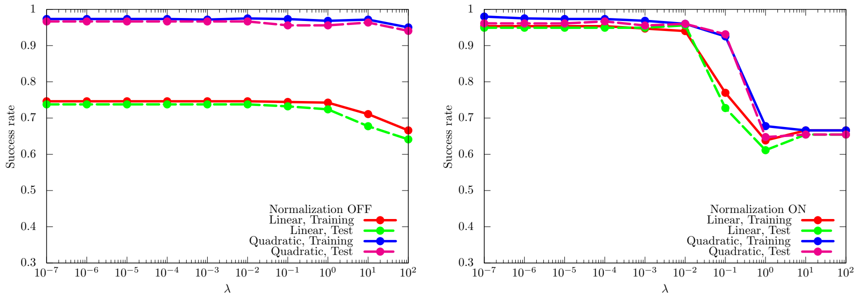

FIG. S-25: Performance dependence of the kernel method on λ, which is the coefficient of the second term in the right-hand side of Eq. (S-VI.2.4) for the iris dataset (1 or non-1). For $\phi(\cdot)$ in Eq. (S-VI.2.1), we use linear and quadratic functions with and without normalization.

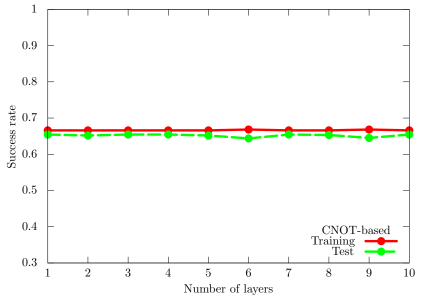

FIG. S-26: Performance dependence of QCL on the number of layers L for the iris dataset (1 or non-1) in the case of the hinge function $\ell_{\text{hinge}}(\cdot,\cdot)$, Eq. (S-V.4.3). We use the CNOT-based circuit. We set $\theta_{\text{bias}} = 0$. We iterate the computation 300 times.

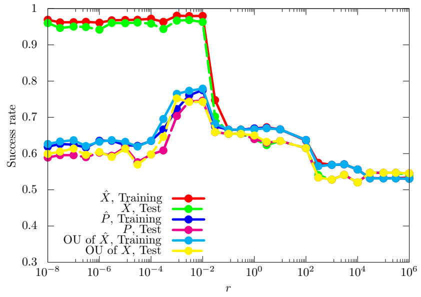

FIG. S-27: Performance dependence of the UKM on r, which is the coefficient of the second term in the right-hand side of Eq. (S-IX.1.8) for the iris dataset (1 or non-1) in the case of the hinge function $\ell_{\text{hinge}}(\cdot,\cdot)$, Eq. (S-V.4.3). We show the performance obtained by $\hat{X}$, $\hat{P}$, and OU of $\hat{X}$. Note that $\hat{P}$, and OU of $\hat{X}$ strictly satisfy the unitarity constraint while $\hat{X}$ does not. We use complex matrices for the initial input and set $\theta_{\text{bias}} = 0$. We set $K = 30$ and $K' = 10$.

G. Cancer dataset (0 or 1)

We here show the numerical result for the cancer dataset (0 or 1). For the UKM, we put $r = 0.010$ and set $K = 30$ and $K' = 10$ in Algo. S-10. For QCL, we run iterations 300 times. We use the squared error function $\ell_{\rm SE}(\cdot,\cdot)$, Eq. (S-V.4.2).

In Fig. S-28, we show the numerical results of QCL for the 5-fold datasets with 5 different random seeds. In

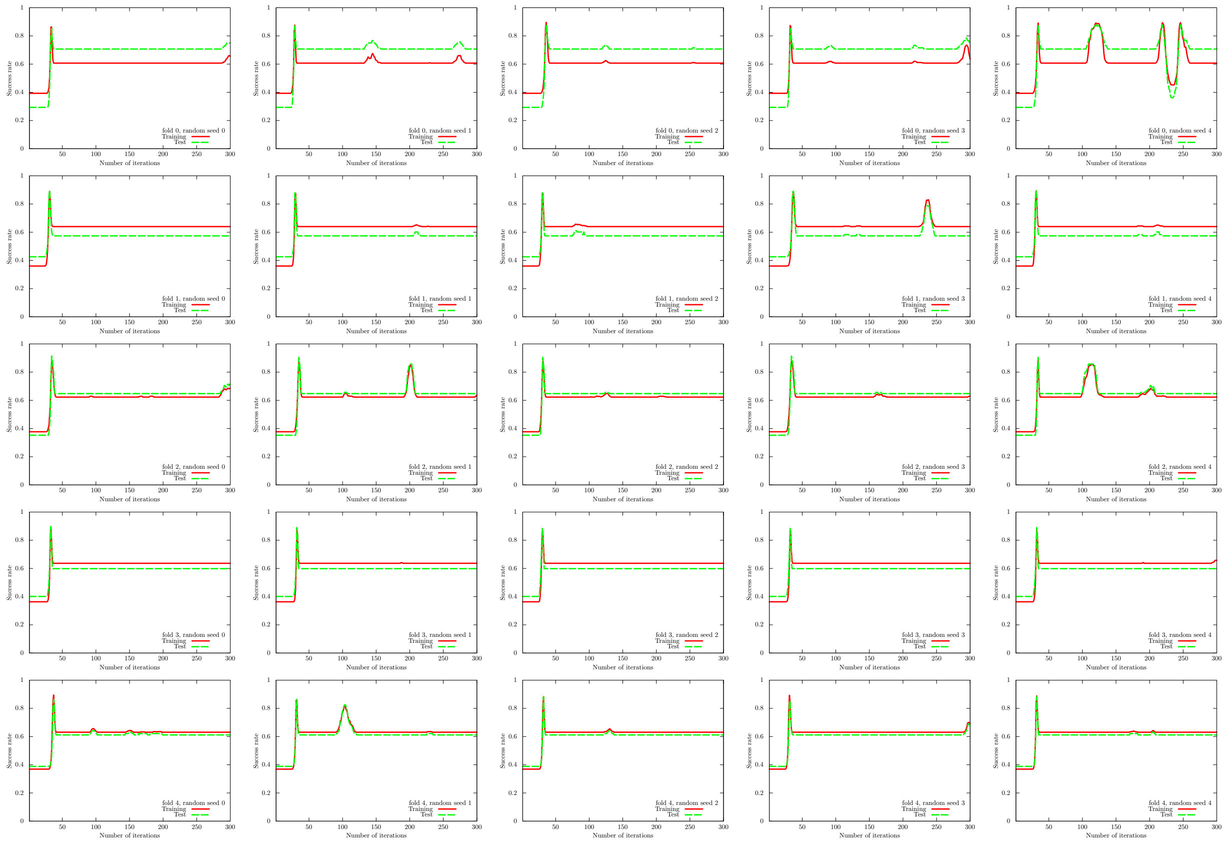

FIG. S-28: Results of QCL on the 5-fold datasets with 5 different random seeds for the cancer dataset (0 or 1). We use the CNOT-based circuit and set $\theta_{\rm bias} = 0$. The number of layers L is set to 5.

Fig. S-29, we show the numerical results of $\hat{P}$ of the UKM for the 5-fold datasets with 5 different random seeds. In Fig. S-30, we also show the numerical results of OU of $\hat{X}$ of the UKM for the 5-fold datasets with 5 different random seeds.

We summarize the results of 5-fold CV with 5 different random seeds of QCL and the UKM in Tables S-12 and S-13, respectively. For QCL and the UKM, we select the best model for the training dataset over iterations to compute the performance. In Fig. S-31, we plot the data shown in Tables S-12 and S-13. We also summarize the results of 5-fold

Algo.	Condition	Training	Test
QCL	CNOT-based, w/o bias	0.8797	0.8768
QCL	CNOT-based, w/ bias	0.8597	0.8577
QCL	CRot-based, w/o bias	0.7866	0.7752
QCL	CRot-based, w/ bias	0.8085	0.8052
QCL	1d Heisenberg, w/o bias	0.6568	0.6512
QCL	1d Heisenberg, w/ bias	0.7515	0.7427
QCL	FC Heisenberg, w/o bias	0.7435	0.7444
QCL	FC Heisenberg, w/ bias	0.7744	0.7789

TABLE S-12: Results of 5-fold CV with 5 different random seeds of QCL for the cancer dataset (0 or 1). We consider four types of circuits with and without the bias term: the CNOT-based circuit, the CRot-based circuit, 1d Heisenberg circuit, and the FC Heisenberg circuit. The number of layers L is set to 5 and the number of iterations is set to 300.

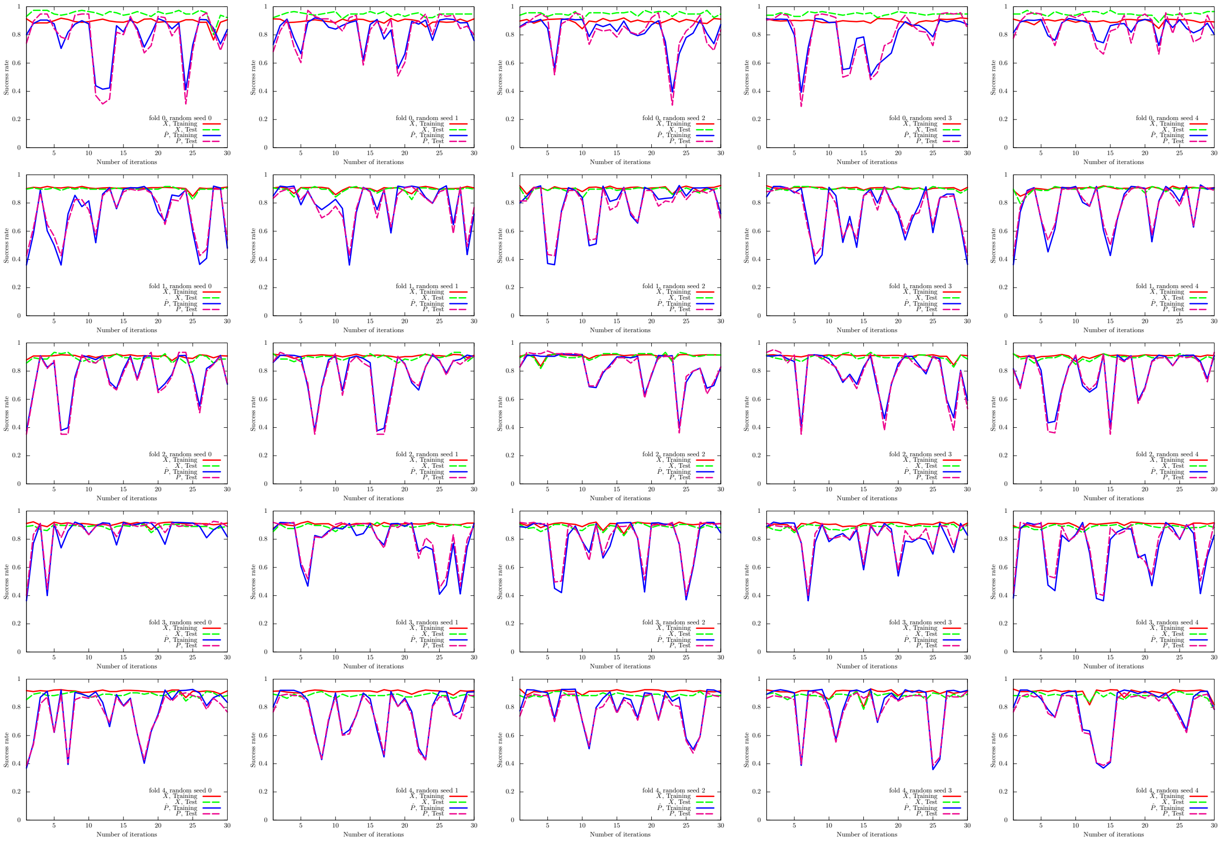

FIG. S-29: Results of the UKM ($\hat{X}$ and $\hat{P}$) on the 5-fold datasets with 5 different random seeds for the cancer dataset (0 or 1). We use complex matrices for the initial input and set $\theta_{\mathrm{bias}} = 0$. We set $r = 0.010$.

Algo.	Condition	Training	Test
UKM	$\hat{X}$, complex, w/o bias	0.9219	0.9143
UKM	$\hat{P}$, complex, w/o bias	0.9204	0.9093
UKM	OU of $\hat{X}$, complex, w/o bias	0.9184	0.9115
UKM	$\hat{X}$, complex, w/ bias	0.9207	0.9143
UKM	$\hat{P}$, complex, w/ bias	0.8870	0.8753
UKM	OU of $\hat{X}$, complex, w/ bias	0.8912	0.8805
UKM	$\hat{X}$, real, w/o bias	0.9213	0.9107
UKM	$\hat{P}$, real, w/o bias	0.9194	0.9131
UKM	OU of $\hat{X}$, real, w/o bias	0.9170	0.9112
UKM	$\hat{X}$, real, w/ bias	0.9218	0.9160
UKM	$\hat{P}$, real, w/ bias	0.7929	0.7879
UKM	OU of $\hat{X}$, real, w/ bias	0.8107	0.8014

TABLE S-13: Results of 5-fold CV with 5 different random seeds of the UKM for the cancer dataset (0 or 1). We show the performance obtained by $\hat{X}$, $\hat{P}$, and OU of $\hat{X}$. Note that $\hat{P}$, and OU of $\hat{X}$ strictly satisfy the unitarity constraint while $\hat{X}$ does not. We consider real and complex matrices for the initial input with and without the bias term. We put $r = 0.010$ and set $K = 30$ and $K' = 10$.

CV with 5 different random seeds of the kernel method in Table S-14. More specifically, we use Ridge classification, which is described in Sec. S-VI B. We consider linear and quadratic functions for $\phi(\cdot)$ in Eq. (S-VI.2.1) with and without normalization. We set $\lambda = 10^{-2}, 10^{-1}, 1$ where λ is the coefficient of the regularization term.

Next, we show the performance dependence of the three algorithms on their key parameters. We see the performance dependence of QCL on the number of layers L. The result is shown in Fig. S-32. We then ee the performance dependence of the UKM on r, which is the coefficient of the second term in the right-hand side of Eq. (S-IX.1.8). The

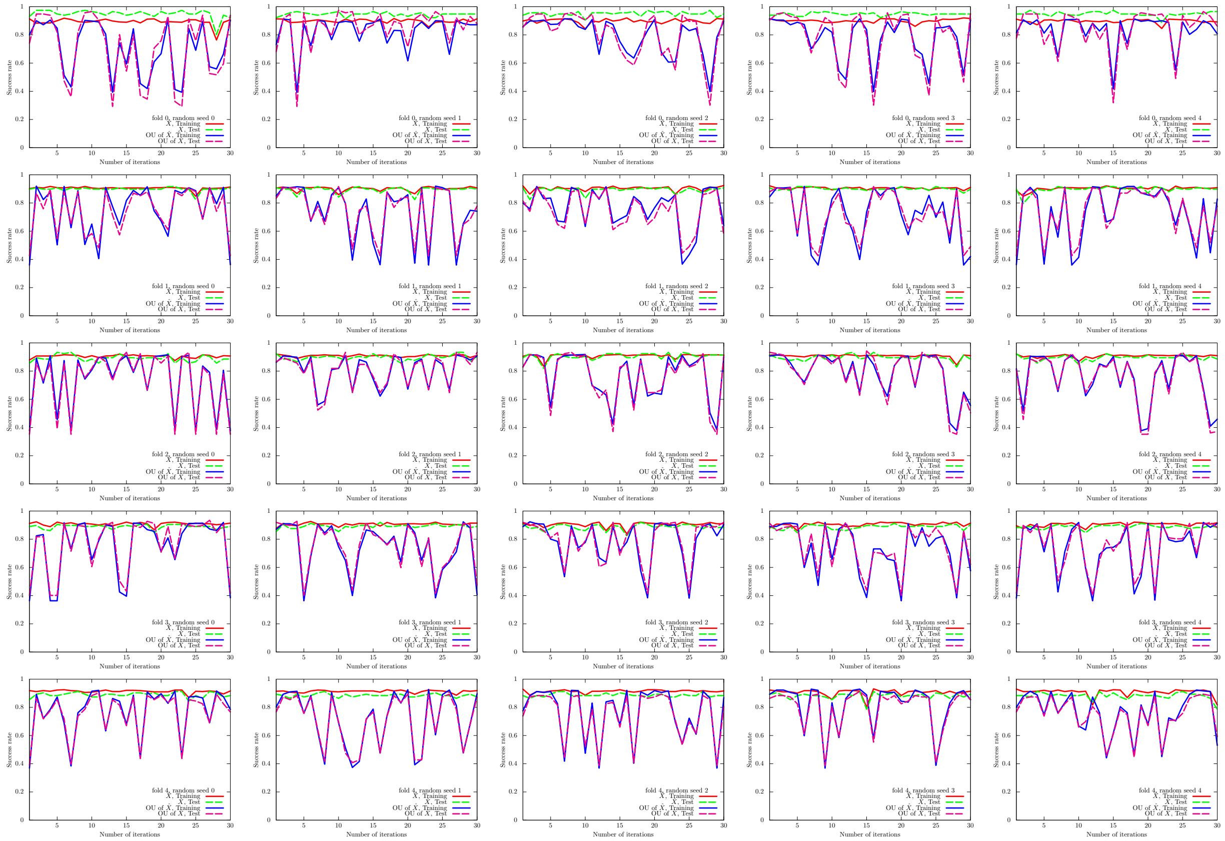

FIG. S-30: Results of the UKM ($\hat{X}$ and OU of $\hat{X}$) on the 5-fold datasets with 5 different random seeds for the cancer dataset (0 or 1). We use complex matrices for the initial input and set $\theta_{\mathrm{bias}} = 0$. We set $r = 0.010$.

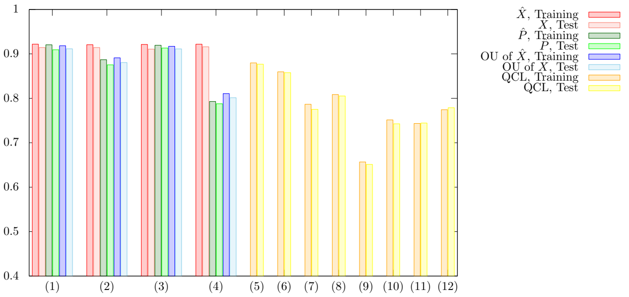

FIG. S-31: Results of 5-fold CV with 5 different random seeds for the cancer dataset (0 or 1). For the UKM, we put $r = 0.010$ and set $K = 30$ and $K' = 10$. For QCL, the number of layers L is 5 and the number of iterations is 300. The numerical settings are as follows: (1) UKM: complex matrices without the bias term, (2) UKM: complex matrices with the bias term, (3) UKM: real matrices without the bias term, (4) UKM: real matrices with the bias term, (5) QCL: CNOT-based circuit without the bias term, (6) QCL: CNOT-based circuit with the bias term, (7) QCL: CRot-based circuit without the bias term, (8) QCL: CRot-based circuit with the bias term, (9) QCL: 1d Heisenberg circuit without the bias term, (10) QCL: 1d Heisenberg circuit with the bias term, (11) QCL: FC Heisenberg circuit without the bias term, and (12) QCL: FC Heisenberg circuit with the bias term.

result is shown in Fig. S-33. In Fig. S-34, we show the performance dependence of the kernel method on λ, which is the coefficient of the second term in the right-hand side of Eq. (S-VI.2.4).

So far, we have used the squared error function $\ell_{\mathrm{SE}}(\cdot,\cdot)$, Eq. (S-V.4.2). In Fig. S-35, we show the performance dependence of QCL on the number of layers L in the case of the hinge function $\ell_{\mathrm{hinge}}(\cdot,\cdot)$, Eq. (S-V.4.3). In Fig. S-36, we show the performance dependence of the UKM on r, which is the coefficient of the second term in the right-hand side of Eq. (S-IX.1.8), in the case of the hinge function $\ell_{\mathrm{hinge}}(\cdot,\cdot)$, Eq. (S-V.4.3).

Algo.	Condition	Training	Test
Kernel method	Linear, w/o normalization, $\lambda = 10^{-2}$	0.9618	0.9568
Kernel method	Linear, w/o normalization, $\lambda = 10^{-1}$	0.9623	0.9549
Kernel method	Linear, w/o normalization, $\lambda = 1$	0.9591	0.9495
Kernel method	Linear, w/ normalization, $\lambda = 10^{-2}$	0.9262	0.9247
Kernel method	Linear, w/ normalization, $\lambda = 10^{-1}$	0.9205	0.9176
Kernel method	Linear, w/ normalization, $\lambda = 1$	0.8830	0.8812
Kernel method	Quadratic, w/o normalization, $\lambda = 10^{-2}$	0.9242	0.8491
Kernel method	Quadratic, w/o normalization, $\lambda = 10^{-1}$	0.9936	0.9361
Kernel method	Quadratic, w/o normalization, $\lambda = 1$	0.9907	0.9454
Kernel method	Quadratic, w/ normalization, $\lambda = 10^{-2}$	0.9298	0.9264
Kernel method	Quadratic, w/ normalization, $\lambda = 10^{-1}$	0.9210	0.9195
Kernel method	Quadratic, w/ normalization, $\lambda = 1$	0.9038	0.9052

TABLE S-14: Results of 5-fold CV with 5 different random seeds of the kernel method for the cancer dataset (0 or 1). We set $\lambda = 10^{-2}, 10^{-1}, 1$. For $\phi(\cdot)$, we use linear and quadratic functions with and without normalization. We use the squared error function.

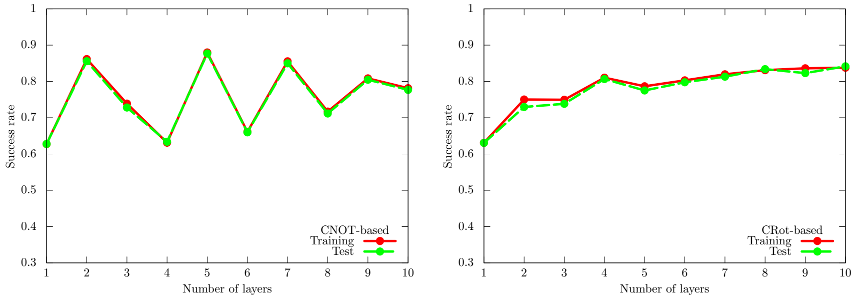

FIG. S-32: Performance dependence of QCL on the number of layers L for the cancer dataset (0 or 1). We use the CNOT-based and CRot-based circuits. We set $\theta_{\text{bias}} = 0$. We iterate the computation 300 times.

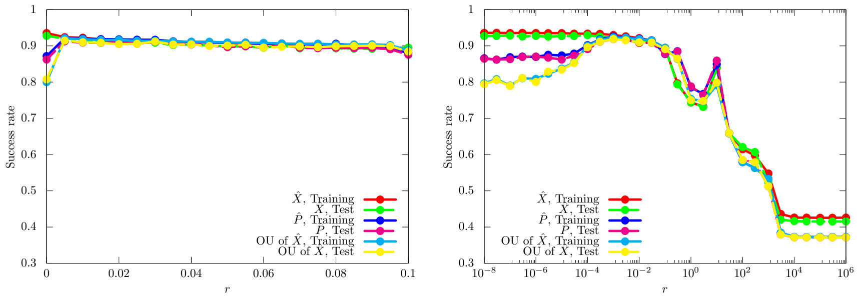

FIG. S-33: Performance dependence of the UKM on r, which is the coefficient of the second term in the right-hand side of Eq. (S-IX.1.8) for the cancer dataset (0 or 1). We show the performance obtained by $\hat{X}$, $\hat{P}$, and OU of $\hat{X}$. Note that $\hat{P}$, and OU of $\hat{X}$ strictly satisfy the unitarity constraint while $\hat{X}$ does not. We use complex matrices for the initial input and set $\theta_{\text{bias}} = 0$. We set $K = 30$ and $K' = 10$.

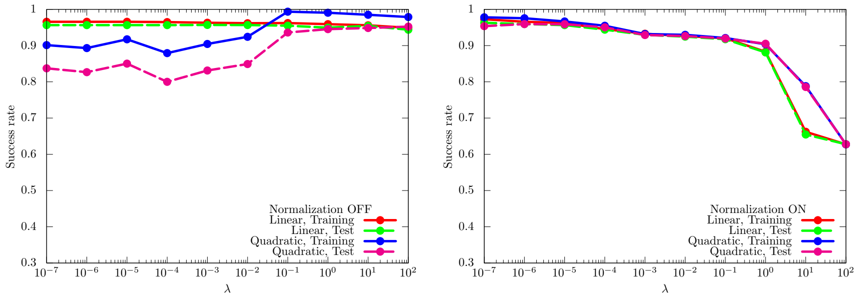

FIG. S-34: Performance dependence of the kernel method on λ, which is the coefficient of the second term in the right-hand side of Eq. (S-VI.2.4) for the cancer dataset (0 or 1). For $\phi(\cdot)$ in Eq. (S-VI.2.1), we use linear and quadratic functions with and without normalization.

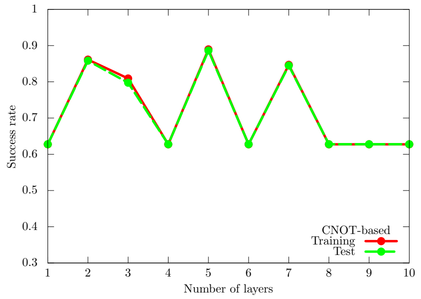

FIG. S-35: Performance dependence of QCL on the number of layers L for the cancer dataset (0 or 1) in the case of the hinge function $\ell_{\text{hinge}}(\cdot,\cdot)$, Eq. (S-V.4.3). We use the CNOT-based circuit. We set $\theta_{\text{bias}} = 0$. We iterate the computation 300 times.

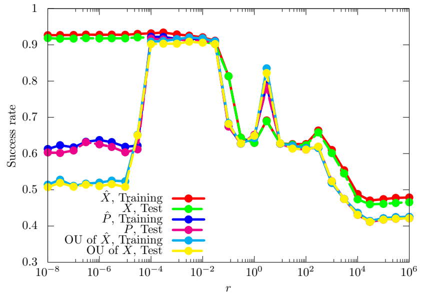

FIG. S-36: Performance dependence of the UKM on r, which is the coefficient of the second term in the right-hand side of Eq. (S-IX.1.8) for the cancer dataset (0 or 1) in the case of the hinge function $\ell_{\text{hinge}}(\cdot,\cdot)$, Eq. (S-V.4.3). We show the performance obtained by $\hat{X}$, $\hat{P}$, and OU of $\hat{X}$. Note that $\hat{P}$, and OU of $\hat{X}$ strictly satisfy the unitarity constraint while $\hat{X}$ does not. We use complex matrices for the initial input and set $\theta_{\text{bias}} = 0$. We set $K = 30$ and $K' = 10$.

H. Sonar dataset (0 or 1)

We here show the numerical result for the sonar dataset (0 or 1). For the UKM, we put $r = 0.010$ and set $K = 30$ and $K' = 10$ in Algo. S-10. For QCL, we run iterations 300 times. We use the squared error function $\ell_{\mathrm{SE}}(\cdot, \cdot)$, Eq. (S-V.4.2).

In Fig. S-37, we show the numerical results of QCL for the 5-fold datasets with 5 different random seeds. In

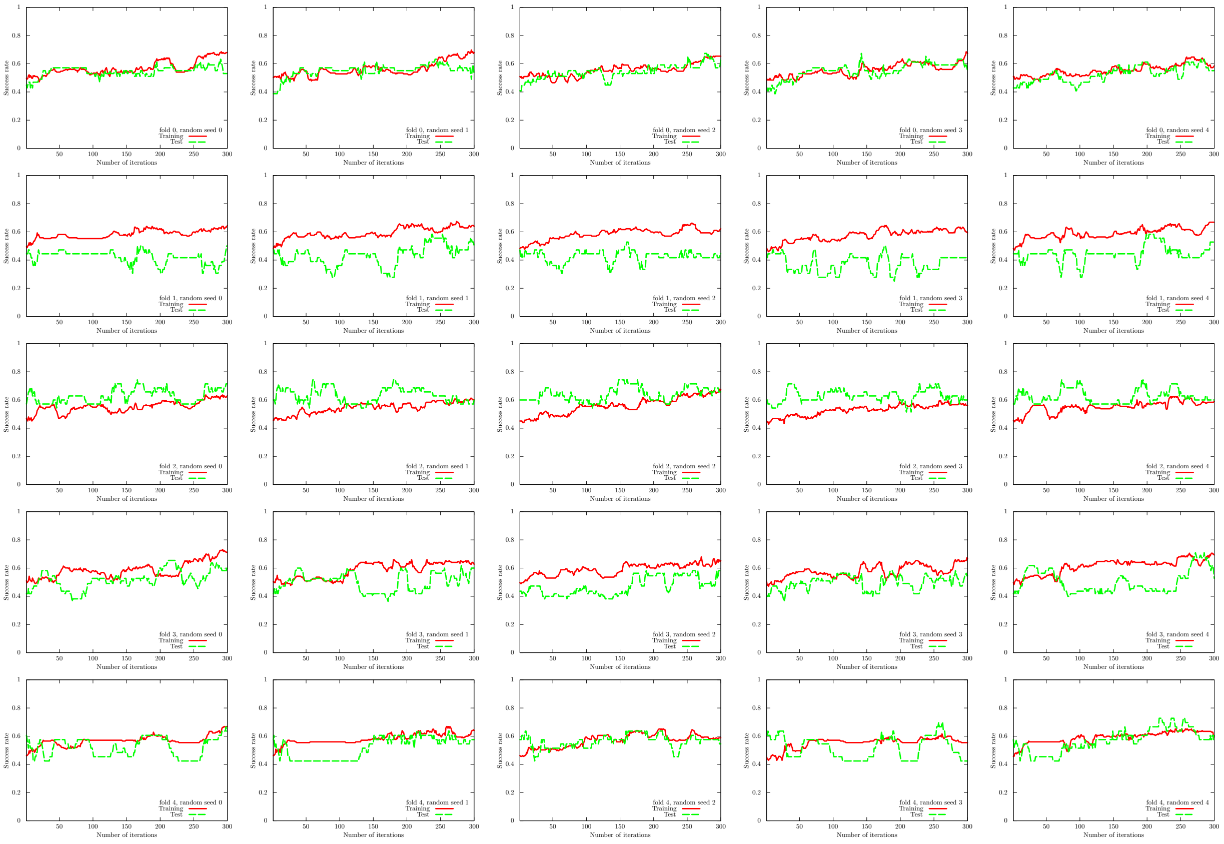

FIG. S-37: Results of QCL on the 5-fold datasets with 5 different random seeds for the sonar dataset (0 or 1). We use the CNOT-based circuit and set $\theta_{\mathrm{bias}} = 0$. The number of layers L is set to 5.

Fig. S-38, we show the numerical results of $\hat{P}$ of the UKM for the 5-fold datasets with 5 different random seeds. In Fig. S-39, we also show the numerical results of OU of $\hat{X}$ of the UKM for the 5-fold datasets with 5 different random seeds.

We summarize the results of 5-fold CV with 5 different random seeds of QCL and the UKM in Tables S-15 and S-16, respectively. For QCL and the UKM, we select the best model for the training dataset over iterations to compute the performance. In Fig. S-40, we plot the data shown in Tables S-15 and S-16. We also summarize the results of 5-fold

Algo.	Condition	Training	Test
QCL	CNOT-based, w/o bias	0.6606	0.5810
QCL	CNOT-based, w/ bias	0.6671	0.5739
QCL	CRot-based, w/o bias	0.6299	0.5703
QCL	CRot-based, w/ bias	0.7102	0.6468
QCL	1d Heisenberg, w/o bias	0.7320	0.6761
QCL	1d Heisenberg, w/ bias	0.7455	0.6924
QCL	FC Heisenberg, w/o bias	0.6653	0.6236
QCL	FC Heisenberg, w/ bias	0.6715	0.6285

TABLE S-15: Results of 5-fold CV with 5 different random seeds of QCL for the sonar dataset (0 or 1). We consider four types of circuits with and without the bias term: the CNOT-based circuit, the CRot-based circuit, 1d Heisenberg circuit, and the FC Heisenberg circuit. The number of layers L is set to 5 and the number of iterations is set to 300.

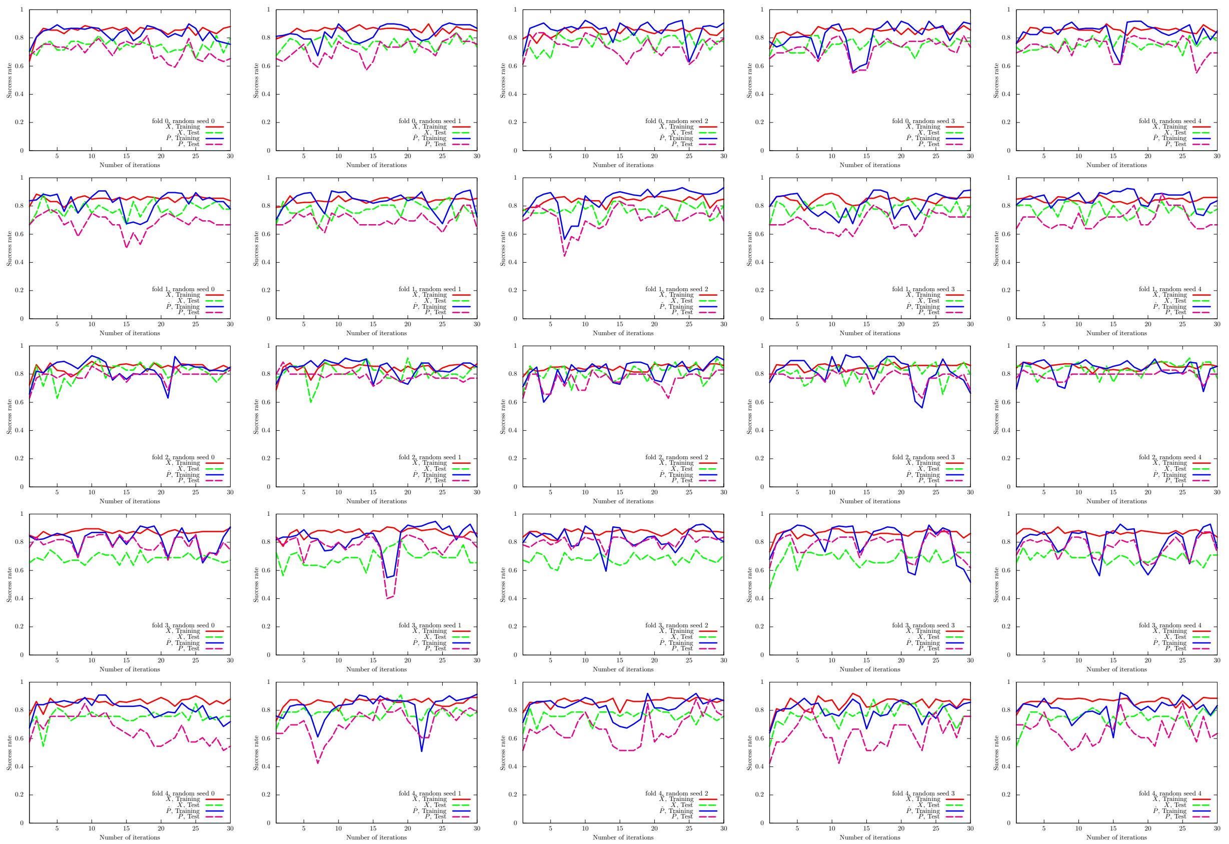

FIG. S-38: Results of the UKM ($\hat{X}$ and $\hat{P}$) on the 5-fold datasets with 5 different random seeds for the sonar dataset (0 or 1). We use complex matrices for the initial input and set $\theta_{\text{bias}} = 0$. We set $r = 0.010$.

Algo.	Condition	Training	Test
UKM	$\hat{X}$, complex, w/o bias	0.8903	0.7774
UKM	$\hat{P}$, complex, w/o bias	0.9159	0.7985
UKM	OU of $\hat{X}$, complex, w/o bias	0.9175	0.7909
UKM	$\hat{X}$, complex, w/ bias	0.9027	0.7723
UKM	$\hat{P}$, complex, w/ bias	0.6621	0.6036
UKM	OU of $\hat{X}$, complex, w/ bias	0.6351	0.5795
UKM	$\hat{X}$, real, w/o bias	0.8899	0.7630
UKM	$\hat{P}$, real, w/o bias	0.9141	0.7842
UKM	OU of $\hat{X}$, real, w/o bias	0.9108	0.7818
UKM	$\hat{X}$, real, w/ bias	0.9014	0.7772
UKM	$\hat{P}$, real, w/ bias	0.6913	0.6391
UKM	OU of $\hat{X}$, real, w/ bias	0.6769	0.6288

TABLE S-16: Results of 5-fold CV with 5 different random seeds of the UKM for the sonar dataset (0 or 1). We show the performance obtained by $\hat{X}$, $\hat{P}$, and OU of $\hat{X}$. Note that $\hat{P}$, and OU of $\hat{X}$ strictly satisfy the unitarity constraint while $\hat{X}$ does not. We consider real and complex matrices for the initial input with and without the bias term. We put $r = 0.010$ and set $K = 30$ and $K' = 10$.

CV with 5 different random seeds of the kernel method in Table S-17. More specifically, we use Ridge classification, which is described in Sec. S-VI B. We consider linear and quadratic functions for $\phi(\cdot)$ in Eq. (S-VI.2.1) with and without normalization. We set $\lambda = 10^{-2}, 10^{-1}, 1$ where λ is the coefficient of the regularization term.

Next, we show the performance dependence of the three algorithms on their key parameters. We see the performance dependence of QCL on the number of layers L. The result is shown in Fig. S-41. We then see the performance dependence of the UKM on r, which is the coefficient of the second term in the right-hand side of Eq. (S-IX.1.8). The

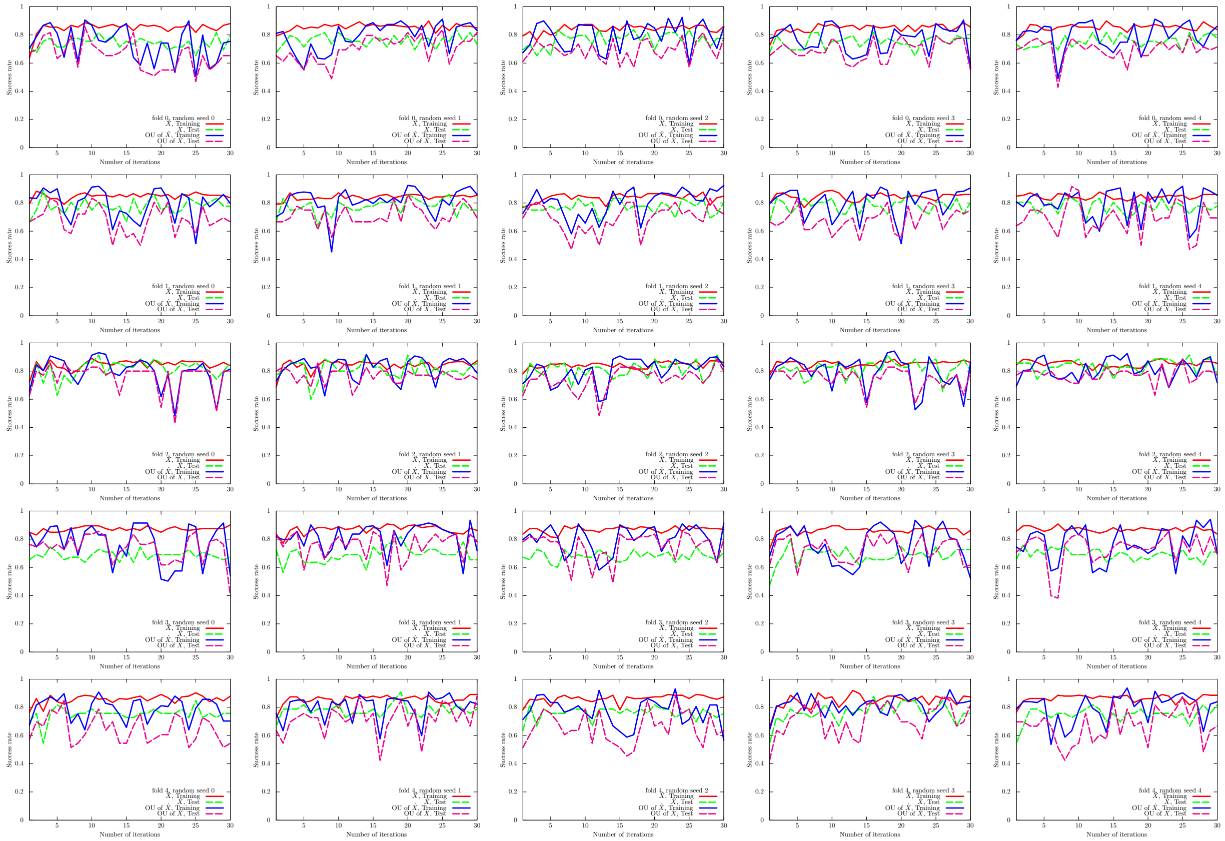

FIG. S-39: Results of the UKM ($\hat{X}$ and OU of $\hat{X}$) on the 5-fold datasets with 5 different random seeds for the sonar dataset (0 or 1). We use complex matrices for the initial input and set $\theta_{\text{bias}} = 0$. We set $r = 0.010$.

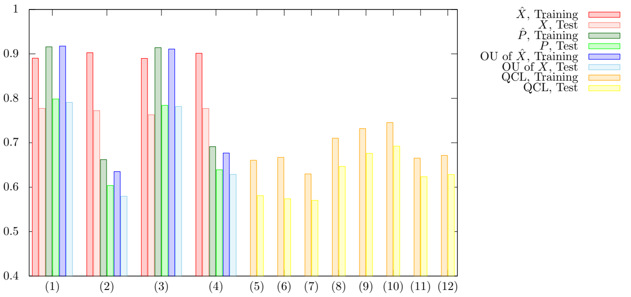

FIG. S-40: Results of 5-fold CV with 5 different random seeds for the sonar dataset (0 or 1). For the UKM, we put $r = 0.010$ and set $K = 30$ and $K' = 10$. For QCL, the number of layers L is 5 and the number of iterations is 300. The numerical settings are as follows: (1) UKM: complex matrices without the bias term, (2) UKM: complex matrices with the bias term, (3) UKM: real matrices without the bias term, (4) UKM: real matrices with the bias term, (5) QCL: CNOT-based circuit without the bias term, (6) QCL: CNOT-based circuit with the bias term, (7) QCL: CRot-based circuit without the bias term, (8) QCL: CRot-based circuit with the bias term, (9) QCL: 1d Heisenberg circuit without the bias term, (10) QCL: 1d Heisenberg circuit with the bias term, (11) QCL: FC Heisenberg circuit without the bias term, and (12) QCL: FC Heisenberg circuit with the bias term.

result is shown in Fig. S-42. In Fig. S-43, we show the performance dependence of the kernel method on λ, which is the coefficient of the second term in the right-hand side of Eq. (S-VI.2.4).

So far, we have used the squared error function $\ell_{\text{SE}}(\cdot,\cdot)$, Eq. (S-V.4.2). In Fig. S-44, we show the performance dependence of QCL on the number of layers L in the case of the hinge function $\ell_{\text{hinge}}(\cdot,\cdot)$, Eq. (S-V.4.3). In Fig. S-45, we show the performance dependence of the UKM on r, which is the coefficient of the second term in the right-hand side of Eq. (S-IX.1.8), in the case of the hinge function $\ell_{\text{hinge}}(\cdot,\cdot)$, Eq. (S-V.4.3).

Algo.	Condition	Training	Test
Kernel method	Linear, w/o normalization, $\lambda = 10^{-2}$	0.9109	0.7574
Kernel method	Linear, w/o normalization, $\lambda = 10^{-1}$	0.8738	0.7717
Kernel method	Linear, w/o normalization, $\lambda = 1$	0.8667	0.7631
Kernel method	Linear, w/ normalization, $\lambda = 10^{-2}$	0.8806	0.7694
Kernel method	Linear, w/ normalization, $\lambda = 10^{-1}$	0.8522	0.7500
Kernel method	Linear, w/ normalization, $\lambda = 1$	0.8093	0.7076
Kernel method	Quadratic, w/o normalization, $\lambda = 10^{-2}$	1.0000	0.8198
Kernel method	Quadratic, w/o normalization, $\lambda = 10^{-1}$	1.0000	0.8084
Kernel method	Quadratic, w/o normalization, $\lambda = 1$	0.9865	0.8035
Kernel method	Quadratic, w/ normalization, $\lambda = 10^{-2}$	0.9901	0.7973
Kernel method	Quadratic, w/ normalization, $\lambda = 10^{-1}$	0.9297	0.8009
Kernel method	Quadratic, w/ normalization, $\lambda = 1$	0.8348	0.7374

TABLE S-17: Results of 5-fold CV with 5 different random seeds of the kernel method for the sonar dataset (0 or 1). We set $\lambda = 10^{-2}, 10^{-1}, 1$. For $\phi(\cdot)$, we use linear and quadratic functions with and without normalization. We use the squared error function.

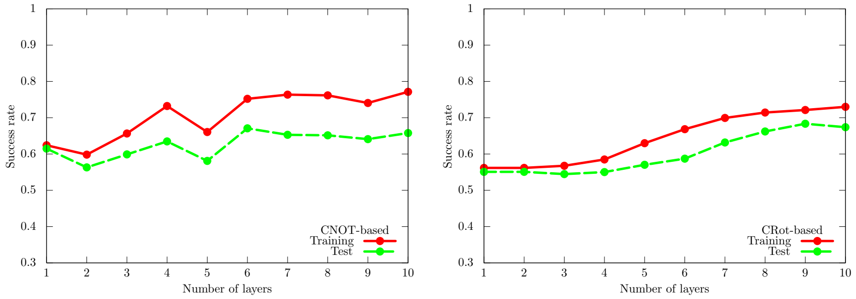

FIG. S-41: Performance dependence of QCL on the number of layers L for the sonar dataset (0 or 1). We use the CNOT-based and CRot-based circuits. We set $\theta_{\text{bias}} = 0$. We iterate the computation 300 times.

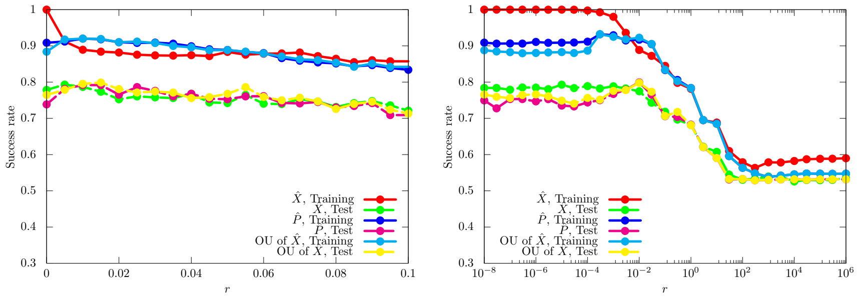

FIG. S-42: Performance dependence of the UKM on r, which is the coefficient of the second term in the right-hand side of Eq. (S-IX.1.8) for the sonar dataset (0 or 1). We show the performance obtained by $\hat{X}$, $\hat{P}$, and OU of $\hat{X}$. Note that $\hat{P}$, and OU of $\hat{X}$ strictly satisfy the unitarity constraint while $\hat{X}$ does not. We use complex matrices for the initial input and set $\theta_{\text{bias}} = 0$. We set $K = 30$ and $K' = 10$.

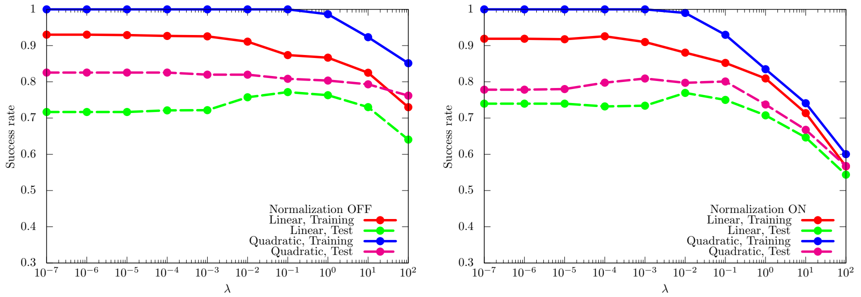

FIG. S-43: Performance dependence of the kernel method on λ, which is the coefficient of the second term in the right-hand side of Eq. (S-VI.2.4) for the sonar dataset (0 or 1). For $\phi(\cdot)$ in Eq. (S-VI.2.1), we use linear and quadratic functions with and without normalization.

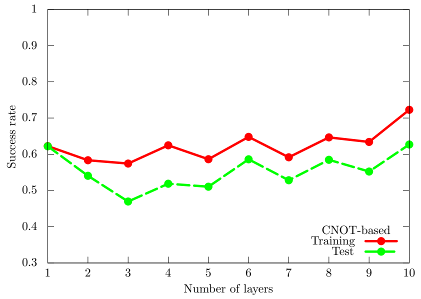

FIG. S-44: Performance dependence of QCL on the number of layers L for the sonar dataset (0 or 1) in the case of the hinge function $\ell_{\text{hinge}}(\cdot,\cdot)$, Eq. (S-V.4.3). We use the CNOT-based circuit. We set $\theta_{\text{bias}} = 0$. We iterate the computation 300 times.

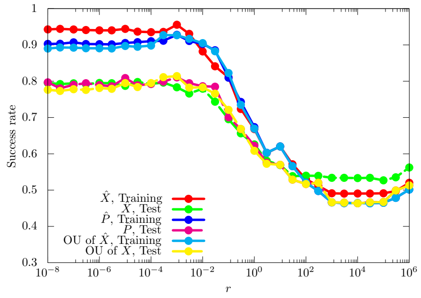

FIG. S-45: Performance dependence of the UKM on r, which is the coefficient of the second term in the right-hand side of Eq. (S-IX.1.8) for the sonar dataset (0 or 1) in the case of the hinge function $\ell_{\text{hinge}}(\cdot,\cdot)$, Eq. (S-V.4.3). We show the performance obtained by $\hat{X}$, $\hat{P}$, and OU of $\hat{X}$. Note that $\hat{P}$, and OU of $\hat{X}$ strictly satisfy the unitarity constraint while $\hat{X}$ does not. We use complex matrices for the initial input and set $\theta_{\text{bias}} = 0$. We set $K = 30$ and $K' = 10$.

I. Wine dataset (0 or non-0)

We here show the numerical result for the wine dataset (0 or non-0). For the UKM, we put $r = 0.010$ and set $K = 30$ and $K' = 10$ in Algo. S-10. For QCL, we run iterations 300 times. We use the squared error function $\ell_{\mathrm{SE}}(\cdot,\cdot)$, Eq. (S-V.4.2).

In Fig. S-46, we show the numerical results of QCL for the 5-fold datasets with 5 different random seeds. In

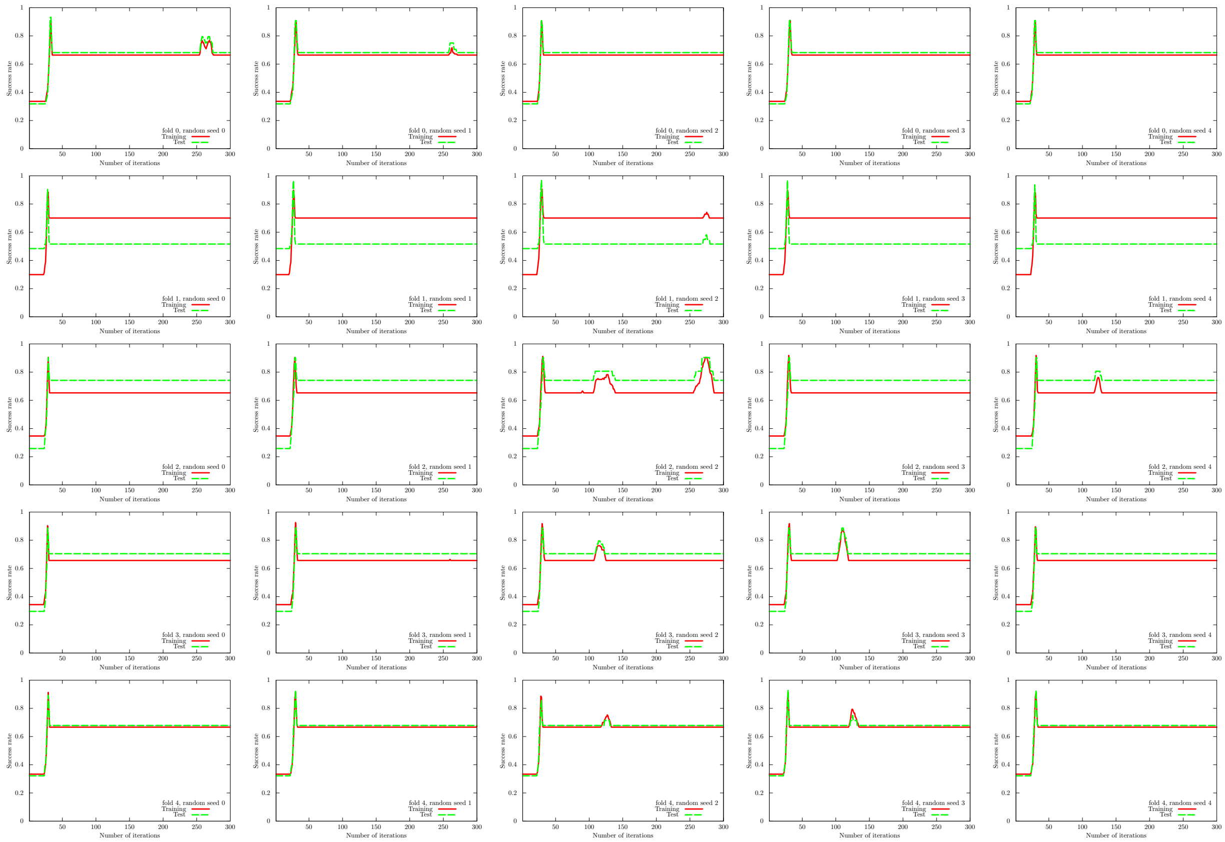

FIG. S-46: Results of QCL on the 5-fold datasets with 5 different random seeds for the wine dataset (0 or non-0). We use the CNOT-based circuit and set $\theta_{\mathrm{bias}} = 0$. The number of layers L is set to 5.

Fig. S-47, we show the numerical results of $\hat{P}$ of the UKM for the 5-fold datasets with 5 different random seeds. In Fig. S-48, we also show the numerical results of OU of $\hat{X}$ of the UKM for the 5-fold datasets with 5 different random seeds.

We summarize the results of 5-fold CV with 5 different random seeds of QCL and the UKM in Tables S-18 and S-19, respectively. For QCL and the UKM, we select the best model for the training dataset over iterations to compute the performance. In Fig. S-49, we plot the data shown in Tables S-18 and S-19. We also summarize the results of 5-fold

Algo.	Condition	Training	Test
QCL	CNOT-based, w/o bias	0.9057	0.9052
QCL	CNOT-based, w/ bias	0.8665	0.8680
QCL	CRot-based, w/o bias	0.9151	0.9086
QCL	CRot-based, w/ bias	0.9145	0.9059
QCL	1d Heisenberg, w/o bias	0.9155	0.9126
QCL	1d Heisenberg, w/ bias	0.8547	0.8440
QCL	FC Heisenberg, w/o bias	0.9103	0.9052
QCL	FC Heisenberg, w/ bias	0.8390	0.8318

TABLE S-18: Results of 5-fold CV with 5 different random seeds of QCL for the wine dataset (0 or non-0). We consider four types of circuits with and without the bias term: the CNOT-based circuit, the CRot-based circuit, 1d Heisenberg circuit, and the FC Heisenberg circuit. The number of layers L is set to 5 and the number of iterations is set to 300.

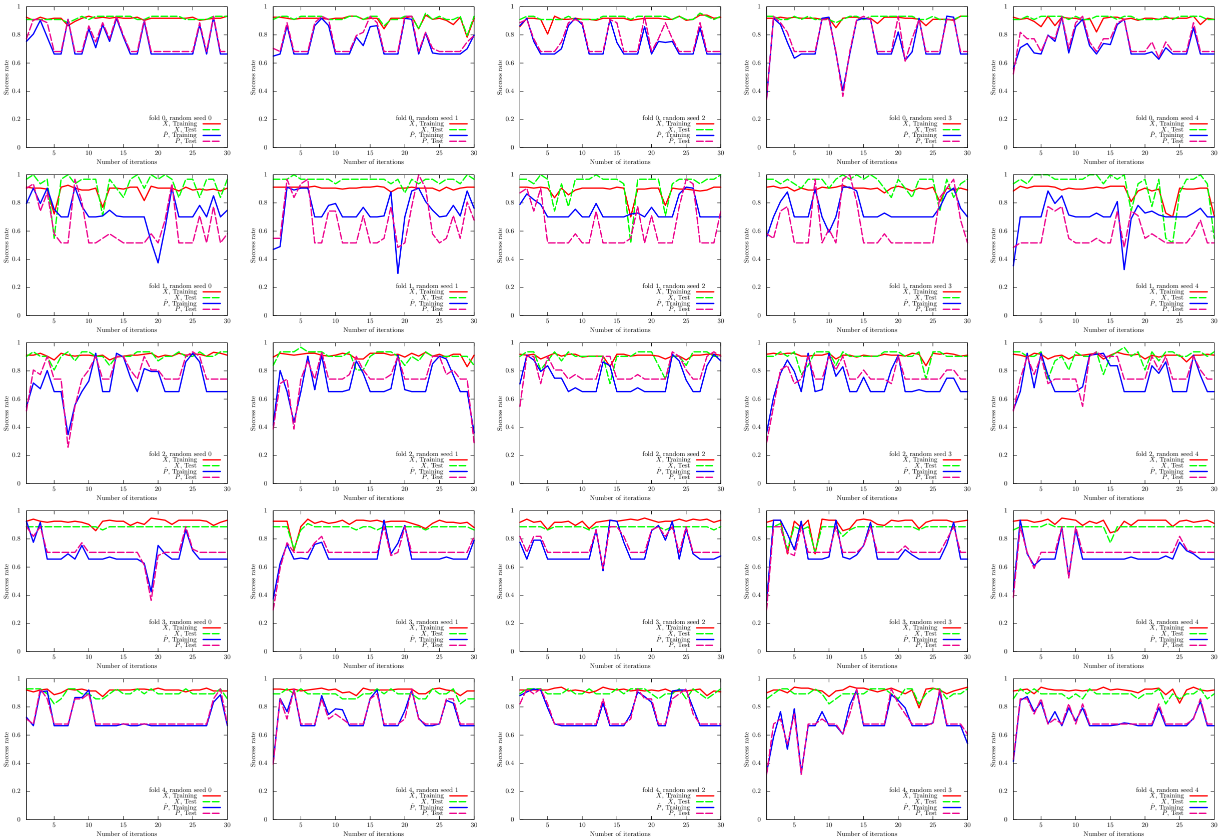

FIG. S-47: Results of the UKM ($\hat{X}$ and $\hat{P}$) on the 5-fold datasets with 5 different random seeds for the wine dataset (0 or non-0). We use complex matrices for the initial input and set $\theta_{\text{bias}} = 0$. We set $r = 0.010$.

Algo.	Condition	Training	Test
UKM	$\hat{X}$, complex, w/o bias	0.9328	0.9243
UKM	$\hat{P}$, complex, w/o bias	0.9213	0.9116
UKM	OU of $\hat{X}$, complex, w/o bias	0.9215	0.9159
UKM	$\hat{X}$, complex, w/ bias	0.9364	0.9313
UKM	$\hat{P}$, complex, w/ bias	0.6420	0.6184
UKM	OU of $\hat{X}$, complex, w/ bias	0.6282	0.6128
UKM	$\hat{X}$, real, w/o bias	0.9359	0.9292
UKM	$\hat{P}$, real, w/o bias	0.9200	0.9185
UKM	OU of $\hat{X}$, real, w/o bias	0.9212	0.9171
UKM	$\hat{X}$, real, w/ bias	0.9345	0.9212
UKM	$\hat{P}$, real, w/ bias	0.6062	0.6055
UKM	OU of $\hat{X}$, real, w/ bias	0.5716	0.5708

TABLE S-19: Results of 5-fold CV with 5 different random seeds of the UKM for the wine dataset (0 or non-0). We show the performance obtained by $\hat{X}$, $\hat{P}$, and OU of $\hat{X}$. Note that $\hat{P}$, and OU of $\hat{X}$ strictly satisfy the unitarity constraint while $\hat{X}$ does not. We consider real and complex matrices for the initial input with and without the bias term. We put $r = 0.010$ and set $K = 30$ and $K' = 10$.

CV with 5 different random seeds of the kernel method in Table S-20. More specifically, we use Ridge classification, which is described in Sec. S-VI B. We consider linear and quadratic functions for $\phi(\cdot)$ in Eq. (S-VI.2.1) with and without normalization. We set $\lambda = 10^{-2}, 10^{-1}, 1$ where λ is the coefficient of the regularization term.

Next, we show the performance dependence of the three algorithms on their key parameters. We see the performance dependence of QCL on the number of layers L. The result is shown in Fig. S-50. We then see the performance dependence of the UKM on r, which is the coefficient of the second term in the right-hand side of Eq. (S-IX.1.8). The

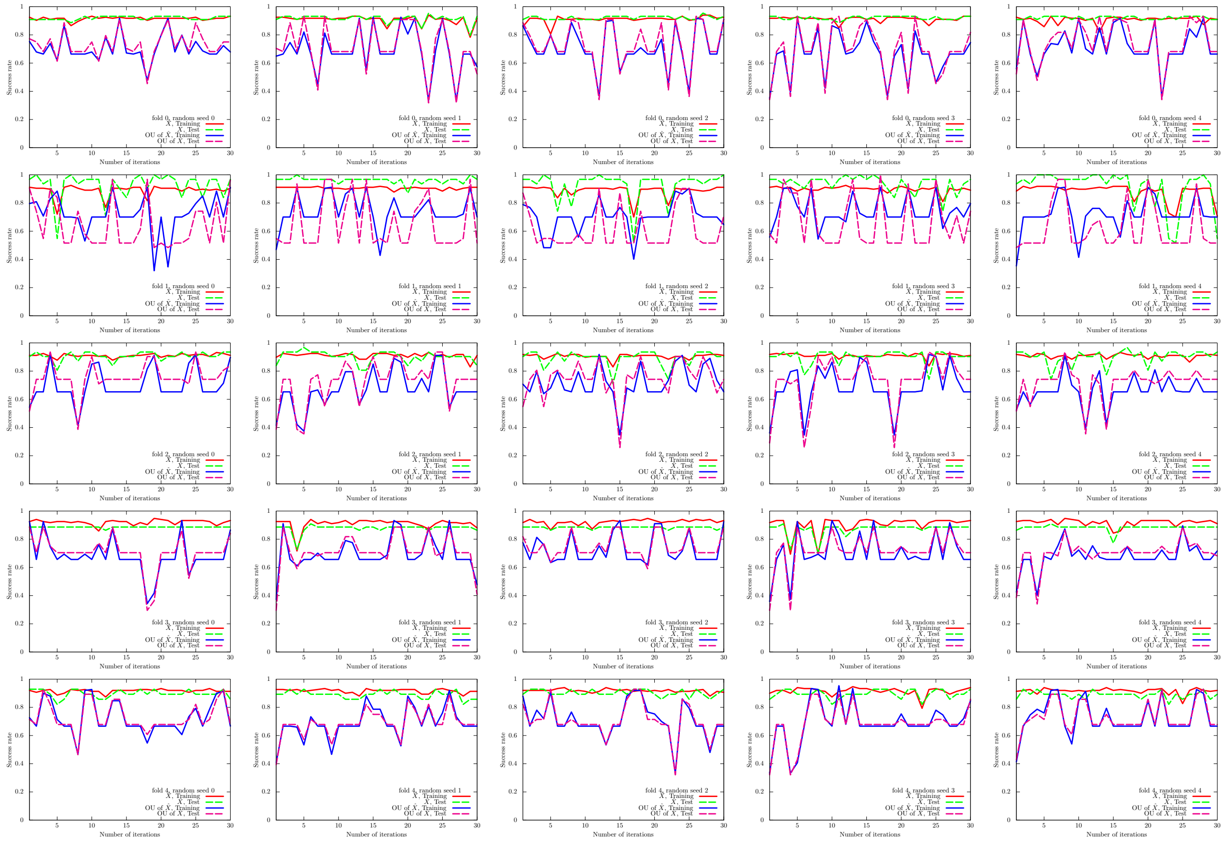

FIG. S-48: Results of the UKM ($\hat{X}$ and OU of $\hat{X}$) on the 5-fold datasets with 5 different random seeds for the wine dataset (0 or non-0). We use complex matrices for the initial input and set $\theta_{\text{bias}} = 0$. We set $r = 0.010$.

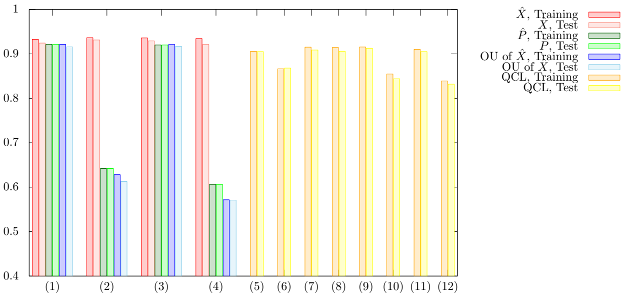

FIG. S-49: Results of 5-fold CV with 5 different random seeds for the wine dataset (0 or non-0). For the UKM, we put $r = 0.010$ and set $K = 30$ and $K' = 10$. For QCL, the number of layers L is 5 and the number of iterations is 300. The numerical settings are as follows: (1) UKM: complex matrices without the bias term, (2) UKM: complex matrices with the bias term, (3) UKM: real matrices without the bias term, (4) UKM: real matrices with the bias term, (5) QCL: CNOT-based circuit without the bias term, (6) QCL: CNOT-based circuit with the bias term, (7) QCL: CRot-based circuit without the bias term, (8) QCL: CRot-based circuit with the bias term, (9) QCL: 1d Heisenberg circuit without the bias term, (10) QCL: 1d Heisenberg circuit with the bias term, (11) QCL: FC Heisenberg circuit without the bias term, and (12) QCL: FC Heisenberg circuit with the bias term.

result is shown in Fig. S-51. In Fig. S-52, we show the performance dependence of the kernel method on λ, which is the coefficient of the second term in the right-hand side of Eq. (S-VI.2.4).

So far, we have used the squared error function $\ell_{\text{SE}}(\cdot,\cdot)$, Eq. (S-V.4.2). In Fig. S-53, we show the performance dependence of QCL on the number of layers L in the case of the hinge function $\ell_{\text{hinge}}(\cdot,\cdot)$, Eq. (S-V.4.3). In Fig. S-54, we show the performance dependence of the UKM on r, which is the coefficient of the second term in the right-hand side of Eq. (S-IX.1.8), in the case of the hinge function $\ell_{\text{hinge}}(\cdot,\cdot)$, Eq. (S-V.4.3).

Algo.	Condition	Training	Test
Kernel method	Linear, w/o normalization, $\lambda = 10^{-2}$	0.9987	0.9955
Kernel method	Linear, w/o normalization, $\lambda = 10^{-1}$	0.9987	0.9883
Kernel method	Linear, w/o normalization, $\lambda = 1$	0.9987	0.9883
Kernel method	Linear, w/ normalization, $\lambda = 10^{-2}$	0.9357	0.9474
Kernel method	Linear, w/ normalization, $\lambda = 10^{-1}$	0.9230	0.9183
Kernel method	Linear, w/ normalization, $\lambda = 1$	0.6807	0.6711
Kernel method	Quadratic, w/o normalization, $\lambda = 10^{-2}$	1.0000	0.9481
Kernel method	Quadratic, w/o normalization, $\lambda = 10^{-1}$	1.0000	0.9558
Kernel method	Quadratic, w/o normalization, $\lambda = 1$	1.0000	0.9683
Kernel method	Quadratic, w/ normalization, $\lambda = 10^{-2}$	0.9455	0.9520
Kernel method	Quadratic, w/ normalization, $\lambda = 10^{-1}$	0.9259	0.9293
Kernel method	Quadratic, w/ normalization, $\lambda = 1$	0.8413	0.8258

TABLE S-20: Results of 5-fold CV with 5 different random seeds of the kernel method for the wine dataset (0 or non-0). We set $\lambda = 10^{-2}, 10^{-1}, 1$. For $\phi(\cdot)$, we use linear and quadratic functions with and without normalization. We use the squared error function.

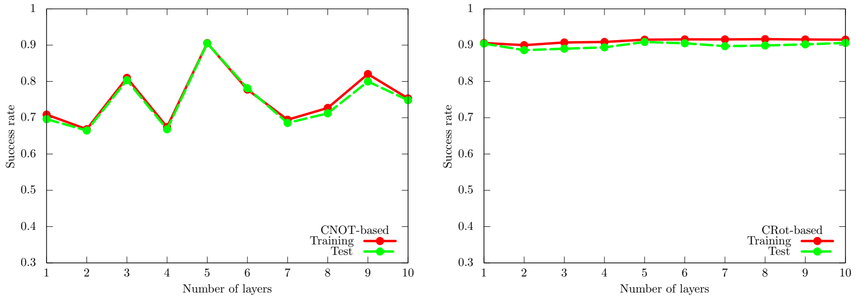

FIG. S-50: Performance dependence of QCL on the number of layers L for the wine dataset (0 or non-0). We use the CNOT-based and CRot-based circuits. We set $\theta_{\text{bias}} = 0$. We iterate the computation 300 times.

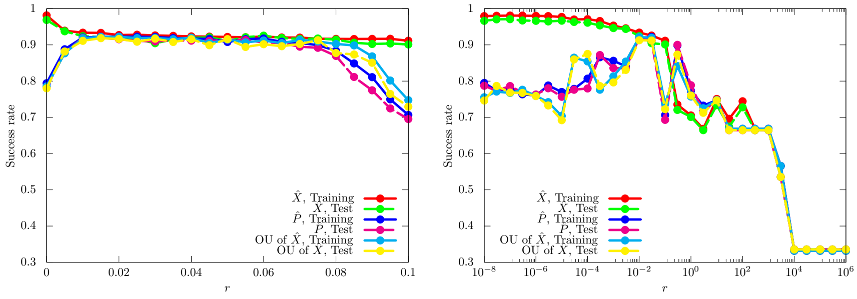

FIG. S-51: Performance dependence of the UKM on r, which is the coefficient of the second term in the right-hand side of Eq. (S-IX.1.8) for the wine dataset (0 or non-0). We show the performance obtained by $\hat{X}$, $\hat{P}$, and OU of $\hat{X}$. Note that $\hat{P}$, and OU of $\hat{X}$ strictly satisfy the unitarity constraint while $\hat{X}$ does not. We use complex matrices for the initial input and set $\theta_{\text{bias}} = 0$. We set $K = 30$ and $K' = 10$.

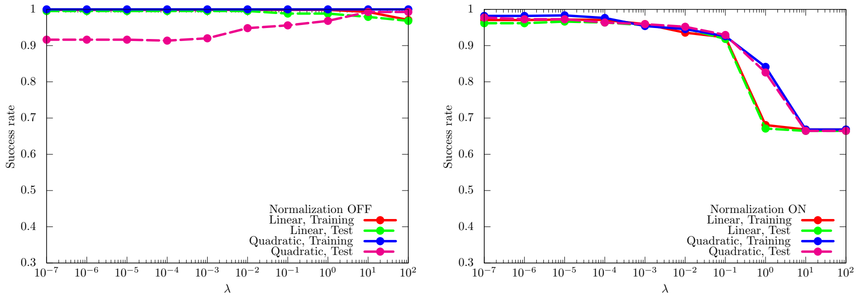

FIG. S-52: Performance dependence of the kernel method on λ, which is the coefficient of the second term in the right-hand side of Eq. (S-VI.2.4) for the wine dataset (0 or non-0). For $\phi(\cdot)$ in Eq. (S-VI.2.1), we use linear and quadratic functions with and without normalization.

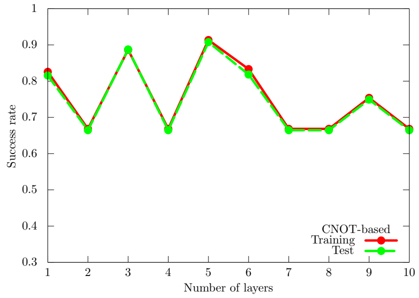

FIG. S-53: Performance dependence of QCL on the number of layers L for the wine dataset (0 or non-0) in the case of the hinge function $\ell_{\text{hinge}}(\cdot,\cdot)$, Eq. (S-V.4.3). We use the CNOT-based circuit. We set $\theta_{\text{bias}} = 0$. We iterate the computation 300 times.

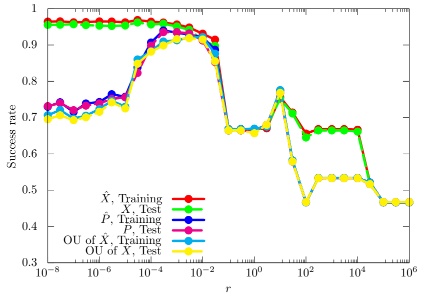

FIG. S-54: Performance dependence of the UKM on r, which is the coefficient of the second term in the right-hand side of Eq. (S-IX.1.8) for the wine dataset (0 or non-0) in the case of the hinge function $\ell_{\text{hinge}}(\cdot,\cdot)$, Eq. (S-V.4.3). We show the performance obtained by $\hat{X}$, $\hat{P}$, and OU of $\hat{X}$. Note that $\hat{P}$, and OU of $\hat{X}$ strictly satisfy the unitarity constraint while $\hat{X}$ does not. We use complex matrices for the initial input and set $\theta_{\text{bias}} = 0$. We set $K = 30$ and $K' = 10$.

J. Semeion dataset (0 or 1)

We here show the numerical result for the semeion dataset (0 or 1). For the UKM, we put $r = 0.010$ and set $K = 20$ and $K' = 10$ in Algo. S-10. For QCL, we run iterations 100 times. We use the squared error function $\ell_{\rm SE}(\cdot,\cdot)$, Eq. (S-V.4.2).

In Fig. S-55, we show the numerical results of QCL for the 5-fold datasets with 5 different random seeds. In

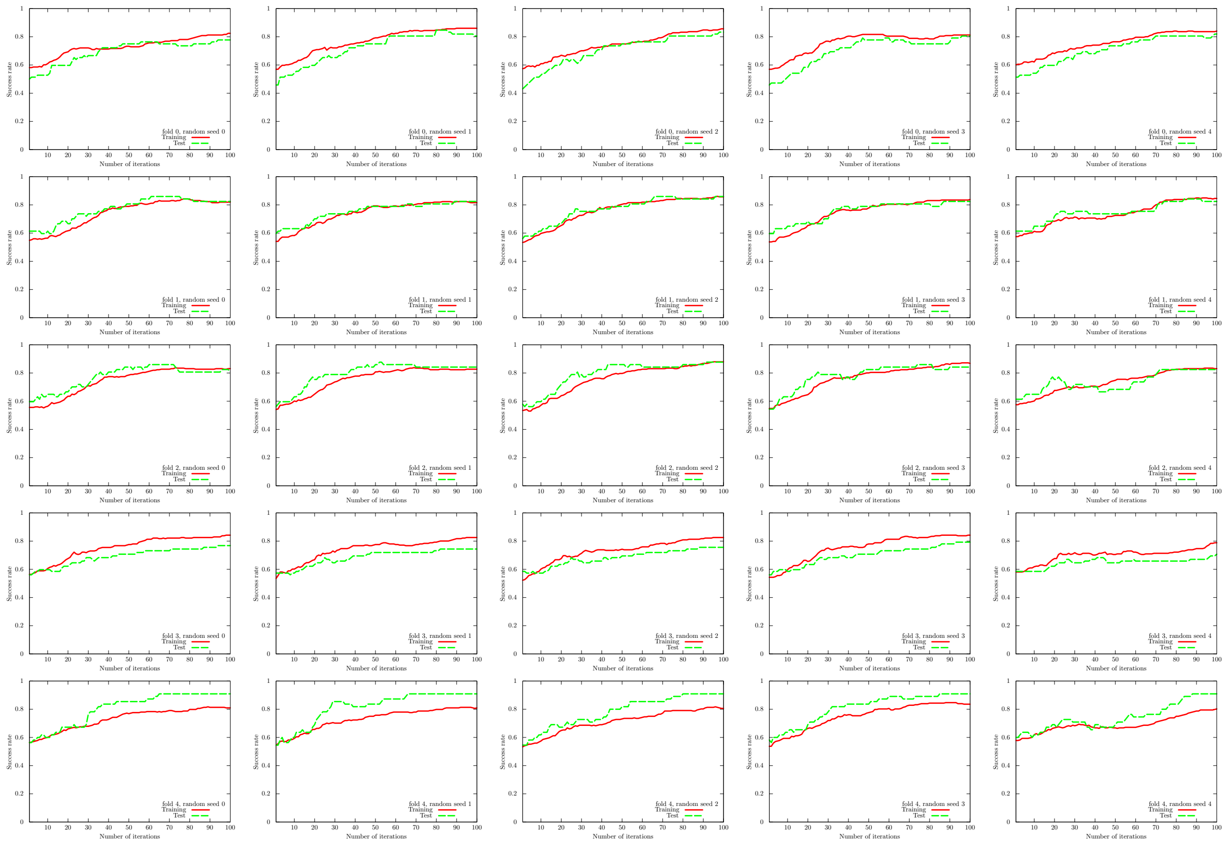

FIG. S-55: Results of QCL on the 5-fold datasets with 5 different random seeds for the semeion dataset (0 or 1). We use the CNOT-based circuit and set $\theta_{\rm bias} = 0$. The number of layers L is set to 5.

Fig. S-56, we show the numerical results of $\hat{P}$ of the UKM for the 5-fold datasets with 5 different random seeds. In Fig. S-57, we also show the numerical results of OU of $\hat{X}$ of the UKM for the 5-fold datasets with 5 different random seeds.

We summarize the results of 5-fold CV with 5 different random seeds of QCL and the UKM in Tables S-21 and S-22, respectively. For QCL and the UKM, we select the best model for the training dataset over iterations to compute the performance. In Fig. S-58, we plot the data shown in Tables S-21 and S-22. We also summarize the results of 5-fold

Algo.	Condition	Training	Test
QCL	CNOT-based, w/o bias	0.8356	0.8288
QCL	CNOT-based, w/ bias	0.9210	0.9099
QCL	CRot-based, w/o bias	0.4867	0.4566
QCL	CRot-based, w/ bias	0.7222	0.7162

TABLE S-21: Results of 5-fold CV with 5 different random seeds of QCL for the semeion dataset (0 or 1). We consider four types of circuits with and without the bias term: the CNOT-based circuit, the CRot-based circuit, 1d Heisenberg circuit, and the FC Heisenberg circuit. The number of layers L is 5 and the number of iterations is 100.

CV with 5 different random seeds of the kernel method in Table S-23. More specifically, we use Ridge classification, which is described in Sec. S-VI B. We consider linear and quadratic functions for $\phi(\cdot)$ in Eq. (S-VI.2.1) with and without normalization. We set $\lambda = 10^{-2}, 10^{-1}, 1$ where λ is the coefficient of the regularization term.

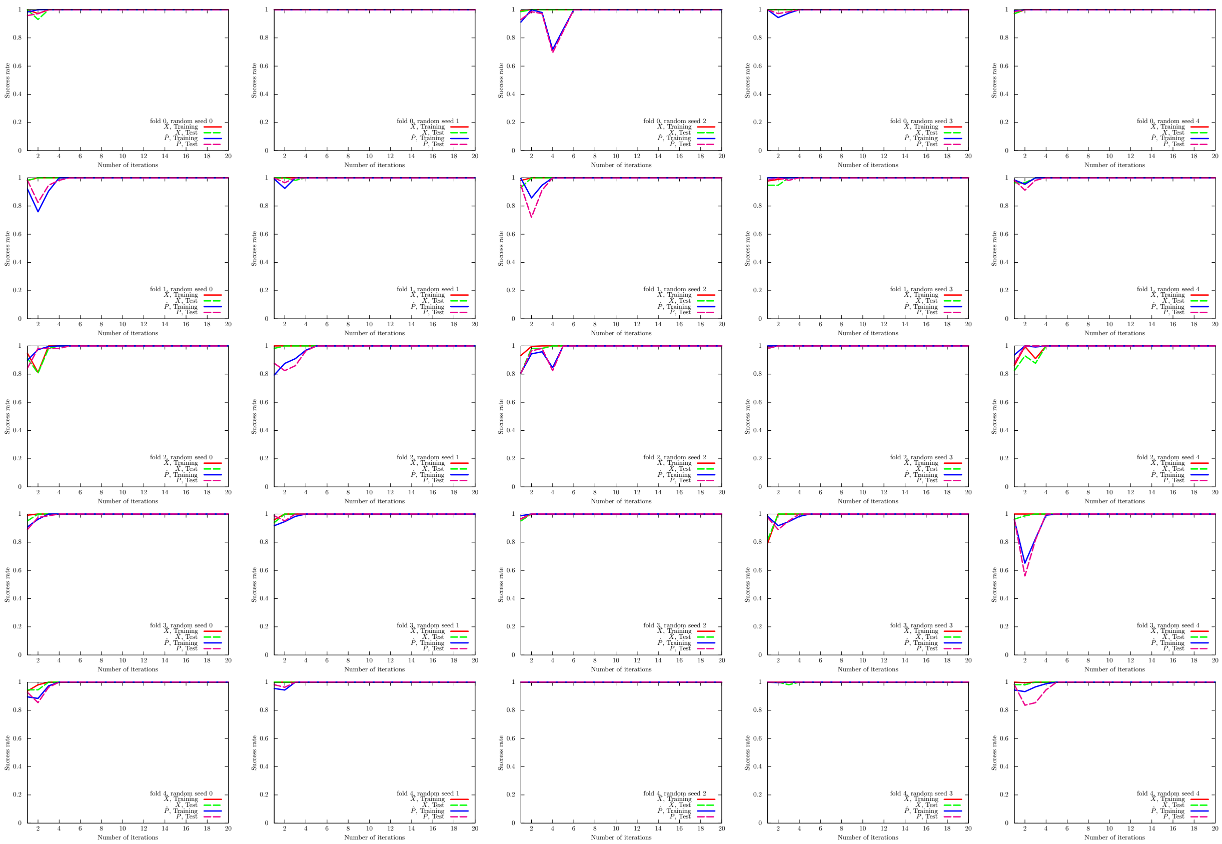

FIG. S-56: Results of the UKM ($\hat{X}$ and $\hat{P}$) on the 5-fold datasets with 5 different random seeds for the semeion dataset (0 or 1). We use complex matrices for the initial input and set $\theta_{\mathrm{bias}} = 0$. We set $r = 0.010$.

Algo.	Condition	Training	Test
UKM	$\hat{X}$, complex, w/o bias	1.0	0.9957
UKM	$\hat{P}$, complex, w/o bias	1.0	0.9943
UKM	OU of $\hat{X}$, complex, w/o bias	1.0	0.9941
UKM	$\hat{X}$, complex, w/ bias	1.0	0.9922
UKM	$\hat{P}$, complex, w/ bias	1.0	0.9735
UKM	OU of $\hat{X}$, complex, w/ bias	1.0	0.9755
UKM	$\hat{X}$, real, w/o bias	1.0	0.9939
UKM	$\hat{P}$, real, w/o bias	1.0	0.9939
UKM	OU of $\hat{X}$, real, w/o bias	1.0	0.9945
UKM	$\hat{X}$, real, w/ bias	1.0	0.9941
UKM	$\hat{P}$, real, w/ bias	1.0	0.9814
UKM	OU of $\hat{X}$, real, w/ bias	1.0	0.9865

TABLE S-22: Results of 5-fold CV with 5 different random seeds of the UKM for the semeion dataset (0 or 1). We show the performance obtained by $\hat{X}$, $\hat{P}$, and OU of $\hat{X}$. Note that $\hat{P}$, and OU of $\hat{X}$ strictly satisfy the unitarity constraint while $\hat{X}$ does not. We consider real and complex matrices for the initial input with and without the bias term. We put $r = 0.010$ and set $K = 20$ and $K = 10$.

Next, we show the performance dependence of the three algorithms on their key parameters. We see the performance dependence of QCL on the number of layers L. The result is shown in Fig. S-59. We then see the performance dependence of the UKM on r, which is the coefficient of the second term in the right-hand side of Eq. (S-IX.1.8). The result is shown in Fig. S-60. In Fig. S-61, we show the performance dependence of the kernel method on λ, which is the coefficient of the second term in the right-hand side of Eq. (S-VI.2.4).

So far, we have used the squared error function $\ell_{\mathrm{SE}}(\cdot,\cdot)$, Eq. (S-V.4.2). In Fig. S-62, we show the performance

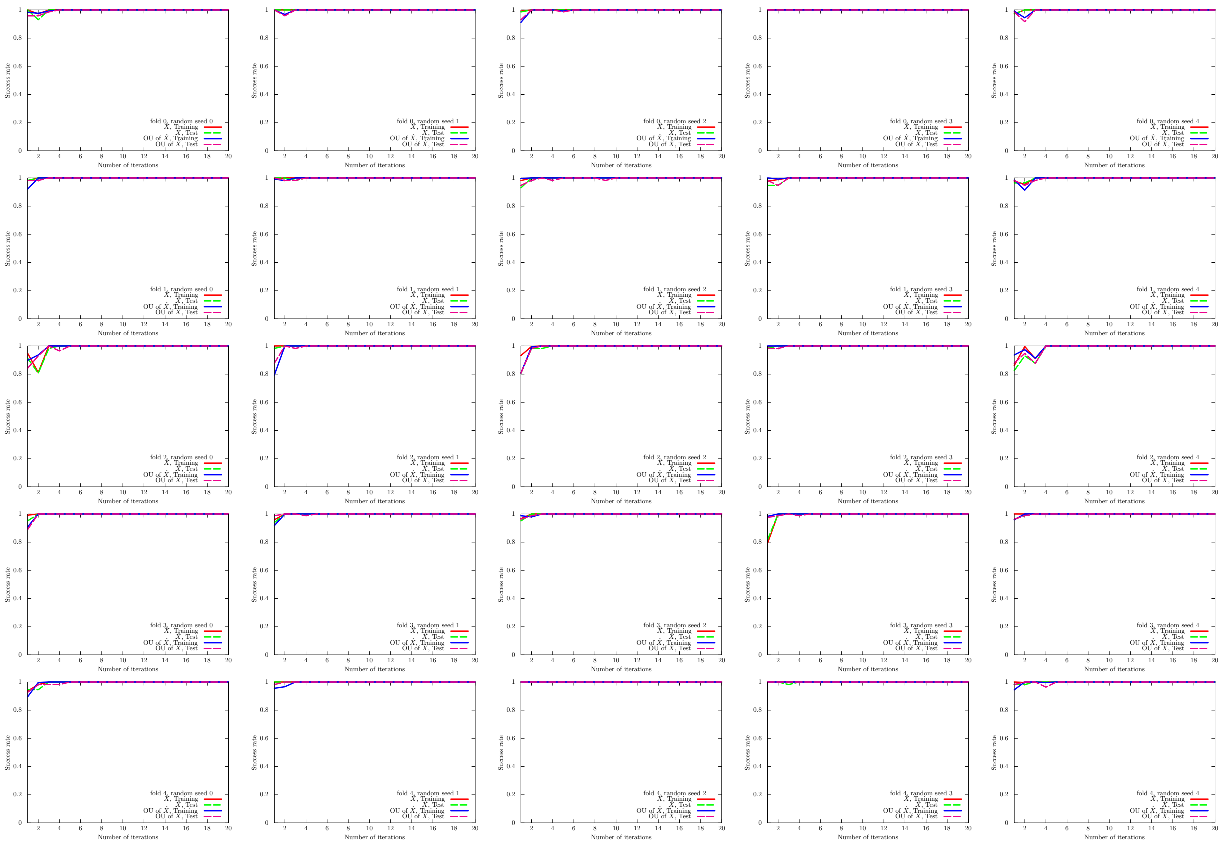

FIG. S-57: Results of the UKM ($\hat{X}$ and OU of $\hat{X}$) on the 5-fold datasets with 5 different random seeds for the semeion dataset (0 or 1). We use complex matrices for the initial input and set $\theta_{\mathrm{bias}} = 0$. We set $r = 0.010$.

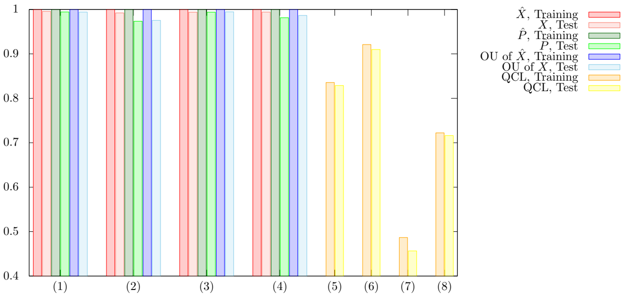

FIG. S-58: Results of 5-fold CV with 5 different random seeds for the semeion dataset (0 or 1). For the UKM, we put $r = 0.010$ and set $K = 20$ and $K' = 10$. For QCL, the number of layers L is 5 and the number of iterations is 100. The numerical settings are as follows: (1) UKM: complex matrices without the bias term, (2) UKM: complex matrices with the bias term, (3) UKM: real matrices without the bias term, (4) UKM: real matrices with the bias term, (5) QCL: CNOT-based circuit without the bias term, (6) QCL: CNOT-based circuit with the bias term, (7) QCL: CRot-based circuit without the bias term, (8) QCL: CRot-based circuit with the bias term, (9) QCL: 1d Heisenberg circuit without the bias term, (10) QCL: 1d Heisenberg circuit with the bias term, (11) QCL: FC Heisenberg circuit without the bias term, and (12) QCL: FC Heisenberg circuit with the bias term.

dependence of QCL on the number of layers L in the case of the hinge function $\ell_{\mathrm{hinge}}(\cdot,\cdot)$, Eq. (S-V.4.3). In Fig. S-63, we show the performance dependence of the UKM on r, which is the coefficient of the second term in the right-hand side of Eq. (S-IX.1.8), in the case of the hinge function $\ell_{\mathrm{hinge}}(\cdot,\cdot)$, Eq. (S-V.4.3).

Algo.	Condition	Training	Test
Kernel method	Linear, w/o normalization, $\lambda = 10^{-2}$	1.0000	0.8688
Kernel method	Linear, w/o normalization, $\lambda = 10^{-1}$	1.0000	0.9413
Kernel method	Linear, w/o normalization, $\lambda = 1$	1.0000	0.9822
Kernel method	Linear, w/ normalization, $\lambda = 10^{-2}$	1.0000	0.9858
Kernel method	Linear, w/ normalization, $\lambda = 10^{-1}$	1.0000	0.9965
Kernel method	Linear, w/ normalization, $\lambda = 1$	1.0000	1.0000
Kernel method	Quadratic, w/o normalization, $\lambda = 10^{-2}$	1.0000	0.9895
Kernel method	Quadratic, w/o normalization, $\lambda = 10^{-1}$	1.0000	0.9895
Kernel method	Quadratic, w/o normalization, $\lambda = 1$	1.0000	0.9895
Kernel method	Quadratic, w/ normalization, $\lambda = 10^{-2}$	1.0000	0.9965
Kernel method	Quadratic, w/ normalization, $\lambda = 10^{-1}$	1.0000	0.9965
Kernel method	Quadratic, w/ normalization, $\lambda = 1$	1.0000	1.0000

TABLE S-23: Results of 5-fold CV with 5 different random seeds of the kernel method for the semeion dataset (0 or 1). We set $\lambda = 10^{-2}, 10^{-1}, 1$. For $\phi(\cdot)$, we use linear and quadratic functions with and without normalization. We use the squared error function.

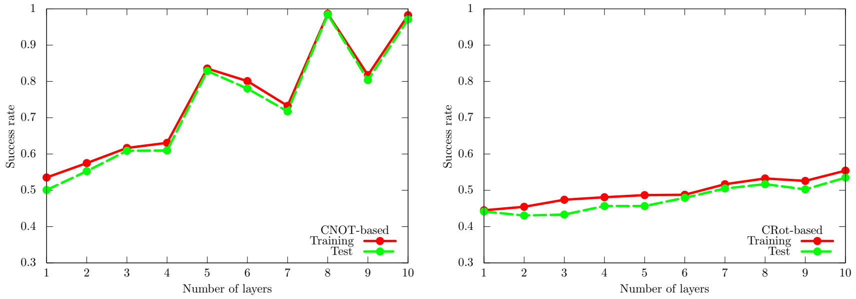

FIG. S-59: Performance dependence of QCL on the number of layers L for the semeion dataset (0 or 1). We use the CNOT-based and CRot-based circuits. We set $\theta_{\text{bias}} = 0$. We iterate the computation 100 times.

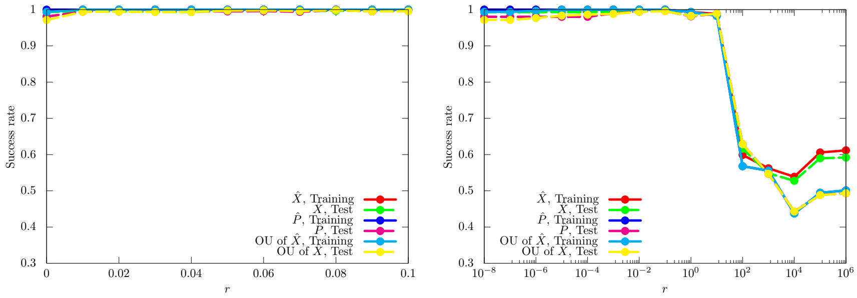

FIG. S-60: Performance dependence of the UKM on r, which is the coefficient of the second term in the right-hand side of Eq. (S-IX.1.8) for the semeion dataset (0 or 1). We show the performance obtained by $\hat{X}$, $\hat{P}$, and OU of $\hat{X}$. Note that $\hat{P}$, and OU of $\hat{X}$ strictly satisfy the unitarity constraint while $\hat{X}$ does not. We use complex matrices for the initial input and set $\theta_{\text{bias}} = 0$. We set $K = 20$ and $K' = 10$.

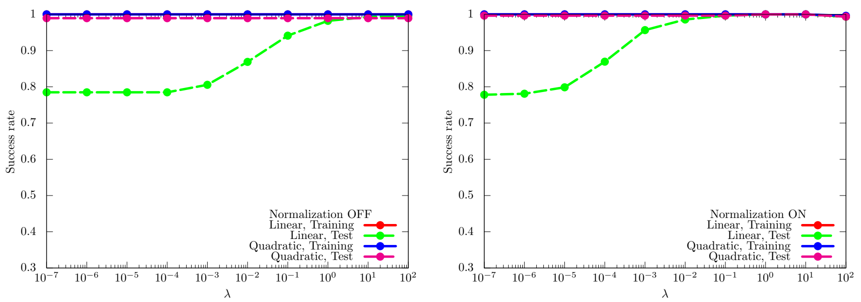

FIG. S-61: Performance dependence of the kernel method on λ, which is the coefficient of the second term in the right-hand side of Eq. (S-VI.2.4) for the semeion dataset (0 or 1). For $\phi(\cdot)$ in Eq. (S-VI.2.1), we use linear and quadratic functions with and without normalization.

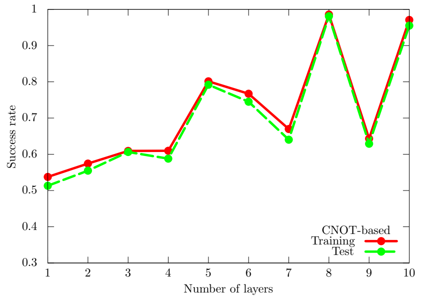

FIG. S-62: Performance dependence of QCL on the number of layers L for the semeion dataset (0 or 1) in the case of the hinge function $\ell_{\text{hinge}}(\cdot,\cdot)$, Eq. (S-V.4.3). We use the CNOT-based circuit. We set $\theta_{\text{bias}} = 0$. We iterate the computation 300 times.

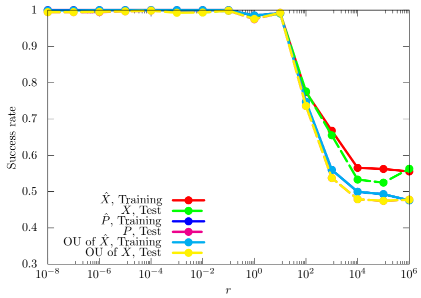

FIG. S-63: Performance dependence of the UKM on r, which is the coefficient of the second term in the right-hand side of Eq. (S-IX.1.8) for the semeion dataset (0 or 1) in the case of the hinge function $\ell_{\text{hinge}}(\cdot,\cdot)$, Eq. (S-V.4.3). We show the performance obtained by $\hat{X}$, $\hat{P}$, and OU of $\hat{X}$. Note that $\hat{P}$, and OU of $\hat{X}$ strictly satisfy the unitarity constraint while $\hat{X}$ does not. We use complex matrices for the initial input and set $\theta_{\text{bias}} = 0$. We set $K = 30$ and $K' = 10$.

K. Semeion dataset (0 or non-0)

We here show the numerical result for the semeion dataset (0 or non-0). For the UKM, we put $r = 0.010$ and set $K = 10$ and $K' = 5$ in Algo. S-10. For QCL, we run iterations 50 times. We use the squared error function $\ell_{\mathrm{SE}}(\cdot,\cdot)$, Eq. (S-V.4.2).

In Fig. S-64, we show the numerical results of QCL for the 5-fold datasets with 5 different random seeds. In

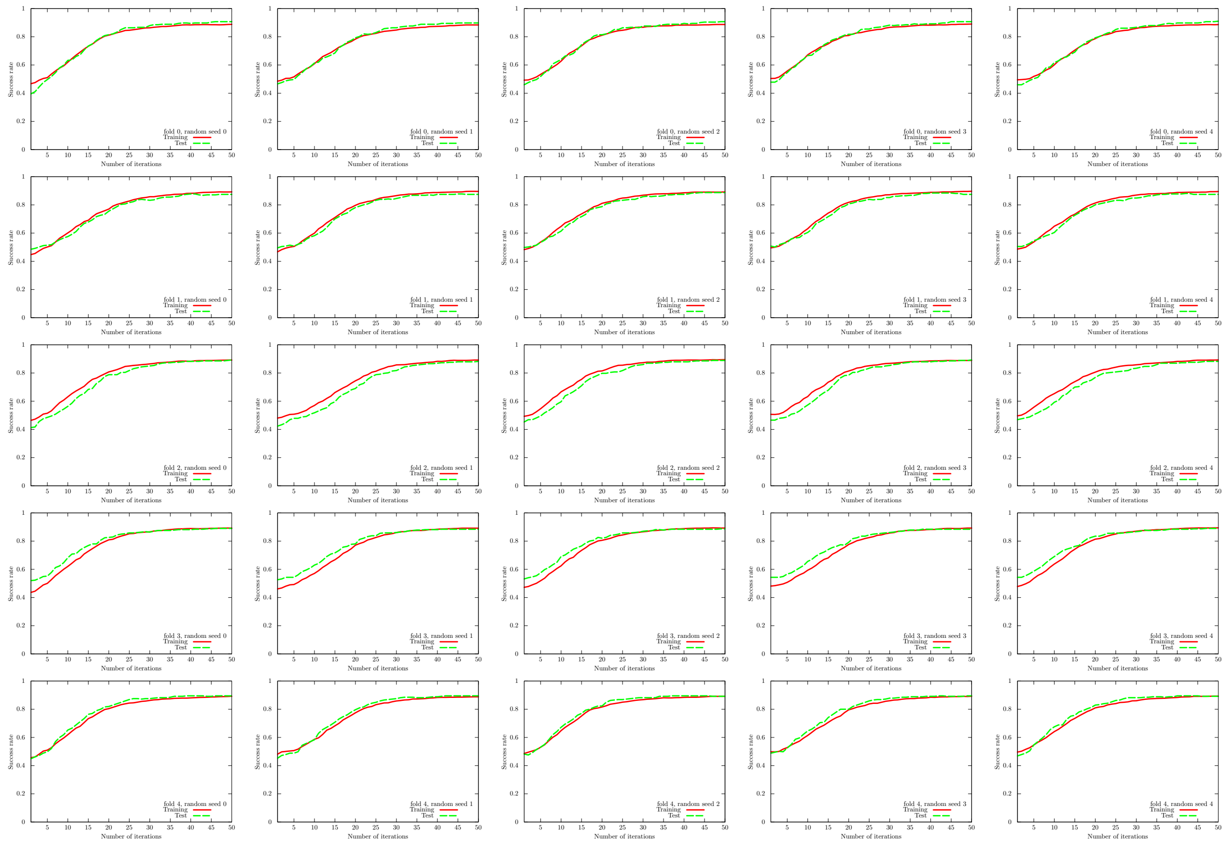

FIG. S-64: Results of QCL on the 5-fold datasets with 5 different random seeds for the semeion dataset (0 or non-0). We use the CNOT-based circuit and set $\theta_{\mathrm{bias}} = 0$. The number of layers L is set to 5.

Fig. S-65, we show the numerical results of $\hat{P}$ of the UKM for the 5-fold datasets with 5 different random seeds. In Fig. S-66, we also show the numerical results of OU of $\hat{X}$ of the UKM for the 5-fold datasets with 5 different random seeds.

We summarize the results of 5-fold CV with 5 different random seeds of QCL and the UKM in Tables S-24 and S-25, respectively. For QCL and the UKM, we select the best model for the training dataset over iterations to compute the performance. In Fig. S-67, we plot the data shown in Tables S-24 and S-25. We also summarize the results of 5-fold

Algo.	Condition	Training	Test
QCL	CNOT-based, w/o bias	0.8913	0.8896
QCL	CNOT-based, w/ bias	0.8989	0.8987
QCL	CRot-based, w/o bias	0.8959	0.8957
QCL	CRot-based, w/ bias	0.8989	0.8982

TABLE S-24: Results of 5-fold CV with 5 different random seeds of QCL for the semeion dataset (0 or non-0). We consider four types of circuits with and without the bias term: the CNOT-based circuit, the CRot-based circuit, 1d Heisenberg circuit, and the FC Heisenberg circuit. The number of layers L is 5 and the number of iterations is 50.

CV with 5 different random seeds of the kernel method in Table S-26. More specifically, we use Ridge classification, which is described in Sec. S-VI B. We consider linear and quadratic functions for $\phi(\cdot)$ in Eq. (S-VI.2.1) with and without normalization. We set $\lambda = 10^{-2}, 10^{-1}, 1$ where λ is the coefficient of the regularization term.

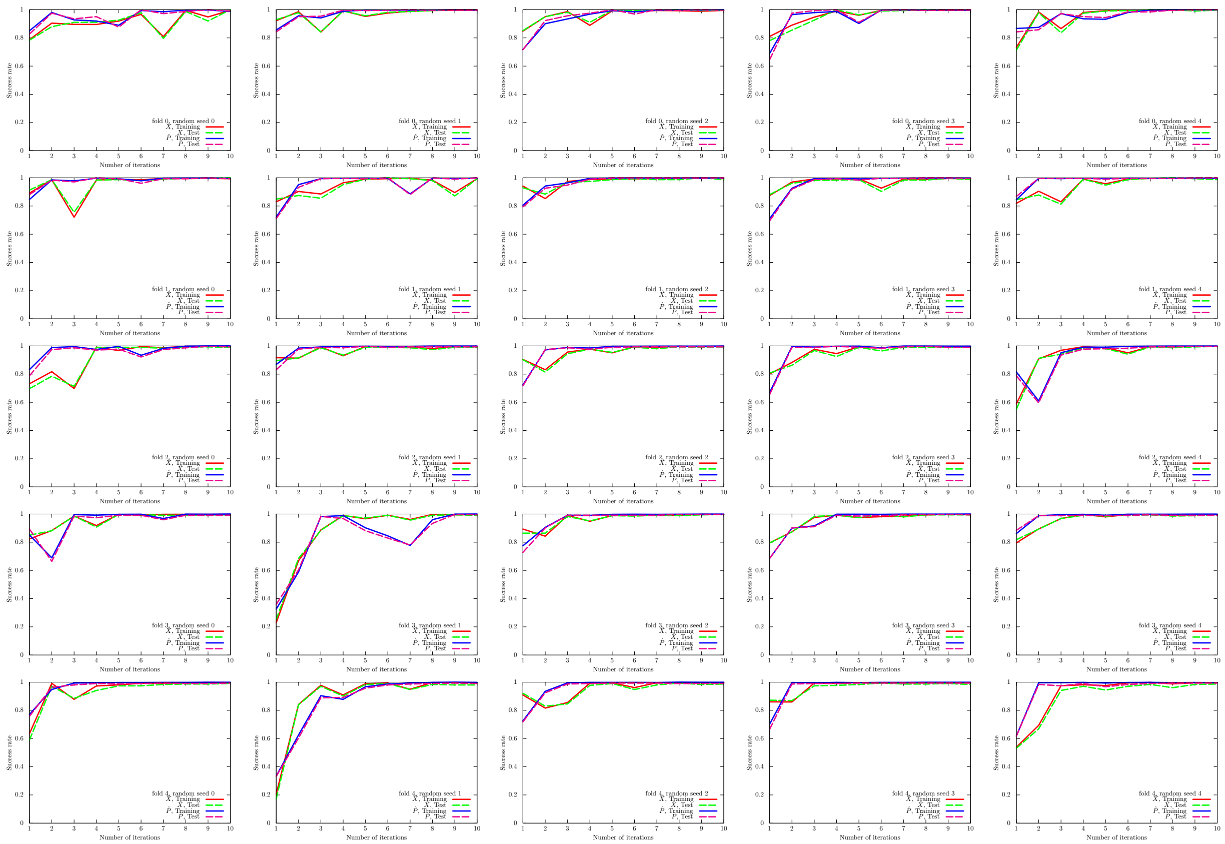

FIG. S-65: Results of the UKM ($\hat{X}$ and $\hat{P}$) on the 5-fold datasets with 5 different random seeds for the semeion dataset (0 or non-0). We use complex matrices for the initial input and set $\theta_{\text{bias}} = 0$. We set $r = 0.010$.

Algo.	Condition	Training	Test
UKM	$\hat{X}$, complex, w/o bias	0.9969	0.9925
UKM	$\hat{P}$, complex, w/o bias	0.9989	0.9945
UKM	OU of $\hat{X}$, complex, w/o bias	0.9990	0.9953
UKM	$\hat{X}$, complex, w/ bias	0.9968	0.9921
UKM	$\hat{P}$, complex, w/ bias	0.9127	0.9094
UKM	OU of $\hat{X}$, complex, w/ bias	0.9113	0.9071
UKM	$\hat{X}$, real, w/o bias	0.9961	0.9920
UKM	$\hat{P}$, real, w/o bias	0.9988	0.9949
UKM	OU of $\hat{X}$, real, w/o bias	0.9985	0.9938
UKM	$\hat{X}$, real, w/ bias	0.9969	0.9912
UKM	$\hat{P}$, real, w/ bias	0.9263	0.9214
UKM	OU of $\hat{X}$, real, w/ bias	0.9251	0.9213

TABLE S-25: Results of 5-fold CV with 5 different random seeds of the UKM for the semeion dataset (0 or non-0). We show the performance obtained by $\hat{X}$, $\hat{P}$, and OU of $\hat{X}$. Note that $\hat{P}$, and OU of $\hat{X}$ strictly satisfy the unitarity constraint while $\hat{X}$ does not. We consider real and complex matrices for the initial input with and without the bias term. We put $r = 0.010$ and set $K = 10$ and $K' = 5$.

Next, we show the performance dependence of the three algorithms on their key parameters. We see the performance dependence of QCL on the number of layers L. The result is shown in Fig. S-68. We then see the performance dependence of the UKM on r, which is the coefficient of the second term in the right-hand side of Eq. (S-IX.1.8). The result is shown in Fig. S-69. In Fig. S-70, we show the performance dependence of the kernel method on λ, which is the coefficient of the second term in the right-hand side of Eq. (S-VI.2.4).

So far, we have used the squared error function $\ell_{\text{SE}}(\cdot, \cdot)$, Eq. (S-V.4.2). In Fig. S-71, we show the performance

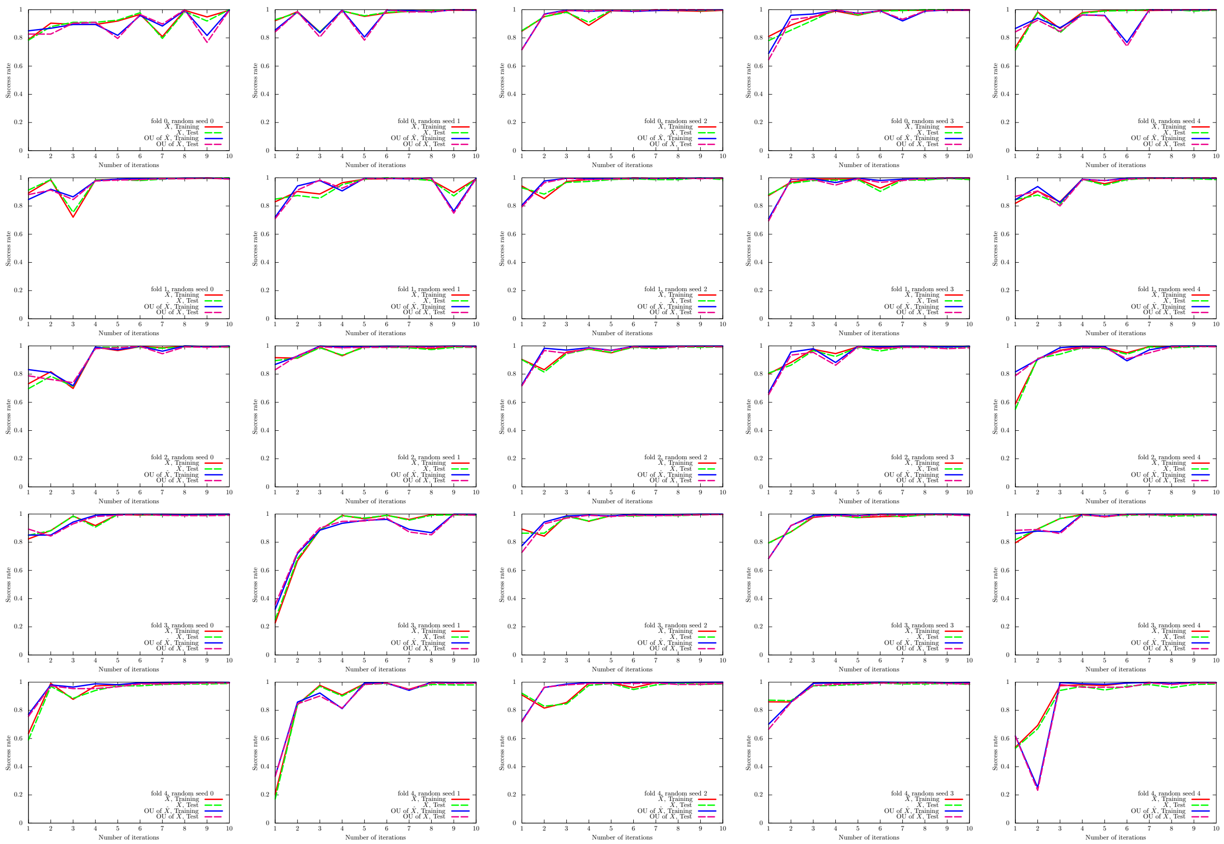

FIG. S-66: Results of the UKM ($\hat{X}$ and OU of $\hat{X}$) on the 5-fold datasets with 5 different random seeds for the semeion dataset (0 or non-0). We use complex matrices for the initial input and set $\theta_{\mathrm{bias}} = 0$. We set $r = 0.010$.

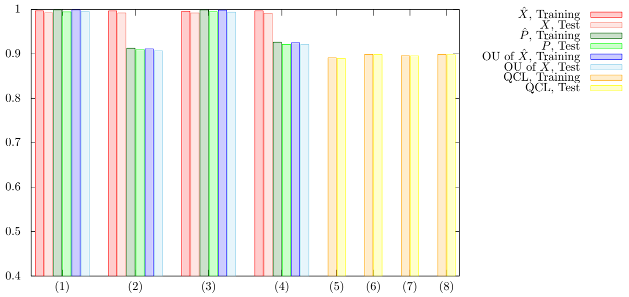

FIG. S-67: Results of 5-fold CV with 5 different random seeds for the semeion dataset (0 or non-0). For the UKM, we put $r = 0.010$ and set $K = 10$ and $K' = 5$. For QCL, the number of layers L is 5 and the number of iterations is 50. The numerical settings are as follows: (1) UKM: complex matrices without the bias term, (2) UKM: complex matrices with the bias term, (3) UKM: real matrices without the bias term, (4) UKM: real matrices with the bias term, (5) QCL: CNOT-based circuit without the bias term, (6) QCL: CNOT-based circuit with the bias term, (7) QCL: CRot-based circuit without the bias term, (8) QCL: CRot-based circuit with the bias term, (9) QCL: 1d Heisenberg circuit without the bias term, (10) QCL: 1d Heisenberg circuit with the bias term, (11) QCL: FC Heisenberg circuit without the bias term, and (12) QCL: FC Heisenberg circuit with the bias term.

dependence of QCL on the number of layers L in the case of the hinge function $\ell_{\mathrm{hinge}}(\cdot,\cdot)$, Eq. (S-V.4.3). In Fig. S-72, we show the performance dependence of the UKM on r, which is the coefficient of the second term in the right-hand side of Eq. (S-IX.1.8), in the case of the hinge function $\ell_{\mathrm{hinge}}(\cdot,\cdot)$, Eq. (S-V.4.3).

Algo.	Condition	Training	Test
Kernel method	Linear, w/o normalization, $\lambda = 10^{-2}$	0.9937	0.9887
Kernel method	Linear, w/o normalization, $\lambda = 10^{-1}$	0.9937	0.9887
Kernel method	Linear, w/o normalization, $\lambda = 1$	0.9937	0.9887
Kernel method	Linear, w/ normalization, $\lambda = 10^{-2}$	0.9936	0.9873
Kernel method	Linear, w/ normalization, $\lambda = 10^{-1}$	0.9934	0.9892
Kernel method	Linear, w/ normalization, $\lambda = 1$	0.9931	0.9898
Kernel method	Quadratic, w/o normalization, $\lambda = 10^{-2}$	1.0000	0.9956
Kernel method	Quadratic, w/o normalization, $\lambda = 10^{-1}$	1.0000	0.9956
Kernel method	Quadratic, w/o normalization, $\lambda = 1$	1.0000	0.9956
Kernel method	Quadratic, w/ normalization, $\lambda = 10^{-2}$	1.0000	0.9955
Kernel method	Quadratic, w/ normalization, $\lambda = 10^{-1}$	1.0000	0.9955
Kernel method	Quadratic, w/ normalization, $\lambda = 1$	0.9980	0.9949

TABLE S-26: Results of 5-fold CV with 5 different random seeds of the kernel method for the semeion dataset (0 or non-0). We set $\lambda = 10^{-2}, 10^{-1}, 1$. For $\phi(\cdot)$, we use linear and quadratic functions with and without normalization. We use the squared error function.

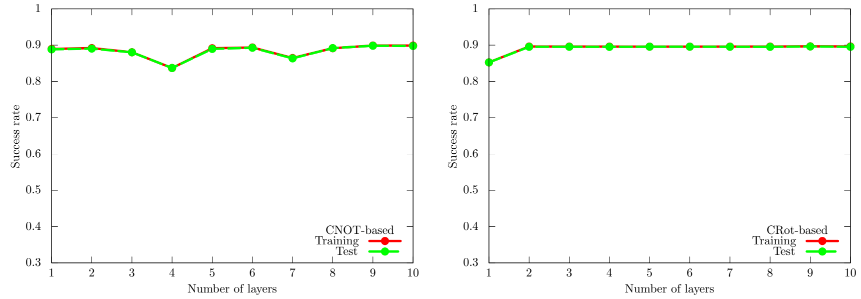

FIG. S-68: Performance dependence of QCL on the number of layers L for the semeion dataset (0 or non-0). We use the CNOT-based and CRot-based circuits. We set $\theta_{\text{bias}} = 0$. We iterate the computation 50 times.

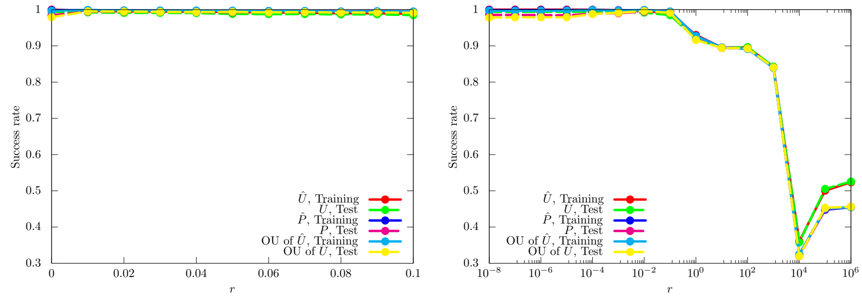

FIG. S-69: Performance dependence of the UKM on r, which is the coefficient of the second term in the right-hand side of Eq. (S-IX.1.8) for the semeion dataset (0 or non-0). We show the performance obtained by $\hat{X}$, $\hat{P}$, and OU of $\hat{X}$. Note that $\hat{P}$, and OU of $\hat{X}$ strictly satisfy the unitarity constraint while $\hat{X}$ does not. We use complex matrices for the initial input and set $\theta_{\text{bias}} = 0$. We set $K = 10$ and $K' = 5$.

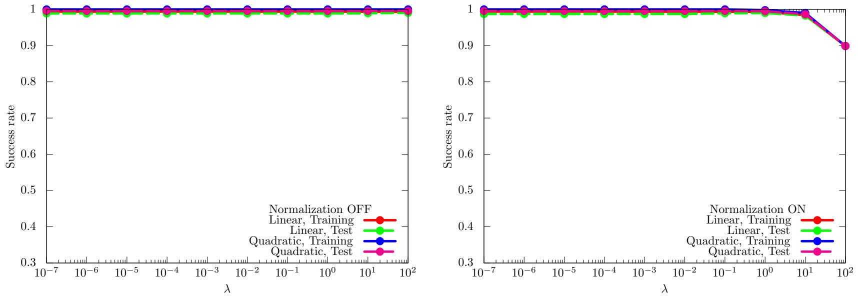

FIG. S-70: Performance dependence of the kernel method on λ, which is the coefficient of the second term in the right-hand side of Eq. (S-VI.2.4) for the semeion dataset (0 or non-0). For $\phi(\cdot)$ in Eq. (S-VI.2.1), we use linear and quadratic functions with and without normalization.

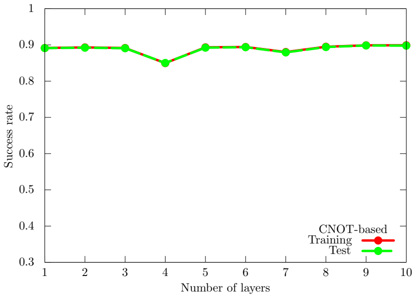

FIG. S-71: Performance dependence of QCL on the number of layers L for the semeion dataset (0 or non-0) in the case of the hinge function $\ell_{\mathrm{hinge}}(\cdot,\cdot)$, Eq. (S-V.4.3). We use the CNOT-based circuit. We set $\theta_{\mathrm{bias}} = 0$. We iterate the computation 300 times.

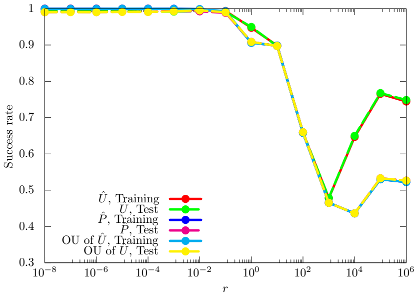

FIG. S-72: Performance dependence of the UKM on r, which is the coefficient of the second term in the right-hand side of Eq. (S-IX.1.8) for the semeion dataset (0 or non-0) in the case of the hinge function $\ell_{\mathrm{hinge}}(\cdot,\cdot)$, Eq. (S-V.4.3). We show the performance obtained by $\hat{X}$, $\hat{P}$, and OU of $\hat{X}$. Note that $\hat{P}$, and OU of $\hat{X}$ strictly satisfy the unitarity constraint while $\hat{X}$ does not. We use complex matrices for the initial input and set $\theta_{\mathrm{bias}} = 0$. We set $K = 30$ and $K' = 10$.

L. MNIST256 dataset (0 or 1)

We here show the numerical result for the MNIST256 dataset (0 or 1). For the UKM, we put $r = 0.010$ and set $K = 20$ and $K' = 10$ in Algo. S-10. For QCL, we run iterations 100 times. We use the squared error function $\ell_{\mathrm{SE}}(\cdot,\cdot)$, Eq. (S-V.4.2).

In Fig. S-73, we show the numerical results of QCL for the 5-fold datasets with 5 different random seeds. In Fig. S-74, we show the numerical results of $\hat{P}$ of the UKM for the 5-fold datasets with 5 different random seeds. In Fig. S-75, we also show the numerical results of OU of $\hat{X}$ of the UKM for the 5-fold datasets with 5 different random seeds.

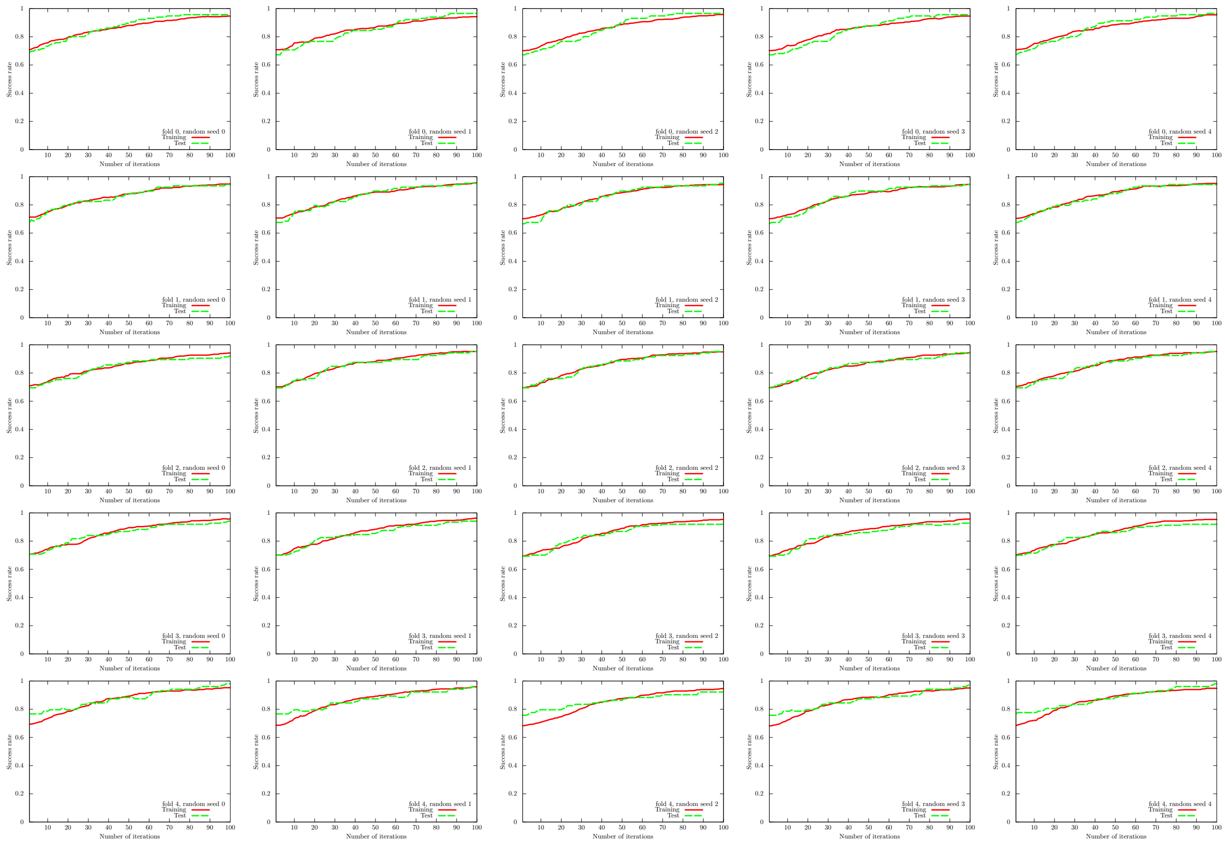

FIG. S-73: Results of QCL on the 5-fold datasets with 5 different random seeds for the MNIST256 dataset (0 or 1). We use the CNOT-based circuit and set $\theta_{\mathrm{bias}} = 0$. The number of layers L is set to 5.

We summarize the results of 5-fold CV with 5 different random seeds of QCL and the UKM in Tables S-27 and S-28, respectively. For QCL and the UKM, we select the best model for the training dataset over iterations to compute the performance. In Fig. S-76, we plot the data shown in Tables S-27 and S-28. We also summarize the results of 5-fold CV with 5 different random seeds of the kernel method in Table S-29. More specifically, we use Ridge classification, which is described in Sec. S-VI B. We consider linear and quadratic functions for $\phi(\cdot)$ in Eq. (S-VI.2.1) with and without normalization. We set $\lambda = 10^{-2}, 10^{-1}, 1$ where λ is the coefficient of the regularization term.

Algo.	Condition	Training	Test
QCL	CNOT-based, w/o bias	0.9511	0.9459
QCL	CNOT-based, w/ bias	0.9452	0.9413
QCL	CRot-based, w/o bias	0.7372	0.7326
QCL	CRot-based, w/ bias	0.7383	0.7273

TABLE S-27: Results of 5-fold CV with 5 different random seeds of QCL for the MNIST256 dataset (0 or 1). We consider four types of circuits with and without the bias term: the CNOT-based circuit, the CRot-based circuit, 1d Heisenberg circuit, and the FC Heisenberg circuit. The number of layers L is 5 and the number of iterations is 100.

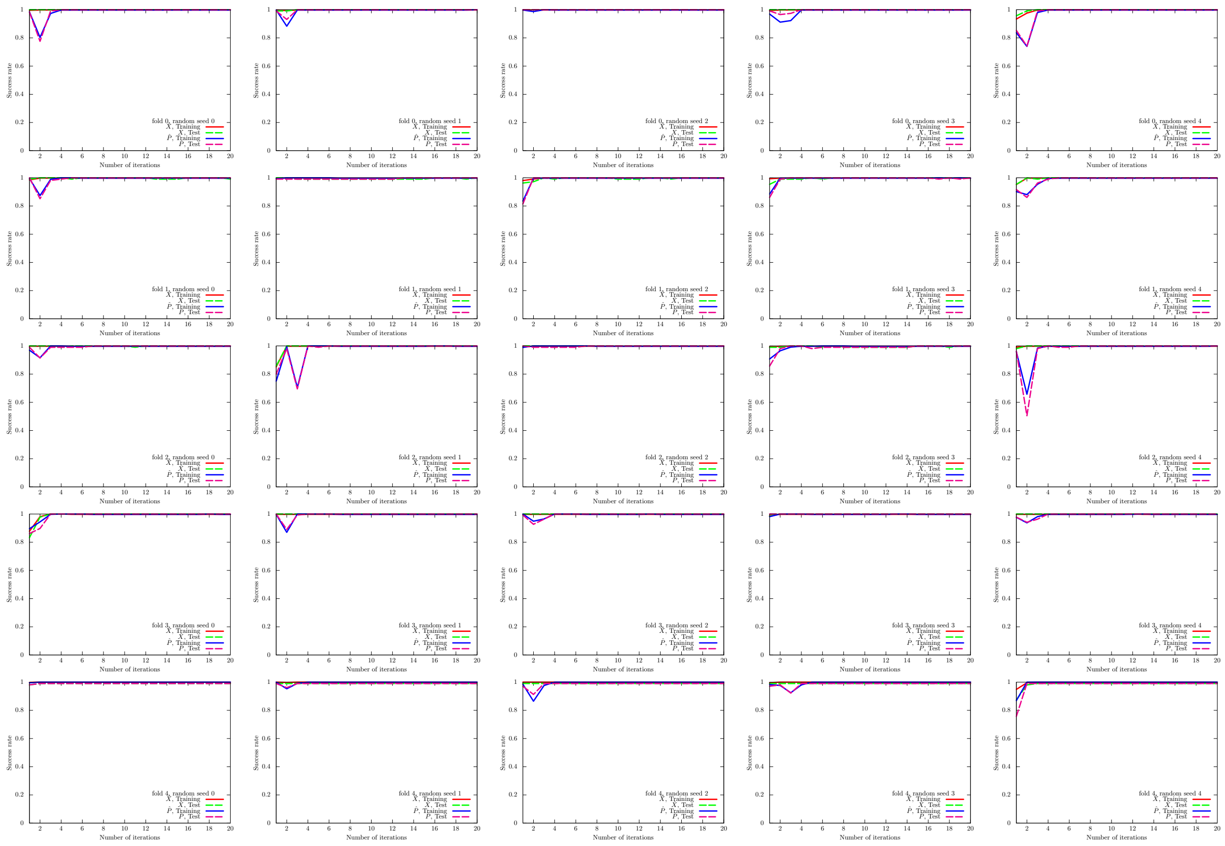

FIG. S-74: Results of the UKM ($\hat{X}$ and $\hat{P}$) on the 5-fold datasets with 5 different random seeds for the MNIST256 dataset (0 or 1). We use complex matrices for the initial input and set $\theta_{\text{bias}} = 0$. We set $r = 0.010$.

Algo.	Condition	Training	Test
UKM	$\hat{X}$, complex, w/o bias	0.9985	0.9966
UKM	$\hat{P}$, complex, w/o bias	0.9992	0.9949
UKM	OU of $\hat{X}$, complex, w/o bias	1.0	0.9951
UKM	$\hat{X}$, complex, w/ bias	0.9984	0.9951
UKM	$\hat{P}$, complex, w/ bias	1.0	0.9945
UKM	OU of $\hat{X}$, complex, w/ bias	1.0	0.9927
UKM	$\hat{X}$, real, w/o bias	0.9987	0.9954
UKM	$\hat{P}$, real, w/o bias	0.9991	0.9969
UKM	OU of $\hat{X}$, real, w/o bias	0.9997	0.9950
UKM	$\hat{X}$, real, w/ bias	0.9987	0.9962
UKM	$\hat{P}$, real, w/ bias	0.9997	0.9944
UKM	OU of $\hat{X}$, real, w/ bias	0.9997	0.9947

TABLE S-28: Results of 5-fold CV with 5 different random seeds of the UKM for the MNIST256 dataset (0 or 1). We show the performance obtained by $\hat{X}$, $\hat{P}$, and OU of $\hat{X}$. Note that $\hat{P}$, and OU of $\hat{X}$ strictly satisfy the unitarity constraint while $\hat{X}$ does not. We consider real and complex matrices for the initial input with and without the bias term. We put $r = 0.010$ and set $K = 20$ and $K' = 10$.

Next, we show the performance dependence of the three algorithms on their key parameters. We see the performance dependence of QCL on the number of layers L. The result is shown in Fig. S-77. We then see the performance dependence of the UKM on r, which is the coefficient of the second term in the right-hand side of Eq. (S-IX.1.8). The result is shown in Fig. S-78. In Fig. S-79, we show the performance dependence of the kernel method on λ, which is the coefficient of the second term in the right-hand side of Eq. (S-VI.2.4).

So far, we have used the squared error function $\ell_{\text{SE}}(\cdot,\cdot)$, Eq. (S-V.4.2). In Fig. S-80, we show the performance

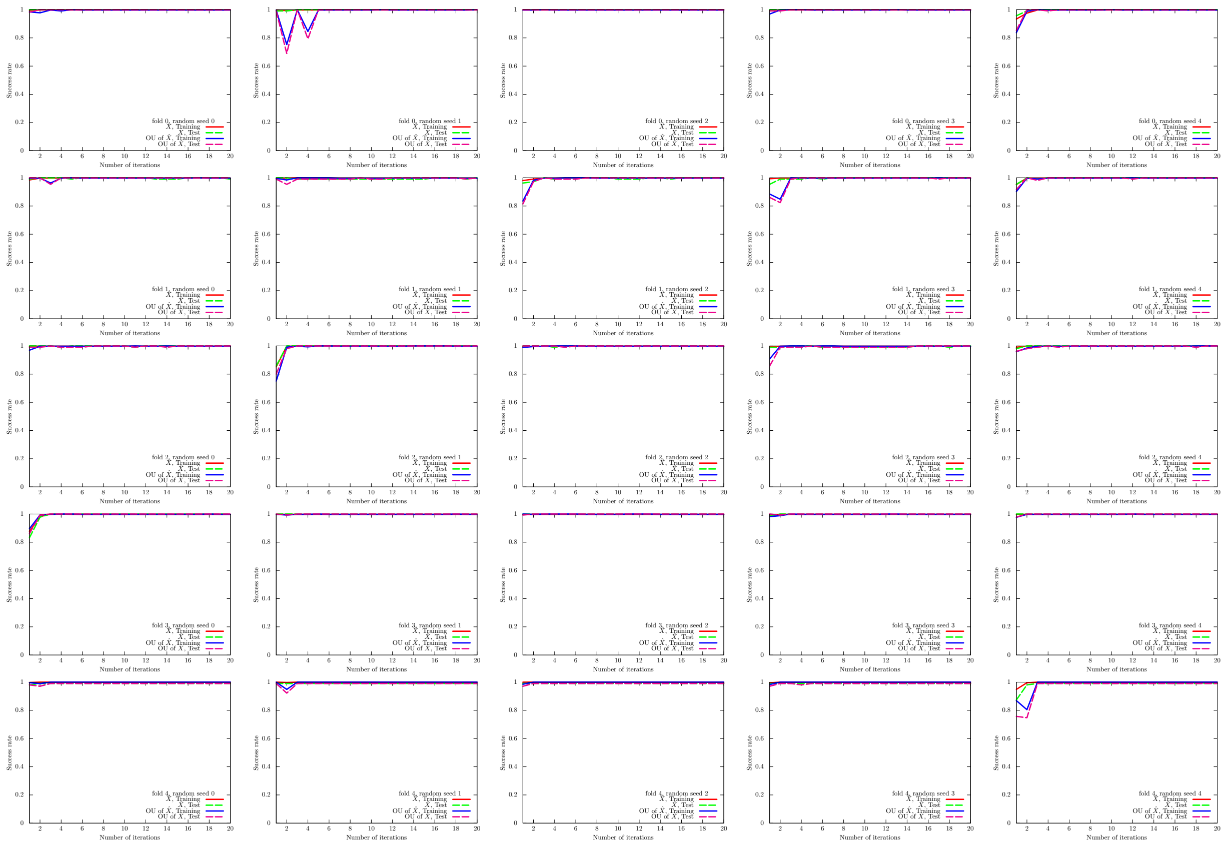

FIG. S-75: Results of the UKM ($\hat{X}$ and OU of $\hat{X}$) on the 5-fold datasets with 5 different random seeds for the MNIST256 dataset (0 or 1). We use complex matrices for the initial input and set $\theta_{\rm bias} = 0$. We set $r = 0.010$.

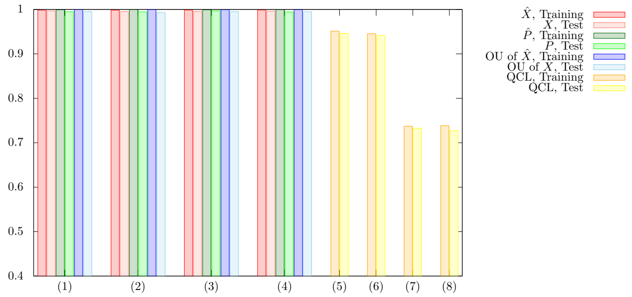

FIG. S-76: Results of 5-fold CV with 5 different random seeds for the MNIST256 dataset (0 or 1). For the UKM, we put $r = 0.010$ and set $K = 20$ and $K' = 10$. For QCL, the number of layers L is 5 and the number of iterations is 100. The numerical settings are as follows: (1) UKM: complex matrices without the bias term, (2) UKM: complex matrices with the bias term, (3) UKM: real matrices without the bias term, (4) UKM: real matrices with the bias term, (5) QCL: CNOT-based circuit without the bias term, (6) QCL: CNOT-based circuit with the bias term, (7) QCL: CRot-based circuit without the bias term, (8) QCL: CRot-based circuit with the bias term, (9) QCL: 1d Heisenberg circuit without the bias term, (10) QCL: 1d Heisenberg circuit with the bias term, (11) QCL: FC Heisenberg circuit without the bias term, and (12) QCL: FC Heisenberg circuit with the bias term.

dependence of QCL on the number of layers L in the case of the hinge function $\ell_{\rm hinge}(\cdot,\cdot)$, Eq. (S-V.4.3). In Fig. S-81, we show the performance dependence of the UKM on r, which is the coefficient of the second term in the right-hand side of Eq. (S-IX.1.8), in the case of the hinge function $\ell_{\rm hinge}(\cdot,\cdot)$, Eq. (S-V.4.3).

Algo.	Condition	Training	Test
Kernel method	Linear, w/o normalization, $\lambda = 10^{-2}$	1.0000	1.0000
Kernel method	Linear, w/o normalization, $\lambda = 10^{-1}$	1.0000	1.0000
Kernel method	Linear, w/o normalization, $\lambda = 1$	1.0000	1.0000
Kernel method	Linear, w/ normalization, $\lambda = 10^{-2}$	0.9991	0.9981
Kernel method	Linear, w/ normalization, $\lambda = 10^{-1}$	0.9982	0.9981
Kernel method	Linear, w/ normalization, $\lambda = 1$	0.9982	0.9981
Kernel method	Quadratic, w/o normalization, $\lambda = 10^{-2}$	1.0000	0.9897
Kernel method	Quadratic, w/o normalization, $\lambda = 10^{-1}$	1.0000	0.9897
Kernel method	Quadratic, w/o normalization, $\lambda = 1$	1.0000	0.9897
Kernel method	Quadratic, w/ normalization, $\lambda = 10^{-2}$	1.0000	0.9962
Kernel method	Quadratic, w/ normalization, $\lambda = 10^{-1}$	1.0000	0.9981
Kernel method	Quadratic, w/ normalization, $\lambda = 1$	0.9982	0.9981

TABLE S-29: Results of 5-fold CV with 5 different random seeds of the kernel method for the MNIST256 dataset (0 or 1). We set $\lambda = 10^{-2}, 10^{-1}, 1$. For $\phi(\cdot)$, we use linear and quadratic functions with and without normalization. We use the squared error function.

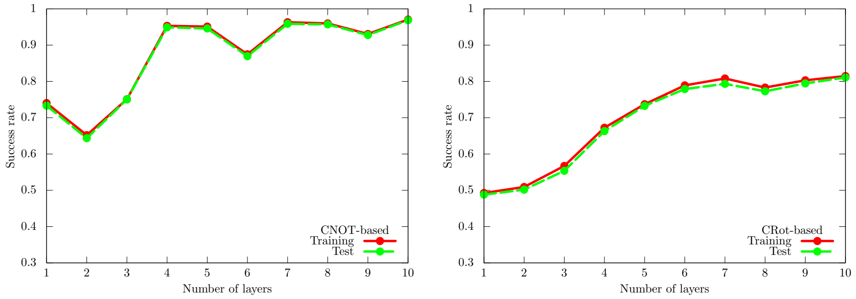

FIG. S-77: Performance dependence of QCL on the number of layers L for the MNIST256 dataset (0 or 1). We use the CNOT-based and CRot-based circuits. We set $\theta_{\mathrm{bias}} = 0$. We iterate the computation 100 times.

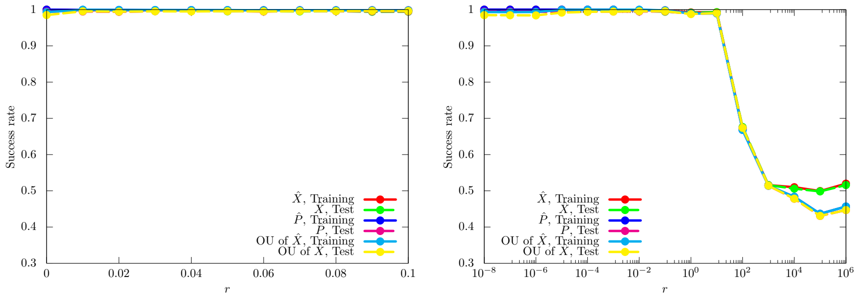

FIG. S-78: Performance dependence of the UKM on r, which is the coefficient of the second term in the right-hand side of Eq. (S-IX.1.8) for the MNIST256 dataset (0 or 1). We show the performance obtained by $\hat{X}$, $\hat{P}$, and OU of $\hat{X}$. Note that $\hat{P}$, and OU of $\hat{X}$ strictly satisfy the unitarity constraint while $\hat{X}$ does not. We use complex matrices for the initial input and set $\theta_{\mathrm{bias}} = 0$. We set $K = 20$ and $K' = 10$.

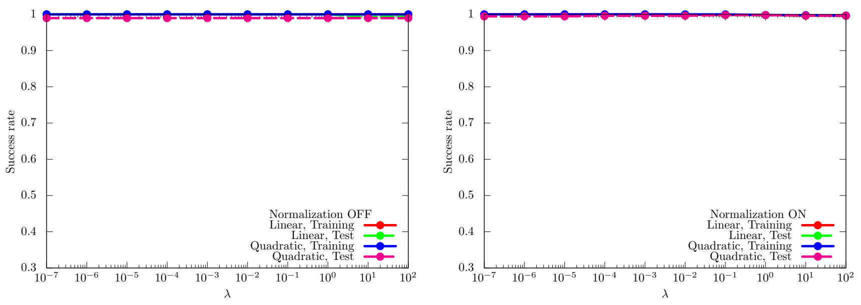

FIG. S-79: Performance dependence of the kernel method on λ, which is the coefficient of the second term in the right-hand side of Eq. (S-VI.2.4) for the MNIST256 dataset (0 or 1). For $\phi(\cdot)$ in Eq. (S-VI.2.1), we use linear and quadratic functions with and without normalization.

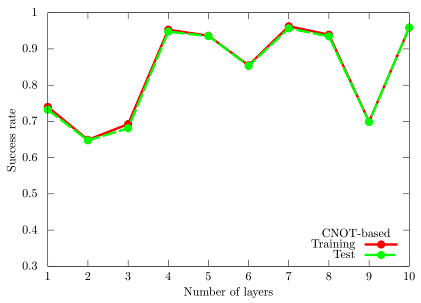

FIG. S-80: Performance dependence of QCL on the number of layers L for the MNIST256 dataset (0 or 1) in the case of the hinge function $\ell_{\text{hinge}}(\cdot,\cdot)$, Eq. (S-V.4.3). We use the CNOT-based circuit. We set $\theta_{\text{bias}} = 0$. We iterate the computation 300 times.

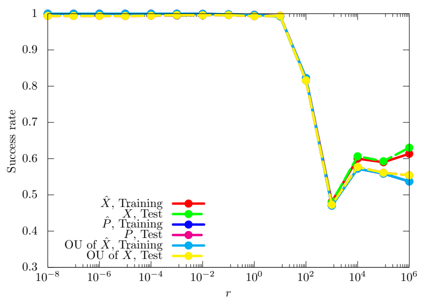

FIG. S-81: Performance dependence of the UKM on r, which is the coefficient of the second term in the right-hand side of Eq. (S-IX.1.8) for the MNIST256 dataset (0 or 1) in the case of the hinge function $\ell_{\text{hinge}}(\cdot,\cdot)$, Eq. (S-V.4.3). We show the performance obtained by $\hat{X}$, $\hat{P}$, and OU of $\hat{X}$. Note that $\hat{P}$, and OU of $\hat{X}$ strictly satisfy the unitarity constraint while $\hat{X}$ does not. We use complex matrices for the initial input and set $\theta_{\text{bias}} = 0$. We set $K = 30$ and $K' = 10$.

M. MNIST256 dataset (0 or non-0)

We here show the numerical result for the MNIST256 dataset (0 or non-0). For the UKM, we put $r = 0.010$ and set $K = 10$ and $K' = 5$ in Algo. S-10. For QCL, we run iterations 50 times. We use the squared error function $\ell_{\mathrm{SE}}(\cdot,\cdot)$, Eq. (S-V.4.2).

In Fig. S-82, we show the numerical results of QCL for the 5-fold datasets with 5 different random seeds. In Fig. S-83, we show the numerical results of $\hat{P}$ of the UKM for the 5-fold datasets with 5 different random seeds. In Fig. S-84, we also show the numerical results of OU of $\hat{X}$ of the UKM for the 5-fold datasets with 5 different random seeds.

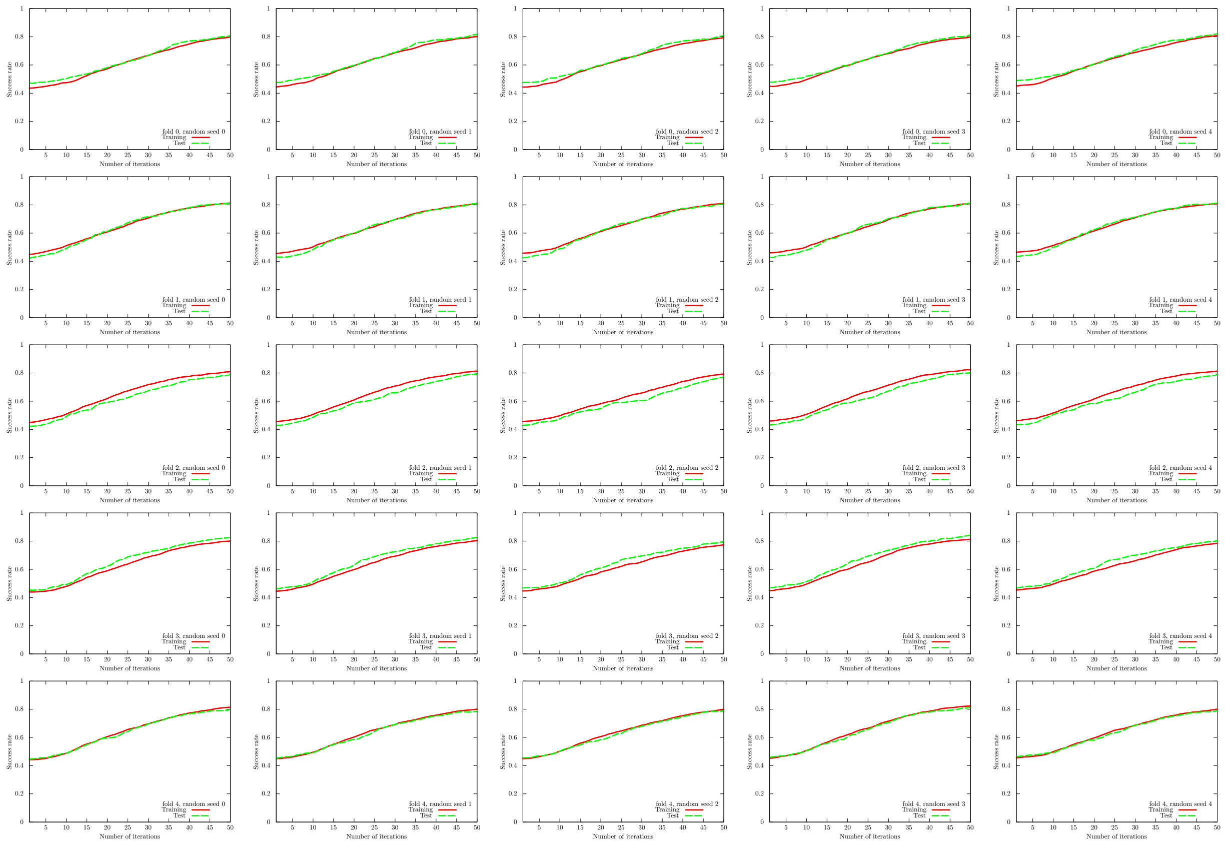

FIG. S-82: Results of QCL on the 5-fold datasets with 5 different random seeds for the MNIST256 dataset (0 or non-0). We use the CNOT-based circuit and set $\theta_{\mathrm{bias}} = 0$. The number of layers L is set to 5.

We summarize the results of 5-fold CV with 5 different random seeds of QCL and the UKM in Tables S-30 and S-31, respectively. For QCL and the UKM, we select the best model for the training dataset over iterations to compute the performance. In Fig. S-85, we plot the data shown in Tables S-30 and S-31. We also summarize the results of 5-fold CV with 5 different random seeds of the kernel method in Table S-32. More specifically, we use Ridge classification, which is described in Sec. S-VI B. We consider linear and quadratic functions for $\phi(\cdot)$ in Eq. (S-VI.2.1) with and without normalization. We set $\lambda = 10^{-2}, 10^{-1}, 1$ where λ is the coefficient of the regularization term.

Algo.	Condition	Training	Test
QCL	CNOT-based, w/o bias	0.8018	0.8006
QCL	CNOT-based, w/ bias	0.9053	0.9050
QCL	CRot-based, w/o bias	0.9010	0.9002
QCL	CRot-based, w/ bias	0.9053	0.9048

TABLE S-30: Results of 5-fold CV with 5 different random seeds of QCL for the MNIST256 dataset (0 or non-0). We consider four types of circuits with and without the bias term: the CNOT-based circuit, the CRot-based circuit, 1d Heisenberg circuit, and the FC Heisenberg circuit. The number of layers L is 5 and the number of iterations is 50.

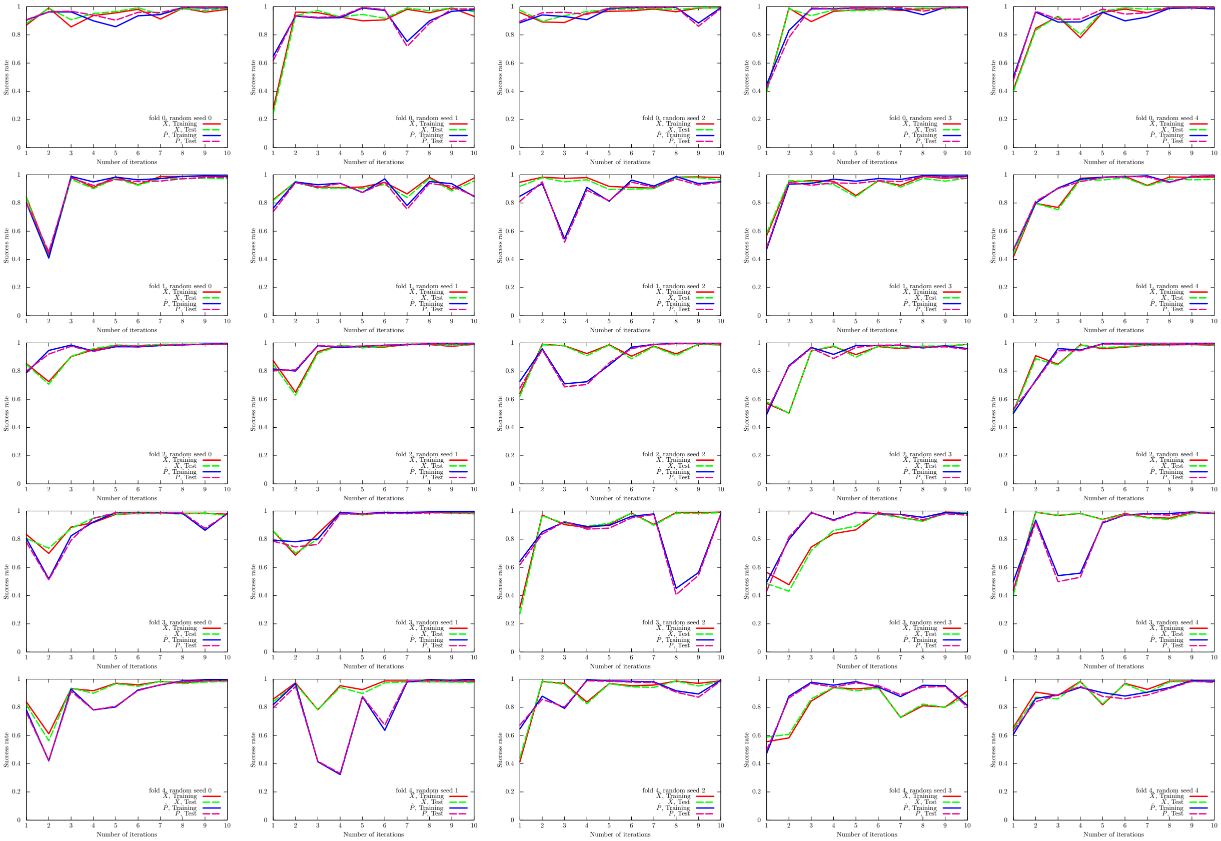

FIG. S-83: Results of the UKM ($\hat{X}$ and $\hat{P}$) on the 5-fold datasets with 5 different random seeds for the MNIST256 dataset (0 or non-0). We use complex matrices for the initial input and set $\theta_{\text{bias}} = 0$. We set $r = 0.010$.

Algo.	Condition	Training	Test
UKM	$\hat{X}$, complex, w/o bias	0.9868	0.9829
UKM	$\hat{P}$, complex, w/o bias	0.9911	0.9870
UKM	OU of $\hat{X}$, complex, w/o bias	0.9915	0.9848
UKM	$\hat{X}$, complex, w/ bias	0.9902	0.9856
UKM	$\hat{P}$, complex, w/ bias	0.9238	0.9199
UKM	OU of $\hat{X}$, complex, w/ bias	0.9264	0.9244
UKM	$\hat{X}$, real, w/o bias	0.9886	0.9836
UKM	$\hat{P}$, real, w/o bias	0.9922	0.9871
UKM	OU of $\hat{X}$, real, w/o bias	0.9927	0.9889
UKM	$\hat{X}$, real, w/ bias	0.9894	0.9859
UKM	$\hat{P}$, real, w/ bias	0.9213	0.9188
UKM	OU of $\hat{X}$, real, w/ bias	0.9208	0.9205

TABLE S-31: Results of 5-fold CV with 5 different random seeds of the UKM for the MNIST256 dataset (0 or non-0). We show the performance obtained by $\hat{X}$, $\hat{P}$, and OU of $\hat{X}$. Note that $\hat{P}$, and OU of $\hat{X}$ strictly satisfy the unitarity constraint while $\hat{X}$ does not. We consider real and complex matrices for the initial input with and without the bias term. We put $r = 0.010$ and set $K = 10$ and $K' = 5$.

Next, we show the performance dependence of the three algorithms on their key parameters. We see the performance dependence of QCL on the number of layers L. The result is shown in Fig. S-86. We then see the performance dependence of the UKM on r, which is the coefficient of the second term in the right-hand side of Eq. (S-IX.1.8). The result is shown in Fig. S-87. In Fig. S-88, we show the performance dependence of the kernel method on λ, which is the coefficient of the second term in the right-hand side of Eq. (S-VI.2.4).

So far, we have used the squared error function $\ell_{\text{SE}}(\cdot,\cdot)$, Eq. (S-V.4.2). In Fig. S-89, we show the performance

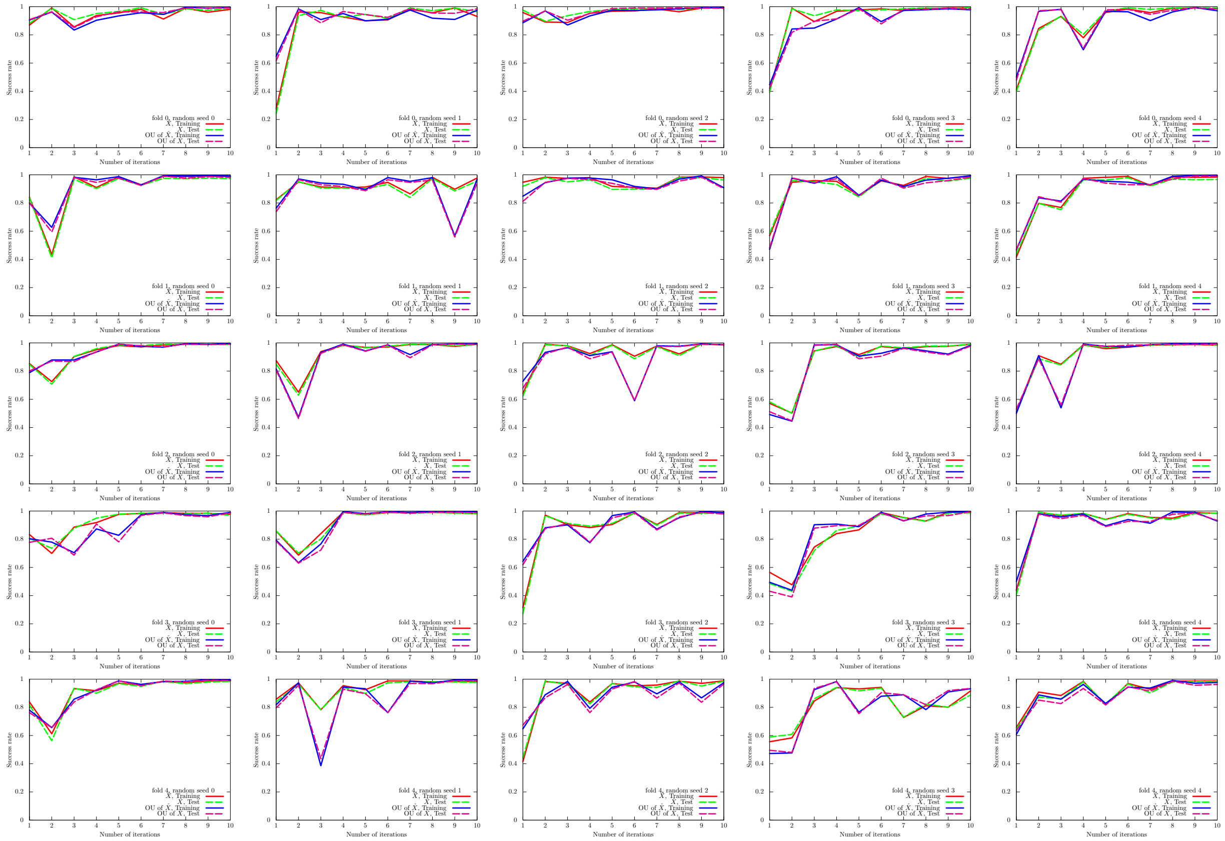

FIG. S-84: Results of the UKM ($\hat{X}$ and OU of $\hat{X}$) on the 5-fold datasets with 5 different random seeds for the MNIST256 dataset (0 or non-0). We use complex matrices for the initial input and set $\theta_{\text{bias}} = 0$. We set $r = 0.010$.

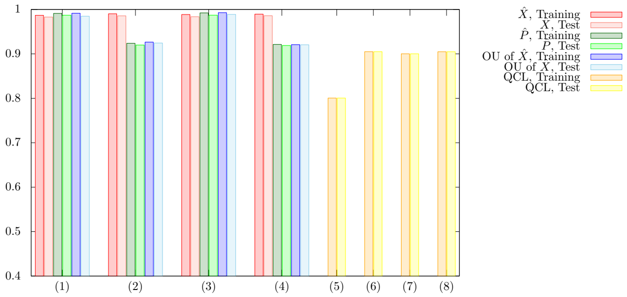

FIG. S-85: Results of 5-fold CV with 5 different random seeds for the MNIST256 dataset (0 or non-0). For the UKM, we put $r = 0.010$ and set $K = 10$ and $K' = 5$. For QCL, the number of layers L is 5 and the number of iterations is 50. The numerical settings are as follows: (1) UKM: complex matrices without the bias term, (2) UKM: complex matrices with the bias term, (3) UKM: real matrices without the bias term, (4) UKM: real matrices with the bias term, (5) QCL: CNOT-based circuit without the bias term, (6) QCL: CNOT-based circuit with the bias term, (7) QCL: CRot-based circuit without the bias term, (8) QCL: CRot-based circuit with the bias term, (9) QCL: 1d Heisenberg circuit without the bias term, (10) QCL: 1d Heisenberg circuit with the bias term, (11) QCL: FC Heisenberg circuit without the bias term, and (12) QCL: FC Heisenberg circuit with the bias term.

dependence of QCL on the number of layers L in the case of the hinge function $\ell_{\text{hinge}}(\cdot,\cdot)$, Eq. (S-V.4.3). In Fig. S-90, we show the performance dependence of the UKM on r, which is the coefficient of the second term in the right-hand side of Eq. (S-IX.1.8), in the case of the hinge function $\ell_{\text{hinge}}(\cdot,\cdot)$, Eq. (S-V.4.3).

Algo.	Condition	Training	Test
Kernel method	Linear, w/o normalization, $\lambda = 10^{-2}$	0.9963	0.9921
Kernel method	Linear, w/o normalization, $\lambda = 10^{-1}$	0.9963	0.9921
Kernel method	Linear, w/o normalization, $\lambda = 1$	0.9963	0.9921
Kernel method	Linear, w/ normalization, $\lambda = 10^{-2}$	0.9893	0.98189
Kernel method	Linear, w/ normalization, $\lambda = 10^{-1}$	0.9856	0.9792
Kernel method	Linear, w/ normalization, $\lambda = 1$	0.9843	0.9787
Kernel method	Quadratic, w/o normalization, $\lambda = 10^{-2}$	1.0000	0.9906
Kernel method	Quadratic, w/o normalization, $\lambda = 10^{-1}$	1.0000	0.9906
Kernel method	Quadratic, w/o normalization, $\lambda = 1$	1.0000	0.9906
Kernel method	Quadratic, w/ normalization, $\lambda = 10^{-2}$	0.9997	0.9949
Kernel method	Quadratic, w/ normalization, $\lambda = 10^{-1}$	0.9992	0.9953
Kernel method	Quadratic, w/ normalization, $\lambda = 1$	0.9946	0.9916

TABLE S-32: Results of 5-fold CV with 5 different random seeds of the kernel method for the MNIST256 dataset (0 or non-0). We set $\lambda = 10^{-2}, 10^{-1}, 1$. For $\phi(\cdot)$, we use linear and quadratic functions with and without normalization. We use the squared error function.

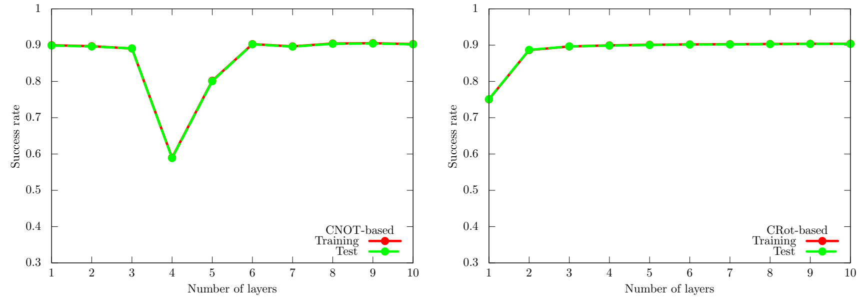

FIG. S-86: Performance dependence of QCL on the number of layers L for the MNIST256 dataset (0 or non-0). We use the CNOT-based and CRot-based circuits. We set $\theta_{\text{bias}} = 0$. We iterate the computation 50 times.

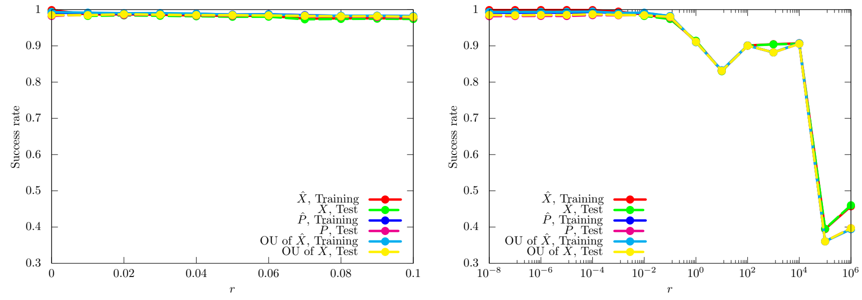

FIG. S-87: Performance dependence of the UKM on r, which is the coefficient of the second term in the right-hand side of Eq. (S-IX.1.8) for the MNIST256 dataset (0 or non-0). We show the performance obtained by $\hat{X}$, $\hat{P}$, and OU of $\hat{X}$. Note that $\hat{P}$, and OU of $\hat{X}$ strictly satisfy the unitarity constraint while $\hat{X}$ does not. We use complex matrices for the initial input and set $\theta_{\text{bias}} = 0$. We set $K = 10$ and $K' = 5$.

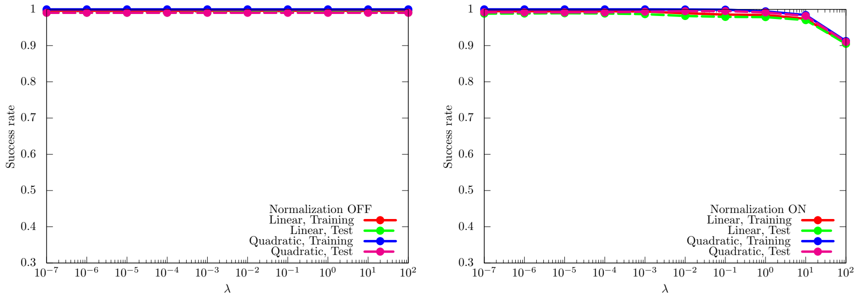

FIG. S-88: Performance dependence of the kernel method on λ, which is the coefficient of the second term in the right-hand side of Eq. (S-VI.2.4) for the MNIST256 dataset (0 or non-0). For $\phi(\cdot)$ in Eq. (S-VI.2.1), we use linear and quadratic functions with and without normalization.

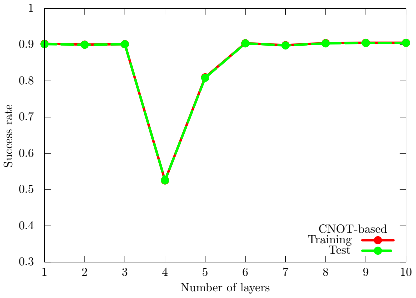

FIG. S-89: Performance dependence of QCL on the number of layers L for the MNIST256 dataset (0 or non-0) in the case of the hinge function $\ell_{\text{hinge}}(\cdot,\cdot)$, Eq. (S-V.4.3). We use the CNOT-based circuit. We set $\theta_{\text{bias}} = 0$. We iterate the computation 300 times.

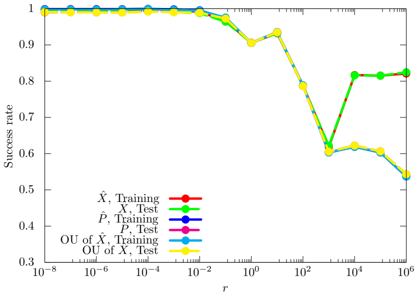

FIG. S-90: Performance dependence of the UKM on r, which is the coefficient of the second term in the right-hand side of Eq. (S-IX.1.8) for the MNIST256 dataset (0 or non-0) in the case of the hinge function $\ell_{\text{hinge}}(\cdot,\cdot)$, Eq. (S-V.4.3). We show the performance obtained by $\hat{X}$, $\hat{P}$, and OU of $\hat{X}$. Note that $\hat{P}$, and OU of $\hat{X}$ strictly satisfy the unitarity constraint while $\hat{X}$ does not. We use complex matrices for the initial input and set $\theta_{\text{bias}} = 0$. We set $K = 30$ and $K' = 10$.

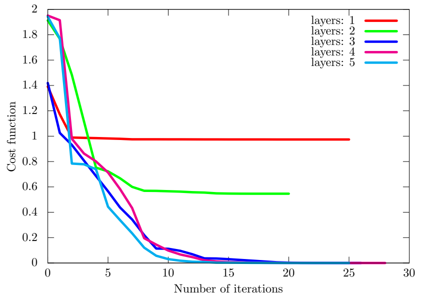

FIG. S-91: Values of the cost function $\mathcal{J}_{\mathrm{cost}}(\theta, \lambda; L, 2, \hat{U})$, Eq. (S-X.1.6) with $p = 2$, for the iris dataset (1 or non-1).

N. Discussions

In the main text, we give the discussions on the numerical results. The results shown in this section are basically consistent with the results shown in the main text and support the statement.

S-XII. NUMERICAL SIMULATION OF THE VCR

In this section, we show numerical simulation on the VCR. For $\hat{U}_{\mathrm{c}}(\theta)$, we use the CNOT-based circuit. Refer to Sec. S-V B for details.

A. Numerical setting

In this section, we use Eq. (S-X.1.6) with $p = 2$ as the optimization function of the VCR. For $\hat{U}_{\mathrm{c}}(\theta)$, we use the CNOT-based circuit.

B. Datasets

In this section, we consider the iris dataset (1 or non-1), the cancer dataset (0 or 1), and the wine dataset (0 or non-0) described in Sec. S-XI B. Refer to Sec. S-XI B for the details.

C. Iris dataset (1 or non-1)

Let us consider the iris dataset (1 or non-1) and minimizing Eq. (S-X.1.6) with $p = 2$. As $\hat{U}$, we use $\hat{P}$ computed by the UKM under the condition of real matrices, $r = 0.010$, and $\theta_{\mathrm{b}} = 0$ that gives the success rate for the training dataset 0.7565 and that for the test dataset 0.8286. In Fig. S-91, we show the values of the cost function $\mathcal{J}_{\mathrm{cost}}(\theta, \lambda; L, 2, \hat{U})$, Eq. (S-X.1.6) with $p = 2$. We vary the number of layers L. In Table S-33, we summarize the performance of the input unitary operator, QCL, and the circuit geometries computed by the VCR. We have $L_{0.001} = 3$.

D. Cancer dataset (0 or 1)

Let us consider the cancer dataset (0 or 1) and minimizing Eq. (S-X.1.6) with $p = 2$. As $\hat{U}$, we use $\hat{P}$ computed by the UKM under the condition of real matrices, $r = 0.010$, and $\theta_{\mathrm{b}} = 0$ that gives the success rate for the training dataset 0.9139 and that for the test dataset 0.9483. In Fig. S-92, we show the values of the cost function $\mathcal{J}_{\mathrm{cost}}(\theta, \lambda; L, 2, \hat{U})$, Eq. (S-X.1.6) with $p = 2$. We vary the number of layers L. In Table S-34, we summarize the performance of the input unitary operator, QCL, and the circuit geometries computed by the VCR. We have $L_{0.001} = 80$.

Algo.	Condition	Cost	Training	Test
Input	UKM, $\hat{P}$, real, w/o bias	—	0.7565	0.8286
VCR	# of layers: 1	0.9740	0.3565	0.2571
VCR	# of layers: 2	0.5465	0.3565	0.2571
VCR	# of layers: 3	0.0000	0.7565	0.8286
VCR	# of layers: 4	0.0000	0.7565	0.8286
VCR	# of layers: 5	0.0000	0.7565	0.8286
UKM	$\hat{P}$, real, w/o bias	—	0.7880	0.7789
QCL	# of layers: 5	—	0.6851	0.5845

TABLE S-33: Performance of the VCR for the iris dataset (1 or non-1). We show the success rates for the training and test datasets and the values of the cost function $\mathcal{J}_{\text{cost}}(\theta, \lambda; L, 2, \hat{U})$, Eq. (S-X.1.6) with $p = 2$. The input for the VCR is $\hat{P}$ created by the UKM under the condition of real matrices, $r = 0.010$, and $\theta_{\text{b}} = 0$. For reference, we add the last two rows that show the results of 5-fold CV.

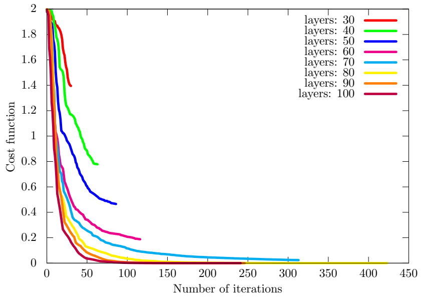

FIG. S-92: Values of the cost function $\mathcal{J}_{\text{cost}}(\theta, \lambda; L, 2, \hat{U})$, Eq. (S-X.1.6) with $p = 2$, for the cancer dataset (0 or 1).

E. Wine dataset (0 or non-0)

Let us consider the wine dataset (0 or non-0) and minimizing Eq. (S-X.1.6) with $p = 2$. As $\hat{U}$, we use $\hat{P}$ computed by the UKM under the condition of real matrices, $r = 0.010$, and $\theta_{\text{b}} = 0$ that gives the success rate for the training dataset 0.9179 and that for the test dataset 0.9091. In Fig. S-93, we show the values of the cost function $\mathcal{J}_{\text{cost}}(\theta, \lambda; L, 2, \hat{U})$, Eq. (S-X.1.6) with $p = 2$. We vary the number of layers L. In Table S-35, we summarize the performance of the input unitary operator, QCL, and the circuit geometries computed by the VCR. We have $L_{0.001} = 25$.

Algo.	Condition	Cost	Training	Test
Input	UKM, $\hat{P}$, real, w/o bias	—	0.9139	0.9483
VCR	# of layers: 10	1.9694	0.3929	0.2931
VCR	# of layers: 20	1.9734	0.6071	0.7069
VCR	# of layers: 30	1.3950	0.6071	0.7069
VCR	# of layers: 40	0.7777	0.6909	0.7586
VCR	# of layers: 50	0.4657	0.8499	0.9224
VCR	# of layers: 60	0.1877	0.9073	0.9483
VCR	# of layers: 70	0.0236	0.9073	0.9483
VCR	# of layers: 80	0.0000	0.9139	0.9483
VCR	# of layers: 90	0.0000	0.9139	0.9483
VCR	# of layers: 100	0.0000	0.9139	0.9483
UKM	$\hat{P}$, real, w/o bias	—	0.9194	0.9131
QCL	# of layers: 5	—	0.8798	0.8768
QCL	# of layers: 10	—	0.7814	0.7767

TABLE S-34: Performance of the VCR for the cancer dataset (0 or 1). We show the success rates for the training and test datasets and the values of the cost function $\mathcal{J}_{\text{cost}}(\theta, \lambda; L, 2, \hat{U})$, Eq. (S-X.1.6) with $p = 2$. The input for the VCR is $\hat{P}$ created by the UKM under the condition of real matrices, $r = 0.010$, and $\theta_{\text{b}} = 0$. For reference, we add the last three rows that show the results of 5-fold CV.

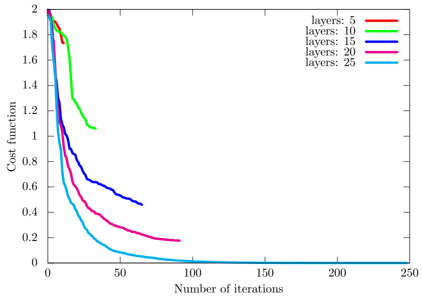

FIG. S-93: Values of the cost function $\mathcal{J}_{\rm cost}(\theta, \lambda; L, 2, \hat{U})$, Eq. (S-X.1.6) with $p = 2$, for the wine dataset (0 or non-0).

Algo.	Condition	Cost	Training	Test
Input	UKM, $\hat{P}$, real, w/o bias	—	0.9179	0.9091
VCR	# of layers: 5	1.7343	0.6642	0.6818
VCR	# of layers: 10	1.0591	0.4254	0.4091
VCR	# of layers: 15	0.4588	0.6791	0.7045
VCR	# of layers: 20	0.1765	0.7836	0.8864
VCR	# of layers: 25	0.0000	0.9179	0.9091
UKM	$\hat{P}$, real, w/o bias	—	0.9200	0.9185
QCL	# of layers: 5	—	0.9057	0.9052

TABLE S-35: Performance of the VCR for the wine dataset (0 or non-0). We show the success rates for the training and test datasets and the values of the cost function $\mathcal{J}_{\rm cost}(\theta, \lambda; L, 2, \hat{U})$, Eq. (S-X.1.6) with $p = 2$. The input for the VCR is $\hat{P}$ created by the UKM under the condition of real matrices, $r = 0.010$, and $\theta_{\rm b} = 0$. For reference, we add the last two rows that show the results of 5-fold CV.

F. Discussions

The discussions on the VCR is given in the main text. In this section, we give the results of the VCR on three different datasets: the iris dataset (1 or non-1), the cancer dataset (0 or 1), and the wine dataset (0 or non-0). The results shown in this section support the statement in the main text.

S-XIII. CONCLUSION

In this SM, we have provided supplemental material on "Ansatz-Independent Variational Quantum Classifier."

[1] K. Petersen and M. Pedersen, Technical University of Denmark **15** (2008).
[2] K. Kreutz-Delgado, arXiv preprint arXiv:0906.4835 (2009).
[3] S. Boyd, S. P. Boyd, and L. Vandenberghe, *Convex optimization* (Cambridge university press, 2004).
[4] R. Fletcher, *Practical methods of optimization* (John Wiley & Sons, 2013).
[5] R. Fletcher and C. M. Reeves, The computer journal **7**, 149 (1964).
[6] J. R. Shewchuk *et al.*, "An introduction to the conjugate gradient method without the agonizing pain," (1994).
[7] C. G. Broyden, IMA Journal of Applied Mathematics **6**, 76 (1970).
[8] R. Fletcher, The computer journal **13**, 317 (1970).
[9] D. Goldfarb, Mathematics of computation **24**, 23 (1970).
[10] D. F. Shanno, Mathematics of computation **24**, 647 (1970).
[11] L. Armijo, Pacific Journal of mathematics **16**, 1 (1966).
[12] P. Wolfe, SIAM review **11**, 226 (1969).
[13] P. Wolfe, SIAM review **13**, 185 (1971).
[14] E. Polak and G. Ribiere, ESAIM: Mathematical Modelling and Numerical Analysis-Modélisation Mathématique et Analyse Numérique **3**, 35 (1969).
[15] M. R. Hestenes, E. Stiefel, *et al.*, Journal of research of the National Bureau of Standards **49**, 409 (1952).
[16] Y.-H. Dai and Y.-x. Yuan, Annals of Operations Research **103**, 33 (2001).

[17] M. Schuld, A. Bocharov, K. M. Svore, and N. Wiebe, Physical Review A **101**, 032308 (2020).
[18] K. Mitarai, M. Negoro, M. Kitagawa, and K. Fujii, Physical Review A **98**, 032309 (2018).
[19] E. Farhi, J. Goldstone, and S. Gutmann, arXiv preprint arXiv:1411.4028 (2014).
[20] J. R. McClean, J. Romero, R. Babbush, and A. Aspuru-Guzik, New Journal of Physics **18**, 023023 (2016).
[21] M. Plesch and Č. Brukner, Physical Review A **83**, 032302 (2011).
[22] M. Schuld, arXiv preprint arXiv:2101.11020 (2021).
[23] F. Arute, K. Arya, R. Babbush, D. Bacon, J. C. Bardin, R. Barends, R. Biswas, S. Boixo, F. G. Brandao, D. A. Buell, *et al.*, Nature **574**, 505 (2019).
[24] M. Schuld, R. Sweke, and J. J. Meyer, arXiv preprint arXiv:2008.08605 (2020).
[25] A. Barenco, C. H. Bennett, R. Cleve, D. P. DiVincenzo, N. Margolus, P. Shor, T. Sleator, J. A. Smolin, and H. Weinfurter, Physical review A **52**, 3457 (1995).
[26] M. A. Nielsen and I. Chuang, *Quantum computation and quantum information* (American Association of Physics Teachers, 2002).
[27] D. P. DiVincenzo, Physical Review A **51**, 1015 (1995).
[28] K. Fujii and K. Nakajima, Physical Review Applied **8**, 024030 (2017).
[29] K. Nakajima, K. Fujii, M. Negoro, K. Mitarai, and M. Kitagawa, Physical Review Applied **11**, 034021 (2019).
[30] C. M. Bishop, *Pattern recognition and machine learning* (springer, 2006).
[31] K. P. Murphy, *Machine learning: a probabilistic perspective* (MIT press, 2012).
[32] R. Lai and S. Osher, Journal of Scientific Computing **58**, 431 (2014).
[33] S. Osher, M. Burger, D. Goldfarb, J. Xu, and W. Yin, Multiscale Modeling & Simulation **4**, 460 (2005).
[34] W. Yin, S. Osher, D. Goldfarb, and J. Darbon, SIAM Journal on Imaging sciences **1**, 143 (2008).
[35] R. T. Rockafellar, *Convex analysis*, 28 (Princeton university press, 1970).
[36] L. M. Bregman, USSR computational mathematics and mathematical physics **7**, 200 (1967).
[37] E. T. Hale, W. Yin, and Y. Zhang, *A fixed-point continuation method for L_1-regularization with application to compressed sensing*, Tech. Rep. (2007).
[38] J. H. Manton, IEEE Transactions on Signal Processing **50**, 635 (2002).
[39] W. Gibson, Psychometrika **27**, 193 (1962).
[40] M. Schuld and N. Killoran, Physical review letters **122**, 040504 (2019).
[41] A. Vijaykumar, A. P. Bardelli, A. Rothberg, A. Hilboll, A. Kloeckner, A. Scopatz, A. Lee, A. Rokem, C. N. Woods, C. Fulton, C. Masson, C. Häggström, C. Fitzgerald, D. A. Nicholson, D. R. Hagen, D. V. Pasechnik, E. Olivetti, E. Martin, E. Wieser, F. Silva, F. Lenders, F. Wilhelm, G. Young, G. A. Price, G.-L. Ingold, G. E. Allen, G. R. Lee, H. Audren, I. Probst, J. P. Dietrich, J. Silterra, J. T. Webber, J. Slavič, J. Nothman, J. Buchner, J. Kulick, J. L. Schönberger, J. V. de Miranda Cardoso, J. Reimer, J. Harrington, J. L. C. Rodríguez, J. Nunez-Iglesias, J. Kuczynski, K. Tritz, M. Thoma, M. Newville, M. Kümmerer, M. Bolingbroke, M. Tartre, M. Pak, N. J. Smith, N. Nowaczyk, N. Shebanov, O. Pavlyk, P. A. Brodtkorb, P. Lee, R. T. McGibbon, R. Feldbauer, S. Lewis, S. Tygier, S. Sievert, S. Vigna, S. Peterson, S. More, T. Pudlik, T. Oshima, T. J. Pingel, T. P. Robitaille, T. Spura, T. R. Jones, T. Cera, T. Leslie, T. Zito, T. Krauss, U. Upadhyay, Y. O. Halchenko, and Y. Vázquez-Baeza, Nature Methods **17**, 261 (2020).
[42] V. V. Shende, S. S. Bullock, and I. L. Markov, IEEE Transactions on Computer-Aided Design of Integrated Circuits and Systems **25**, 1000 (2006).
[43] E. Knill, arXiv preprint quant-ph/9508006 (1995).
[44] R. Iten, R. Colbeck, I. Kukuljan, J. Home, and M. Christandl, Physical Review A **93**, 032318 (2016).
[45] V. Bergholm *et al.*, arXiv preprint arXiv:1811.04968 (2018).
[46] F. Pedregosa, G. Varoquaux, A. Gramfort, V. Michel, B. Thirion, O. Grisel, M. Blondel, P. Prettenhofer, R. Weiss, V. Dubourg, J. Vanderplas, A. Passos, D. Cournapeau, M. Brucher, M. Perrot, and E. Duchesnay, Journal of Machine Learning Research **12**, 2825 (2011).
[47] D. Dua and C. Graff, "UCI machine learning repository," (2017).
[48] Y. LeCun and C. Cortes, (2010).
